# Dysfunctional Network and Mutation Genes of Hypertrophic Cardiomyopathy

**DOI:** 10.1155/2022/8680178

**Published:** 2022-01-28

**Authors:** Yunwen Cui, Cheng Liu, Jian Luo, Jie Liang

**Affiliations:** The First Affiliated Hospital of Xinjiang Medical University, No. 1 Liyushan Road, Xinshi, Urumqi 830054, China

## Abstract

**Background:**

Hypertrophic cardiomyopathy (HCM) is a group of heterogeneous diseases that affects the myocardium. It is also a common familial disease. The symptoms are not common and easy to find.

**Objective:**

In this paper, we aim to explore and analyze the dysfunctional gene network related to hypertrophic cardiomyopathy, and the key target genes with diagnostic and therapeutic significance for HCM were screened.

**Methods:**

The gene expression profiles of 37 samples (GSE130036) were downloaded from the GEO database. Differential analysis was used to identify the related dysregulated genes in patients with HCM. Enrichment analysis identified the biological function and signaling pathway of these differentially expressed genes. Then, PPI network was built and verified in the GSE36961 dataset. Finally, the gene of single-nucleotide variants (SNVs) in HCM samples was screened by means of maftools.

**Results:**

In this study, 920 differentially expressed genes were obtained, and these genes were mainly related to metabolism-related signaling pathways. 187 interacting genes were identified by PPI network analysis, and the expression trends of C1QB, F13A1, CD163, FCN3, PLA2G2A, and CHRDL2 were verified by another dataset and quantitative real-time polymerase chain reaction. ROC curve analysis showed that they had certain clinical diagnostic ability, and they were the potential key dysfunctional genes of HCM. In addition, we found that PRMT5 mutation was the most frequent in HCM samples, which may affect the pathogenesis of HCM.

**Conclusion:**

Therefore, the key genes and enrichment results identified by our analysis may provide a reference for the occurrence and development mechanism of HCM. In addition, mutations in PRMT5 may be a useful therapeutic and diagnostic target for HCM. Our results also provide an independent quantitative assessment of functional limitations in patients with unknown history.

## 1. Introduction

Hypertrophic cardiomyopathy (HCM) is a common inherited cardiovascular disease, which exists in one patient in every 500 people [1, 2]. Recently, the awareness of the disease had been improved in clinical practice [3]. However, the diagnosis of HCM was often misdiagnosed as asthma, anxiety, mitral valve prolapse, and coronary artery disease [4]. The clinical manifestations of hypertrophic cardiomyopathy were various, with a natural history [5]. However, once correctly diagnosed, patients with hypertrophic cardiomyopathy could be effectively managed to improve symptoms and survival.

At present, echocardiography, cardiac magnetic resonance, and other imaging methods were important for the diagnosis, treatment, and risk stratification of HCM, but the accuracy of detection was relatively low [6]. Patients with hypertrophic cardiomyopathy were relieved by various physical exercise methods, which had made significant contributions to disease management and were carried out without increasing risk [7]. Surgical treatment was the first choice for HCM, but due to the high morbidity and mortality in the early stage of the disease, some clinicians were still hesitant [8, 9].

In recent years, research on complex molecular pathophysiology of hypertrophic cardiomyopathy had increased rapidly [10]. Gene testing had become more accessible and was increasingly being included in the care of patients with hypertrophic cardiomyopathy [11]. It was worth noting that HCM has many genotypes and phenotypic variations [12, 13]. Hypertrophic cardiomyopathy was associated with more than 1400 mutations in 11 or more genes encoding cardiac sarcolemmal protein [14]. In most cases, the disease was caused by a single heterozygous mutation, with few (3% to 5%) multiple mutations leading to particularly severe hypertrophy and more adverse events [15]. Increasingly, genetic testing reports provided an assessment of the pathogenicity of all identified genes [16]. By identifying the differentially expressed genes between HCM and control, candidate target genes of HCM could be screened. Among the DEGs, the mRNA KRT1 with the largest upregulation multiple and the mRNA CYP1A1 with the largest downregulation multiple may play an important role in the disease. Note that KRT1 expression increased in patients with heart failure and cardiac pressure overload [17, 18]. It had been reported that severe cytochrome P450 (P450) enzymes had been altered during cardiac hypertrophy [19]. However, contrary to our results, 3-methylcholanthrene and benzo(a)pyrene-induced cardiac hypertrophy increased the expression of CYP1A1 [20].

In this paper, we analyzed the dysfunctional gene network related to hypertrophic cardiomyopathy and screened the key target genes with diagnostic and therapeutic significance for HCM. The results of gene mutation analysis may be helpful to the genetic detection of the subjects and their families. Furthermore, the results of this study can help to improve the diagnosis and distinguish between relatives at risk and those without risk.

## 2. Materials and Methods

### 2.1. GEO Dataset and Differentially Expressed Genes (DEGs)

The gene expression datasets GSE130036 and GSE36961 were downloaded from the GEO database (http://www.ncbi.nlm.nih.gov/geo/) [21]. The GSE130036 series (GPL20795 platform) contained a total of 37 myocardial tissue samples (28 HCM patients and 9 healthy control donors). The expression of the transcripts was quantified with kallisto v0.43.1 under default settings. The GSE36961 series (GPL15389 platform) contained a total of 145 cardiac tissue samples (106 HCM patients and 39 healthy control donors). The normalized Fastlo and transformed log2 were performed for normalized data.

The limma package was used in R to screen DEGs between HCM and healthy control samples. A threshold of twofold change and *P* value <0.05 were set for DEGs in this study.

### 2.2. Functional and Pathway Enrichment Analyses of DEGs

To explore the biological characteristics of these DEGs, gene ontology (GO) was performed with clusterProfiler R package. Then, ClueGO plugin of Cytoscape was used to perform the Kyoto Encyclopedia of Genes and Genomes (KEGG) pathway for DEGs. Significant results were determined with a *P* value <0.05.

### 2.3. Construction of the PPI Network

To establish the PPI network of all identified DEGs, the STRING (version 10.5) (http://string-db.org/) online database was used [22]. The parameter was set as medium confidence >0.4. To visualize the PPI network, Cytoscape software was used to draw their interactions. ROC curve was plotted, and AUC was calculated with “pROC” R package.

### 2.4. Single-Nucleotide Variants (SNVs)

maftools R package [23] was used to analyze the SNVs in HCM samples. maftools is implemented as an open-source R package and available as a part of the Bioconductor project. Visualization modules in maftools were generated using the ComplexHeatmap Bioconductor package.

#### 2.4.1. Sample Collection and Quantitative Real-Time Polymerase Chain Reaction (RT-PCR)

The blood samples were collected from 5 HCM patients and 5 healthy controls. This study was approved by the Medical Ethics Committee of the First Affiliated Hospital of Xinjiang Medical University. All patients and their families signed the informed consent. Total RNA was isolated with TRIzol (Thermo Fisher) and quantified by NanoDrop. cDNA was created using the cDNA synthesis kit (Invitrogen). cDNA was transcribed into DNA through SYBR Green PCR Master Mix (Thermo Fisher) using a real-time PCR machine (Applied Biosystems). The resulting Ct values were relative to a GAPDH reference gene, and 2^−ΔΔCt^ was obtained. Please see Table 1 for primer sequences.

## 3. Results

### 3.1. Differentially Expressed Genes in Hypertrophic Cardiomyopathy

**Figure 1:**
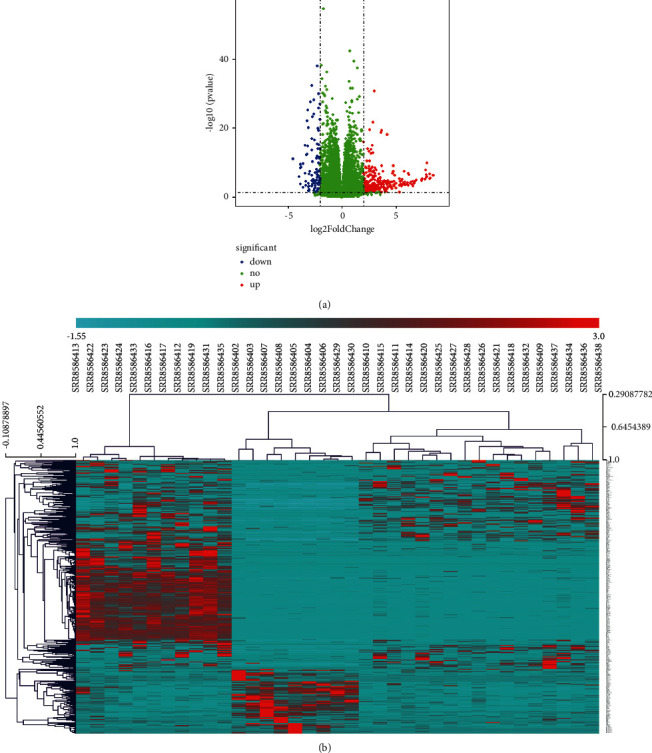
The differentially expressed genes between hypertrophic cardiomyopathy patients and controls. (a) Volcano plot of differentially expressed genes between 28 HCM patients and 9 controls in GSE130036. Red dots represent upregulated genes, and blue dots represent downregulated genes. (b) Thermogram of differentially expressed genes between 28 HCM patients and 9 controls in GSE130036. The node color changes from blue to red, indicating that the gene expression level changes from low to high.

The difference of gene expression between patients in the disease state and healthy people may be closely related to the disease. The data of GSE130036 were analyzed in the GEO database to explore the genes related to hypertrophic cardiomyopathy. By setting the screening threshold twofold, 920 differentially expressed genes (DEGs) were identified between hypertrophic cardiomyopathy and the healthy controls (Table S1). There were 636 upregulated genes with high expression levels and 284 downregulated genes with low expression levels (Figures 1(a) and 1(b)). It was important to compare these differentially expressed genes with those of HCM and control groups in GSE36961 data. We found 18 common differentially expressed genes (Table 2). It suggested that the DEGs between HCM and the control may be dysfunctional genes related to hypertrophic cardiomyopathy.

### 3.2. Mechanism of Dysregulation Related to Differentially Expressed Genes

**Figure 2:**
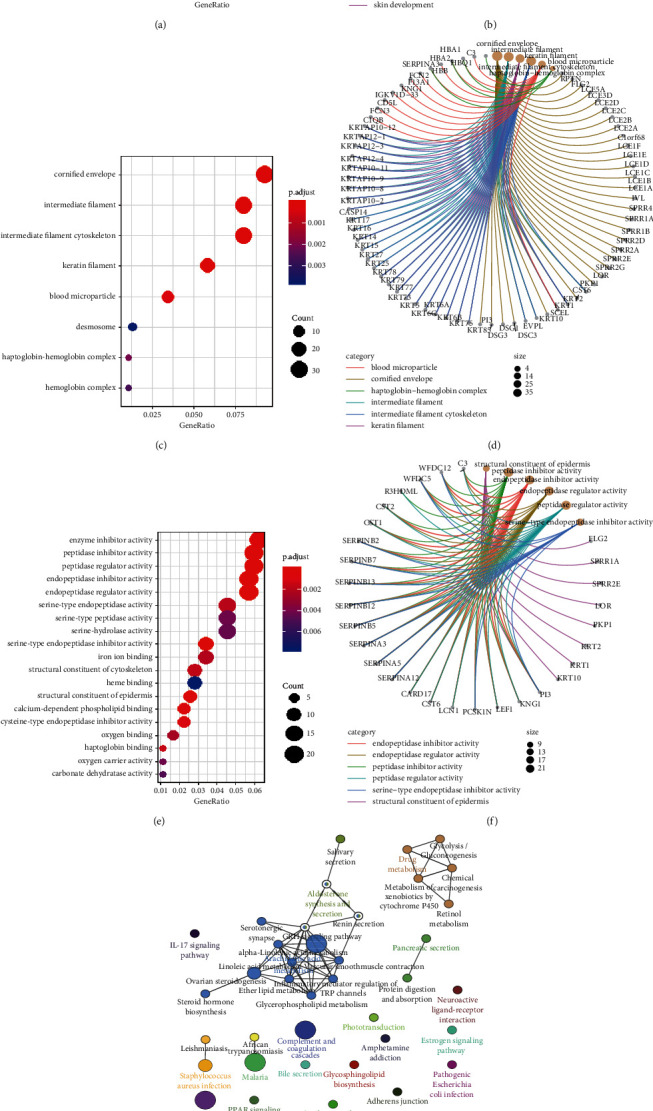
GO and KEGG enrichment of differentially expressed genes. (a, b) The biological process which differentially expressed genes participate in. (c, d) The cellular component which differentially expressed genes participate in. (e, f) The molecular function which differentially expressed genes participate in. The larger the dot is, the more genes are involved. (g, h) The KEGG pathway enrichment of differentially expressed genes.

To explore the biological functions of these dysfunctional genes in HCM, the enrichment of DEG with GO and KEGG was analyzed. From the results of biological process (BP) enrichment, we found that the maladjusted genes are mainly involved in the biological process related to epidermis development (Figures 2(a) and 2(b)). In addition, in the enrichment results of cell component (CC), the maladjusted genes were mainly related to the hemoglobin complex (Figures 2(c) and 2(d)). In the molecular function (MF), the maladjusted genes were mainly enriched in the molecular activity-related molecular function (Figures 2(e) and 2(f)). On the contrary, we found that the signal pathways involved in the maladjusted genes mainly include arachidonic acid metabolism, aldosterone synthesis and secretion, and drug metabolism (Figures 2(g) and 2(h)). The results showed that the maladjusted genes were mainly related to the metabolism-related signaling pathway in the course of HCM.

### 3.3. PPI Network Identification of Key Dysregulated Molecules

**Figure 3:**
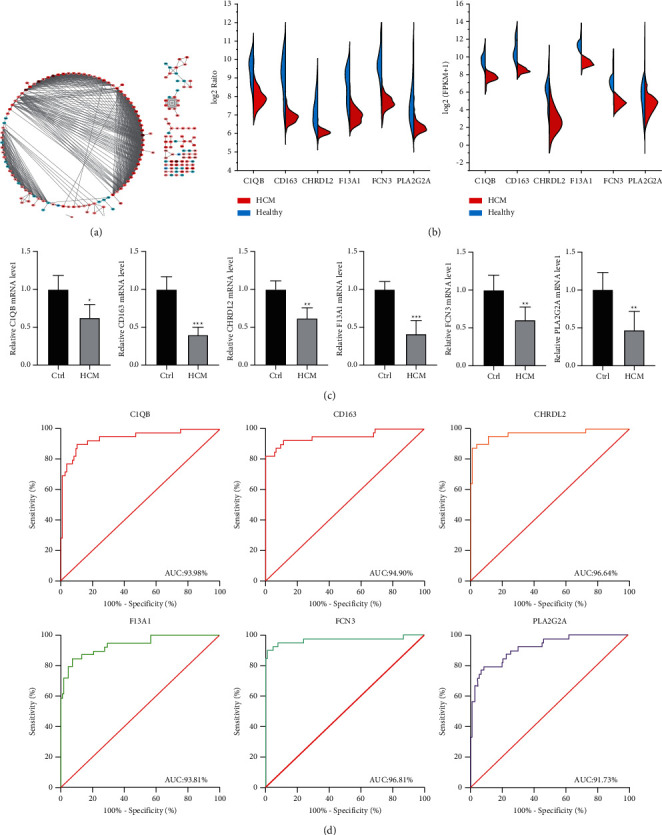
PPI network construction and identification of hub genes. (a) PPI network of DEGs. (b) The expression of six common differentially expressed genes in the PPI network in two groups of data. (c) Differential expression of key genes was verified with qRT-PCR. ^*∗*^*P* < 0.05, ^*∗∗*^*P* < 0.01, and ^*∗∗∗*^*P* < 0.001. (d) ROC curves of six genes with common differential expression.

To provide the contents and ways of DEG participating in cell biological activities, we constructed a full view of their interacting proteins to elucidate their functional networks. After mapping upregulated and downregulated genes into the network, the PPI network of 187 dysregulated genes (Figure 3(a)) was screened. Currently, we mapped 18 common differentially expressed genes into the PPI network. Six genes were screened, including C1QB, F13A1, CD163, FCN3, PLA2G2A, and CHRDL2. Their expression in the two groups of data was similar, with consistent downregulation behavior (Figure 3(b)). Importantly, we validated the differential expression of key genes in blood samples of HCM patients and controls (Figure 3(c)). In addition, ROC analysis showed that the AUC values of the six genes were all over 91%, indicating that they had a certain clinical diagnostic ability (Figure 3(d)). Therefore, we thought the six genes may be potential biological target genes of HCM.

### 3.4. Regulatory Mechanism of HCM Influenced by Mutation

**Figure 4:**
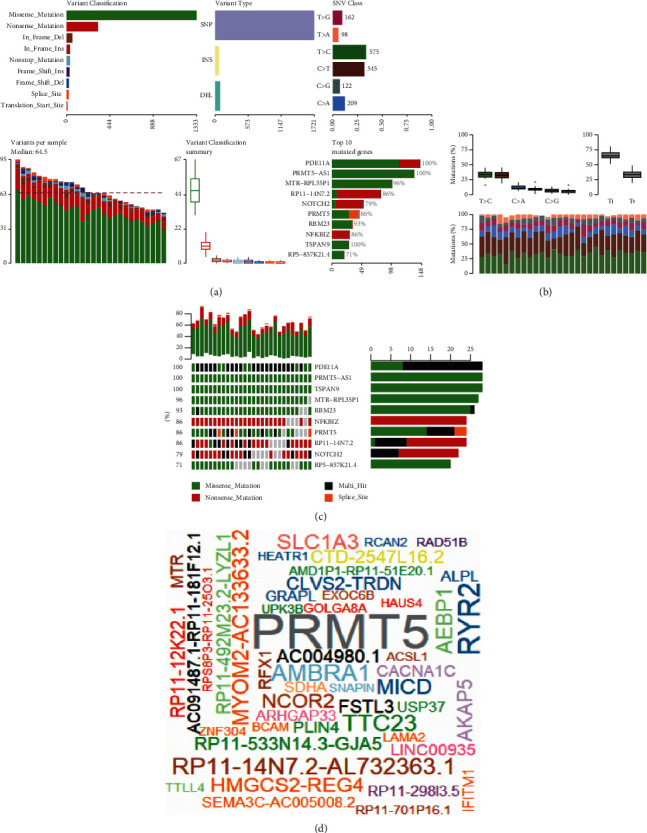
Mutations of the expressed genes in HCM samples. (a) The summary of the mutations in all samples. (b) Mutations in the top 10 genes. Genes are ordered by their mutation frequency and the mutation rate in the samples. (c) Transition and transversion plot displaying the distribution of SNVs in HCM classified into six transition and transversion events. Stacked bar plot shows the distribution of mutation spectra for every sample. (d) Word cloud plot for mutated genes. Size of each gene is proportional to the total number of samples in which it is mutated.

Using gene cohorts in GSE130036, we identified mutations in key genes generated with maftools (Table S2 and Figure 4(a)). The SNVs in the sample could be divided into six categories (Figure 4(b)). Thus, we presented the mutation types and frequencies of the first 10 mutations (Figure 4(c)), including PDE11A, PRMT5−AS1, TSPAN9, MTR−RPL35P1, RBM23, NFKBIZ, PRMT5, RP11−14N7.2, NOTCH2, and RP5−857K21.4. In addition, we found that the total number of samples with the PRMT5 mutation was the largest (Figure 4(d)). It is suggested that the mutation frequency of HCM is the highest, which may have a wider impact.

## 4. Discussion

Clarifying the exact genetic cause of hypertrophic cardiomyopathy can improve the level of clinical management. This study was based on the genes expressed in HCM patients, combined with network analysis, to identify the key genes. We also analyzed the single-nucleotide variants of genes expressed in HCM patients to explore the molecular mechanism of HCM disease.

Furthermore, enrichment analysis showed that HCM-related dysregulated genes were mainly involved in epidermis development and other related biological functions and metabolism-related signaling pathways. Similar to our analysis results, the differentially expressed genes were also related to epidermis development in the dilated cardiomyopathy [24]. In addition, the mechanism of metabolism had an important influence on the pathophysiological process of HCM [25]. Studies had shown that the decrease of left ventricular systolic function in female HCM mice was related to the decrease of activities of fatty acid transporter (CD36) and AMP-activated protein kinase (AMPK) [26].

On the contrary, through the PPI network of differentially expressed genes, we identified the dysregulated genes with interaction. Among them, we found six genes verified by GSE36961 data, and their expression levels were downregulated. In recent research results, F13A1 expression changes in the myocardial infection model of pigs [27]. HCM was the result of cardiac remodeling caused by myocardial cell injury. Macrophages participate in this process by maintaining the inflammatory and fibrogenic environment [28]. CD163 promoted the transformation of macrophages from M1 to M2 phenotype and released anti-inflammatory factors to solve inflammation [29]. In our analysis, CD163 was downregulated in HCM, which may be a key factor in the persistent inflammatory environment of HCM. However, the expression of PLA2G2A decreased in patients with dilated cardiac pathway [30]. It was reported that the occurrence of cardiovascular disease is closely related to PLA2G2A [31].

Most HCM cases were caused by mutations in the oncoprotein coding gene in Mendel's autosomal dominant genetic pattern [32]. In HCM patients, gene detection of these genes was of great value for diagnosis and early recognition of individuals at risk [33, 34]. However, new HCM-related mutations were being discovered, and more genes need to be found. PRMT5 is known as type II PRMT, which can produce monomethylarginine and symmetric dimethylarginine [35]. PRMT5 directly methylated GATA-4 transcription factor and inhibited phenylephrine-induced hypertrophy of neonatal rat ventricular myocytes [36]. Therefore, the HCM samples used in this study may be related to PRMT5 mutations. PDE11A had been proved to be an important gene which contains clinically significant variants [37]. The identification of pathogenic mutation genes was helpful for the determination of clinical diagnosis. Gene expression data may also help guide the use of new therapies. Thus, the results of this study may be conducive to guide the treatment of hypertrophic cardiomyopathy and provide an independent quantitative assessment of functional limitations in patients with an unknown history.

## 5. Conclusion

In this study, the key dysfunctional genes C1QB, F13A1, CD163, FCN3, PLA2G2A, and CHRDL2 were identified by studying the network of differentially expressed genes between HCM and healthy controls. Enrichment analysis showed that the molecular mechanism of HCM was related to the metabolism-related signaling pathway. Interestingly, we found that PRMT5 was the gene with the highest mutation rate in HCM samples, indicating that PRMT5 may have an important impact on the pathogenesis of HCM. However, this study still needs a lot of experiments to verify the analysis results. The limitation of this study is that the research object is developmental diseases. No clinical data can be used to correct the origin, age, and gender of the patients. Generalization and applicability to specific groups are restrictive. In addition, there is a lack of clinical samples to validate important analysis results.

## Figures and Tables

**Table 1:** The primers in this study.

Genes	Primers
GAPDH	F: 5′-TGTGGGCATCAATGGATTTGG-3′
	R: 5′-ACACCATGTATTCCGGGTCAAT-3′

C1QB	F: 5′-AAGGTGCCCGGTCTCTACTA-3′
	R: 5′-ACCTGGAAGGTGTTGTAGGC-3′

F13A1	F: 5′-TGCTGGTGTCTTTAACACATTTTTAA-3′
	R: 5′-TGGGCCGAGAAGTAATTGGT-3′

CD163	F: 5′-ATGGGTGGACACAGAATGGTT-3′
	R: 5′-CAGGAGCGTTAGTGACAGCAG-3′

FCN3	F: 5′-CCCAGTCTTTTGTGACATGGA-3′
	R: 5′-CCTGCTCTGTAGGAGGACCA-3′

PLA2G2A	F: 5′-GCACTCAGTTATGGCTTCT-3′
	R: 5′-ATTGTAGGTCGTCTTGTTTC-3′

CHRDL2	F: 5′-CTGGCACCCCTACTTGGAG-3′
	R: 5′-GCGGTAACAACTCACATGGG-3′

**Table 2:** The common differentially expressed genes in GSE130036 and GSE36961.

Symbol	GSE130036		GSE36961	
	log2FoldChange	*P* value	logFC	*P* value
CENPA	2.428995	1.71*E* − 06	1.699106	2.22*E* − 18
TUBA3E	−3.20702	8.72*E* − 23	−2.17305	3.12*E* − 47
TUBA3D	−3.1646	6.82*E* − 26	−2.20486	2.59*E* − 44
CORIN	−3.2704	8.28*E* − 13	−1.56596	5.15*E* − 11
SLITRK4	3.644176	1.91*E* − 19	1.260442	1.38*E* − 14
CA3	2.876428	5.99*E* − 08	1.362298	9.87*E* − 13
LYVE1	−2.0283	9.92*E* − 28	−1.87319	2.62*E* − 39
METTL7B	−2.17555	6.86*E* − 20	−1.35577	6*E* − 23
SERPINA3	−3.81907	4.52*E* − 09	−3.56141	3.7*E* − 42
RASD1	−2.08726	6.49*E* − 12	−3.51434	2.45*E* − 45
SLCO4A1	−2.59057	1.24*E* − 24	−1.00128	1.37*E* − 32
COMP	2.861793	8.22*E* − 07	1.099086	7.8*E* − 09
C1QB	−2.15322	1.30*E* − 30	−1.46863	4.93*E* − 28
F13A1	−2.29595	1.12*E* − 38	−1.5787	3.76*E* − 30
CD163	−2.76903	4.77*E* − 33	−2.13651	1.05*E* − 37
FCN3	−2.50434	2.72*E* − 17	−2.03805	4.98*E* − 40
PLA2G2A	−2.93842	3.35*E* − 08	−1.35137	2.51*E* − 20
CHRDL2	−3.62053	2.89*E* − 10	−1.17134	1.47*E* − 29

## Data Availability

The datasets used and/or analyzed during the current study are available from the corresponding author upon reasonable request.

## Abbreviations

HCM: Hypertrophic cardiomyopathy
SNVs: Single-nucleotide variants
GO: Gene ontology
KEGG: Kyoto Encyclopedia of Genes and Genomes
DEGs: Differentially expressed genes
BP: Biological process
CC: Cell component
MF: Molecular function.

## Supplementary Materials

Supplementary Materials Table S1: differentially expressed genes between HCM patients and the control group. Table S2: SNVs in HCM patients.
