# Supplementary material for: Dysfunctional Network and Mutation Genes of Hypertrophic Cardiomyopathy
Source: J Healthc Eng. 2022 Jan 28;2022:8680178. doi: 10.1155/2022/8680178 (PMC8816546; doi:10.1155/2022/8680178)
Supplement: Supplementary Materials — Table S1: differentially expressed genes between HCM patients and the control group. Table S2: SNVs in HCM patients. [file 8680178.f1.zip › 8680178.f1/Table S1.docx]

**Table S1. Differentially expressed genes between HCM patients and control group.**

| ID | baseMean | log2FoldChange | pvalue | symbol | type |
| --- | --- | --- | --- | --- | --- |
| ENSG00000186891 | 3.032622498 | 2.20814575 | 0.000788897 | TNFRSF18 | protein_coding |
| ENSG00000233542 | 1.079737675 | 2.760169728 | 0.001420196 | RP11-547D24.1 | antisense |
| ENSG00000227589 | 1.156715608 | 2.170560955 | 0.013390966 | RP5-1092A11.5 | antisense |
| ENSG00000116254 | 29.75622628 | 2.316135713 | 5.19E-11 | CHD5 | protein_coding |
| ENSG00000069812 | 2.390519291 | 4.248112036 | 2.80E-05 | HES2 | protein_coding |
| ENSG00000131686 | 2.174462827 | 2.139011266 | 0.014218064 | CA6 | protein_coding |
| ENSG00000224904 | 1.086515236 | 2.125009275 | 0.013104071 | RP5-934G17.6 | pseudogene |
| ENSG00000188984 | 1.022400183 | 3.025696361 | 0.025758337 | AADACL3 | protein_coding |
| ENSG00000231606 | 1.494528466 | -2.604019048 | 6.79E-06 | RP11-344F13.1 | lincRNA |
| ENSG00000233421 | 7.359082933 | -3.180418435 | 3.09E-17 | U1 | lincRNA |
| ENSG00000228549 | 10.90700413 | -3.033406039 | 5.62E-21 | U1 | lincRNA |
| ENSG00000142623 | 0.771553241 | 2.218914438 | 0.056817078 | PADI1 | protein_coding |
| ENSG00000009709 | 1.086103849 | 3.122122101 | 0.012112483 | PAX7 | protein_coding |
| ENSG00000188257 | 73.57547307 | -2.938420864 | 3.35E-08 | PLA2G2A | protein_coding |
| ENSG00000173369 | 371.8772724 | -2.153215884 | 1.30E-30 | C1QB | protein_coding |
| ENSG00000176092 | 3.477526251 | 2.413546089 | 0.000357428 | AIM1L | protein_coding |
| ENSG00000176083 | 2.329331851 | 2.025700238 | 0.010478955 | ZNF683 | protein_coding |
| ENSG00000175793 | 52.88862876 | 5.787745015 | 4.90E-08 | SFN | protein_coding |
| ENSG00000142748 | 64.79560048 | -2.504341296 | 2.72E-17 | FCN3 | protein_coding |
| ENSG00000174950 | 0.573634458 | 2.207417454 | 0.056773044 | CD164L2 | protein_coding |
| ENSG00000274978 | 1.455113197 | 2.524320134 | 0.004759674 | RNU11 | snRNA |
| ENSG00000189280 | 5.582140171 | 2.170957406 | 0.00014414 | GJB5 | protein_coding |
| ENSG00000284623 | 2.20386845 | 3.817863351 | 1.62E-06 | LINC02786 | LncRNA |
| ENSG00000253313 | 1.259860604 | 2.613742096 | 0.003310832 | C1orf210 | protein_coding |
| ENSG00000200169 | 39.50581163 | 3.322300913 | 8.72E-23 | RNU5D-1 | snRNA |
| ENSG00000142973 | 227.571142 | -3.404775802 | 1.28E-15 | CYP4B1 | protein_coding |
| ENSG00000154198 | 50.29707552 | -2.210513911 | 8.93E-06 | CYP4Z2P | pseudogene |
| ENSG00000187048 | 32.05831926 | -2.094620331 | 2.57E-05 | CYP4A11 | protein_coding |
| ENSG00000259832 | 1.636682969 | -2.280398592 | 0.001527015 | CYP4A26P | pseudogene |
| ENSG00000261196 | 0.480122829 | -2.237651563 | 0.013962764 | CYP4A43P | pseudogene |
| ENSG00000261593 | 2.005495945 | -2.137310537 | 0.000184859 | CYP4A27P | pseudogene |
| ENSG00000186160 | 13.7564983 | -2.136132314 | 4.65E-08 | CYP4Z1 | protein_coding |
| ENSG00000162366 | 5.052914182 | 2.864536915 | 0.0032732 | PDZK1IP1 | protein_coding |
| ENSG00000279096 | 113.2688328 | 3.269653778 | 3.84E-05 | AL356289.2 | TEC |
| ENSG00000225623 | 2.87708404 | -2.104763353 | 0.001508903 | AGBL4-IT1 | sense_intronic |
| ENSG00000237478 | 1.792808008 | -2.144965669 | 0.000785145 | RP5-926E3.1 | pseudogene |
| ENSG00000279324 | 3.433128915 | -2.186728868 | 0.000134844 | AL645730.1 | TEC |
| ENSG00000284700 | 0.870906201 | 2.116992535 | 0.022063723 | AL049637.2 | lncRNA |
| ENSG00000184292 | 43.08307771 | 4.680825592 | 1.40E-07 | TACSTD2 | protein_coding |
| ENSG00000240563 | 7.161989109 | 2.045908456 | 1.97E-05 | L1TD1 | protein_coding |
| ENSG00000213579 | 13.08764875 | 2.273010644 | 6.17E-07 | RP4-595K12.1 | pseudogene |
| ENSG00000225605 | 1.930457353 | 3.29867595 | 7.60E-05 | RP11-550H2.1 | lincRNA |
| ENSG00000202263 | 0.859515785 | 2.416446978 | 0.01057269 | RNA5SP22 | rRNA |
| ENSG00000137975 | 13.84033737 | 3.575130356 | 3.12E-06 | CLCA2 | protein_coding |
| ENSG00000271578 | 1.344006536 | 2.037193727 | 0.021699261 | RP5-1033H2.1 | pseudogene |
| ENSG00000060718 | 6.027476328 | 3.474413556 | 2.70E-07 | COL11A1 | protein_coding |
| ENSG00000121933 | 7.937667264 | -2.250712673 | 7.54E-08 | ADORA3 | protein_coding |
| ENSG00000282608 | 17.32221821 | -2.537008958 | 1.04E-17 | ADORA3 | protein_coding |
| ENSG00000271810 | 0.369526715 | -2.056384487 | 0.099161606 | RP11-426L16.10 | protein_coding |
| ENSG00000177551 | 2.22142966 | 3.500243007 | 3.36E-05 | NHLH2 | protein_coding |
| ENSG00000203859 | 1.610528777 | 3.009145734 | 0.000383945 | HSD3B2 | protein_coding |
| ENSG00000275538 | 33.00349533 | 2.384705838 | 7.76E-12 | RNVU1-19 | protein_coding |
| ENSG00000232721 | 0.71432375 | 2.520854928 | 0.015874204 | RP11-403I13.5 | lincRNA |
| ENSG00000274428 | 3.057855644 | 2.273441704 | 0.000514329 | RF00003 | protein_coding |
| ENSG00000215853 | 1.518093074 | 3.243271727 | 0.001116799 | RPTN | protein_coding |
| ENSG00000143520 | 367.2745453 | 2.863041407 | 2.25E-06 | FLG2 | protein_coding |
| ENSG00000186207 | 7.025099309 | 4.841375916 | 0.000695854 | LCE5A | protein_coding |
| ENSG00000169509 | 8.713937957 | 6.128232732 | 0.000527335 | CRCT1 | protein_coding |
| ENSG00000185966 | 0.574887938 | 2.208381711 | 0.105952618 | LCE3E | protein_coding |
| ENSG00000163202 | 3.013268748 | 4.590257807 | 0.001796561 | LCE3D | protein_coding |
| ENSG00000285753 | 2.119760476 | 4.088201969 | 0.003558276 | AL139247.1 | protein_coding |
| ENSG00000187223 | 5.744408299 | 5.526755712 | 0.000981172 | LCE2D | protein_coding |
| ENSG00000187180 | 11.52468218 | 6.532101212 | 0.000319816 | LCE2C | protein_coding |
| ENSG00000159455 | 26.33151554 | 7.7242805 | 9.74E-06 | LCE2B | protein_coding |
| ENSG00000187173 | 6.729860152 | 5.755377079 | 0.000378331 | LCE2A | protein_coding |
| ENSG00000198854 | 28.62168459 | 7.844581356 | 2.81E-06 | C1orf68 | protein_coding |
| ENSG00000203786 | 29.34277639 | 7.880438594 | 1.51E-06 | KPRP | protein_coding |
| ENSG00000240386 | 8.036948125 | 6.011544942 | 0.00027244 | LCE1F | protein_coding |
| ENSG00000186226 | 1.878963446 | 3.907244982 | 0.001534054 | LCE1E | protein_coding |
| ENSG00000172155 | 3.153939672 | 4.662270154 | 0.005172957 | LCE1D | protein_coding |
| ENSG00000197084 | 21.81580307 | 7.452736982 | 5.23E-06 | LCE1C | protein_coding |
| ENSG00000196734 | 13.43839677 | 6.753588841 | 3.68E-05 | LCE1B | protein_coding |
| ENSG00000186844 | 13.24523018 | 6.732743976 | 9.55E-05 | LCE1A | protein_coding |
| ENSG00000235942 | 5.46970843 | 5.455880144 | 0.000240893 | LCE6A | protein_coding |
| ENSG00000163207 | 12.02662031 | 6.593124834 | 3.27E-05 | IVL | protein_coding |
| ENSG00000283227 | 4.162030164 | 5.061486986 | 0.000960232 | SPRR5 | protein_coding |
| ENSG00000184148 | 2.022782882 | 4.019038437 | 0.011397986 | SPRR4 | protein_coding |
| ENSG00000169474 | 4.007353767 | 5.007505711 | 0.003800549 | SPRR1A | protein_coding |
| ENSG00000169469 | 11.95340895 | 6.584677064 | 0.000763417 | SPRR1B | protein_coding |
| ENSG00000163216 | 5.130888408 | 5.362738934 | 0.000554921 | SPRR2D | protein_coding |
| ENSG00000241794 | 4.977403492 | 5.320029109 | 0.049555405 | SPRR2A | protein_coding |
| ENSG00000196805 | 2.022121496 | 4.017735378 | 0.060633527 | SPRR2B | protein_coding |
| ENSG00000203785 | 35.11141361 | 8.139466458 | 6.24E-06 | SPRR2E | protein_coding |
| ENSG00000159516 | 28.25804119 | 7.826146795 | 4.57E-07 | SPRR2G | protein_coding |
| ENSG00000203783 | 1.38257143 | 3.468085658 | 0.051372694 | PRR9 | protein_coding |
| ENSG00000203782 | 142.7585863 | 24.89968102 | 4.87E-38 | LOR | protein_coding |
| ENSG00000159527 | 1.631331625 | 3.697624671 | 0.001541028 | PGLYRP3 | protein_coding |
| ENSG00000143556 | 16.30090489 | 6.064088777 | 0.000317982 | S100A7 | protein_coding |
| ENSG00000196754 | 25.95585228 | 2.154990801 | 7.19E-06 | S100A2 | protein_coding |
| ENSG00000132698 | 6.214582546 | 5.312660145 | 4.79E-06 | RAB25 | protein_coding |
| ENSG00000073754 | 5.632113185 | -3.030030301 | 2.07E-07 | CD5L | protein_coding |
| ENSG00000158477 | 1.503779179 | 2.54062926 | 0.00752868 | CD1A | protein_coding |
| ENSG00000143217 | 10.65215965 | 2.635132861 | 0.003533217 | PVRL4 | protein_coding |
| ENSG00000162747 | 26.61982032 | 3.719312526 | 4.67E-20 | FCGR3B | protein_coding |
| ENSG00000259788 | 1.165921734 | -2.029838325 | 0.000383785 | RP11-359K18.3 | antisense |
| ENSG00000225487 | 0.347936966 | -2.173922828 | 0.085538308 | RP11-403P14.1 | pseudogene |
| ENSG00000236206 | 0.509568151 | -2.126492076 | 0.065975518 | RP11-306I1.2 | lincRNA |
| ENSG00000227579 | 2.695476301 | 3.126089112 | 0.000145645 | RP1-35C21.2 | lincRNA |
| ENSG00000236720 | 0.877589458 | 2.819733071 | 0.001646296 | RP11-63B19.1 | lincRNA |
| ENSG00000143340 | 4.141765272 | -2.032132137 | 4.06E-06 | FAM163A | protein_coding |
| ENSG00000081248 | 2.27442253 | 2.072224398 | 0.001857249 | CACNA1S | protein_coding |
| ENSG00000081277 | 140.049975 | 3.709964929 | 1.02E-09 | PKP1 | protein_coding |
| ENSG00000203710 | 52.01319972 | -2.046617534 | 1.48E-14 | CR1 | protein_coding |
| ENSG00000230937 | 3.707552279 | 4.892741 | 0.001047262 | MIR205HG | lincRNA |
| ENSG00000198570 | 0.802891949 | -3.324349302 | 0.000622244 | RD3 | protein_coding |
| ENSG00000232809 | 0.362264869 | -2.776515472 | 0.067671477 | VDAC1P10 | pseudogene |
| ENSG00000228247 | 0.316677191 | -2.067003811 | 0.144117008 | UBBP2 | pseudogene |
| ENSG00000238232 | 0.388297767 | -2.394329032 | 0.027609951 | RP11-95P13.2 | lincRNA |
| ENSG00000272865 | 1.619116339 | 2.027628116 | 0.006599154 | RP11-561I11.4 | antisense |
| ENSG00000213690 | 1.067304889 | 2.358221872 | 0.010623347 | RP11-513D4.1 | pseudogene |
| ENSG00000235403 | 1.135159484 | -2.039991836 | 0.001227395 | AC114808.3 | antisense |
| ENSG00000228391 | 0.667197448 | 2.413819961 | 0.015569682 | AC011995.3 | lincRNA |
| ENSG00000234423 | 3.961738018 | 2.311091777 | 8.64E-05 | AC019118.2 | lincRNA |
| ENSG00000240687 | 11.60997626 | 2.430386524 | 4.54E-10 | RP11-521D12.1 | lincRNA |
| ENSG00000233718 | 2.233831059 | 2.509921444 | 0.000700999 | MYCNOS | antisense |
| ENSG00000151379 | 0.647909684 | 2.016346081 | 0.021229081 | MSGN1 | protein_coding |
| ENSG00000237992 | 3.347416265 | -2.046064528 | 0.000321909 | AC010096.2 | lincRNA |
| ENSG00000115884 | 39.91208173 | 3.132901216 | 1.11E-06 | SDC1 | protein_coding |
| ENSG00000231204 | 4.907011961 | 2.504886328 | 8.47E-05 | AC011752.1 | lincRNA |
| ENSG00000228538 | 1.518580007 | 2.571637182 | 0.002611886 | AC009411.1 | lincRNA |
| ENSG00000115163 | 244.1016568 | 2.428994638 | 1.71E-06 | CENPA | protein_coding |
| ENSG00000189350 | 176.4091991 | -2.181923791 | 0.000161445 | FAM179A | protein_coding |
| ENSG00000236854 | 3.791310086 | 2.269771789 | 1.31E-05 | AL121656.5 | lincRNA |
| ENSG00000235064 | 1.386913236 | 2.070139389 | 0.01347935 | SLC25A5P2 | pseudogene |
| ENSG00000282828 | 2.685736086 | 3.444674001 | 0.000177377 | AC009971.1 | lncRNA |
| ENSG00000270335 | 1.059907868 | 2.406529741 | 0.005480465 | RP11-642D6.1 | pseudogene |
| ENSG00000223863 | 0.600232518 | 2.252382815 | 0.055568114 | AC008074.4 | lincRNA |
| ENSG00000286002 | 1.863209018 | -2.520743229 | 3.56E-05 | AC010987.1 | lncRNA |
| ENSG00000199460 | 1.54145403 | 2.606435639 | 0.002748246 | RNU6-1216P | snRNA |
| ENSG00000214429 | 1.192094531 | 2.898191415 | 0.01703036 | CYCSP6 | pseudogene |
| ENSG00000115386 | 0.254441481 | -2.470313207 | 0.469306839 | REG1A | protein_coding |
| ENSG00000233999 | 0.692397933 | 2.474343488 | 0.037230569 | IGKV3OR2-268 | IG_V_gene |
| ENSG00000222724 | 6.3073571 | 2.428719593 | 2.46E-07 | RNU2-63P | snRNA |
| ENSG00000244116 | 1.060030354 | -2.586532796 | 0.019012953 | IGKV2-28 | IG_V_gene |
| ENSG00000283132 | 0.559073866 | 2.151567251 | 0.215090999 | AC006453.1 | mRNA |
| ENSG00000239975 | 0.494121803 | -3.039557169 | 0.024042367 | IGKV1D-33 | IG_V_gene |
| ENSG00000233275 | 0.682405535 | 2.060428695 | 0.055367229 | AC009238.8 | pseudogene |
| ENSG00000284337 | 0.881698609 | 2.461495444 | 0.0235913 | AC013271.1 | pseudogene |
| ENSG00000125571 | 6.202045351 | 5.636696789 | 0.000194175 | IL37 | protein_coding |
| ENSG00000136688 | 1.246274253 | 3.30450234 | 0.00834561 | IL36G | protein_coding |
| ENSG00000136695 | 6.55637785 | 5.717284388 | 7.75E-05 | IL36RN | protein_coding |
| ENSG00000234148 | 0.95367667 | 2.969646295 | 0.003312005 | LINC01961 | lincRNA |
| ENSG00000163064 | 1.171113637 | 2.512889976 | 0.003445186 | EN1 | protein_coding |
| ENSG00000019169 | 18.7688611 | -2.625408878 | 8.92E-06 | MARCO | protein_coding |
| ENSG00000163083 | 109.3345662 | 2.009761111 | 1.60E-07 | INHBB | protein_coding |
| ENSG00000152086 | 70.7130884 | -3.207015567 | 8.72E-23 | TUBA3E | protein_coding |
| ENSG00000075886 | 155.7527459 | -3.164595813 | 6.82E-26 | TUBA3D | protein_coding |
| ENSG00000150551 | 8.724942456 | 2.254915883 | 1.10E-07 | LYPD1 | protein_coding |
| ENSG00000230065 | 0.993466054 | 2.291259403 | 0.02323129 | AC010974.3 | antisense |
| ENSG00000231567 | 34.84408603 | 2.005941531 | 0.064260912 | MTND2P19 | pseudogene |
| ENSG00000223921 | 32.56988879 | 2.558939237 | 0.006888294 | MTND1P27 | pseudogene |
| ENSG00000277306 | 3.393048071 | 3.060913044 | 0.000563173 | RF02271 | mRNA |
| ENSG00000150556 | 2.890139093 | 3.86280813 | 0.000364334 | LYPD6B | protein_coding |
| ENSG00000163331 | 4.811441886 | 4.614379696 | 6.38E-05 | DAPL1 | protein_coding |
| ENSG00000279844 | 1.094798399 | -2.035343824 | 0.000270779 | AC096656.1 | TEC |
| ENSG00000244372 | 0.362720902 | -2.068827079 | 0.037745436 | RN7SL423P | misc_RNA |
| ENSG00000227403 | 1.500401723 | 2.543420788 | 0.002921673 | AC009299.3 | lincRNA |
| ENSG00000144285 | 30.30759259 | -2.287260837 | 3.73E-07 | SCN1A | protein_coding |
| ENSG00000207402 | 2.329012819 | 2.344145618 | 0.00174417 | RNU6-959P | snRNA |
| ENSG00000237194 | 0.748312109 | 2.21099874 | 0.030248571 | SNAI1P1 | pseudogene |
| ENSG00000180871 | 14.02501852 | 2.240566137 | 4.68E-09 | CXCR2 | protein_coding |
| ENSG00000163464 | 6.199100795 | 2.985557412 | 1.11E-07 | CXCR1 | protein_coding |
| ENSG00000054356 | 2.018275703 | 2.104186466 | 0.004938087 | PTPRN | protein_coding |
| ENSG00000171951 | 13.59160222 | 2.105922749 | 7.88E-05 | SCG2 | protein_coding |
| ENSG00000115009 | 0.611925882 | -2.399877458 | 0.046615858 | CCL20 | protein_coding |
| ENSG00000270540 | 2.568374073 | 2.423831974 | 0.000327958 | RP11-785G17.1 | antisense |
| ENSG00000172478 | 2.712291679 | 4.101100668 | 4.30E-05 | C2orf54 | protein_coding |
| ENSG00000233026 | 0.473653556 | -2.179946012 | 0.134542807 | AC026166.2 | pseudogene |
| ENSG00000227260 | 1.647719286 | -2.090076849 | 0.001819919 | AC116035.1 | lincRNA |
| ENSG00000228112 | 0.668290035 | 2.038169511 | 0.073187896 | AC112220.2 | pseudogene |
| ENSG00000172995 | 1.433619879 | 2.478647821 | 0.00134623 | ARPP21 | protein_coding |
| ENSG00000230067 | 2.320654148 | 2.016383076 | 0.001603311 | HSPD1P6 | pseudogene |
| ENSG00000212145 | 1.205492149 | 2.574911929 | 0.002359998 | U8 | snoRNA |
| ENSG00000240203 | 0.856229812 | 2.418055377 | 0.013926268 | RN7SL567P | misc_RNA |
| ENSG00000283473 | 1.471958085 | 2.189336843 | 0.014750613 | FAM240A | FAM240A |
| ENSG00000200379 | 0.713174257 | 2.129219599 | 0.044963951 | RN7SKP45 | misc_RNA |
| ENSG00000277592 | 1.055133197 | 2.01971959 | 0.018033245 | RF00017 | RF00017 |
| ENSG00000241359 | 1.029485576 | 2.66498672 | 0.003057402 | SYNPR-AS1 | antisense |
| ENSG00000271034 | 0.816157958 | 2.334440726 | 0.015561114 | RP11-963H1.1 | pseudogene |
| ENSG00000163421 | 2.611703357 | 3.394945912 | 6.25E-06 | PROK2 | protein_coding |
| ENSG00000182921 | 1.503265167 | 3.250820994 | 0.000627594 | CCDC75P1 | pseudogene |
| ENSG00000242641 | 1.331269789 | 2.386012465 | 0.008471215 | LINC00971 | lincRNA |
| ENSG00000144820 | 1.972186017 | 2.36302026 | 0.005620903 | GPR128 | protein_coding |
| ENSG00000241469 | 1.805359629 | 2.219664971 | 0.008342701 | LINC00635 | lincRNA |
| ENSG00000241490 | 0.712020335 | 2.111927837 | 0.029046264 | RP11-553L6.2 | antisense |
| ENSG00000185565 | 351.0846939 | -2.048542447 | 1.07E-05 | LSAMP | protein_coding |
| ENSG00000239268 | 1.009238203 | -2.115054642 | 0.0011174 | RP11-384F7.2 | lincRNA |
| ENSG00000241213 | 1.347862245 | -2.094702479 | 0.00207393 | RP11-768G7.2 | lincRNA |
| ENSG00000242613 | 1.088683499 | -2.507054666 | 0.000588608 | RP11-174O3.4 | pseudogene |
| ENSG00000240774 | 4.996348859 | -2.884160108 | 2.23E-09 | RP11-359H3.4 | pseudogene |
| ENSG00000244441 | 4.041994188 | -3.342162074 | 3.96E-12 | RP11-359H3.1 | pseudogene |
| ENSG00000183770 | 0.750695565 | 2.196546509 | 0.049533203 | FOXL2 | protein_coding |
| ENSG00000243629 | 10.83241473 | -2.743586133 | 1.09E-21 | LINC00880 | lincRNA |
| ENSG00000241135 | 315.6952946 | -2.754595413 | 1.71E-35 | LINC00881 | lincRNA |
| ENSG00000174899 | 20.82930087 | -2.600627415 | 8.16E-29 | C3orf55 | protein_coding |
| ENSG00000182447 | 0.672965562 | 2.45146834 | 0.010113113 | OTOL1 | protein_coding |
| ENSG00000090402 | 0.511970268 | 2.021019502 | 0.075555256 | SI | protein_coding |
| ENSG00000283474 | 0.254393654 | -2.214632837 | 0.221200039 | MIR6828 | miRNA |
| ENSG00000113889 | 2.55737262 | -2.160238529 | 0.000125649 | KNG1 | protein_coding |
| ENSG00000226482 | 1.044425956 | -2.735895629 | 0.004843824 | ADIPOQ-AS1 | antisense |
| ENSG00000216058 | 0.685317286 | 2.119584456 | 0.032680869 | MIR944 | miRNA |
| ENSG00000230077 | 3.744360979 | 2.083438282 | 0.000165828 | MTAPP2 | pseudogene |
| ENSG00000163347 | 49.35452436 | 3.210615017 | 1.27E-08 | CLDN1 | protein_coding |
| ENSG00000114279 | 3211.610958 | -2.336371911 | 1.05E-08 | FGF12 | protein_coding |
| ENSG00000231383 | 140.0644093 | -2.28778243 | 8.51E-11 | FGF12-AS1 | antisense |
| ENSG00000230126 | 100.4698577 | -2.452372584 | 1.62E-09 | FGF12-AS2 | antisense |
| ENSG00000226709 | 91.1055747 | -2.594390989 | 1.13E-09 | FGF12-AS3 | antisense |
| ENSG00000238902 | 34.5092688 | -3.083854995 | 5.04E-10 | snoU13 | snoRNA |
| ENSG00000238031 | 1.525709651 | 2.595859536 | 0.002261802 | AC090505.1 | lincRNA |
| ENSG00000273238 | 0.510491698 | 2.020315301 | 0.122037708 | RP11-1263C18.1 | lincRNA |
| ENSG00000252002 | 0.904638738 | -2.200779752 | 0.00033234 | RNA5SP154 | rRNA |
| ENSG00000246095 | 0.844370562 | 2.060771467 | 0.027803353 | LINC01096 | lincRNA |
| ENSG00000137440 | 2.9436987 | 4.229503772 | 0.0042802 | FGFBP1 | protein_coding |
| ENSG00000250092 | 0.609358264 | 2.248944093 | 0.021544181 | RP11-556G22.3 | antisense |
| ENSG00000157765 | 1.295802373 | 2.70062496 | 0.012553336 | SLC34A2 | protein_coding |
| ENSG00000286141 | 1.706424254 | 3.767022567 | 8.06E-06 | AC097480.2 | lncRNA |
| ENSG00000248780 | 0.555422276 | 2.180044926 | 0.041871353 | RP11-632F7.1 | pseudogene |
| ENSG00000109132 | 0.59363538 | 2.238484074 | 0.040229633 | PHOX2B | protein_coding |
| ENSG00000178343 | 100.4344195 | -2.26329963 | 4.82E-16 | SHISA3 | protein_coding |
| ENSG00000239679 | 1.011248747 | 2.992990217 | 0.00164179 | RN7SL193P | misc_RNA |
| ENSG00000145244 | 1136.696039 | -3.270404851 | 8.28E-13 | CORIN | protein_coding |
| ENSG00000277388 | 6.922865056 | -3.594851769 | 8.64E-11 | MIR8053 | miRNA |
| ENSG00000242262 | 12.61867958 | -3.30673385 | 3.31E-15 | RP11-100N21.1 | pseudogene |
| ENSG00000282917 | 37.79316938 | -3.85882803 | 9.61E-18 | AC107068.2 | lncRNA |
| ENSG00000109182 | 2.835646356 | 4.50074705 | 4.54E-05 | CWH43 | protein_coding |
| ENSG00000109255 | 1.578223203 | 2.631324438 | 0.010093775 | NMU | protein_coding |
| ENSG00000280285 | 9.12822106 | -3.008196359 | 2.03E-09 | AC108215.1 | TEC |
| ENSG00000198092 | 2.691178551 | 2.091064647 | 0.000641485 | TMPRSS11F | protein_coding |
| ENSG00000249956 | 1.138579988 | 2.136098219 | 0.030772119 | RP11-790I12.2 | pseudogene |
| ENSG00000169435 | 0.793594017 | 2.310251822 | 0.018556197 | RASSF6 | protein_coding |
| ENSG00000163736 | 5.108553908 | 2.101904745 | 1.93E-05 | PPBP | protein_coding |
| ENSG00000124882 | 43.20315091 | -2.474452479 | 0.001893068 | EREG | protein_coding |
| ENSG00000250315 | 0.538531527 | 2.067345708 | 0.099832527 | RP11-44F21.4 | pseudogene |
| ENSG00000263445 | 1.797686005 | 3.182617397 | 0.000122613 | MIR4450 | miRNA |
| ENSG00000250325 | 1.015370567 | 2.692359358 | 0.001772374 | IGBP1P4 | pseudogene |
| ENSG00000163623 | 0.848770319 | 2.423706283 | 0.011988257 | NKX6-1 | protein_coding |
| ENSG00000258013 | 0.322727897 | -2.30147569 | 0.029346324 | RPL3P13 | pseudogene |
| ENSG00000196616 | 1023.978401 | -2.004073016 | 4.50E-23 | ADH1B | protein_coding |
| ENSG00000244470 | 1.468909659 | 2.183878052 | 0.015591062 | RP11-395M19.1 | pseudogene |
| ENSG00000138795 | 59.90800527 | 2.549469404 | 4.51E-20 | LEF1 | protein_coding |
| ENSG00000232021 | 9.32626452 | 2.927167332 | 3.20E-11 | LEF1-AS1 | processed_transcript |
| ENSG00000164089 | 85.28820505 | -2.760754432 | 3.27E-14 | ETNPPL | protein_coding |
| ENSG00000249519 | 2.189527704 | 2.499729463 | 0.002665273 | RP11-777N19.1 | lincRNA |
| ENSG00000248491 | 0.708725308 | 2.124224092 | 0.080651083 | RP11-125O18.1 | lincRNA |
| ENSG00000249618 | 0.693439829 | 2.105139463 | 0.066501074 | RP11-422J15.1 | lincRNA |
| ENSG00000248397 | 0.816557107 | -2.134100206 | 0.014983338 | LINC00498 | lincRNA |
| ENSG00000286133 | 3.040765668 | 2.487795583 | 9.02E-05 | AC093817.2 | lncRNA |
| ENSG00000234111 | 2.19461174 | 3.131245999 | 8.56E-05 | RP11-364P22.1 | lincRNA |
| ENSG00000200521 | 0.67842774 | 2.471628321 | 0.030867278 | Y_RNA | misc_RNA |
| ENSG00000249627 | 1.967956129 | 2.300671531 | 0.004761081 | RP11-312A15.1 | pseudogene |
| ENSG00000279703 | 2.184996846 | 3.1296405 | 7.33E-05 | AC017037.3 | TEC |
| ENSG00000279994 | 2.856477103 | 4.508437549 | 2.89E-09 | AC017037.4 | TEC |
| ENSG00000250475 | 2.637867865 | 3.411457979 | 1.13E-05 | RP11-312A15.3 | pseudogene |
| ENSG00000250488 | 1.683952335 | 3.09573733 | 0.000100372 | RP11-6C14.1 | lincRNA |
| ENSG00000168843 | 3.122056603 | 3.979707788 | 0.008071013 | FSTL5 | protein_coding |
| ENSG00000248396 | 0.822201788 | 2.349780024 | 0.039550987 | TOMM22P4 | pseudogene |
| ENSG00000249460 | 71.31296579 | -2.111840969 | 0.000249278 | RP11-665C14.2 | lincRNA |
| ENSG00000164362 | 2.475045691 | 3.963388583 | 3.08E-05 | TERT | protein_coding |
| ENSG00000249396 | 0.742270338 | 2.234665847 | 0.015358537 | RP11-1C1.4 | lincRNA |
| ENSG00000183654 | 26.00659854 | -2.141482103 | 8.88E-16 | 43535 | protein_coding |
| ENSG00000234865 | 0.71113351 | 2.111369886 | 0.025402585 | CTD-2138O14.1 | pseudogene |
| ENSG00000251273 | 0.555190954 | -2.310996277 | 0.078217574 | RP11-549K20.1 | lincRNA |
| ENSG00000251654 | 1.263369749 | -2.591466873 | 0.115554868 | RP11-192H6.2 | lincRNA |
| ENSG00000260761 | 0.735091 | -3.013718587 | 0.03037716 | RP11-184E9.2 | lincRNA |
| ENSG00000168671 | 1.141979367 | 2.131101484 | 0.025938475 | UGT3A2 | protein_coding |
| ENSG00000259663 | 1.857305492 | 2.573213415 | 0.00141777 | CTD-2314G24.2 | antisense |
| ENSG00000232517 | 1.762278678 | 2.450703332 | 0.004612108 | AC112198.1 | pseudogene |
| ENSG00000164283 | 102.1692737 | 4.177249213 | 9.43E-19 | ESM1 | protein_coding |
| ENSG00000164287 | 1.122172843 | 2.136391595 | 0.031342107 | CDC20B | protein_coding |
| ENSG00000164509 | 71.17610095 | 2.691285588 | 2.25E-14 | IL31RA | protein_coding |
| ENSG00000169067 | 1.188188018 | 2.211681202 | 0.010112643 | ACTBL2 | protein_coding |
| ENSG00000249584 | 2.243525026 | 2.18881834 | 0.001848634 | RP11-478P10.1 | lincRNA |
| ENSG00000213830 | 0.58802792 | 2.234636793 | 0.122879822 | CFL1P5 | pseudogene |
| ENSG00000273841 | 1.161240112 | 2.524706 | 0.03049364 | TAF9 | mRNA |
| ENSG00000249743 | 1.752999313 | 3.818932669 | 3.77E-05 | RP11-60A8.1 | lincRNA |
| ENSG00000248942 | 0.866694769 | 2.807382933 | 0.008423394 | CTD-2275D24.4 | lincRNA |
| ENSG00000248673 | 0.717285533 | 2.524110442 | 0.069669872 | CTC-419K13.1 | lincRNA |
| ENSG00000251078 | 1.547230268 | 2.96519387 | 0.000415109 | SLC25A5P9 | pseudogene |
| ENSG00000251419 | 1.210145998 | 2.615225091 | 0.001512014 | RP11-524L6.4 | pseudogene |
| ENSG00000206744 | 0.838789229 | 2.74198124 | 0.003087726 | RNU6-620P | snRNA |
| ENSG00000249958 | 1.818122025 | 2.202611301 | 0.004353881 | CCT7P2 | pseudogene |
| ENSG00000248528 | 1.674153107 | 3.76169855 | 9.40E-06 | CTC-458G6.2 | lincRNA |
| ENSG00000248966 | 2.74928027 | 2.134816277 | 0.002888391 | CTD-2089O24.1 | pseudogene |
| ENSG00000241888 | 0.450650518 | -2.483510123 | 0.071885634 | RPSAP37 | pseudogene |
| ENSG00000248107 | 4.59270484 | -2.052553933 | 0.000208012 | CTC-339D2.1 | lincRNA |
| ENSG00000249421 | 0.793276179 | -2.993445737 | 0.001047956 | ADAMTS19-AS1 | antisense |
| ENSG00000280420 | 1.086073044 | -2.483269341 | 5.71E-06 | AC005355.2 | TEC |
| ENSG00000145832 | 1.348896694 | -2.158574161 | 0.000104689 | SLC25A48 | protein_coding |
| ENSG00000069018 | 0.562884279 | 2.151078809 | 0.111019608 | TRPC7 | protein_coding |
| ENSG00000199990 | 2.102955873 | -2.729284117 | 5.10E-06 | VTRNA1-1 | misc_RNA |
| ENSG00000204969 | 2.942094858 | 2.204898105 | 0.000745973 | PCDHA2 | protein_coding |
| ENSG00000251664 | 2.326234809 | 2.276555873 | 0.001104869 | PCDHA12 | protein_coding |
| ENSG00000279047 | 0.75569716 | 2.622308194 | 0.030444358 | AC244517.5 | lncRNA |
| ENSG00000171815 | 3.669860745 | 2.317099878 | 0.000578228 | PCDHB1 | protein_coding |
| ENSG00000225147 | 0.570354636 | 2.171624529 | 0.03464129 | RPS12P10 | pseudogene |
| ENSG00000236714 | 104.786354 | -2.566290711 | 1.48E-16 | AC005592.1 | lincRNA |
| ENSG00000145863 | 1.643207479 | 3.720298435 | 0.045246906 | GABRA6 | protein_coding |
| ENSG00000164438 | 0.711337894 | 2.171175373 | 0.026906429 | TLX3 | protein_coding |
| ENSG00000252169 | 1.436305161 | -2.168920536 | 0.000386018 | RNA5SP200 | rRNA |
| ENSG00000184845 | 1.481511709 | 2.538681206 | 0.002575727 | DRD1 | protein_coding |
| ENSG00000164379 | 3.182256861 | 2.134420455 | 0.005318148 | FOXQ1 | protein_coding |
| ENSG00000260604 | 4211.405024 | 2.037944095 | 1.44E-13 | RP1-140K8.5 | lincRNA |
| ENSG00000205444 | 0.949465802 | 2.555994537 | 0.003726794 | RP4-529N6.1 | lincRNA |
| ENSG00000124491 | 1286.559263 | -2.295945889 | 1.12E-38 | F13A1 | protein_coding |
| ENSG00000223342 | 0.443485142 | -2.028172332 | 0.260247216 | RP3-429O6.1 | lincRNA |
| ENSG00000181355 | 0.567444749 | 2.202417703 | 0.023586437 | OFCC1 | protein_coding |
| ENSG00000230873 | 0.582725795 | 2.225277454 | 0.12898847 | STMND1 | protein_coding |
| ENSG00000219404 | 1.260723453 | 2.292804558 | 0.007176072 | RP11-524C21.1 | pseudogene |
| ENSG00000212579 | 8.562702026 | -2.228726326 | 3.80E-10 | SNORA40 | snoRNA |
| ENSG00000265527 | 16.09305508 | -2.021180213 | 7.64E-09 | MIR5690 | miRNA |
| ENSG00000180316 | 1.70918196 | 2.40218793 | 0.006498225 | PNPLA1 | protein_coding |
| ENSG00000124772 | 517.8418147 | -3.138848985 | 2.22E-15 | CPNE5 | protein_coding |
| ENSG00000285888 | 22.87722412 | -3.096671082 | 3.86E-14 | Z85996.2 | lncRNA |
| ENSG00000112077 | 6.174495134 | -2.087108292 | 4.59E-12 | RHAG | protein_coding |
| ENSG00000096006 | 0.823751234 | 2.362001544 | 0.015949358 | CRISP3 | protein_coding |
| ENSG00000168143 | 18.69335992 | -2.303437594 | 1.25E-05 | FAM83B | protein_coding |
| ENSG00000239626 | 0.822656443 | 2.016748699 | 0.040313625 | RPSAP41 | pseudogene |
| ENSG00000272243 | 3.8399953 | 2.415287794 | 8.62E-06 | RP11-554D15.3 | lincRNA |
| ENSG00000219736 | 1.501905062 | 2.195810485 | 0.004734793 | RP11-560O20.1 | pseudogene |
| ENSG00000135312 | 3.753337149 | 3.590602069 | 1.84E-05 | HTR1B | protein_coding |
| ENSG00000217334 | 7.346512836 | 2.481014583 | 2.05E-05 | RP11-33E24.3 | pseudogene |
| ENSG00000198738 | 2.324445166 | 2.887440585 | 0.014838462 | SMIM11P1 | pseudogene |
| ENSG00000222686 | 2.268629217 | 3.170757589 | 0.000986001 | RNU4-72P | snRNA |
| ENSG00000172461 | 1.663725056 | 2.704855731 | 0.001644454 | FUT9 | protein_coding |
| ENSG00000219088 | 0.773115833 | 2.632638679 | 0.003748899 | RP3-359N14.1 | pseudogene |
| ENSG00000112333 | 0.626820726 | 2.368729067 | 0.014285238 | NR2E1 | protein_coding |
| ENSG00000230943 | 8.644138992 | 2.737453909 | 1.88E-06 | RP11-367G18.1 | lincRNA |
| ENSG00000170162 | 81.61195789 | 2.037625022 | 0.004890051 | VGLL2 | protein_coding |
| ENSG00000219784 | 4.550051992 | 2.768214343 | 0.000581925 | RP1-172I22.1 | pseudogene |
| ENSG00000220326 | 0.815166819 | 2.341223659 | 0.042565635 | RP11-129H15.1 | pseudogene |
| ENSG00000164434 | 4.038889139 | 2.634621517 | 0.009554126 | FABP7 | protein_coding |
| ENSG00000146352 | 2.205275694 | 2.175293065 | 0.016924616 | CLVS2 | protein_coding |
| ENSG00000164485 | 4.501455692 | 2.138653373 | 0.000116979 | IL22RA2 | protein_coding |
| ENSG00000231178 | 0.869755302 | 2.427985908 | 0.010322939 | RP11-125D12.1 | lincRNA |
| ENSG00000234777 | 1.40363793 | 3.482355245 | 3.76E-05 | RP11-125D12.2 | lincRNA |
| ENSG00000112494 | 1.4358951 | 3.523436194 | 0.00626944 | UNC93A | protein_coding |
| ENSG00000224417 | 0.542168212 | -2.033885968 | 0.038871874 | RP11-503C24.6 | sense_intronic |
| ENSG00000217455 | 0.893708408 | 2.465876654 | 0.009259594 | AC091801.1 | protein_coding |
| ENSG00000272595 | 0.590485241 | 2.256413465 | 0.045844922 | OR10AH1P | pseudogene |
| ENSG00000239696 | 3.158741524 | 2.493470822 | 0.00104359 | RP11-397J20.1 | pseudogene |
| ENSG00000105997 | 6.041421651 | -2.288377112 | 0.000142825 | HOXA3 | protein_coding |
| ENSG00000197576 | 1.436592327 | -2.199431959 | 0.004655128 | HOXA4 | protein_coding |
| ENSG00000223437 | 0.926499807 | 2.173182764 | 0.008811534 | TMSB4XP3 | pseudogene |
| ENSG00000197085 | 3.51264395 | 2.006219473 | 0.000136254 | NPSR1-AS1 | processed_transcript |
| ENSG00000187258 | 1.438632761 | 2.497370687 | 0.002074306 | NPSR1 | protein_coding |
| ENSG00000226102 | 7.402182656 | 2.347789485 | 0.001848568 | SEPT7P3 | pseudogene |
| ENSG00000231638 | 3.953032999 | 2.501486992 | 9.47E-05 | AC011738.4 | antisense |
| ENSG00000275295 | 0.636115686 | -2.324354027 | 0.002577762 | #N/A | #N/A |
| ENSG00000232944 | 1.297393428 | 3.029286336 | 0.005333675 | RP13-492C18.1 | pseudogene |
| ENSG00000237268 | 7.343008513 | 2.215819872 | 0.004508999 | RP13-492C18.2 | pseudogene |
| ENSG00000227182 | 0.360266561 | -2.097280537 | 0.470450453 | VN1R28P | pseudogene |
| ENSG00000235349 | 13.63741755 | 3.585472365 | 1.30E-12 | RP11-561N12.6 | pseudogene |
| ENSG00000213642 | 19.24193242 | 3.376285752 | 2.92E-16 | RP11-561N12.5 | pseudogene |
| ENSG00000276475 | 0.625987609 | 2.33862099 | 0.017675576 | AC016769.4 | pseudogene |
| ENSG00000106178 | 1.485161108 | -3.394650139 | 1.66E-07 | CCL24 | protein_coding |
| ENSG00000146707 | 0.822814106 | 2.349380179 | 0.336833017 | POMZP3 | protein_coding |
| ENSG00000166448 | 3.785475946 | 2.746544241 | 1.71E-05 | TMEM130 | protein_coding |
| ENSG00000234826 | 0.542721371 | -2.610325532 | 0.033728514 | AC003084.2 | lincRNA |
| ENSG00000106304 | 7.467415105 | 2.505638233 | 8.01E-07 | SPAM1 | protein_coding |
| ENSG00000241345 | 1.798177597 | 2.488807336 | 0.001760591 | RP4-630C24.3 | antisense |
| ENSG00000128510 | 6.657707458 | 2.578229629 | 0.001796403 | CPA4 | protein_coding |
| ENSG00000221858 | 1.093081548 | 2.073825985 | 0.034164035 | OR2A12 | protein_coding |
| ENSG00000201098 | 0.525858941 | -2.392504229 | 0.005919688 | RNY1 | misc_RNA |
| ENSG00000241134 | 0.741941011 | 2.209068016 | 0.02812863 | RP5-1051J4.4 | pseudogene |
| ENSG00000243836 | 3.921738621 | -2.333211094 | 0.004027127 | WDR86-AS1 | processed_transcript |
| ENSG00000223872 | 0.956371146 | 2.22547777 | 0.009567517 | AC006372.5 | lincRNA |
| ENSG00000282306 | 0.62977668 | -2.58140806 | 0.07285763 | #N/A | #N/A |
| ENSG00000281955 | 1.283221214 | -3.00103637 | 0.061410372 | #N/A | #N/A |
| ENSG00000282300 | 0.556161176 | -2.780495657 | 0.074461721 | #N/A | #N/A |
| ENSG00000198759 | 2.099428162 | 3.06683037 | 0.000321901 | EGFL6 | protein_coding |
| ENSG00000165164 | 2.320119449 | 2.538787703 | 0.00083983 | CXorf22 | protein_coding |
| ENSG00000223723 | 0.923507701 | 2.899892598 | 0.000670247 | BX842568.2 | pseudogene |
| ENSG00000259977 | 9.138752728 | 2.580678691 | 7.87E-07 | AL121578.2 | lincRNA |
| ENSG00000224339 | 2.663282996 | 3.430317797 | 7.13E-06 | AL121578.5 | pseudogene |
| ENSG00000187516 | 2.031658554 | 3.342014788 | 1.88E-05 | CXorf27 | protein_coding |
| ENSG00000147041 | 180.629393 | 2.488356194 | 2.04E-09 | SYTL5 | protein_coding |
| ENSG00000236393 | 4.482847596 | 2.353867968 | 7.89E-06 | RP11-320G24.1 | lincRNA |
| ENSG00000277721 | 0.511783047 | -2.104958982 | 0.015307122 | #N/A | #N/A |
| ENSG00000220125 | 0.319561707 | -2.023623461 | 0.116680223 | MRPL32P1 | pseudogene |
| ENSG00000102109 | 5.072390211 | 2.900813279 | 6.52E-06 | PCSK1N | protein_coding |
| ENSG00000236852 | 0.805890884 | 2.318719512 | 0.009850483 | RP11-3D23.1 | pseudogene |
| ENSG00000155659 | 242.4875957 | -2.124940265 | 2.18E-26 | VSIG4 | protein_coding |
| ENSG00000230146 | 6.039597139 | 2.084924836 | 0.202606297 | SEPHS1P4 | pseudogene |
| ENSG00000223592 | 1.825988628 | 2.201595733 | 0.002581465 | FNDC3CP | pseudogene |
| ENSG00000101890 | 1.026063638 | 2.006130602 | 0.022942511 | GUCY2F | protein_coding |
| ENSG00000260802 | 3.209190957 | 2.782866779 | 0.000164404 | LINC00890 | lincRNA |
| ENSG00000268104 | 0.90951693 | 2.858184345 | 0.017132574 | SLC6A14 | protein_coding |
| ENSG00000101892 | 185.073988 | 2.493980376 | 4.70E-08 | ATP1B4 | protein_coding |
| ENSG00000218728 | 0.419141317 | -2.072131585 | 0.066170426 | KRT18P44 | pseudogene |
| ENSG00000216001 | 0.394763595 | -2.346383906 | 0.012696061 | MIR450B | miRNA |
| ENSG00000179542 | 672.2598192 | 3.644176284 | 1.91E-19 | SLITRK4 | protein_coding |
| ENSG00000147381 | 1.513308444 | 3.604706079 | 0.294162583 | MAGEA4 | protein_coding |
| ENSG00000268089 | 3.971139763 | 3.393318304 | 0.000100867 | AC244102.1 | protein_coding |
| ENSG00000164825 | 4.270111382 | 2.320829567 | 0.008802912 | DEFB1 | protein_coding |
| ENSG00000284717 | 0.398485732 | -2.022145051 | 0.053757396 | #N/A | #N/A |
| ENSG00000171060 | 4.300413123 | 2.594240006 | 2.94E-05 | C8orf74 | protein_coding |
| ENSG00000184608 | 8.914604736 | -2.137278643 | 5.49E-12 | C8orf12 | protein_coding |
| ENSG00000134028 | 2.351280706 | -3.966716784 | 1.82E-06 | ADAMDEC1 | protein_coding |
| ENSG00000069206 | 1.05085789 | 2.023374063 | 0.025917148 | ADAM7 | protein_coding |
| ENSG00000184661 | 174.0371307 | -2.190098323 | 1.22E-14 | CDCA2 | protein_coding |
| ENSG00000253899 | 1.146964423 | 2.482994983 | 0.006083416 | RP11-613H2.2 | pseudogene |
| ENSG00000251191 | 1.118138124 | 2.146011116 | 0.012678111 | LINC00589 | lincRNA |
| ENSG00000168619 | 1.041094414 | 3.066117942 | 0.003729332 | ADAM18 | protein_coding |
| ENSG00000253838 | 0.96004401 | -2.037189501 | 0.008351851 | RP11-44K6.2 | sense_intronic |
| ENSG00000260484 | 39.82102237 | -3.358321706 | 2.92E-17 | RP11-1081M5.2 | lincRNA |
| ENSG00000274295 | 49.77948603 | -3.319171754 | 1.30E-16 | #N/A | #N/A |
| ENSG00000253369 | 55.46519733 | -3.485963341 | 5.07E-33 | RP11-1081M5.1 | lincRNA |
| ENSG00000240919 | 0.199468843 | -2.11852069 | 0.399414688 | RP11-400K9.1 | pseudogene |
| ENSG00000170786 | 7.827488066 | 2.824462308 | 0.000554202 | SDR16C5 | protein_coding |
| ENSG00000181195 | 81.635562 | 2.670106606 | 5.91E-09 | PENK | protein_coding |
| ENSG00000253894 | 0.647606763 | 2.391105663 | 0.016480643 | RP11-45K10.2 | lincRNA |
| ENSG00000260838 | 0.541871827 | 2.166210115 | 0.033226397 | RP11-531A24.3 | lincRNA |
| ENSG00000276673 | 0.824931141 | -2.117981476 | 0.001886088 | #N/A | #N/A |
| ENSG00000164879 | 606.8171726 | 2.876428283 | 5.99E-08 | CA3 | protein_coding |
| ENSG00000253699 | 0.521083021 | 2.101202235 | 0.046215732 | CTD-3118D11.3 | antisense |
| ENSG00000251563 | 1.744439362 | 2.431338613 | 0.002167674 | AF121898.1 | pseudogene |
| ENSG00000253901 | 0.98110007 | 2.247922765 | 0.015527335 | RP11-122C21.1 | lincRNA |
| ENSG00000253960 | 0.538178441 | -2.593568956 | 0.002758554 | RP11-388K12.1 | lincRNA |
| ENSG00000176406 | 3.346987475 | 2.832956912 | 0.000199649 | RIMS2 | protein_coding |
| ENSG00000205038 | 231.0460216 | -2.125139116 | 1.01E-06 | PKHD1L1 | protein_coding |
| ENSG00000254185 | 1.560443745 | -2.613670511 | 0.005965404 | RP11-419L20.2 | pseudogene |
| ENSG00000253972 | 1.115167032 | 2.794683142 | 0.000400852 | RP11-4K16.2 | lincRNA |
| ENSG00000253495 | 1.429639218 | 3.174901253 | 0.000450951 | RP11-99I9.2 | pseudogene |
| ENSG00000170961 | 59.04341216 | -2.688147911 | 8.24E-24 | HAS2 | protein_coding |
| ENSG00000254303 | 0.609147749 | -2.686858193 | 0.002216696 | RP11-398G24.2 | lincRNA |
| ENSG00000255364 | 4.464422281 | -2.150330644 | 0.000310816 | RP11-94A24.1 | lincRNA |
| ENSG00000214814 | 19.13086127 | -2.1537545 | 1.88E-10 | FER1L6 | protein_coding |
| ENSG00000249816 | 16.43847732 | -2.182807193 | 2.45E-08 | LINC00964 | lincRNA |
| ENSG00000253220 | 0.641142356 | -2.888713961 | 0.000349757 | RP11-103H7.2 | lincRNA |
| ENSG00000132297 | 0.963029306 | 2.611442556 | 0.007262106 | HHLA1 | protein_coding |
| ENSG00000169436 | 31.5356039 | 2.36450827 | 7.95E-07 | COL22A1 | protein_coding |
| ENSG00000226807 | 2.154632108 | 2.44619954 | 0.001522028 | MROH5 | polymorphic_pseudogene |
| ENSG00000126233 | 11.79428085 | 3.320708469 | 6.88E-05 | SLURP1 | protein_coding |
| ENSG00000167656 | 9.449167951 | 5.596917458 | 1.11E-05 | LY6D | protein_coding |
| ENSG00000147813 | 12.97180357 | 2.471629114 | 6.82E-05 | NAPRT1 | protein_coding |
| ENSG00000137090 | 0.518427791 | 2.098744092 | 0.045106392 | DMRT1 | protein_coding |
| ENSG00000064218 | 0.837846897 | 2.418148586 | 0.051403238 | DMRT3 | protein_coding |
| ENSG00000264615 | 0.723699647 | 2.215593117 | 0.018368604 | RN7SL592P | misc_RNA |
| ENSG00000270547 | 3.047382231 | 2.073498913 | 0.000816256 | RP11-536O18.2 | lincRNA |
| ENSG00000260412 | 1.103535428 | -2.053545012 | 0.008072523 | RP11-438B23.2 | sense_overlapping |
| ENSG00000122728 | 1.528207479 | 2.216826878 | 0.020879329 | TAF1L | protein_coding |
| ENSG00000230453 | 27.13648864 | 2.160164234 | 3.02E-09 | ANKRD18B | protein_coding |
| ENSG00000234373 | 3.984840757 | 2.879168813 | 3.29E-05 | SNX18P7 | pseudogene |
| ENSG00000231165 | 4.800316242 | 2.113233371 | 6.19E-06 | TRBV26OR9-2 | IG_V_pseudogene |
| ENSG00000010438 | 2.554445423 | 2.159259506 | 0.001726469 | PRSS3 | protein_coding |
| ENSG00000196092 | 2.328433444 | 3.550385467 | 0.000198366 | PAX5 | protein_coding |
| ENSG00000234680 | 6.45459012 | -2.314272391 | 0.000233512 | VN2R3P | pseudogene |
| ENSG00000283886 | 1.543361827 | 2.25613358 | 0.021888695 | #N/A | #N/A |
| ENSG00000277618 | 0.966800679 | 2.250023867 | 0.039610446 | #N/A | #N/A |
| ENSG00000204711 | 1.844495174 | -2.123852759 | 0.000316996 | C9orf135 | protein_coding |
| ENSG00000228248 | 2.987773307 | 2.018229112 | 0.000538274 | RP11-111E2.1 | pseudogene |
| ENSG00000285987 | 1.073211375 | 2.411594366 | 0.005253335 | #N/A | #N/A |
| ENSG00000188029 | 0.75523942 | -2.702877534 | 0.001170426 | CTSL3P | pseudogene |
| ENSG00000225385 | 0.488193254 | -2.618887987 | 0.01847453 | RP11-350E12.4 | pseudogene |
| ENSG00000237372 | 0.452272325 | -2.029226362 | 0.035662706 | RP11-316P17.2 | lincRNA |
| ENSG00000226206 | 1.102954005 | 2.121165404 | 0.010056516 | RP5-1050E16.2 | lincRNA |
| ENSG00000136943 | 10.32761931 | 2.291902028 | 0.000946846 | CTSV | protein_coding |
| ENSG00000078725 | 42.99304441 | 2.515619608 | 7.21E-14 | BRINP1 | protein_coding |
| ENSG00000173679 | 0.673737647 | 2.046183143 | 0.036318814 | OR1L1 | protein_coding |
| ENSG00000160339 | 0.522718668 | -2.553210181 | 0.006061469 | FCN2 | protein_coding |
| ENSG00000236403 | 0.303832436 | -2.469485165 | 0.114683604 | RP11-447M12.2 | lincRNA |
| ENSG00000160349 | 4.592579305 | 4.879567498 | 5.68E-07 | LCN1 | protein_coding |
| ENSG00000122136 | 1.389409739 | 3.136221131 | 0.00493238 | OBP2A | protein_coding |
| ENSG00000148408 | 36.30309582 | 2.089645316 | 3.12E-07 | CACNA1B | protein_coding |
| ENSG00000149043 | 2.602787063 | 4.044909737 | 0.00274343 | SYT8 | protein_coding |
| ENSG00000244734 | 300.8076923 | 2.712695598 | 4.59E-08 | HBB | protein_coding |
| ENSG00000181017 | 1.482522939 | -2.199537426 | 0.001014208 | OR56B2P | pseudogene |
| ENSG00000133800 | 343.4652978 | -2.028299788 | 9.92E-28 | LYVE1 | protein_coding |
| ENSG00000255260 | 0.603959283 | 2.279746645 | 0.120299565 | CTD-3224I3.3 | lincRNA |
| ENSG00000254645 | 0.813645014 | 2.008930214 | 0.031770077 | RP11-396O20.2 | lincRNA |
| ENSG00000166796 | 2.0791908 | 3.052985682 | 0.006476245 | LDHC | protein_coding |
| ENSG00000169550 | 3.966110053 | 2.974995433 | 0.011919152 | MUC15 | protein_coding |
| ENSG00000135374 | 0.759899731 | 2.224185674 | 0.021963348 | ELF5 | protein_coding |
| ENSG00000254654 | 1.447412769 | 2.854825321 | 0.001749892 | AC068858.1 | lincRNA |
| ENSG00000254519 | 1.210759039 | -4.01117539 | 4.67E-08 | CTD-2210P24.1 | lincRNA |
| ENSG00000255042 | 2.595053052 | 2.732843853 | 0.003578612 | RP11-347H15.5 | pseudogene |
| ENSG00000110484 | 7.52597505 | 5.916766524 | 0.000286197 | SCGB2A2 | protein_coding |
| ENSG00000172818 | 1.602202279 | 2.982691403 | 0.005210844 | OVOL1 | protein_coding |
| ENSG00000175315 | 16.65774258 | 2.583752316 | 0.006644892 | CST6 | protein_coding |
| ENSG00000174576 | 1.184347161 | 2.209189383 | 0.014583893 | NPAS4 | protein_coding |
| ENSG00000173227 | 17.37376958 | 2.860152231 | 1.52E-13 | SYT12 | protein_coding |
| ENSG00000132746 | 8.829347264 | 6.146226771 | 1.59E-07 | ALDH3B2 | protein_coding |
| ENSG00000069482 | 1.464417603 | 3.213441582 | 0.001057197 | GAL | protein_coding |
| ENSG00000254605 | 15.69054751 | 2.230479789 | 7.50E-14 | RP11-626H12.2 | lincRNA |
| ENSG00000226627 | 1.5227213 | 2.270326055 | 0.005993052 | SHANK2-AS1 | antisense |
| ENSG00000204971 | 0.873611831 | -3.497897868 | 0.000181517 | RP11-807H22.7 | antisense |
| ENSG00000054938 | 36.03800479 | -3.620534418 | 2.89E-10 | CHRDL2 | protein_coding |
| ENSG00000279353 | 1.966426042 | -2.776430793 | 6.21E-08 | #N/A | #N/A |
| ENSG00000279295 | 1.137007622 | 2.474718999 | 0.002209491 | #N/A | #N/A |
| ENSG00000255063 | 1.310366158 | 3.020571416 | 0.000327797 | RP11-718B12.1 | pseudogene |
| ENSG00000165323 | 190.9455434 | 2.833791103 | 2.94E-09 | FAT3 | protein_coding |
| ENSG00000249255 | 1.04702251 | 2.743109371 | 0.012614386 | PGAM1P9 | pseudogene |
| ENSG00000254705 | 2.907585297 | 2.981064284 | 0.00097815 | RP11-675M1.2 | antisense |
| ENSG00000256779 | 1.170200916 | 2.179118939 | 0.006278167 | RP11-60C6.5 | pseudogene |
| ENSG00000149968 | 2.958843196 | 4.237521218 | 0.002221508 | MMP3 | protein_coding |
| ENSG00000254750 | 8.234778136 | -2.062272095 | 5.29E-05 | CASP1P2 | pseudogene |
| ENSG00000255221 | 2.195538812 | -2.114560092 | 0.000154062 | CARD17 | protein_coding |
| ENSG00000185742 | 3.209469595 | -2.451318464 | 1.07E-07 | C11orf87 | protein_coding |
| ENSG00000154096 | 142.3323589 | 2.708503206 | 2.57E-13 | THY1 | protein_coding |
| ENSG00000137699 | 66.99530683 | 3.844041591 | 2.91E-05 | TRIM29 | protein_coding |
| ENSG00000137709 | 7.48099954 | 2.162093314 | 5.97E-05 | POU2F3 | protein_coding |
| ENSG00000286023 | 0.76441396 | 2.621329009 | 0.006001975 | #N/A | #N/A |
| ENSG00000181518 | 1.095832123 | -2.504194182 | 0.004458315 | OR8D4 | protein_coding |
| ENSG00000198674 | 0.210355191 | -2.199173009 | 0.376995537 | OR10G6 | protein_coding |
| ENSG00000272575 | 0.461182059 | -2.654084467 | 0.075453567 | RP11-702B10.1 | lincRNA |
| ENSG00000043039 | 1.785599018 | 3.494973006 | 9.85E-05 | BARX2 | protein_coding |
| ENSG00000254896 | 0.402222322 | -2.299980188 | 0.040286747 | OPCML-IT1 | sense_intronic |
| ENSG00000251226 | 1.205906372 | -2.264752806 | 0.004707392 | RP11-469N6.1 | lincRNA |
| ENSG00000285735 | 0.549599083 | 2.143776743 | 0.067569403 | #N/A | #N/A |
| ENSG00000229205 | 4.91620299 | 4.323513998 | 0.000261063 | LINC00200 | lincRNA |
| ENSG00000282390 | 2.581269463 | 2.723000548 | 0.00514249 | #N/A | #N/A |
| ENSG00000233117 | 2535.162197 | 2.284345468 | 8.18E-37 | LINC00702 | lincRNA |
| ENSG00000178372 | 64.2360423 | 9.011007618 | 1.03E-06 | CALML5 | protein_coding |
| ENSG00000232591 | 0.552682889 | -2.199079997 | 0.012363661 | RP5-1031D4.2 | lincRNA |
| ENSG00000225383 | 8.947486137 | 2.479343727 | 1.64E-06 | SFTA1P | lincRNA |
| ENSG00000280086 | 0.696731546 | 2.105192345 | 0.063376871 | #N/A | #N/A |
| ENSG00000235410 | 0.807485226 | -2.191464291 | 0.008191567 | RP11-397C18.2 | antisense |
| ENSG00000095777 | 9.085997797 | 2.533822545 | 7.74E-09 | MYO3A | protein_coding |
| ENSG00000227313 | 0.820686283 | -3.417369153 | 0.000143117 | RP11-309N24.1 | lincRNA |
| ENSG00000148513 | 0.567501033 | 2.200164417 | 0.043438338 | ANKRD30A | protein_coding |
| ENSG00000263639 | 0.669029211 | 2.397968846 | 0.037446412 | MSMB | protein_coding |
| ENSG00000264230 | 5.754049014 | 4.550244403 | 0.001267248 | AC244230.1 | protein_coding |
| ENSG00000204175 | 1.464671395 | 2.850027239 | 0.009834152 | GPRIN2 | protein_coding |
| ENSG00000265190 | 8.67691796 | 4.050417321 | 9.27E-05 | ANXA8 | protein_coding |
| ENSG00000276430 | 0.662912089 | 2.028068161 | 0.022550832 | #N/A | #N/A |
| ENSG00000234173 | 3.622586537 | 2.25246328 | 0.000809449 | RP11-257I14.1 | antisense |
| ENSG00000224697 | 1.514218756 | 2.208982401 | 0.014553287 | NEFMP1 | pseudogene |
| ENSG00000236744 | 0.654172865 | -2.383401573 | 0.042427779 | RP11-168O22.1 | sense_intronic |
| ENSG00000228048 | 0.799899121 | -2.497523425 | 0.002567093 | RP11-598C10.1 | lincRNA |
| ENSG00000223841 | 0.433113819 | -2.167433602 | 0.132893445 | RP11-448K10.1 | pseudogene |
| ENSG00000226426 | 1.578884996 | 2.63393507 | 0.002109195 | RP11-174J11.1 | lincRNA |
| ENSG00000233197 | 1.490898271 | 2.249542349 | 0.013532533 | TMEM256P1 | pseudogene |
| ENSG00000122859 | 0.689456546 | -2.128009471 | 0.006046606 | NEUROG3 | protein_coding |
| ENSG00000226794 | 0.324842672 | -2.341950441 | 0.088741954 | MTND1P20 | pseudogene |
| ENSG00000272536 | 2.216889382 | 2.810533303 | 0.000436401 | Metazoa_SRP | misc_RNA |
| ENSG00000188373 | 1.091861225 | 3.120758109 | 0.014012062 | C10orf99 | protein_coding |
| ENSG00000237740 | 0.9803453 | 2.625374926 | 0.00120802 | NPAP1P3 | pseudogene |
| ENSG00000204021 | 1.27223439 | 2.985980486 | 0.008027627 | LIPK | protein_coding |
| ENSG00000173239 | 1.336873859 | 3.405278187 | 0.005020777 | LIPM | protein_coding |
| ENSG00000224851 | 3.641277752 | 2.99027152 | 2.64E-05 | LINC00502 | lincRNA |
| ENSG00000220585 | 3.082812505 | 2.508177325 | 0.000160601 | DDX18P6 | pseudogene |
| ENSG00000138207 | 9.192625332 | -2.454490375 | 0.000145249 | RBP4 | protein_coding |
| ENSG00000280660 | 4.646827366 | 2.068730318 | 3.62E-05 | #N/A | #N/A |
| ENSG00000206631 | 3.090258529 | 2.091366871 | 0.000120619 | RNU6-657P | snRNA |
| ENSG00000165841 | 0.664380785 | 2.005191041 | 0.050770668 | CYP2C19 | protein_coding |
| ENSG00000260863 | 0.31449139 | -2.107894108 | 0.245079429 | CYP2C60P | pseudogene |
| ENSG00000227128 | 0.537420503 | 2.155401369 | 0.04427438 | LBX1-AS1 | antisense |
| ENSG00000229466 | 0.271619144 | -2.326117985 | 0.201482024 | RP11-56I23.1 | lincRNA |
| ENSG00000151892 | 160.7578827 | -2.235124745 | 1.55E-20 | GFRA1 | protein_coding |
| ENSG00000203837 | 2.259270252 | 4.179639541 | 0.000413608 | PNLIPRP3 | protein_coding |
| ENSG00000225152 | 1.165064144 | 2.511498999 | 0.002482366 | RP11-338O1.2 | lincRNA |
| ENSG00000266043 | 0.695428589 | -2.149496907 | 0.011851369 | MIR3649 | miRNA |
| ENSG00000256654 | 0.970770283 | 2.232713814 | 0.01243855 | RP11-429A20.4 | lincRNA |
| ENSG00000177575 | 855.6577718 | -2.769027957 | 4.77E-33 | CD163 | protein_coding |
| ENSG00000280345 | 0.641873552 | -2.943075092 | 0.002830603 | #N/A | #N/A |
| ENSG00000008394 | 72.70951819 | -2.88647853 | 3.72E-28 | MGST1 | protein_coding |
| ENSG00000255871 | 0.428443625 | -2.382517452 | 0.01440009 | RP11-69C13.1 | lincRNA |
| ENSG00000257037 | 1.130743413 | 2.490704906 | 0.004160548 | RP13-200J3.2 | pseudogene |
| ENSG00000257849 | 1.79721958 | -2.120511288 | 0.000109373 | RP11-547C5.1 | lincRNA |
| ENSG00000257373 | 0.965395952 | -2.695222816 | 0.003303289 | RP11-547C5.2 | lincRNA |
| ENSG00000257927 | 0.640292531 | -2.600808518 | 0.028311084 | MRPS36P5 | pseudogene |
| ENSG00000277672 | 2.089311312 | 2.133422947 | 0.006248051 | #N/A | #N/A |
| ENSG00000135472 | 45.4181753 | -2.804440923 | 1.15E-17 | FAIM2 | protein_coding |
| ENSG00000170523 | 0.837219678 | 2.746668353 | 0.323586501 | KRT83 | protein_coding |
| ENSG00000274928 | 0.273182774 | -2.252638279 | 0.206940087 | #N/A | #N/A |
| ENSG00000135443 | 1.996831069 | 4.002812817 | 0.025865834 | KRT85 | protein_coding |
| ENSG00000170454 | 0.82084008 | 2.707248096 | 0.020450052 | KRT75 | protein_coding |
| ENSG00000185479 | 13.07317554 | 3.126512177 | 0.006002687 | KRT6B | protein_coding |
| ENSG00000170465 | 4.38248301 | 4.153173702 | 0.032291923 | KRT6C | protein_coding |
| ENSG00000205420 | 29.46226619 | 6.324222757 | 0.000118586 | KRT6A | protein_coding |
| ENSG00000186081 | 449.0588515 | 7.933565524 | 1.96E-10 | KRT5 | protein_coding |
| ENSG00000139648 | 1.889636179 | 3.23699572 | 0.067700707 | KRT71 | protein_coding |
| ENSG00000186049 | 0.589329412 | 2.191699329 | 0.032286954 | KRT73 | protein_coding |
| ENSG00000172867 | 346.4625715 | 12.27683366 | 4.81E-10 | KRT2 | protein_coding |
| ENSG00000167768 | 1229.193727 | 25.39126025 | 3.84E-35 | KRT1 | protein_coding |
| ENSG00000189182 | 18.85445945 | 6.919643552 | 1.48E-05 | KRT77 | protein_coding |
| ENSG00000185640 | 2.12261278 | 4.087177624 | 0.019040528 | KRT79 | protein_coding |
| ENSG00000170423 | 6.660763289 | 5.739426935 | 9.36E-05 | KRT78 | protein_coding |
| ENSG00000161634 | 37.41881047 | 8.231358771 | 3.77E-06 | DCD | protein_coding |
| ENSG00000172551 | 7.271492083 | 5.540563862 | 2.10E-05 | MUCL1 | protein_coding |
| ENSG00000185821 | 0.506994201 | -2.715233076 | 0.000192428 | OR6C76 | protein_coding |
| ENSG00000258763 | 5.313202111 | -2.333216776 | 8.21E-11 | RP11-110A12.2 | antisense |
| ENSG00000170439 | 60.03154126 | -2.175546993 | 6.86E-20 | METTL7B | protein_coding |
| ENSG00000182379 | 83.55838754 | 2.003325572 | 1.24E-16 | NXPH4 | protein_coding |
| ENSG00000255693 | 1.13664244 | 2.154760516 | 0.018278793 | RP11-766N7.3 | lincRNA |
| ENSG00000241749 | 1.921615831 | 2.271130169 | 0.003264914 | RPSAP52 | pseudogene |
| ENSG00000256355 | 1.75270089 | 2.462218084 | 0.001191814 | NTAN1P3 | pseudogene |
| ENSG00000256172 | 1.151996525 | 2.52007761 | 0.004965587 | RP11-473M14.3 | lincRNA |
| ENSG00000203585 | 8.23104412 | 2.205279614 | 4.22E-08 | RP11-542B15.1 | lincRNA |
| ENSG00000256708 | 1.360356045 | -2.467393815 | 0.000117384 | RP11-444B24.2 | pseudogene |
| ENSG00000258077 | 1.055709431 | 2.057883129 | 0.011289372 | RP11-114H23.1 | lincRNA |
| ENSG00000286259 | 0.648469555 | 2.426229026 | 0.008567324 | #N/A | #N/A |
| ENSG00000257429 | 0.554715705 | 2.18333773 | 0.036539679 | RP11-272K23.3 | antisense |
| ENSG00000231738 | 2.950616327 | -2.434810364 | 2.84E-05 | TSPAN19 | protein_coding |
| ENSG00000286021 | 25.31507187 | -3.848319902 | 3.60E-06 | #N/A | #N/A |
| ENSG00000257995 | 5.867620788 | -4.726811926 | 4.33E-08 | RP11-632B21.1 | lincRNA |
| ENSG00000139330 | 1.197273304 | 2.908095644 | 0.002684757 | KERA | protein_coding |
| ENSG00000075035 | 4.265069782 | -2.027862374 | 2.67E-05 | WSCD2 | protein_coding |
| ENSG00000111249 | 30.21885442 | 2.317614305 | 0.000111951 | CUX2 | protein_coding |
| ENSG00000255398 | 0.813018758 | 2.71243863 | 0.007262016 | HCAR3 | protein_coding |
| ENSG00000279931 | 0.784283986 | -2.450730302 | 0.001592408 | #N/A | #N/A |
| ENSG00000139364 | 40.57354117 | -2.068589585 | 3.05E-08 | TMEM132B | protein_coding |
| ENSG00000230294 | 1.020829024 | 2.364962262 | 0.004541462 | RP13-507P19.2 | lincRNA |
| ENSG00000165474 | 10.83740298 | 2.300396488 | 6.62E-05 | GJB2 | protein_coding |
| ENSG00000121742 | 3.142325212 | 2.964966211 | 0.004678565 | GJB6 | protein_coding |
| ENSG00000075673 | 0.9907086 | 2.979184125 | 0.03359443 | ATP12A | protein_coding |
| ENSG00000180730 | 33.80918404 | 2.405585604 | 1.28E-14 | SHISA2 | protein_coding |
| ENSG00000133105 | 35.40348026 | 3.002964869 | 2.14E-31 | RXFP2 | protein_coding |
| ENSG00000180440 | 7.740504742 | -2.459774485 | 4.00E-05 | SERTM1 | protein_coding |
| ENSG00000181358 | 0.809022219 | 2.347012484 | 0.006975927 | CTAGE10P | pseudogene |
| ENSG00000263581 | 8.003104964 | -2.534838023 | 1.84E-11 | MIR548X2 | miRNA |
| ENSG00000223458 | 2.513928956 | 2.152456325 | 0.000498256 | RP11-332E3.2 | sense_intronic |
| ENSG00000136155 | 27.25748008 | 7.773835524 | 1.30E-08 | SCEL | protein_coding |
| ENSG00000233379 | 1.088339694 | 2.055118635 | 0.018745108 | RP11-318G21.4 | lincRNA |
| ENSG00000271776 | 0.724166135 | 2.199927182 | 0.037053735 | RP11-52L5.6 | antisense |
| ENSG00000282997 | 2.973612193 | -2.129471072 | 0.000616236 | #N/A | #N/A |
| ENSG00000229287 | 0.90453472 | -2.412867688 | 0.000974622 | FABP5P4 | pseudogene |
| ENSG00000232885 | 2.132504792 | -2.448783954 | 3.09E-05 | GPC5-AS2 | antisense |
| ENSG00000236240 | 2.034672735 | -2.416352276 | 0.001253334 | GPC5-IT1 | sense_intronic |
| ENSG00000235984 | 1.208134143 | -3.428447111 | 2.56E-06 | GPC5-AS1 | antisense |
| ENSG00000125285 | 2.111470446 | 3.392766048 | 0.000408707 | SOX21 | protein_coding |
| ENSG00000227640 | 1.482124719 | 2.159920076 | 0.012359924 | SOX21-AS1 | lincRNA |
| ENSG00000088386 | 2.216178661 | 3.128846431 | 0.000585796 | SLC15A1 | protein_coding |
| ENSG00000125255 | 5.624950151 | 2.286367127 | 4.24E-05 | SLC10A2 | protein_coding |
| ENSG00000272274 | 0.645336018 | 2.01805949 | 0.032753559 | LINC00551 | lincRNA |
| ENSG00000277128 | 4.640393706 | 2.243018193 | 0.000419021 | #N/A | #N/A |
| ENSG00000278594 | 1.537035129 | 2.258306102 | 0.010351379 | #N/A | #N/A |
| ENSG00000169397 | 2.003905781 | 2.036477164 | 0.004639026 | RNASE3 | protein_coding |
| ENSG00000258918 | 1.62213341 | 2.021939942 | 0.011171023 | RP11-219E7.4 | lincRNA |
| ENSG00000243817 | 0.703590375 | 2.513195421 | 0.005543308 | RN7SL189P | misc_RNA |
| ENSG00000100867 | 2.199807623 | 2.153995108 | 0.03397411 | DHRS2 | protein_coding |
| ENSG00000258098 | 9.714548886 | -2.02803916 | 0.000155299 | RP11-89K22.1 | lincRNA |
| ENSG00000257612 | 2.016528853 | 3.334955437 | 0.000242273 | RP11-384J4.1 | lincRNA |
| ENSG00000207366 | 1.686208578 | 2.449166397 | 0.007992808 | RNU6-297P | snRNA |
| ENSG00000258751 | 14.52207846 | 2.358093568 | 1.17E-06 | RP11-2G1.1 | lincRNA |
| ENSG00000258750 | 8.029961627 | 3.235513598 | 1.45E-07 | RP11-2G1.2 | pseudogene |
| ENSG00000242417 | 2.831693991 | 2.110283094 | 0.002765389 | RP11-286B5.1 | pseudogene |
| ENSG00000248550 | 3.478291902 | 3.817095123 | 2.41E-06 | OTX2-AS1 | antisense |
| ENSG00000258776 | 0.932305984 | 2.929777603 | 0.000787546 | RP11-1085N6.5 | lincRNA |
| ENSG00000286257 | 3.745085917 | 2.989959949 | 3.58E-05 | #N/A | #N/A |
| ENSG00000179008 | 1.139102226 | 2.866102716 | 0.000682011 | C14orf39 | protein_coding |
| ENSG00000258952 | 1.059656645 | 2.756909846 | 0.003500327 | RP11-1042B17.5 | lincRNA |
| ENSG00000126778 | 4.626112375 | 3.59463665 | 2.73E-06 | SIX1 | protein_coding |
| ENSG00000259076 | 1.548672596 | 3.311918606 | 9.64E-05 | RP11-973N13.3 | antisense |
| ENSG00000259010 | 1.196711076 | 2.921731199 | 0.001787062 | RP11-973N13.2 | pseudogene |
| ENSG00000266553 | 2.331552599 | 2.209242903 | 0.020327517 | RN7SL356P | misc_RNA |
| ENSG00000258723 | 4.169272496 | 3.747496515 | 1.53E-06 | RP11-332E19.2 | antisense |
| ENSG00000258719 | 0.689893235 | 2.075946926 | 0.079140101 | RP11-232C2.3 | antisense |
| ENSG00000258478 | 1.819756682 | 3.523750799 | 0.000173538 | RP11-232C2.1 | antisense |
| ENSG00000258829 | 9.039388472 | 3.8703062 | 7.03E-08 | CTD-2243E23.1 | antisense |
| ENSG00000258419 | 8.373291253 | 3.717085266 | 3.35E-07 | RP11-588P7.1 | sense_intronic |
| ENSG00000258662 | 7.252069688 | 3.941533608 | 1.76E-06 | RP11-588P7.2 | antisense |
| ENSG00000259167 | 1.901522064 | -2.580676283 | 3.18E-05 | NMNAT1P1 | pseudogene |
| ENSG00000258902 | 0.708628134 | 2.512924747 | 0.038414303 | CTD-2128A3.2 | lincRNA |
| ENSG00000233208 | 2.169985572 | 3.089125524 | 8.35E-05 | LINC00642 | lincRNA |
| ENSG00000175699 | 2.66484405 | 2.314047613 | 0.000751812 | LINC00521 | processed_transcript |
| ENSG00000165953 | 26.01225414 | 7.706598258 | 7.35E-06 | SERPINA12 | protein_coding |
| ENSG00000188488 | 13.97885513 | -2.257541793 | 2.11E-12 | SERPINA5 | protein_coding |
| ENSG00000196136 | 3.001952453 | -3.819068241 | 4.52E-09 | SERPINA3 | protein_coding |
| ENSG00000200632 | 0.829504976 | -2.416296172 | 0.00149332 | SNORD113-7 | snoRNA |
| ENSG00000201263 | 1.316030756 | -2.635021874 | 0.002768431 | SNORD114-6 | snoRNA |
| ENSG00000283588 | 0.422990887 | -2.32847936 | 0.029898393 | #N/A | #N/A |
| ENSG00000207742 | 0.960256488 | -2.078133939 | 0.001814511 | MIR382 | miRNA |
| ENSG00000259428 | 0.932236677 | -2.393254641 | 0.001805206 | HMGB3P26 | pseudogene |
| ENSG00000211935 | 1.269049452 | -2.012253426 | 0.087552935 | IGHV1-3 | IG_V_gene |
| ENSG00000282122 | 0.580297635 | -2.753370344 | 0.026657476 | #N/A | #N/A |
| ENSG00000211941 | 0.321952159 | -2.083391135 | 0.06621864 | IGHV3-11 | IG_V_gene |
| ENSG00000259156 | 0.660168484 | 2.384829089 | 0.045576395 | CHEK2P2 | pseudogene |
| ENSG00000270831 | 0.33687458 | -2.351606819 | 0.130816634 | NF1P1 | pseudogene |
| ENSG00000258710 | 1.223028971 | -3.276381676 | 0.000501554 | CT60 | lincRNA |
| ENSG00000261418 | 1.742568103 | -2.106685873 | 0.000124223 | RP11-529J17.3 | pseudogene |
| ENSG00000260232 | 0.784626012 | -2.50027834 | 0.004477649 | PWRN4 | lincRNA |
| ENSG00000200812 | 1.576250177 | 2.29462281 | 0.002597299 | SNORD115-6 | snoRNA |
| ENSG00000200757 | 0.692194294 | 2.098094664 | 0.048795639 | SNORD115-16 | snoRNA |
| ENSG00000199833 | 2.628458727 | 2.489909421 | 0.000316234 | SNORD115-21 | snoRNA |
| ENSG00000235160 | 32.23060873 | -2.781954777 | 2.77E-05 | AC009878.2 | lincRNA |
| ENSG00000198826 | 0.72835151 | 2.160480316 | 0.024659247 | ARHGAP11A | protein_coding |
| ENSG00000259390 | 2.491577937 | 2.097735843 | 0.003109872 | RP11-27M9.1 | lincRNA |
| ENSG00000104140 | 4.777786921 | 4.274169426 | 9.57E-05 | RHOV | protein_coding |
| ENSG00000188089 | 5.28653541 | 3.049316081 | 0.000242753 | PLA2G4E | protein_coding |
| ENSG00000159337 | 1.794511914 | 2.087385434 | 0.028796187 | PLA2G4D | protein_coding |
| ENSG00000259671 | 5.653161842 | 2.016056286 | 0.001798931 | RP11-925D8.6 | pseudogene |
| ENSG00000074410 | 18.24459515 | 2.285365663 | 0.000195127 | CA12 | protein_coding |
| ENSG00000140465 | 22.73042181 | -4.504802796 | 9.05E-12 | CYP1A1 | protein_coding |
| ENSG00000260477 | 0.789937541 | 2.306232834 | 0.012487093 | RP11-553E24.2 | lincRNA |
| ENSG00000261441 | 16.42735963 | -3.138396531 | 1.56E-19 | RP11-217B1.2 | antisense |
| ENSG00000140519 | 12.21418927 | 2.065019042 | 7.18E-06 | RHCG | protein_coding |
| ENSG00000166819 | 44.13573077 | -2.274328013 | 2.42E-06 | PLIN1 | protein_coding |
| ENSG00000277987 | 0.408882294 | -2.272451295 | 0.025452666 | #N/A | #N/A |
| ENSG00000188536 | 49.92854877 | 2.582578931 | 1.48E-08 | HBA2 | protein_coding |
| ENSG00000206172 | 20.69170389 | 3.044064607 | 4.43E-09 | HBA1 | protein_coding |
| ENSG00000086506 | 1.043101601 | 2.688737563 | 0.00104353 | HBQ1 | protein_coding |
| ENSG00000261713 | 23.99871804 | -2.086033436 | 0.002389777 | SSTR5-AS1 | processed_transcript |
| ENSG00000260532 | 1.067510834 | 2.757613938 | 0.013476873 | LA16c-381G6.1 | lincRNA |
| ENSG00000205890 | 0.71951424 | 2.157851208 | 0.021512861 | RP11-473M20.5 | antisense |
| ENSG00000261889 | 3.96704064 | -2.864512955 | 6.71E-13 | RP11-473M20.16 | lincRNA |
| ENSG00000260289 | 0.651060995 | 2.392047786 | 0.079337877 | CTD-2535I10.1 | lincRNA |
| ENSG00000260003 | 4.630634673 | 4.568241137 | 1.54E-07 | RP11-279O17.2 | lincRNA |
| ENSG00000261319 | 3.570748802 | 3.530690862 | 4.31E-05 | RP11-279O17.1 | lincRNA |
| ENSG00000260058 | 0.550392096 | 2.164374626 | 0.107821583 | RP11-279O17.3 | lincRNA |
| ENSG00000232258 | 1.234975084 | 2.937486327 | 0.001902405 | TMEM114 | protein_coding |
| ENSG00000261617 | 10.8722455 | -2.365885034 | 3.57E-07 | RP11-243A14.1 | lincRNA |
| ENSG00000261810 | 0.791139967 | 2.310437458 | 0.010510796 | RP11-895K13.2 | antisense |
| ENSG00000261647 | 1.417278012 | 2.826990827 | 0.000985378 | IMPDH1P11 | pseudogene |
| ENSG00000262322 | 0.84422785 | 2.431300007 | 0.004786905 | RP11-109M19.2 | pseudogene |
| ENSG00000262703 | 3.179834883 | 2.130560304 | 0.000138592 | RP11-485G7.6 | antisense |
| ENSG00000262801 | 0.63167964 | -2.377521606 | 0.004485516 | U91319.1 | lincRNA |
| ENSG00000207294 | 0.622982531 | -2.115962305 | 0.003716606 | Y_RNA | misc_RNA |
| ENSG00000133392 | 32.55743983 | 2.059069998 | 2.65E-05 | MYH11 | protein_coding |
| ENSG00000122254 | 16.28862961 | -3.239851856 | 4.19E-13 | HS3ST2 | protein_coding |
| ENSG00000166869 | 8.408455079 | 5.426331382 | 4.07E-05 | CHP2 | protein_coding |
| ENSG00000261089 | 2.455356943 | 2.097176485 | 0.001150853 | RP11-435I10.3 | pseudogene |
| ENSG00000239791 | 0.96292002 | -2.160809717 | 0.000519398 | AC002310.7 | antisense |
| ENSG00000261599 | 0.893507218 | 2.109551174 | 0.061316764 | HERC2P8 | pseudogene |
| ENSG00000260078 | 1.064513301 | 2.025515371 | 0.032245974 | RP11-44F14.1 | pseudogene |
| ENSG00000261804 | 1.736376141 | 3.797217713 | 0.019213958 | RP11-44F14.2 | lincRNA |
| ENSG00000102891 | 0.609218974 | 2.270210207 | 0.097812678 | MT4 | protein_coding |
| ENSG00000205361 | 0.388651123 | -2.461104992 | 0.039056414 | MT1DP | pseudogene |
| ENSG00000260600 | 0.575539479 | 2.21047719 | 0.038045268 | RP11-109D24.1 | sense_intronic |
| ENSG00000039068 | 18.489856 | 4.616545877 | 5.13E-05 | CDH1 | protein_coding |
| ENSG00000140873 | 2.897057349 | 2.382235242 | 0.008923393 | ADAMTS18 | protein_coding |
| ENSG00000086696 | 0.877401289 | 2.11056114 | 0.030330947 | HSD17B2 | protein_coding |
| ENSG00000268388 | 0.487903726 | -2.1870284 | 0.174097436 | FENDRR | lincRNA |
| ENSG00000260026 | 1.979138339 | 2.060435207 | 0.003554307 | CTD-2015G9.1 | lincRNA |
| ENSG00000174990 | 1.171441807 | -2.437813653 | 0.000452549 | CA5A | protein_coding |
| ENSG00000178773 | 1.782428368 | 2.156473565 | 0.004254257 | CPNE7 | protein_coding |
| ENSG00000180090 | 0.703459408 | 2.150394124 | 0.068567951 | OR3A1 | protein_coding |
| ENSG00000167741 | 5.540017022 | 2.81456606 | 0.002293984 | GGT6 | protein_coding |
| ENSG00000285593 | 0.518894512 | 2.065888545 | 0.066896482 | #N/A | #N/A |
| ENSG00000132518 | 1.073725346 | -2.607385117 | 5.81E-05 | GUCY2D | protein_coding |
| ENSG00000179593 | 3.690163971 | -2.189177835 | 0.00721143 | ALOX15B | protein_coding |
| ENSG00000179477 | 3.589032416 | 3.84693784 | 0.000154798 | ALOX12B | protein_coding |
| ENSG00000179148 | 4.2012172 | 4.415783249 | 9.49E-05 | ALOXE3 | protein_coding |
| ENSG00000265975 | 1.227150035 | -2.037080061 | 0.001856208 | CTB-41I6.2 | lincRNA |
| ENSG00000184544 | 33.57545949 | -2.792085982 | 5.12E-07 | DHRS7C | protein_coding |
| ENSG00000265163 | 0.742026427 | -2.04118722 | 0.047595692 | CDRT8 | lincRNA |
| ENSG00000214946 | 0.818120045 | 2.354285889 | 0.017124934 | TBC1D26 | protein_coding |
| ENSG00000264673 | 1.885548939 | 2.907510222 | 0.007229825 | RP11-92B11.3 | antisense |
| ENSG00000108551 | 63.98302905 | -2.087263833 | 6.49E-12 | RASD1 | protein_coding |
| ENSG00000226521 | 0.667152029 | 2.4346506 | 0.046818873 | AC126365.1 | pseudogene |
| ENSG00000264660 | 1.479269837 | 2.538809105 | 0.001202085 | RP11-381P6.1 | antisense |
| ENSG00000260458 | 0.594667591 | 2.241230867 | 0.041436992 | KCNJ12 | protein_coding |
| ENSG00000237575 | 1.17297157 | 2.184953726 | 0.011137706 | PYY2 | pseudogene |
| ENSG00000109101 | 1.845190495 | 2.867661815 | 0.015183931 | FOXN1 | protein_coding |
| ENSG00000279668 | 16.91196912 | -2.693950899 | 5.30E-11 | #N/A | #N/A |
| ENSG00000181374 | 3.798288017 | -2.03795273 | 0.000170441 | CCL13 | protein_coding |
| ENSG00000131746 | 10.63294013 | 3.009816736 | 0.000161526 | TNS4 | protein_coding |
| ENSG00000204897 | 2.395407136 | 3.595290889 | 0.04615517 | KRT25 | protein_coding |
| ENSG00000171446 | 2.195933277 | 2.81044074 | 0.011179863 | KRT27 | protein_coding |
| ENSG00000186395 | 2348.460585 | 2.938449154 | 2.30E-06 | KRT10 | protein_coding |
| ENSG00000197079 | 0.645453367 | 2.372613319 | 0.372473403 | KRT35 | protein_coding |
| ENSG00000171346 | 35.17826797 | 8.142108722 | 2.56E-07 | KRT15 | protein_coding |
| ENSG00000186847 | 375.945003 | 25.34298698 | 6.18E-45 | KRT14 | protein_coding |
| ENSG00000186832 | 25.24784149 | 6.69720644 | 0.00013435 | KRT16 | protein_coding |
| ENSG00000128422 | 28.81777513 | 3.918048811 | 0.002229498 | KRT17 | protein_coding |
| ENSG00000073670 | 122.7253412 | -2.308956432 | 8.40E-12 | ADAM11 | protein_coding |
| ENSG00000064195 | 3.472180546 | 3.802122185 | 0.000143965 | DLX3 | protein_coding |
| ENSG00000263317 | 0.53140753 | -2.103141708 | 0.056931768 | RP11-429O1.1 | lincRNA |
| ENSG00000212195 | 45.63610231 | 2.280914386 | 1.17E-16 | U3 | snoRNA |
| ENSG00000265971 | 0.810692702 | 2.688360001 | 0.001498964 | RP11-269G24.6 | lincRNA |
| ENSG00000172782 | 0.863944334 | 2.378206845 | 0.014983386 | FADS6 | protein_coding |
| ENSG00000167880 | 25.09662934 | 2.148888112 | 9.35E-05 | EVPL | protein_coding |
| ENSG00000275479 | 0.789380408 | -2.663487525 | 1.51E-05 | #N/A | #N/A |
| ENSG00000206422 | 0.281051509 | -2.092911748 | 0.188991094 | LRRC30 | protein_coding |
| ENSG00000266604 | 0.466467444 | -2.360097207 | 0.022202243 | RP11-856M7.6 | lincRNA |
| ENSG00000267733 | 1.626641341 | -2.045040193 | 0.000876964 | RP11-64C12.3 | pseudogene |
| ENSG00000274214 | 0.706390726 | 2.153216453 | 0.127181671 | #N/A | #N/A |
| ENSG00000265015 | 0.371841705 | -2.590545835 | 0.057530884 | RP11-454P7.3 | pseudogene |
| ENSG00000171885 | 21.0686578 | -2.805271091 | 2.04E-05 | AQP4 | protein_coding |
| ENSG00000266549 | 0.466460925 | -2.791766746 | 0.002761451 | RP11-526I8.2 | pseudogene |
| ENSG00000134762 | 99.7320516 | 4.778736731 | 9.13E-10 | DSC3 | protein_coding |
| ENSG00000134760 | 207.956762 | 2.665790212 | 3.50E-07 | DSG1 | protein_coding |
| ENSG00000134757 | 10.95703023 | 4.843960504 | 4.27E-05 | DSG3 | protein_coding |
| ENSG00000268566 | 0.5422071 | 2.131386081 | 0.041927445 | RP11-687D19.1 | lincRNA |
| ENSG00000267374 | 2.079779161 | 3.051944541 | 0.000518782 | LINC00669 | lincRNA |
| ENSG00000177511 | 0.769276937 | 2.283114035 | 0.021402258 | ST8SIA3 | protein_coding |
| ENSG00000224367 | 8.995886662 | 3.127491849 | 2.45E-07 | OACYLP | pseudogene |
| ENSG00000267513 | 0.344663919 | -2.150929469 | 0.119533402 | RP11-622J9.1 | pseudogene |
| ENSG00000206075 | 36.00773692 | 6.926025844 | 1.48E-05 | SERPINB5 | protein_coding |
| ENSG00000166634 | 2.701628011 | 4.43286172 | 9.22E-05 | SERPINB12 | protein_coding |
| ENSG00000197641 | 2.810704216 | 4.490764824 | 0.000505838 | SERPINB13 | protein_coding |
| ENSG00000206073 | 1.56645914 | 2.27323678 | 0.066857518 | SERPINB4 | protein_coding |
| ENSG00000057149 | 1.413581891 | 3.157668148 | 0.065535007 | SERPINB3 | protein_coding |
| ENSG00000166396 | 13.39842053 | 4.446461227 | 0.000468897 | SERPINB7 | protein_coding |
| ENSG00000197632 | 3.941151876 | 3.645712342 | 0.000375774 | SERPINB2 | protein_coding |
| ENSG00000263711 | 3.204666686 | 2.290094576 | 0.003950374 | RP11-169F17.1 | protein_coding |
| ENSG00000259779 | 1.954964802 | 2.630229222 | 0.001050382 | RP11-231E4.2 | lincRNA |
| ENSG00000215397 | 0.713712454 | 2.523089061 | 0.009394631 | SCRT2 | protein_coding |
| ENSG00000171864 | 8.486176005 | 4.790816696 | 4.50E-08 | PRND | protein_coding |
| ENSG00000125869 | 7.791959636 | 2.9510773 | 2.97E-08 | LAMP5 | protein_coding |
| ENSG00000101349 | 2.174277982 | 3.448328327 | 0.002450204 | PAK7 | protein_coding |
| ENSG00000226875 | 0.512914308 | -2.086743402 | 0.081167098 | ZNF877P | pseudogene |
| ENSG00000170373 | 3.6583032 | 3.281578995 | 0.005874796 | CST1 | protein_coding |
| ENSG00000170369 | 7.80055386 | 3.836274521 | 3.60E-08 | CST2 | protein_coding |
| ENSG00000125998 | 4.206208073 | 2.737925852 | 0.023725541 | FAM83C | protein_coding |
| ENSG00000237063 | 0.455351354 | -2.152064508 | 0.030596872 | RP4-550H1.5 | lincRNA |
| ENSG00000182035 | 1.236227265 | -2.110821711 | 0.000129231 | ADIG | protein_coding |
| ENSG00000124143 | 1.215372664 | 2.205793766 | 0.013721934 | ARHGAP40 | protein_coding |
| ENSG00000261431 | 0.773472419 | -2.069257119 | 0.005505276 | RP4-616B8.4 | antisense |
| ENSG00000223190 | 0.814330273 | 2.309598979 | 0.028522177 | RN7SKP100 | misc_RNA |
| ENSG00000101074 | 3.857114597 | 2.090235806 | 7.67E-05 | R3HDML | protein_coding |
| ENSG00000175121 | 8.052096211 | 5.036748362 | 0.000122896 | WFDC5 | protein_coding |
| ENSG00000168703 | 2.822181324 | 3.499026786 | 0.007342065 | WFDC12 | protein_coding |
| ENSG00000124102 | 5.37822055 | 4.446083168 | 0.001000263 | PI3 | protein_coding |
| ENSG00000054803 | 2.47795149 | 3.962456616 | 2.67E-05 | CBLN4 | protein_coding |
| ENSG00000124253 | 4.673977808 | -2.996034178 | 0.00073835 | PCK1 | protein_coding |
| ENSG00000124205 | 9.141032287 | 3.045155039 | 1.60E-07 | EDN3 | protein_coding |
| ENSG00000238777 | 1.072098001 | 2.015952577 | 0.029152816 | RNU7-141P | snRNA |
| ENSG00000276786 | 0.21149074 | -2.214799359 | 0.372572461 | #N/A | #N/A |
| ENSG00000229882 | 1.276726541 | 3.031216405 | 0.000524864 | RP13-30A9.2 | antisense |
| ENSG00000101187 | 61.22765328 | -2.590566287 | 1.24E-24 | SLCO4A1 | protein_coding |
| ENSG00000232803 | 7.855823303 | -2.397418765 | 1.25E-14 | RP11-93B14.5 | antisense |
| ENSG00000075043 | 3.458642537 | 2.491809879 | 6.03E-05 | KCNQ2 | protein_coding |
| ENSG00000125508 | 0.886328601 | 2.462281502 | 0.005628668 | SRMS | protein_coding |
| ENSG00000099617 | 1.397913314 | 2.073999331 | 0.011738268 | EFNA2 | protein_coding |
| ENSG00000161082 | 1.067598691 | 2.039962149 | 0.037002895 | CELF5 | protein_coding |
| ENSG00000223573 | 14.63049551 | 2.143758241 | 3.54E-06 | TINCR | lincRNA |
| ENSG00000174898 | 1.126451679 | 2.118717527 | 0.017221826 | CATSPERD | protein_coding |
| ENSG00000171124 | 1.114700239 | 2.438896078 | 0.003795592 | FUT3 | protein_coding |
| ENSG00000167769 | 3.557351792 | 4.833481484 | 0.000288328 | ACER1 | protein_coding |
| ENSG00000275234 | 1.081363075 | 2.047039622 | 0.039866306 | #N/A | #N/A |
| ENSG00000125726 | 1.327853405 | 2.705120253 | 0.058532419 | CD70 | protein_coding |
| ENSG00000125730 | 1634.745944 | -3.06603756 | 1.17E-20 | C3 | protein_coding |
| ENSG00000276980 | 2.078912315 | -2.835461373 | 5.19E-09 | #N/A | #N/A |
| ENSG00000182566 | 3.689271028 | -2.740031147 | 8.26E-11 | CLEC4G | protein_coding |
| ENSG00000180739 | 9.721879521 | 2.549473653 | 4.86E-11 | S1PR5 | protein_coding |
| ENSG00000129354 | 1.828536009 | 3.192159832 | 0.001615435 | AP1M2 | protein_coding |
| ENSG00000131355 | 4.010463633 | 2.6907007 | 1.24E-05 | EMR3 | protein_coding |
| ENSG00000105143 | 0.6919893 | 2.44696473 | 0.074297681 | SLC1A6 | protein_coding |
| ENSG00000105141 | 43.94375781 | 8.463170193 | 5.34E-07 | CASP14 | protein_coding |
| ENSG00000171954 | 3.264918211 | 3.713663888 | 0.00031929 | CYP4F22 | protein_coding |
| ENSG00000186529 | 7.205111969 | 3.043310916 | 5.75E-07 | CYP4F3 | protein_coding |
| ENSG00000105664 | 123.831063 | 2.86179271 | 8.22E-07 | COMP | protein_coding |
| ENSG00000268105 | 1.428129537 | 2.128258156 | 0.005822297 | RP11-369G6.2 | pseudogene |
| ENSG00000266976 | 1.368133396 | -2.530015155 | 0.000290384 | AC079466.1 | lincRNA |
| ENSG00000221857 | 0.488319745 | 2.010970569 | 0.083509433 | CTD-2527I21.4 | processed_transcript |
| ENSG00000105695 | 1.760318339 | 2.1254877 | 0.032420625 | MAG | protein_coding |
| ENSG00000126262 | 3.291180291 | 2.376421894 | 5.23E-05 | FFAR2 | protein_coding |
| ENSG00000188508 | 66.64601979 | 6.23529518 | 3.99E-07 | KRTDAP | protein_coding |
| ENSG00000189001 | 241.1444164 | 10.75357911 | 4.96E-09 | SBSN | protein_coding |
| ENSG00000205076 | 26.87466133 | 7.431657138 | 2.59E-05 | LGALS7 | protein_coding |
| ENSG00000178934 | 40.59734063 | 7.384880638 | 9.27E-06 | LGALS7B | protein_coding |
| ENSG00000183760 | 1.339989412 | 3.051669931 | 0.001783128 | PAPL | protein_coding |
| ENSG00000188505 | 13.76154593 | 5.817987383 | 4.68E-05 | NCCRP1 | protein_coding |
| ENSG00000197408 | 0.826705257 | 2.724153812 | 0.074145508 | CYP2B6 | protein_coding |
| ENSG00000105369 | 3.824733743 | 2.066780186 | 0.01677431 | CD79A | protein_coding |
| ENSG00000124466 | 28.85874088 | 3.058083732 | 1.63E-05 | LYPD3 | protein_coding |
| ENSG00000273777 | 0.817610572 | -2.00584284 | 0.030110575 | #N/A | #N/A |
| ENSG00000142273 | 0.883372346 | 2.81665321 | 0.023673782 | CBLC | protein_coding |
| ENSG00000125740 | 94.07294793 | 2.560988277 | 4.08E-07 | FOSB | protein_coding |
| ENSG00000188624 | 0.847669656 | 2.386398136 | 0.013260809 | IGFL3 | protein_coding |
| ENSG00000268621 | 1.074813724 | 2.742431819 | 0.004560057 | AC006262.5 | lincRNA |
| ENSG00000275719 | 0.939752046 | -3.144856059 | 5.01E-05 | #N/A | #N/A |
| ENSG00000088002 | 5.912639943 | 3.254133795 | 0.000658872 | SULT2B1 | protein_coding |
| ENSG00000142538 | 0.969981536 | 2.266160454 | 0.018628993 | PTH2 | protein_coding |
| ENSG00000261949 | 0.672913254 | 2.448849588 | 0.018921457 | GFY | protein_coding |
| ENSG00000167754 | 8.455836196 | 6.084991816 | 0.000106329 | KLK5 | protein_coding |
| ENSG00000169035 | 10.50829107 | 6.39829935 | 5.09E-05 | KLK7 | protein_coding |
| ENSG00000129455 | 1.658487941 | 3.7275368 | 0.002357122 | KLK8 | protein_coding |
| ENSG00000167757 | 7.780534043 | 2.769389053 | 0.000336606 | KLK11 | protein_coding |
| ENSG00000269072 | 0.900950418 | -2.038159691 | 0.002003041 | CTD-3187F8.14 | antisense |
| ENSG00000171101 | 31.86447637 | 3.189859601 | 4.37E-21 | SIGLEC17P | pseudogene |
| ENSG00000273837 | 1.493688252 | -2.104121483 | 0.00550925 | #N/A | #N/A |
| ENSG00000204595 | 1.474285695 | 2.860497138 | 0.000173335 | DPRX | protein_coding |
| ENSG00000167608 | 1.679319879 | 3.082330898 | 0.000947072 | TMC4 | protein_coding |
| ENSG00000244482 | 0.498547262 | -2.062514139 | 0.051927536 | LILRA6 | protein_coding |
| ENSG00000105609 | 3.74140204 | -2.270973043 | 0.017761503 | LILRB5 | protein_coding |
| ENSG00000129990 | 1.156752646 | 2.175945795 | 0.010935197 | SYT5 | protein_coding |
| ENSG00000279063 | 0.838265824 | 2.395164461 | 0.014998551 | #N/A | #N/A |
| ENSG00000223638 | 3.999714865 | 2.884821078 | 0.000955608 | RFPL4A | protein_coding |
| ENSG00000269855 | 1.288808848 | 2.307257896 | 0.011005383 | AC012313.1 | protein_coding |
| ENSG00000233995 | 0.841476656 | 2.738617792 | 0.026622289 | KB-67B5.12 | pseudogene |
| ENSG00000211642 | 0.397849987 | -2.538436929 | 0.15379449 | IGLV10-54 | IG_V_gene |
| ENSG00000211664 | 0.26240998 | -2.258300823 | 0.276057485 | IGLV2-18 | IG_V_gene |
| ENSG00000229770 | 1.785915335 | 2.484257119 | 0.004668162 | CTA-796E4.4 | antisense |
| ENSG00000100095 | 214.4345172 | 2.893910707 | 2.65E-22 | SEZ6L | protein_coding |
| ENSG00000224192 | 5.090481772 | 3.234979036 | 1.88E-06 | RP11-259P1.1 | antisense |
| ENSG00000226912 | 0.732968842 | -2.441550584 | 0.005021204 | ISCA2P1 | pseudogene |
| ENSG00000227117 | 1.759712706 | 2.139915387 | 0.004844235 | CTA-85E5.10 | antisense |
| ENSG00000214491 | 3.709268196 | 2.292583848 | 0.002653743 | SEC14L6 | protein_coding |
| ENSG00000205856 | 1.107711631 | 2.10097969 | 0.016801855 | C22orf42 | protein_coding |
| ENSG00000184459 | 2.613103542 | 3.716185631 | 0.000170406 | BPIFC | protein_coding |
| ENSG00000188064 | 3.111275002 | 3.358130971 | 0.006927002 | WNT7B | protein_coding |
| ENSG00000224271 | 0.892438081 | 2.102126208 | 0.061926946 | RP11-191L9.4 | lincRNA |
| ENSG00000217442 | 1.281752635 | 2.284228762 | 0.013600486 | SYCE3 | protein_coding |
| ENSG00000277739 | 0.512765844 | -2.289879045 | 0.083121953 | #N/A | #N/A |
| ENSG00000281181 | 3.704381415 | 3.377589854 | 0.001104144 | #N/A | #N/A |
| ENSG00000229425 | 0.969275092 | 2.251011819 | 0.016958902 | AJ006998.2 | lincRNA |
| ENSG00000206802 | 0.348936238 | -2.143269395 | 0.061180137 | RNU6-926P | snRNA |
| ENSG00000229025 | 0.664035397 | -2.383877258 | 0.003205311 | AP001595.1 | lincRNA |
| ENSG00000156265 | 469.4029081 | 2.811088199 | 1.14E-15 | MAP3K7CL | protein_coding |
| ENSG00000201984 | 2.426944239 | 2.638600085 | 0.000637323 | Y_RNA | misc_RNA |
| ENSG00000212479 | 1.646708489 | 2.378842144 | 0.00461698 | U3 | snoRNA |
| ENSG00000224649 | 4.267276882 | 2.767771211 | 7.13E-06 | AF124730.4 | antisense |
| ENSG00000156284 | 1.1036115 | 3.146430697 | 0.013249607 | CLDN8 | protein_coding |
| ENSG00000231106 | 1.040980734 | -2.105092793 | 0.011806218 | AP000688.8 | lincRNA |
| ENSG00000226012 | 0.865187326 | 2.420113647 | 0.008053662 | AP001434.2 | lincRNA |
| ENSG00000183778 | 49.36360018 | 2.01996701 | 1.40E-07 | B3GALT5 | protein_coding |
| ENSG00000225330 | 2.150815773 | 2.404635747 | 0.001208389 | AF064860.5 | sense_overlapping |
| ENSG00000231713 | 1.528486992 | 2.587353229 | 0.002358097 | AF064860.7 | lincRNA |
| ENSG00000171587 | 1.52460714 | 2.218242993 | 0.013946536 | DSCAM | protein_coding |
| ENSG00000184012 | 1.485861704 | 2.516172945 | 0.008019287 | TMPRSS2 | protein_coding |
| ENSG00000277352 | 0.702357053 | -2.154880754 | 0.005719055 | #N/A | #N/A |
| ENSG00000175894 | 35.32117627 | -3.766964164 | 2.57E-09 | TSPEAR | protein_coding |
| ENSG00000235890 | 23.11437216 | -3.223353898 | 3.53E-13 | TSPEAR-AS1 | antisense |
| ENSG00000182912 | 33.07681105 | -3.816739175 | 4.12E-10 | C21orf90 | protein_coding |
| ENSG00000205445 | 0.56712005 | -2.136342349 | 0.006579339 | KRTAP10-2 | protein_coding |
| ENSG00000215454 | 0.443934377 | -2.602805016 | 0.094074543 | KRTAP10-4 | protein_coding |
| ENSG00000272804 | 0.842964942 | -3.088881618 | 0.000148761 | AP001067.1 | protein_coding |
| ENSG00000187766 | 0.568472988 | -3.098208849 | 0.023461366 | KRTAP10-8 | protein_coding |
| ENSG00000221837 | 1.098920737 | -3.869529308 | 0.000242626 | KRTAP10-9 | protein_coding |
| ENSG00000243489 | 1.738124506 | -3.318240391 | 3.52E-05 | KRTAP10-11 | protein_coding |
| ENSG00000212933 | 0.605354358 | -3.05706264 | 0.010268488 | KRTAP12-4 | protein_coding |
| ENSG00000205439 | 0.864486527 | -3.609780911 | 0.001967646 | KRTAP12-3 | protein_coding |
| ENSG00000229880 | 6.442010474 | -4.48696748 | 3.32E-08 | IMMTP1 | pseudogene |
| ENSG00000187175 | 0.757935116 | -3.077580431 | 0.001588265 | KRTAP12-1 | protein_coding |
| ENSG00000276647 | 0.337005785 | -2.877490861 | 0.39830339 | #N/A | #N/A |
| ENSG00000189169 | 0.869287578 | -2.796830728 | 0.000700677 | KRTAP10-12 | protein_coding |
| ENSG00000236382 | 0.961564505 | -3.188047526 | 0.000710709 | KRTAP10-13P | pseudogene |
| ENSG00000226115 | 0.37135454 | -2.370072064 | 0.087635335 | AP001476.3 | lincRNA |
