# Supplementary material for: Dysfunctional Network and Mutation Genes of Hypertrophic Cardiomyopathy
Source: J Healthc Eng. 2022 Jan 28;2022:8680178. doi: 10.1155/2022/8680178 (PMC8816546; doi:10.1155/2022/8680178)
Supplement: Supplementary Materials — Table S1: differentially expressed genes between HCM patients and the control group. Table S2: SNVs in HCM patients. [file 8680178.f1.zip › 8680178.f1/Table S2.docx]

Table S2. SNVs in HCM patients.

| Hugo_Symbol | ID | NCBI_Build | Chromosome | Start_Position | End_position | Strand | Variant_Type | Reference_Allele | Tumor_Seq_Allele1 | Tumor_Seq_Allele2 | Tumor_Sample_Barcode | Variant_Classification | SRR8586409_1.fastq.gz.bam | DP |
| --- | --- | --- | --- | --- | --- | --- | --- | --- | --- | --- | --- | --- | --- | --- |
| RP5-857K21.4 | rs6594029 | 37 | 1 | 630026 | 630026 | + | SNP | C | T | T | SRR8586409 | Missense_Mutation | 1/1:5,221:226:99:7747,594,0 | 370;ANN=T|intron_variant|MODIFIER|RP5-857K21.4|ENSG00000230021|transcript|ENST00000440200|lincRNA|1/2|n.169+25386G>A|||||| |
| HMGCS2-REG4 | rs587687250 | 37 | 1 | 120329559 | 120329559 | + | SNP | A | A | G | SRR8586409 | Missense_Mutation | 0/1:103,42:145:99:829,0,2840 | 145;ANN=G|intergenic_region|MODIFIER|HMGCS2-REG4|ENSG00000134240-ENSG00000134193|intergenic_region|ENSG00000134240-ENSG00000134193|||n.120329559A>G|||||| |
| NOTCH2 | rs1699761 | 37 | 1 | 120489416 | 120489416 | + | SNP | C | C | A | SRR8586409 | Nonsense_Mutation | 0/1:176,136:312:99:3519,0,4277 | 313;ANN=A|downstream_gene_variant|MODIFIER|NOTCH2|ENSG00000134250|transcript|ENST00000479412|retained_intron||n.*3327G>T|||||3327|,A|intron_variant|MODIFIER|NOTCH2|ENSG00000134250|transcript|ENST00000256646|protein_coding|17/33|c.2752+1621G>T|||||| |
| NOTCH2 | rs113514538 | 37 | 1 | 120489584 | 120489584 | + | SNP | T | T | G | SRR8586409 | Nonsense_Mutation | 0/1:147,79:226:99:1823,0,4066 | 226;ANN=G|downstream_gene_variant|MODIFIER|NOTCH2|ENSG00000134250|transcript|ENST00000479412|retained_intron||n.*3159A>C|||||3159|,G|intron_variant|MODIFIER|NOTCH2|ENSG00000134250|transcript|ENST00000256646|protein_coding|17/33|c.2752+1453A>C|||||| |
| NOTCH2 | rs74872612 | 37 | 1 | 120489620 | 120489620 | + | SNP | G | G | T | SRR8586409 | Nonsense_Mutation | 0/1:168,49:217:99:904,0,4708 | 217;ANN=T|downstream_gene_variant|MODIFIER|NOTCH2|ENSG00000134250|transcript|ENST00000479412|retained_intron||n.*3123C>A|||||3123|,T|intron_variant|MODIFIER|NOTCH2|ENSG00000134250|transcript|ENST00000256646|protein_coding|17/33|c.2752+1417C>A|||||| |
| RP11-763B22.9 | rs1664022 | 37 | 1 | 148889827 | 148889827 | + | SNP | G | G | T | SRR8586409 | Missense_Mutation | 0/1:394,45:439:99:230,0,11253 | 441;ANN=T|intron_variant|MODIFIER|RP11-763B22.9|ENSG00000231448|transcript|ENST00000444424|unprocessed_pseudogene|8/9|n.1017+138G>T|||||| |
| DRD5P2 | rs1778581 | 37 | 1 | 148903195 | 148903195 | + | SNP | C | C | T | SRR8586409 | Nonsense_Mutation | 0/1:47,11:58:99:231,0,1376 | 58;ANN=T|downstream_gene_variant|MODIFIER|DRD5P2|ENSG00000175658|transcript|ENST00000535197|processed_pseudogene||n.*92C>T|||||92|,T|downstream_gene_variant|MODIFIER|DRD5P2|ENSG00000175658|transcript|ENST00000309892|processed_pseudogene||n.*282C>T|||||282|,T|non_coding_transcript_exon_variant|MODIFIER|DRD5P2|ENSG00000175658|transcript|ENST00000421395|processed_pseudogene|2/2|n.1492C>T|||||| |
| RP11-14N7.2 | rs138706944 | 37 | 1 | 148924965 | 148924965 | + | INS | T | T | TA | SRR8586409 | Frame_Shift_Ins | 0/1:20,10:30:99:284,0,659 | 30;ANN=TA|upstream_gene_variant|MODIFIER|RP11-14N7.2|ENSG00000232527|transcript|ENST00000539543|lincRNA||n.-3380_-3379insA|||||3379|,TA|upstream_gene_variant|MODIFIER|RP11-14N7.2|ENSG00000232527|transcript|ENST00000420597|lincRNA||n.-3357_-3356insA|||||3356|,TA|upstream_gene_variant|MODIFIER|RP11-14N7.2|ENSG00000232527|transcript|ENST00000452399|lincRNA||n.-3357_-3356insA|||||3356|,TA|upstream_gene_variant|MODIFIER|RP11-14N7.2|ENSG00000232527|transcript|ENST00000294715|lincRNA||n.-3366_-3365insA|||||3365|,TA|intergenic_region|MODIFIER|RNA5SP59-RP11-14N7.2|ENSG00000222854-ENSG00000232527|intergenic_region|ENSG00000222854-ENSG00000232527|||n.148924965_148924966insA|||||| |
| RP11-14N7.2 | rs512570 | 37 | 1 | 148933778 | 148933778 | + | SNP | A | A | G | SRR8586409 | Nonsense_Mutation | 0/1:23,44:67:99:1281,0,621 | 67;ANN=G|downstream_gene_variant|MODIFIER|RP11-14N7.2|ENSG00000232527|transcript|ENST00000420597|lincRNA||n.*858A>G|||||858|,G|downstream_gene_variant|MODIFIER|RP11-14N7.2|ENSG00000232527|transcript|ENST00000294715|lincRNA||n.*410A>G|||||410|,G|downstream_gene_variant|MODIFIER|RP11-14N7.2|ENSG00000232527|transcript|ENST00000457390|lincRNA||n.*199A>G|||||199|,G|intron_variant|MODIFIER|RP11-14N7.2|ENSG00000232527|transcript|ENST00000539543|lincRNA|3/3|n.254+410A>G||||||,G|intron_variant|MODIFIER|RP11-14N7.2|ENSG00000232527|transcript|ENST00000452399|lincRNA|2/2|n.199+858A>G|||||| |
| RP11-385F5.5 | rs113420058 | 37 | 1 | 236695572 | 236695572 | + | DEL | CT | CT | C | SRR8586409 | Frame_Shift_Del | 0/1:19,36:55:99:843,0,508 | 60;ANN=C|downstream_gene_variant|MODIFIER|RP11-385F5.5|ENSG00000273058|transcript|ENST00000608547|antisense||n.*3900delA|||||3900|,C|intron_variant|MODIFIER|LGALS8|ENSG00000116977|transcript|ENST00000352231|protein_coding|3/11|c.46-5224delT||||||,C|intron_variant|MODIFIER|LGALS8|ENSG00000116977|transcript|ENST00000481485|protein_coding|3/4|c.46-5224delT||||||WARNING_TRANSCRIPT_INCOMPLETE,C|intron_variant|MODIFIER|LGALS8|ENSG00000116977|transcript|ENST00000454943|protein_coding|4/7|c.46-5224delT||||||WARNING_TRANSCRIPT_INCOMPLETE,C|intron_variant|MODIFIER|LGALS8|ENSG00000116977|transcript|ENST00000527974|protein_coding|2/10|c.46-5224delT||||||,C|intron_variant|MODIFIER|LGALS8|ENSG00000116977|transcript|ENST00000430527|protein_coding|3/5|c.46-5224delT||||||WARNING_TRANSCRIPT_INCOMPLETE,C|intron_variant|MODIFIER|LGALS8|ENSG00000116977|transcript|ENST00000406509|protein_coding|5/10|c.46-5224delT||||||WARNING_TRANSCRIPT_INCOMPLETE,C|intron_variant|MODIFIER|LGALS8|ENSG00000116977|transcript|ENST00000526589|protein_coding|5/13|c.46-5224delT||||||,C|intron_variant|MODIFIER|LGALS8|ENSG00000116977|transcript|ENST00000529489|protein_coding|3/3|c.46-5224delT||||||WARNING_TRANSCRIPT_NO_STOP_CODON,C|intron_variant|MODIFIER|LGALS8|ENSG00000116977|transcript|ENST00000341872|protein_coding|3/10|c.46-5224delT||||||,C|intron_variant|MODIFIER|LGALS8|ENSG00000116977|transcript|ENST00000450372|protein_coding|3/11|c.46-5224delT||||||,C|intron_variant|MODIFIER|LGALS8|ENSG00000116977|transcript|ENST00000366584|protein_coding|2/9|c.46-5224delT||||||,C|intron_variant|MODIFIER|LGALS8|ENSG00000116977|transcript|ENST00000238181|protein_coding|2/6|c.46-5224delT||||||WARNING_TRANSCRIPT_INCOMPLETE,C|intron_variant|MODIFIER|LGALS8|ENSG00000116977|transcript|ENST00000532826|retained_intron|2/3|n.226-5224delT||||||,C|intron_variant|MODIFIER|LGALS8|ENSG00000116977|transcript|ENST00000528782|retained_intron|2/4|n.226-5224delT||||||,C|intron_variant|MODIFIER|LGALS8|ENSG00000116977|transcript|ENST00000366583|retained_intron|2/6|n.222-5224delT||||||,C|intron_variant|MODIFIER|LGALS8|ENSG00000116977|transcript|ENST00000442397|nonsense_mediated_decay|2/4|c.46-5224delT||||||,C|intron_variant|MODIFIER|LGALS8|ENSG00000116977|transcript|ENST00000434231|nonsense_mediated_decay|2/4|c.45+6158delT||||||,C|intron_variant|MODIFIER|LGALS8|ENSG00000116977|transcript|ENST00000416919|protein_coding|2/8|c.46-5224delT||||||,C|intron_variant|MODIFIER|LGALS8|ENSG00000116977|transcript|ENST00000323938|protein_coding|2/9|c.46-5224delT||||||,C|intron_variant|MODIFIER|LGALS8|ENSG00000116977|transcript|ENST00000526634|protein_coding|2/9|c.46-5224delT||||||,C|intron_variant|MODIFIER|LGALS8|ENSG00000116977|transcript|ENST00000525042|protein_coding|1/7|c.46-5224delT|||||| |
| MTR-RPL35P1 | rs201902635 | 37 | 1 | 237077160 | 237077160 | + | SNP | T | T | G | SRR8586409 | Missense_Mutation | 0/1:354,40:394:99:225,0,9715 | 395;ANN=G|intergenic_region|MODIFIER|MTR-RPL35P1|ENSG00000116984-ENSG00000237991|intergenic_region|ENSG00000116984-ENSG00000237991|||n.237077160T>G|||||| |
| MTR-RPL35P1 | rs6686489 | 37 | 1 | 237091429 | 237091429 | + | SNP | G | G | T | SRR8586409 | Missense_Mutation | 0/1:42,94:136:99:2022,0,1043 | 200;ANN=T|intergenic_region|MODIFIER|MTR-RPL35P1|ENSG00000116984-ENSG00000237991|intergenic_region|ENSG00000116984-ENSG00000237991|||n.237091429G>T|||||| |
| MTR-RPL35P1 | rs6686490 | 37 | 1 | 237091430 | 237091430 | + | SNP | G | G | T | SRR8586409 | Missense_Mutation | 0/1:41,86:127:99:1927,0,1098 | 197;ANN=T|intergenic_region|MODIFIER|MTR-RPL35P1|ENSG00000116984-ENSG00000237991|intergenic_region|ENSG00000116984-ENSG00000237991|||n.237091430G>T|||||| |
| MTR-RPL35P1 | rs368474001 | 37 | 1 | 237091437 | 237091437 | + | SNP | C | C | G | SRR8586409 | Missense_Mutation | 0/1:104,27:131:99:484,0,2881 | 139;ANN=G|intergenic_region|MODIFIER|MTR-RPL35P1|ENSG00000116984-ENSG00000237991|intergenic_region|ENSG00000116984-ENSG00000237991|||n.237091437C>G|||||| |
| MIR4428 | rs750102784 | 37 | 1 | 237639197 | 237639197 | + | SNP | A | A | G | SRR8586409 | Nonsense_Mutation | 0/1:207,164:371:99:4724,0,6057 | 384;ANN=G|downstream_gene_variant|MODIFIER|MIR4428|ENSG00000266262|transcript|ENST00000584884|miRNA||n.*4706A>G|||||4706|,G|intron_variant|MODIFIER|RYR2|ENSG00000198626|transcript|ENST00000360064|protein_coding|18/106|c.1702+6710A>G||||||,G|intron_variant|MODIFIER|RYR2|ENSG00000198626|transcript|ENST00000366574|protein_coding|17/104|c.1708+6710A>G||||||,G|intron_variant|MODIFIER|RYR2|ENSG00000198626|transcript|ENST00000542537|protein_coding|16/103|c.1660+6710A>G||||||WARNING_TRANSCRIPT_NO_START_CODON |
| AMD1P1-RP11-51E20.1 | rs1830297 | 37 | 10 | 20820684 | 20820684 | + | SNP | G | G | C | SRR8586409 | Missense_Mutation | 0/1:104,94:198:99:2409,0,2775 | 198;ANN=C|intergenic_region|MODIFIER|AMD1P1-RP11-51E20.1|ENSG00000228339-ENSG00000270727|intergenic_region|ENSG00000228339-ENSG00000270727|||n.20820684G>C|||||| |
| PDE6C | rs11188311 | 37 | 10 | 95394941 | 95394941 | + | SNP | G | G | A | SRR8586409 | Missense_Mutation | 0/1:100,66:166:99:1623,0,2772 | 166;ANN=A|intron_variant|MODIFIER|PDE6C|ENSG00000095464|transcript|ENST00000371447|protein_coding|9/21|c.1269+277G>A|||||| |
| POLR2G | rs188468517 | 37 | 11 | 62527604 | 62527604 | + | SNP | G | G | A | SRR8586409 | Missense_Mutation | 0/1:863,80:943:99:701,0,36169 | 944;ANN=A|upstream_gene_variant|MODIFIER|POLR2G|ENSG00000168002|transcript|ENST00000301788|protein_coding||c.-1517G>A|||||1412|,A|upstream_gene_variant|MODIFIER|POLR2G|ENSG00000168002|transcript|ENST00000533442|protein_coding||c.-5077G>A|||||1433|WARNING_TRANSCRIPT_INCOMPLETE,A|upstream_gene_variant|MODIFIER|POLR2G|ENSG00000168002|transcript|ENST00000525455|nonsense_mediated_decay||c.-1517G>A|||||1448|,A|upstream_gene_variant|MODIFIER|POLR2G|ENSG00000168002|transcript|ENST00000524819|nonsense_mediated_decay||c.-1517G>A|||||1451|,A|upstream_gene_variant|MODIFIER|POLR2G|ENSG00000168002|transcript|ENST00000531944|nonsense_mediated_decay||c.-1517G>A|||||1485|,A|upstream_gene_variant|MODIFIER|POLR2G|ENSG00000168002|transcript|ENST00000527435|retained_intron||n.-1505G>A|||||1505|,A|upstream_gene_variant|MODIFIER|POLR2G|ENSG00000168002|transcript|ENST00000531996|nonsense_mediated_decay||c.-1663G>A|||||1663|WARNING_TRANSCRIPT_NO_START_CODON,A|upstream_gene_variant|MODIFIER|POLR2G|ENSG00000168002|transcript|ENST00000526368|retained_intron||n.-2622G>A|||||2622|,A|intergenic_region|MODIFIER|ZBTB3-POLR2G|ENSG00000185670-ENSG00000168002|intergenic_region|ENSG00000185670-ENSG00000168002|||n.62527604G>A|||||| |
| TSPAN9 | rs58837502 | 37 | 12 | 3211762 | 3211762 | + | SNP | T | T | C | SRR8586409 | Missense_Mutation | 0/1:31,91:122:99:2731,0,648 | 122;ANN=C|intron_variant|MODIFIER|TSPAN9|ENSG00000011105|transcript|ENST00000537971|protein_coding|1/7|c.-18+25143T>C||||||,C|intron_variant|MODIFIER|TSPAN9|ENSG00000011105|transcript|ENST00000444315|nonsense_mediated_decay|2/5|c.-18+18877T>C||||||,C|intron_variant|MODIFIER|TSPAN9|ENSG00000011105|transcript|ENST00000011898|protein_coding|2/8|c.-18+18877T>C|||||| |
| ADCY6 | rs754280693 | 37 | 12 | 49185703 | 49185703 | + | SNP | A | A | G | SRR8586409 | Missense_Mutation | 0/1:441,39:480:99:250,0,22684 | 480;ANN=G|upstream_gene_variant|MODIFIER|ADCY6|ENSG00000174233|transcript|ENST00000550422|protein_coding||c.-8486T>C|||||2883|,G|downstream_gene_variant|MODIFIER|RP11-579D7.4|ENSG00000257660|transcript|ENST00000549864|lincRNA||n.*1385A>G|||||1385|,G|downstream_gene_variant|MODIFIER|RP11-579D7.4|ENSG00000257660|transcript|ENST00000547774|lincRNA||n.*1385A>G|||||1385|,G|intergenic_region|MODIFIER|RP11-579D7.4-RP11-579D7.8|ENSG00000257660-ENSG00000271547|intergenic_region|ENSG00000257660-ENSG00000271547|||n.49185703A>G|||||| |
| ADCY6 | rs146680997 | 37 | 12 | 49185709 | 49185709 | + | SNP | A | A | G | SRR8586409 | Missense_Mutation | 0/1:446,40:486:99:307,0,21730 | 486;ANN=G|upstream_gene_variant|MODIFIER|ADCY6|ENSG00000174233|transcript|ENST00000550422|protein_coding||c.-8492T>C|||||2889|,G|downstream_gene_variant|MODIFIER|RP11-579D7.4|ENSG00000257660|transcript|ENST00000549864|lincRNA||n.*1391A>G|||||1391|,G|downstream_gene_variant|MODIFIER|RP11-579D7.4|ENSG00000257660|transcript|ENST00000547774|lincRNA||n.*1391A>G|||||1391|,G|intergenic_region|MODIFIER|RP11-579D7.4-RP11-579D7.8|ENSG00000257660-ENSG00000271547|intergenic_region|ENSG00000257660-ENSG00000271547|||n.49185709A>G|||||| |
| AL139319.1-RPL21P111 | rs71204715 | 37 | 13 | 79319949 | 79319949 | + | INS | C | C | CTTT | SRR8586409 | In_Frame_Ins | 0/1:12,12:24:99:254,0,433 | 43;ANN=CTTT|intergenic_region|MODIFIER|AL139319.1-RPL21P111|ENSG00000263868-ENSG00000237109|intergenic_region|ENSG00000263868-ENSG00000237109|||n.79319949_79319950insTTT|||||| |
| PRMT5-AS1 | rs753335327 | 37 | 14 | 23384640 | 23384640 | + | SNP | C | C | T | SRR8586409 | Missense_Mutation | 0/1:415,166:581:99:3813,0,32502 | 581;ANN=T|upstream_gene_variant|MODIFIER|PRMT5-AS1|ENSG00000237054|transcript|ENST00000424245|antisense||n.-4025C>T|||||4025|,T|upstream_gene_variant|MODIFIER|RBM23|ENSG00000100461|transcript|ENST00000555691|protein_coding||c.-4038G>A|||||3639|WARNING_TRANSCRIPT_INCOMPLETE,T|upstream_gene_variant|MODIFIER|PRMT5-AS1|ENSG00000237054|transcript|ENST00000609885|antisense||n.-4190C>T|||||4190|,T|downstream_gene_variant|MODIFIER|RBM23|ENSG00000100461|transcript|ENST00000553777|processed_transcript||n.*2133G>A|||||2133|,T|intron_variant|MODIFIER|RBM23|ENSG00000100461|transcript|ENST00000359890|protein_coding|1/13|c.-11+3568G>A||||||,T|intron_variant|MODIFIER|RBM23|ENSG00000100461|transcript|ENST00000555209|protein_coding|1/10|c.-493+3568G>A||||||,T|intron_variant|MODIFIER|RBM23|ENSG00000100461|transcript|ENST00000557667|retained_intron|1/13|n.138+3568G>A||||||,T|intron_variant|MODIFIER|RBM23|ENSG00000100461|transcript|ENST00000399922|protein_coding|1/12|c.-11+3568G>A||||||,T|intron_variant|MODIFIER|RBM23|ENSG00000100461|transcript|ENST00000346528|protein_coding|1/11|c.-11+3568G>A||||||,T|intron_variant|MODIFIER|RBM23|ENSG00000100461|transcript|ENST00000542016|protein_coding|1/11|c.-360+3568G>A||||||,T|intron_variant|MODIFIER|RBM23|ENSG00000100461|transcript|ENST00000307814|retained_intron|1/10|n.151+3568G>A||||||,T|intron_variant|MODIFIER|RBM23|ENSG00000100461|transcript|ENST00000557403|protein_coding|1/5|c.-343+3568G>A||||||WARNING_TRANSCRIPT_INCOMPLETE,T|intron_variant|MODIFIER|RBM23|ENSG00000100461|transcript|ENST00000555722|protein_coding|1/4|c.-284+3568G>A||||||WARNING_TRANSCRIPT_INCOMPLETE,T|intron_variant|MODIFIER|RBM23|ENSG00000100461|transcript|ENST00000553920|processed_transcript|1/4|n.138+3568G>A||||||,T|intron_variant|MODIFIER|RBM23|ENSG00000100461|transcript|ENST00000557227|protein_coding|1/3|c.-306+3568G>A||||||WARNING_TRANSCRIPT_NO_STOP_CODON,T|intron_variant|MODIFIER|RBM23|ENSG00000100461|transcript|ENST00000555714|processed_transcript|1/5|n.151+3568G>A||||||,T|intron_variant|MODIFIER|RBM23|ENSG00000100461|transcript|ENST00000554256|protein_coding|1/5|c.-11+3568G>A||||||WARNING_TRANSCRIPT_NO_STOP_CODON,T|intron_variant|MODIFIER|RBM23|ENSG00000100461|transcript|ENST00000556687|retained_intron|1/3|n.151+3568G>A||||||,T|intron_variant|MODIFIER|RBM23|ENSG00000100461|transcript|ENST00000553902|retained_intron|1/2|n.151+3568G>A||||||,T|intron_variant|MODIFIER|RBM23|ENSG00000100461|transcript|ENST00000554955|retained_intron|1/4|n.112+3568G>A||||||,T|intron_variant|MODIFIER|RBM23|ENSG00000100461|transcript|ENST00000556984|processed_transcript|1/4|n.151+3568G>A||||||,T|intron_variant|MODIFIER|RBM23|ENSG00000100461|transcript|ENST00000557549|protein_coding|1/4|c.-128-3528G>A||||||WARNING_TRANSCRIPT_NO_STOP_CODON,T|intron_variant|MODIFIER|RBM23|ENSG00000100461|transcript|ENST00000555676|protein_coding|1/4|c.-132-1586G>A||||||WARNING_TRANSCRIPT_NO_STOP_CODON,T|intron_variant|MODIFIER|RBM23|ENSG00000100461|transcript|ENST00000557571|protein_coding|2/4|c.-11+1597G>A||||||WARNING_TRANSCRIPT_NO_STOP_CODON,T|intron_variant|MODIFIER|RBM23|ENSG00000100461|transcript|ENST00000556862|protein_coding|1/5|c.-132-1586G>A||||||WARNING_TRANSCRIPT_INCOMPLETE,T|intron_variant|MODIFIER|RBM23|ENSG00000100461|transcript|ENST00000557464|protein_coding|1/4|c.-108-1610G>A||||||WARNING_TRANSCRIPT_NO_STOP_CODON,T|intron_variant|MODIFIER|RBM23|ENSG00000100461|transcript|ENST00000554618|protein_coding|1/5|c.-210-1415G>A||||||WARNING_TRANSCRIPT_INCOMPLETE,T|intron_variant|MODIFIER|RBM23|ENSG00000100461|transcript|ENST00000556365|processed_transcript|1/3|n.151+3568G>A||||||,T|intron_variant|MODIFIER|RBM23|ENSG00000100461|transcript|ENST00000553876|protein_coding|1/2|c.-11+2048G>A||||||WARNING_TRANSCRIPT_NO_STOP_CODON |
| RBM23 | rs115742584 | 37 | 14 | 23389444 | 23389444 | + | SNP | A | A | G | SRR8586409 | Missense_Mutation | 0/1:276,48:324:99:1167,0,12095 | 324;ANN=G|upstream_gene_variant|MODIFIER|RBM23|ENSG00000100461|transcript|ENST00000359890|protein_coding||c.-8842T>C|||||1051|,G|upstream_gene_variant|MODIFIER|RBM23|ENSG00000100461|transcript|ENST00000555209|protein_coding||c.-15247T>C|||||1106|,G|upstream_gene_variant|MODIFIER|RBM23|ENSG00000100461|transcript|ENST00000557667|retained_intron||n.-1099T>C|||||1099|,G|upstream_gene_variant|MODIFIER|RBM23|ENSG00000100461|transcript|ENST00000399922|protein_coding||c.-8842T>C|||||1053|,G|upstream_gene_variant|MODIFIER|RBM23|ENSG00000100461|transcript|ENST00000346528|protein_coding||c.-8842T>C|||||1057|,G|upstream_gene_variant|MODIFIER|RBM23|ENSG00000100461|transcript|ENST00000542016|protein_coding||c.-14837T>C|||||1057|,G|upstream_gene_variant|MODIFIER|RBM23|ENSG00000100461|transcript|ENST00000307814|retained_intron||n.-1086T>C|||||1086|,G|upstream_gene_variant|MODIFIER|RBM23|ENSG00000100461|transcript|ENST00000557403|protein_coding||c.-14837T>C|||||1097|WARNING_TRANSCRIPT_INCOMPLETE,G|upstream_gene_variant|MODIFIER|RBM23|ENSG00000100461|transcript|ENST00000555722|protein_coding||c.-14837T>C|||||1070|WARNING_TRANSCRIPT_INCOMPLETE,G|upstream_gene_variant|MODIFIER|RBM23|ENSG00000100461|transcript|ENST00000553920|processed_transcript||n.-1099T>C|||||1099|,G|upstream_gene_variant|MODIFIER|RBM23|ENSG00000100461|transcript|ENST00000557227|protein_coding||c.-14837T>C|||||1053|WARNING_TRANSCRIPT_NO_STOP_CODON,G|upstream_gene_variant|MODIFIER|RBM23|ENSG00000100461|transcript|ENST00000555714|processed_transcript||n.-1086T>C|||||1086|,G|upstream_gene_variant|MODIFIER|RBM23|ENSG00000100461|transcript|ENST00000554256|protein_coding||c.-8842T>C|||||1098|WARNING_TRANSCRIPT_NO_STOP_CODON,G|upstream_gene_variant|MODIFIER|RBM23|ENSG00000100461|transcript|ENST00000556687|retained_intron||n.-1086T>C|||||1086|,G|upstream_gene_variant|MODIFIER|RBM23|ENSG00000100461|transcript|ENST00000553902|retained_intron||n.-1086T>C|||||1086|,G|upstream_gene_variant|MODIFIER|RBM23|ENSG00000100461|transcript|ENST00000554955|retained_intron||n.-1125T>C|||||1125|,G|upstream_gene_variant|MODIFIER|RBM23|ENSG00000100461|transcript|ENST00000556984|processed_transcript||n.-1086T>C|||||1086|,G|upstream_gene_variant|MODIFIER|RBM23|ENSG00000100461|transcript|ENST00000557549|protein_coding||c.-8842T>C|||||1086|WARNING_TRANSCRIPT_NO_STOP_CODON,G|upstream_gene_variant|MODIFIER|RBM23|ENSG00000100461|transcript|ENST00000555676|protein_coding||c.-8842T>C|||||1086|WARNING_TRANSCRIPT_NO_STOP_CODON,G|upstream_gene_variant|MODIFIER|RBM23|ENSG00000100461|transcript|ENST00000557571|protein_coding||c.-8842T>C|||||1086|WARNING_TRANSCRIPT_NO_STOP_CODON,G|upstream_gene_variant|MODIFIER|RBM23|ENSG00000100461|transcript|ENST00000556862|protein_coding||c.-8842T>C|||||1078|WARNING_TRANSCRIPT_INCOMPLETE,G|upstream_gene_variant|MODIFIER|RBM23|ENSG00000100461|transcript|ENST00000557464|protein_coding||c.-8842T>C|||||1086|WARNING_TRANSCRIPT_NO_STOP_CODON,G|upstream_gene_variant|MODIFIER|RBM23|ENSG00000100461|transcript|ENST00000554618|protein_coding||c.-8842T>C|||||1086|WARNING_TRANSCRIPT_INCOMPLETE,G|upstream_gene_variant|MODIFIER|RBM23|ENSG00000100461|transcript|ENST00000556365|processed_transcript||n.-1086T>C|||||1086|,G|upstream_gene_variant|MODIFIER|RBM23|ENSG00000100461|transcript|ENST00000553876|protein_coding||c.-8842T>C|||||2679|WARNING_TRANSCRIPT_NO_STOP_CODON,G|upstream_gene_variant|MODIFIER|RBM23|ENSG00000100461|transcript|ENST00000553777|processed_transcript||n.-1148T>C|||||1148|,G|upstream_gene_variant|MODIFIER|PRMT5-AS1|ENSG00000237054|transcript|ENST00000457443|antisense||n.-404A>G|||||404|,G|upstream_gene_variant|MODIFIER|PRMT5-AS1|ENSG00000237054|transcript|ENST00000599580|antisense||n.-676A>G|||||676|,G|upstream_gene_variant|MODIFIER|PRMT5-AS1|ENSG00000237054|transcript|ENST00000595662|antisense||n.-803A>G|||||803|,G|upstream_gene_variant|MODIFIER|PRMT5-AS1|ENSG00000237054|transcript|ENST00000587245|antisense||n.-803A>G|||||803|,G|upstream_gene_variant|MODIFIER|PRMT5-AS1|ENSG00000237054|transcript|ENST00000590290|antisense||n.-806A>G|||||806|,G|downstream_gene_variant|MODIFIER|PRMT5|ENSG00000100462|transcript|ENST00000324366|protein_coding||c.*669T>C|||||276|,G|downstream_gene_variant|MODIFIER|PRMT5|ENSG00000100462|transcript|ENST00000397441|protein_coding||c.*669T>C|||||290|,G|downstream_gene_variant|MODIFIER|PRMT5|ENSG00000100462|transcript|ENST00000557443|protein_coding||c.*446T>C|||||294|WARNING_TRANSCRIPT_NO_START_CODON,G|downstream_gene_variant|MODIFIER|PRMT5|ENSG00000100462|transcript|ENST00000476175|processed_transcript||n.*294T>C|||||294|,G|downstream_gene_variant|MODIFIER|PRMT5|ENSG00000100462|transcript|ENST00000397440|protein_coding||c.*669T>C|||||294|,G|downstream_gene_variant|MODIFIER|PRMT5|ENSG00000100462|transcript|ENST00000216350|protein_coding||c.*669T>C|||||295|,G|downstream_gene_variant|MODIFIER|PRMT5|ENSG00000100462|transcript|ENST00000555454|protein_coding||c.*812T>C|||||575|WARNING_TRANSCRIPT_NO_START_CODON,G|downstream_gene_variant|MODIFIER|PRMT5|ENSG00000100462|transcript|ENST00000454731|protein_coding||c.*669T>C|||||590|WARNING_TRANSCRIPT_NO_START_CODON,G|downstream_gene_variant|MODIFIER|PRMT5|ENSG00000100462|transcript|ENST00000553915|nonsense_mediated_decay||c.*7361T>C|||||622|,G|downstream_gene_variant|MODIFIER|PRMT5|ENSG00000100462|transcript|ENST00000538452|protein_coding||c.*669T>C|||||622|,G|downstream_gene_variant|MODIFIER|PRMT5|ENSG00000100462|transcript|ENST00000553897|protein_coding||c.*669T>C|||||641|,G|downstream_gene_variant|MODIFIER|PRMT5|ENSG00000100462|transcript|ENST00000557758|retained_intron||n.*1897T>C|||||1897|,G|downstream_gene_variant|MODIFIER|PRMT5|ENSG00000100462|transcript|ENST00000553502|protein_coding||c.*3927T>C|||||3927|WARNING_TRANSCRIPT_NO_START_CODON,G|downstream_gene_variant|MODIFIER|PRMT5|ENSG00000100462|transcript|ENST00000555530|protein_coding||c.*4082T>C|||||4082|WARNING_TRANSCRIPT_NO_START_CODON,G|downstream_gene_variant|MODIFIER|PRMT5|ENSG00000100462|transcript|ENST00000556043|protein_coding||c.*4264T>C|||||4264|WARNING_TRANSCRIPT_NO_STOP_CODON,G|downstream_gene_variant|MODIFIER|PRMT5|ENSG00000100462|transcript|ENST00000553550|protein_coding||c.*4403T>C|||||4403|WARNING_TRANSCRIPT_NO_STOP_CODON,G|downstream_gene_variant|MODIFIER|PRMT5|ENSG00000100462|transcript|ENST00000554716|processed_transcript||n.*4428T>C|||||4428|,G|downstream_gene_variant|MODIFIER|PRMT5|ENSG00000100462|transcript|ENST00000553787|nonsense_mediated_decay||c.*7361T>C|||||4765|,G|intron_variant|MODIFIER|PRMT5-AS1|ENSG00000237054|transcript|ENST00000609885|antisense|1/1|n.578+37A>G||||||,G|non_coding_transcript_exon_variant|MODIFIER|PRMT5-AS1|ENSG00000237054|transcript|ENST00000424245|antisense|1/2|n.780A>G||||||WARNING_REF_DOES_NOT_MATCH_GENOME |
| RBM23 | rs28730771 | 37 | 14 | 23390401 | 23390401 | + | SNP | C | C | T | SRR8586409 | Missense_Mutation | 0/1:309,63:372:99:611,0,8289 | 373;ANN=T|upstream_gene_variant|MODIFIER|RBM23|ENSG00000100461|transcript|ENST00000359890|protein_coding||c.-9799G>A|||||2008|,T|upstream_gene_variant|MODIFIER|RBM23|ENSG00000100461|transcript|ENST00000555209|protein_coding||c.-16204G>A|||||2063|,T|upstream_gene_variant|MODIFIER|RBM23|ENSG00000100461|transcript|ENST00000557667|retained_intron||n.-2056G>A|||||2056|,T|upstream_gene_variant|MODIFIER|RBM23|ENSG00000100461|transcript|ENST00000399922|protein_coding||c.-9799G>A|||||2010|,T|upstream_gene_variant|MODIFIER|RBM23|ENSG00000100461|transcript|ENST00000346528|protein_coding||c.-9799G>A|||||2014|,T|upstream_gene_variant|MODIFIER|RBM23|ENSG00000100461|transcript|ENST00000542016|protein_coding||c.-15794G>A|||||2014|,T|upstream_gene_variant|MODIFIER|RBM23|ENSG00000100461|transcript|ENST00000307814|retained_intron||n.-2043G>A|||||2043|,T|upstream_gene_variant|MODIFIER|RBM23|ENSG00000100461|transcript|ENST00000557403|protein_coding||c.-15794G>A|||||2054|WARNING_TRANSCRIPT_INCOMPLETE,T|upstream_gene_variant|MODIFIER|RBM23|ENSG00000100461|transcript|ENST00000555722|protein_coding||c.-15794G>A|||||2027|WARNING_TRANSCRIPT_INCOMPLETE,T|upstream_gene_variant|MODIFIER|RBM23|ENSG00000100461|transcript|ENST00000553920|processed_transcript||n.-2056G>A|||||2056|,T|upstream_gene_variant|MODIFIER|RBM23|ENSG00000100461|transcript|ENST00000557227|protein_coding||c.-15794G>A|||||2010|WARNING_TRANSCRIPT_NO_STOP_CODON,T|upstream_gene_variant|MODIFIER|RBM23|ENSG00000100461|transcript|ENST00000555714|processed_transcript||n.-2043G>A|||||2043|,T|upstream_gene_variant|MODIFIER|RBM23|ENSG00000100461|transcript|ENST00000554256|protein_coding||c.-9799G>A|||||2055|WARNING_TRANSCRIPT_NO_STOP_CODON,T|upstream_gene_variant|MODIFIER|RBM23|ENSG00000100461|transcript|ENST00000556687|retained_intron||n.-2043G>A|||||2043|,T|upstream_gene_variant|MODIFIER|RBM23|ENSG00000100461|transcript|ENST00000553902|retained_intron||n.-2043G>A|||||2043|,T|upstream_gene_variant|MODIFIER|RBM23|ENSG00000100461|transcript|ENST00000554955|retained_intron||n.-2082G>A|||||2082|,T|upstream_gene_variant|MODIFIER|RBM23|ENSG00000100461|transcript|ENST00000556984|processed_transcript||n.-2043G>A|||||2043|,T|upstream_gene_variant|MODIFIER|RBM23|ENSG00000100461|transcript|ENST00000557549|protein_coding||c.-9799G>A|||||2043|WARNING_TRANSCRIPT_NO_STOP_CODON,T|upstream_gene_variant|MODIFIER|RBM23|ENSG00000100461|transcript|ENST00000555676|protein_coding||c.-9799G>A|||||2043|WARNING_TRANSCRIPT_NO_STOP_CODON,T|upstream_gene_variant|MODIFIER|RBM23|ENSG00000100461|transcript|ENST00000557571|protein_coding||c.-9799G>A|||||2043|WARNING_TRANSCRIPT_NO_STOP_CODON,T|upstream_gene_variant|MODIFIER|RBM23|ENSG00000100461|transcript|ENST00000556862|protein_coding||c.-9799G>A|||||2035|WARNING_TRANSCRIPT_INCOMPLETE,T|upstream_gene_variant|MODIFIER|RBM23|ENSG00000100461|transcript|ENST00000557464|protein_coding||c.-9799G>A|||||2043|WARNING_TRANSCRIPT_NO_STOP_CODON,T|upstream_gene_variant|MODIFIER|RBM23|ENSG00000100461|transcript|ENST00000554618|protein_coding||c.-9799G>A|||||2043|WARNING_TRANSCRIPT_INCOMPLETE,T|upstream_gene_variant|MODIFIER|RBM23|ENSG00000100461|transcript|ENST00000556365|processed_transcript||n.-2043G>A|||||2043|,T|upstream_gene_variant|MODIFIER|RBM23|ENSG00000100461|transcript|ENST00000553876|protein_coding||c.-9799G>A|||||3636|WARNING_TRANSCRIPT_NO_STOP_CODON,T|upstream_gene_variant|MODIFIER|RBM23|ENSG00000100461|transcript|ENST00000553777|processed_transcript||n.-2105G>A|||||2105|,T|downstream_gene_variant|MODIFIER|PRMT5|ENSG00000100462|transcript|ENST00000557758|retained_intron||n.*940G>A|||||940|,T|downstream_gene_variant|MODIFIER|PRMT5|ENSG00000100462|transcript|ENST00000553502|protein_coding||c.*2970G>A|||||2970|WARNING_TRANSCRIPT_NO_START_CODON,T|downstream_gene_variant|MODIFIER|PRMT5|ENSG00000100462|transcript|ENST00000555530|protein_coding||c.*3125G>A|||||3125|WARNING_TRANSCRIPT_NO_START_CODON,T|downstream_gene_variant|MODIFIER|PRMT5|ENSG00000100462|transcript|ENST00000556043|protein_coding||c.*3307G>A|||||3307|WARNING_TRANSCRIPT_NO_STOP_CODON,T|downstream_gene_variant|MODIFIER|PRMT5|ENSG00000100462|transcript|ENST00000553550|protein_coding||c.*3446G>A|||||3446|WARNING_TRANSCRIPT_NO_STOP_CODON,T|downstream_gene_variant|MODIFIER|PRMT5|ENSG00000100462|transcript|ENST00000554716|processed_transcript||n.*3471G>A|||||3471|,T|downstream_gene_variant|MODIFIER|PRMT5|ENSG00000100462|transcript|ENST00000553787|nonsense_mediated_decay||c.*6404G>A|||||3808|,T|downstream_gene_variant|MODIFIER|PRMT5|ENSG00000100462|transcript|ENST00000553641|processed_transcript||n.*4941G>A|||||4941|,T|intron_variant|MODIFIER|PRMT5-AS1|ENSG00000237054|transcript|ENST00000424245|antisense|1/1|n.1665+72C>T||||||,T|intron_variant|MODIFIER|PRMT5|ENSG00000100462|transcript|ENST00000324366|protein_coding|16/16|c.1762-136G>A||||||,T|intron_variant|MODIFIER|PRMT5-AS1|ENSG00000237054|transcript|ENST00000609885|antisense|1/1|n.578+994C>T||||||,T|intron_variant|MODIFIER|PRMT5|ENSG00000100462|transcript|ENST00000397441|protein_coding|16/16|c.1711-136G>A||||||,T|intron_variant|MODIFIER|PRMT5|ENSG00000100462|transcript|ENST00000557443|protein_coding|1/2|c.64-136G>A||||||WARNING_TRANSCRIPT_NO_START_CODON,T|intron_variant|MODIFIER|PRMT5|ENSG00000100462|transcript|ENST00000476175|processed_transcript|3/4|n.263-136G>A||||||,T|intron_variant|MODIFIER|PRMT5|ENSG00000100462|transcript|ENST00000397440|protein_coding|12/12|c.1249-136G>A||||||,T|intron_variant|MODIFIER|PRMT5|ENSG00000100462|transcript|ENST00000216350|protein_coding|15/15|c.1579-136G>A||||||,T|intron_variant|MODIFIER|PRMT5-AS1|ENSG00000237054|transcript|ENST00000457443|antisense|1/1|n.482+72C>T||||||,T|intron_variant|MODIFIER|PRMT5|ENSG00000100462|transcript|ENST00000555454|protein_coding|5/5|c.525-136G>A||||||WARNING_TRANSCRIPT_NO_START_CODON,T|intron_variant|MODIFIER|PRMT5|ENSG00000100462|transcript|ENST00000454731|protein_coding|4/4|c.388-139G>A||||||WARNING_TRANSCRIPT_NO_START_CODON,T|intron_variant|MODIFIER|PRMT5|ENSG00000100462|transcript|ENST00000553915|nonsense_mediated_decay|15/15|c.*1382-136G>A||||||,T|intron_variant|MODIFIER|PRMT5|ENSG00000100462|transcript|ENST00000538452|protein_coding|15/15|c.1444-136G>A||||||,T|intron_variant|MODIFIER|PRMT5|ENSG00000100462|transcript|ENST00000553897|protein_coding|15/15|c.1630-136G>A||||||,T|intron_variant|MODIFIER|PRMT5-AS1|ENSG00000237054|transcript|ENST00000599580|antisense|1/3|n.210+72C>T||||||,T|intron_variant|MODIFIER|PRMT5-AS1|ENSG00000237054|transcript|ENST00000595662|antisense|1/3|n.83+72C>T||||||,T|intron_variant|MODIFIER|PRMT5-AS1|ENSG00000237054|transcript|ENST00000587245|antisense|1/2|n.83+72C>T||||||,T|intron_variant|MODIFIER|PRMT5-AS1|ENSG00000237054|transcript|ENST00000590290|antisense|1/2|n.80+72C>T|||||| |
| PRMT5 | rs763963623 | 37 | 14 | 23396797 | 23396797 | + | SNP | A | A | G | SRR8586409 | Missense_Mutation | 0/1:285,436:721:99:10371,0,7348 | 722;ANN=G|missense_variant|MODERATE|PRMT5|ENSG00000100462|transcript|ENST00000324366|protein_coding|4/17|c.388T>C|p.Asp130His|612/2531|388/1914|130/637||WARNING_REF_DOES_NOT_MATCH_GENOME,G|missense_variant|MODERATE|PRMT5|ENSG00000100462|transcript|ENST00000397441|protein_coding|4/17|c.337T>C|p.Asp113His|513/2418|337/1863|113/620||WARNING_REF_DOES_NOT_MATCH_GENOME,G|missense_variant|MODERATE|PRMT5|ENSG00000100462|transcript|ENST00000216350|protein_coding|3/16|c.205T>C|p.Asp69His|371/2271|205/1731|69/576||WARNING_REF_DOES_NOT_MATCH_GENOME,G|missense_variant|MODERATE|PRMT5|ENSG00000100462|transcript|ENST00000538452|protein_coding|3/16|c.70T>C|p.Asp24His|360/1933|70/1596|24/531||WARNING_REF_DOES_NOT_MATCH_GENOME,G|missense_variant|MODERATE|PRMT5|ENSG00000100462|transcript|ENST00000553897|protein_coding|3/16|c.256T>C|p.Asp86His|303/1857|256/1782|86/593||WARNING_REF_DOES_NOT_MATCH_GENOME,G|missense_variant|MODERATE|PRMT5|ENSG00000100462|transcript|ENST00000555530|protein_coding|2/9|c.91T>C|p.Asp31His|91/837|91/837|31/278||WARNING_TRANSCRIPT_NO_START_CODON&WARNING_REF_DOES_NOT_MATCH_GENOME,G|missense_variant|MODERATE|PRMT5|ENSG00000100462|transcript|ENST00000556616|protein_coding|3/6|c.274T>C|p.Asp92His|289/571|274/556|92/184||WARNING_TRANSCRIPT_INCOMPLETE&WARNING_REF_DOES_NOT_MATCH_GENOME,G|missense_variant|MODERATE|PRMT5|ENSG00000100462|transcript|ENST00000554910|protein_coding|4/7|c.262T>C|p.Asp88His|330/577|262/509|88/168||WARNING_TRANSCRIPT_INCOMPLETE&WARNING_REF_DOES_NOT_MATCH_GENOME,G|missense_variant|MODERATE|PRMT5|ENSG00000100462|transcript|ENST00000421938|protein_coding|4/5|c.418T>C|p.Asp140His|425/565|418/558|140/185||WARNING_TRANSCRIPT_NO_STOP_CODON&WARNING_REF_DOES_NOT_MATCH_GENOME,G|3_prime_UTR_variant|MODIFIER|PRMT5|ENSG00000100462|transcript|ENST00000553915|nonsense_mediated_decay|3/16|c.*8T>C|||||8|WARNING_REF_DOES_NOT_MATCH_GENOME,G|3_prime_UTR_variant|MODIFIER|PRMT5|ENSG00000100462|transcript|ENST00000553787|nonsense_mediated_decay|2/6|c.*8T>C|||||8|WARNING_REF_DOES_NOT_MATCH_GENOME,G|3_prime_UTR_variant|MODIFIER|PRMT5|ENSG00000100462|transcript|ENST00000557415|nonsense_mediated_decay|3/6|c.*8T>C|||||8|WARNING_REF_DOES_NOT_MATCH_GENOME,G|upstream_gene_variant|MODIFIER|RP11-298I3.1|ENSG00000257285|transcript|ENST00000548322|antisense||n.-2057A>G|||||2057|,G|upstream_gene_variant|MODIFIER|PRMT5|ENSG00000100462|transcript|ENST00000476175|processed_transcript||n.-4767T>C|||||4767|,G|upstream_gene_variant|MODIFIER|PRMT5|ENSG00000100462|transcript|ENST00000555454|protein_coding||c.-3409T>C|||||3409|WARNING_TRANSCRIPT_NO_START_CODON,G|upstream_gene_variant|MODIFIER|PRMT5|ENSG00000100462|transcript|ENST00000454731|protein_coding||c.-4427T>C|||||4426|WARNING_TRANSCRIPT_NO_START_CODON,G|upstream_gene_variant|MODIFIER|PRMT5|ENSG00000100462|transcript|ENST00000557758|retained_intron||n.-4678T>C|||||4678|,G|upstream_gene_variant|MODIFIER|PRMT5|ENSG00000100462|transcript|ENST00000553502|protein_coding||c.-2542T>C|||||2542|WARNING_TRANSCRIPT_NO_START_CODON,G|upstream_gene_variant|MODIFIER|PRMT5|ENSG00000100462|transcript|ENST00000556043|protein_coding||c.-1423T>C|||||1132|WARNING_TRANSCRIPT_NO_STOP_CODON,G|upstream_gene_variant|MODIFIER|RP11-298I3.1|ENSG00000257285|transcript|ENST00000548819|antisense||n.-2021A>G|||||2021|,G|downstream_gene_variant|MODIFIER|PRMT5-AS1|ENSG00000237054|transcript|ENST00000424245|antisense||n.*4181A>G|||||4181|,G|downstream_gene_variant|MODIFIER|PRMT5-AS1|ENSG00000237054|transcript|ENST00000609885|antisense||n.*4337A>G|||||4337|,G|downstream_gene_variant|MODIFIER|PRMT5-AS1|ENSG00000237054|transcript|ENST00000457443|antisense||n.*4185A>G|||||4185|,G|downstream_gene_variant|MODIFIER|PRMT5-AS1|ENSG00000237054|transcript|ENST00000599580|antisense||n.*758A>G|||||758|,G|downstream_gene_variant|MODIFIER|PRMT5-AS1|ENSG00000237054|transcript|ENST00000595662|antisense||n.*969A>G|||||969|,G|downstream_gene_variant|MODIFIER|PRMT5-AS1|ENSG00000237054|transcript|ENST00000587245|antisense||n.*763A>G|||||763|,G|downstream_gene_variant|MODIFIER|PRMT5-AS1|ENSG00000237054|transcript|ENST00000590290|antisense||n.*692A>G|||||692|,G|downstream_gene_variant|MODIFIER|PRMT5|ENSG00000100462|transcript|ENST00000557015|retained_intron||n.*543T>C|||||543|,G|downstream_gene_variant|MODIFIER|PRMT5|ENSG00000100462|transcript|ENST00000556426|processed_transcript||n.*675T>C|||||675|,G|intron_variant|MODIFIER|PRMT5|ENSG00000100462|transcript|ENST00000397440|protein_coding|3/12|c.264+538T>C||||||,G|intron_variant|MODIFIER|PRMT5|ENSG00000100462|transcript|ENST00000553550|protein_coding|3/4|c.315+538T>C||||||WARNING_TRANSCRIPT_NO_STOP_CODON,G|intron_variant|MODIFIER|PRMT5|ENSG00000100462|transcript|ENST00000554716|processed_transcript|3/4|n.352+538T>C||||||,G|intron_variant|MODIFIER|PRMT5|ENSG00000100462|transcript|ENST00000554867|protein_coding|3/5|c.315+538T>C||||||WARNING_TRANSCRIPT_NO_STOP_CODON,G|non_coding_transcript_exon_variant|MODIFIER|PRMT5|ENSG00000100462|transcript|ENST00000553641|processed_transcript|4/7|n.411T>C||||||WARNING_REF_DOES_NOT_MATCH_GENOME,G|non_coding_transcript_exon_variant|MODIFIER|PRMT5|ENSG00000100462|transcript|ENST00000553417|retained_intron|1/3|n.9T>C||||||WARNING_REF_DOES_NOT_MATCH_GENOME,G|non_coding_transcript_exon_variant|MODIFIER|PRMT5|ENSG00000100462|transcript|ENST00000556032|retained_intron|3/3|n.581T>C||||||WARNING_REF_DOES_NOT_MATCH_GENOME |
| RP11-298I3.1 | rs765316931 | 37 | 14 | 23397004 | 23397004 | + | SNP | C | C | T | SRR8586409 | Missense_Mutation | 0/1:197,398:595:99:12058,0,4737 | 596;ANN=T|upstream_gene_variant|MODIFIER|RP11-298I3.1|ENSG00000257285|transcript|ENST00000548322|antisense||n.-1850C>T|||||1850|,T|upstream_gene_variant|MODIFIER|PRMT5|ENSG00000100462|transcript|ENST00000476175|processed_transcript||n.-4974G>A|||||4974|,T|upstream_gene_variant|MODIFIER|PRMT5|ENSG00000100462|transcript|ENST00000555454|protein_coding||c.-3616G>A|||||3616|WARNING_TRANSCRIPT_NO_START_CODON,T|upstream_gene_variant|MODIFIER|PRMT5|ENSG00000100462|transcript|ENST00000454731|protein_coding||c.-4634G>A|||||4633|WARNING_TRANSCRIPT_NO_START_CODON,T|upstream_gene_variant|MODIFIER|PRMT5|ENSG00000100462|transcript|ENST00000557758|retained_intron||n.-4885G>A|||||4885|,T|upstream_gene_variant|MODIFIER|PRMT5|ENSG00000100462|transcript|ENST00000553502|protein_coding||c.-2749G>A|||||2749|WARNING_TRANSCRIPT_NO_START_CODON,T|upstream_gene_variant|MODIFIER|PRMT5|ENSG00000100462|transcript|ENST00000556043|protein_coding||c.-1630G>A|||||1339|WARNING_TRANSCRIPT_NO_STOP_CODON,T|upstream_gene_variant|MODIFIER|PRMT5|ENSG00000100462|transcript|ENST00000553417|retained_intron||n.-199G>A|||||199|,T|upstream_gene_variant|MODIFIER|RP11-298I3.1|ENSG00000257285|transcript|ENST00000548819|antisense||n.-1814C>T|||||1814|,T|downstream_gene_variant|MODIFIER|PRMT5-AS1|ENSG00000237054|transcript|ENST00000424245|antisense||n.*4388C>T|||||4388|,T|downstream_gene_variant|MODIFIER|PRMT5-AS1|ENSG00000237054|transcript|ENST00000609885|antisense||n.*4544C>T|||||4544|,T|downstream_gene_variant|MODIFIER|PRMT5-AS1|ENSG00000237054|transcript|ENST00000457443|antisense||n.*4392C>T|||||4392|,T|downstream_gene_variant|MODIFIER|PRMT5-AS1|ENSG00000237054|transcript|ENST00000599580|antisense||n.*965C>T|||||965|,T|downstream_gene_variant|MODIFIER|PRMT5-AS1|ENSG00000237054|transcript|ENST00000595662|antisense||n.*1176C>T|||||1176|,T|downstream_gene_variant|MODIFIER|PRMT5-AS1|ENSG00000237054|transcript|ENST00000587245|antisense||n.*970C>T|||||970|,T|downstream_gene_variant|MODIFIER|PRMT5-AS1|ENSG00000237054|transcript|ENST00000590290|antisense||n.*899C>T|||||899|,T|downstream_gene_variant|MODIFIER|PRMT5|ENSG00000100462|transcript|ENST00000557015|retained_intron||n.*336G>A|||||336|,T|downstream_gene_variant|MODIFIER|PRMT5|ENSG00000100462|transcript|ENST00000556426|processed_transcript||n.*468G>A|||||468|,T|intron_variant|MODIFIER|PRMT5|ENSG00000100462|transcript|ENST00000324366|protein_coding|3/16|c.316-135G>A||||||,T|intron_variant|MODIFIER|PRMT5|ENSG00000100462|transcript|ENST00000397441|protein_coding|3/16|c.265-135G>A||||||,T|intron_variant|MODIFIER|PRMT5|ENSG00000100462|transcript|ENST00000397440|protein_coding|3/12|c.264+331G>A||||||,T|intron_variant|MODIFIER|PRMT5|ENSG00000100462|transcript|ENST00000216350|protein_coding|2/15|c.179-181G>A||||||,T|intron_variant|MODIFIER|PRMT5|ENSG00000100462|transcript|ENST00000553915|nonsense_mediated_decay|2/15|c.146-135G>A||||||,T|intron_variant|MODIFIER|PRMT5|ENSG00000100462|transcript|ENST00000538452|protein_coding|2/15|c.-3-135G>A||||||,T|intron_variant|MODIFIER|PRMT5|ENSG00000100462|transcript|ENST00000553897|protein_coding|2/15|c.230-181G>A||||||,T|intron_variant|MODIFIER|PRMT5|ENSG00000100462|transcript|ENST00000555530|protein_coding|1/8|c.19-135G>A||||||WARNING_TRANSCRIPT_NO_START_CODON,T|intron_variant|MODIFIER|PRMT5|ENSG00000100462|transcript|ENST00000553550|protein_coding|3/4|c.315+331G>A||||||WARNING_TRANSCRIPT_NO_STOP_CODON,T|intron_variant|MODIFIER|PRMT5|ENSG00000100462|transcript|ENST00000554716|processed_transcript|3/4|n.352+331G>A||||||,T|intron_variant|MODIFIER|PRMT5|ENSG00000100462|transcript|ENST00000553787|nonsense_mediated_decay|1/5|c.111-181G>A||||||,T|intron_variant|MODIFIER|PRMT5|ENSG00000100462|transcript|ENST00000553641|processed_transcript|3/6|n.339-135G>A||||||,T|intron_variant|MODIFIER|PRMT5|ENSG00000100462|transcript|ENST00000554867|protein_coding|3/5|c.315+331G>A||||||WARNING_TRANSCRIPT_NO_STOP_CODON,T|intron_variant|MODIFIER|PRMT5|ENSG00000100462|transcript|ENST00000557415|nonsense_mediated_decay|2/5|c.197-135G>A||||||,T|intron_variant|MODIFIER|PRMT5|ENSG00000100462|transcript|ENST00000556616|protein_coding|2/5|c.202-135G>A||||||WARNING_TRANSCRIPT_INCOMPLETE,T|intron_variant|MODIFIER|PRMT5|ENSG00000100462|transcript|ENST00000554910|protein_coding|3/6|c.190-135G>A||||||WARNING_TRANSCRIPT_INCOMPLETE,T|intron_variant|MODIFIER|PRMT5|ENSG00000100462|transcript|ENST00000421938|protein_coding|3/4|c.346-135G>A||||||WARNING_TRANSCRIPT_NO_STOP_CODON,T|intron_variant|MODIFIER|PRMT5|ENSG00000100462|transcript|ENST00000556032|retained_intron|2/2|n.509-135G>A|||||| |
| RP11-298I3.1 | rs149734381 | 37 | 14 | 23397034 | 23397034 | + | SNP | G | G | A | SRR8586409 | Missense_Mutation | 0/1:156,367:523:99:11355,0,3234 | 524;ANN=A|upstream_gene_variant|MODIFIER|RP11-298I3.1|ENSG00000257285|transcript|ENST00000548322|antisense||n.-1820G>A|||||1820|,A|upstream_gene_variant|MODIFIER|PRMT5|ENSG00000100462|transcript|ENST00000555454|protein_coding||c.-3646C>T|||||3646|WARNING_TRANSCRIPT_NO_START_CODON,A|upstream_gene_variant|MODIFIER|PRMT5|ENSG00000100462|transcript|ENST00000454731|protein_coding||c.-4664C>T|||||4663|WARNING_TRANSCRIPT_NO_START_CODON,A|upstream_gene_variant|MODIFIER|PRMT5|ENSG00000100462|transcript|ENST00000557758|retained_intron||n.-4915C>T|||||4915|,A|upstream_gene_variant|MODIFIER|PRMT5|ENSG00000100462|transcript|ENST00000553502|protein_coding||c.-2779C>T|||||2779|WARNING_TRANSCRIPT_NO_START_CODON,A|upstream_gene_variant|MODIFIER|PRMT5|ENSG00000100462|transcript|ENST00000556043|protein_coding||c.-1660C>T|||||1369|WARNING_TRANSCRIPT_NO_STOP_CODON,A|upstream_gene_variant|MODIFIER|PRMT5|ENSG00000100462|transcript|ENST00000553417|retained_intron||n.-229C>T|||||229|,A|upstream_gene_variant|MODIFIER|RP11-298I3.1|ENSG00000257285|transcript|ENST00000548819|antisense||n.-1784G>A|||||1784|,A|downstream_gene_variant|MODIFIER|PRMT5-AS1|ENSG00000237054|transcript|ENST00000424245|antisense||n.*4418G>A|||||4418|,A|downstream_gene_variant|MODIFIER|PRMT5-AS1|ENSG00000237054|transcript|ENST00000609885|antisense||n.*4574G>A|||||4574|,A|downstream_gene_variant|MODIFIER|PRMT5-AS1|ENSG00000237054|transcript|ENST00000457443|antisense||n.*4422G>A|||||4422|,A|downstream_gene_variant|MODIFIER|PRMT5-AS1|ENSG00000237054|transcript|ENST00000599580|antisense||n.*995G>A|||||995|,A|downstream_gene_variant|MODIFIER|PRMT5-AS1|ENSG00000237054|transcript|ENST00000595662|antisense||n.*1206G>A|||||1206|,A|downstream_gene_variant|MODIFIER|PRMT5-AS1|ENSG00000237054|transcript|ENST00000587245|antisense||n.*1000G>A|||||1000|,A|downstream_gene_variant|MODIFIER|PRMT5-AS1|ENSG00000237054|transcript|ENST00000590290|antisense||n.*929G>A|||||929|,A|downstream_gene_variant|MODIFIER|PRMT5|ENSG00000100462|transcript|ENST00000557015|retained_intron||n.*306C>T|||||306|,A|downstream_gene_variant|MODIFIER|PRMT5|ENSG00000100462|transcript|ENST00000556426|processed_transcript||n.*438C>T|||||438|,A|intron_variant|MODIFIER|PRMT5|ENSG00000100462|transcript|ENST00000324366|protein_coding|3/16|c.316-165C>T||||||,A|intron_variant|MODIFIER|PRMT5|ENSG00000100462|transcript|ENST00000397441|protein_coding|3/16|c.265-165C>T||||||,A|intron_variant|MODIFIER|PRMT5|ENSG00000100462|transcript|ENST00000397440|protein_coding|3/12|c.264+301C>T||||||,A|intron_variant|MODIFIER|PRMT5|ENSG00000100462|transcript|ENST00000216350|protein_coding|2/15|c.179-211C>T||||||,A|intron_variant|MODIFIER|PRMT5|ENSG00000100462|transcript|ENST00000553915|nonsense_mediated_decay|2/15|c.146-165C>T||||||,A|intron_variant|MODIFIER|PRMT5|ENSG00000100462|transcript|ENST00000538452|protein_coding|2/15|c.-3-165C>T||||||,A|intron_variant|MODIFIER|PRMT5|ENSG00000100462|transcript|ENST00000553897|protein_coding|2/15|c.230-211C>T||||||,A|intron_variant|MODIFIER|PRMT5|ENSG00000100462|transcript|ENST00000555530|protein_coding|1/8|c.19-165C>T||||||WARNING_TRANSCRIPT_NO_START_CODON,A|intron_variant|MODIFIER|PRMT5|ENSG00000100462|transcript|ENST00000553550|protein_coding|3/4|c.315+301C>T||||||WARNING_TRANSCRIPT_NO_STOP_CODON,A|intron_variant|MODIFIER|PRMT5|ENSG00000100462|transcript|ENST00000554716|processed_transcript|3/4|n.352+301C>T||||||,A|intron_variant|MODIFIER|PRMT5|ENSG00000100462|transcript|ENST00000553787|nonsense_mediated_decay|1/5|c.111-211C>T||||||,A|intron_variant|MODIFIER|PRMT5|ENSG00000100462|transcript|ENST00000553641|processed_transcript|3/6|n.339-165C>T||||||,A|intron_variant|MODIFIER|PRMT5|ENSG00000100462|transcript|ENST00000554867|protein_coding|3/5|c.315+301C>T||||||WARNING_TRANSCRIPT_NO_STOP_CODON,A|intron_variant|MODIFIER|PRMT5|ENSG00000100462|transcript|ENST00000557415|nonsense_mediated_decay|2/5|c.197-165C>T||||||,A|intron_variant|MODIFIER|PRMT5|ENSG00000100462|transcript|ENST00000556616|protein_coding|2/5|c.202-165C>T||||||WARNING_TRANSCRIPT_INCOMPLETE,A|intron_variant|MODIFIER|PRMT5|ENSG00000100462|transcript|ENST00000554910|protein_coding|3/6|c.190-165C>T||||||WARNING_TRANSCRIPT_INCOMPLETE,A|intron_variant|MODIFIER|PRMT5|ENSG00000100462|transcript|ENST00000421938|protein_coding|3/4|c.346-165C>T||||||WARNING_TRANSCRIPT_NO_STOP_CODON,A|intron_variant|MODIFIER|PRMT5|ENSG00000100462|transcript|ENST00000556032|retained_intron|2/2|n.509-165C>T|||||| |
| HAUS4 | rs730880754 | 37 | 14 | 23424131 | 23424131 | + | SNP | C | C | T | SRR8586409 | Missense_Mutation | 0/1:1038,328:1366:99:8176,0,34062 | 1412;ANN=T|upstream_gene_variant|MODIFIER|HAUS4|ENSG00000092036|transcript|ENST00000553859|nonsense_mediated_decay||c.-2517G>A|||||2517|WARNING_TRANSCRIPT_NO_START_CODON,T|upstream_gene_variant|MODIFIER|HAUS4|ENSG00000092036|transcript|ENST00000553420|protein_coding||c.-2241G>A|||||2239|WARNING_TRANSCRIPT_NO_START_CODON,T|downstream_gene_variant|MODIFIER|MIR4707|ENSG00000265037|transcript|ENST00000579686|miRNA||n.*2028G>A|||||2028|,T|downstream_gene_variant|MODIFIER|RP11-298I3.1|ENSG00000257285|transcript|ENST00000548819|antisense||n.*230C>T|||||230|,T|intron_variant|MODIFIER|RP11-298I3.1|ENSG00000257285|transcript|ENST00000548322|antisense|2/2|n.159-208C>T||||||,T|intron_variant|MODIFIER|HAUS4|ENSG00000092036|transcript|ENST00000206474|protein_coding|2/9|c.55+178G>A||||||,T|intron_variant|MODIFIER|RP11-298I3.5|ENSG00000259132|transcript|ENST00000555074|protein_coding|1/4|c.50-6909G>A||||||WARNING_TRANSCRIPT_NO_START_CODON,T|intron_variant|MODIFIER|HAUS4|ENSG00000092036|transcript|ENST00000490506|protein_coding|2/8|c.-175+178G>A||||||,T|intron_variant|MODIFIER|HAUS4|ENSG00000092036|transcript|ENST00000554446|processed_transcript|1/6|n.77+178G>A||||||,T|intron_variant|MODIFIER|HAUS4|ENSG00000092036|transcript|ENST00000541587|protein_coding|2/9|c.55+178G>A||||||,T|intron_variant|MODIFIER|HAUS4|ENSG00000092036|transcript|ENST00000397409|protein_coding|2/6|c.55+178G>A||||||,T|intron_variant|MODIFIER|HAUS4|ENSG00000092036|transcript|ENST00000342454|protein_coding|2/8|c.55+178G>A||||||,T|intron_variant|MODIFIER|HAUS4|ENSG00000092036|transcript|ENST00000347758|protein_coding|2/6|c.55+178G>A||||||,T|intron_variant|MODIFIER|HAUS4|ENSG00000092036|transcript|ENST00000555367|protein_coding|2/8|c.55+178G>A||||||,T|intron_variant|MODIFIER|HAUS4|ENSG00000092036|transcript|ENST00000555986|protein_coding|2/8|c.55+178G>A||||||,T|intron_variant|MODIFIER|HAUS4|ENSG00000092036|transcript|ENST00000555040|protein_coding|2/7|c.55+178G>A||||||WARNING_TRANSCRIPT_INCOMPLETE,T|intron_variant|MODIFIER|HAUS4|ENSG00000092036|transcript|ENST00000556915|protein_coding|2/5|c.55+178G>A||||||WARNING_TRANSCRIPT_INCOMPLETE,T|intron_variant|MODIFIER|HAUS4|ENSG00000092036|transcript|ENST00000554349|processed_transcript|2/5|n.212+178G>A||||||,T|intron_variant|MODIFIER|HAUS4|ENSG00000092036|transcript|ENST00000553794|processed_transcript|2/2|n.423+178G>A||||||,T|intron_variant|MODIFIER|HAUS4|ENSG00000092036|transcript|ENST00000554373|processed_transcript|2/6|n.275+178G>A||||||,T|intron_variant|MODIFIER|HAUS4|ENSG00000092036|transcript|ENST00000554651|processed_transcript|2/6|n.235+178G>A||||||,T|intron_variant|MODIFIER|HAUS4|ENSG00000092036|transcript|ENST00000554516|protein_coding|2/5|c.55+178G>A||||||WARNING_TRANSCRIPT_INCOMPLETE,T|intron_variant|MODIFIER|HAUS4|ENSG00000092036|transcript|ENST00000557591|protein_coding|3/6|c.55+178G>A||||||WARNING_TRANSCRIPT_INCOMPLETE,T|intron_variant|MODIFIER|HAUS4|ENSG00000092036|transcript|ENST00000554406|protein_coding|2/3|c.-175+178G>A||||||WARNING_TRANSCRIPT_INCOMPLETE |
| GPHN | rs1980817 | 37 | 14 | 67385454 | 67385454 | + | SNP | A | A | C | SRR8586409 | Nonsense_Mutation | 0/1:27,54:81:99:2086,0,997 | 81;ANN=C|downstream_gene_variant|MODIFIER|GPHN|ENSG00000171723|transcript|ENST00000553936|nonsense_mediated_decay||c.*367640A>C|||||2703|,C|intron_variant|MODIFIER|GPHN|ENSG00000171723|transcript|ENST00000543237|protein_coding|7/24|c.495+2668A>C||||||,C|intron_variant|MODIFIER|GPHN|ENSG00000171723|transcript|ENST00000315266|protein_coding|6/21|c.456+2668A>C||||||,C|intron_variant|MODIFIER|GPHN|ENSG00000171723|transcript|ENST00000478722|protein_coding|6/22|c.456+2668A>C||||||,C|intron_variant|MODIFIER|GPHN|ENSG00000171723|transcript|ENST00000459628|protein_coding|6/10|c.402+2668A>C||||||,C|intron_variant|MODIFIER|GPHN|ENSG00000171723|transcript|ENST00000556633|nonsense_mediated_decay|5/5|c.*245+2668A>C||||||,C|intron_variant|MODIFIER|GPHN|ENSG00000171723|transcript|ENST00000305960|protein_coding|5/20|c.363+2668A>C||||||,C|intron_variant|MODIFIER|GPHN|ENSG00000171723|transcript|ENST00000544752|processed_transcript|4/20|n.504+2668A>C||||||,C|intron_variant|MODIFIER|GPHN|ENSG00000171723|transcript|ENST00000555456|protein_coding|3/7|c.255+2668A>C||||||WARNING_TRANSCRIPT_INCOMPLETE |
| RP11-603B24.1-RP11-467N20.1 | rs200570133 | 37 | 15 | 22590305 | 22590305 | + | SNP | T | T | G | SRR8586409 | Missense_Mutation | 0/1:38,10:48:99:201,0,1121 | 48;ANN=G|intergenic_region|MODIFIER|RP11-603B24.1-RP11-467N20.1|ENSG00000258732-ENSG00000259501|intergenic_region|ENSG00000258732-ENSG00000259501|||n.22590305T>G|||||| |
| NA3 | rs1435163 | 37 | 15 | 78893556 | 78893556 | + | SNP | C | C | A | SRR8586409 | Missense_Mutation | 0/1:24,22:46:99:690,0,823 | 46;ANN=A|upstream_gene_variant|MODIFIER|NA3|ENSG00000080644|transcript|ENST00000559002|processed_transcript||n.-4482G>T|||||4482|,A|downstream_gene_variant|MODIFIER|NA3|ENSG00000080644|transcript|ENST00000558903|processed_transcript||n.*562G>T|||||562|,A|intron_variant|MODIFIER|NA3|ENSG00000080644|transcript|ENST00000326828|protein_coding|5/5|c.1389+39G>T||||||,A|intron_variant|MODIFIER|NA3|ENSG00000080644|transcript|ENST00000559658|nonsense_mediated_decay|5/7|c.1389+39G>T||||||,A|intron_variant|MODIFIER|NA3|ENSG00000080644|transcript|ENST00000348639|protein_coding|5/5|c.1389+39G>T|||||| |
| CTD-2544M6.1-FAM169B | rs58523117 | 37 | 15 | 98959970 | 98959970 | + | DEL | GGT | GGT | G | SRR8586409 | In_Frame_Del | 0/1:32,32:64:99:1026,0,1029 | 69;ANN=G|intergenic_region|MODIFIER|CTD-2544M6.1-FAM169B|ENSG00000259199-ENSG00000185087|intergenic_region|ENSG00000259199-ENSG00000185087|||n.98959971_98959972delGT|||||| |
| NPIPB5 | rs4114947 | 37 | 16 | 22545054 | 22545054 | + | SNP | G | G | C | SRR8586409 | Nonsense_Mutation | 0/1:5,41:46:38:1409,0,38 | 46;ANN=C|synonymous_variant|LOW|NPIPB5|ENSG00000243716|transcript|ENST00000424340|protein_coding|7/7|c.750G>C|p.Pro250Pro|1029/3801|750/3402|250/1133||WARNING_REF_DOES_NOT_MATCH_GENOME,C|synonymous_variant|LOW|NPIPB5|ENSG00000243716|transcript|ENST00000415833|protein_coding|11/11|c.750G>C|p.Pro250Pro|1232/2268|750/1786|250/594||WARNING_TRANSCRIPT_INCOMPLETE&WARNING_REF_DOES_NOT_MATCH_GENOME,C|synonymous_variant|LOW|NPIPB5|ENSG00000243716|transcript|ENST00000517539|protein_coding|8/8|c.750G>C|p.Pro250Pro|825/3569|750/3402|250/1133||WARNING_REF_DOES_NOT_MATCH_GENOME,C|synonymous_variant|LOW|NPIPB5|ENSG00000243716|transcript|ENST00000528249|protein_coding|7/7|c.750G>C|p.Pro250Pro|870/1906|750/1786|250/594||WARNING_TRANSCRIPT_INCOMPLETE&WARNING_REF_DOES_NOT_MATCH_GENOME,C|3_prime_UTR_variant|MODIFIER|NPIPB5|ENSG00000243716|transcript|ENST00000521555|nonsense_mediated_decay|9/9|c.*732G>C|||||14566|WARNING_REF_DOES_NOT_MATCH_GENOME,C|3_prime_UTR_variant|MODIFIER|NPIPB5|ENSG00000243716|transcript|ENST00000541664|nonsense_mediated_decay|9/9|c.*713G>C|||||14402|WARNING_REF_DOES_NOT_MATCH_GENOME,C|downstream_gene_variant|MODIFIER|NPIPB5|ENSG00000243716|transcript|ENST00000543997|retained_intron||n.*1644G>C|||||1644|,C|downstream_gene_variant|MODIFIER|NPIPB5|ENSG00000243716|transcript|ENST00000539604|nonsense_mediated_decay||c.*5950G>C|||||8|WARNING_TRANSCRIPT_NO_START_CODON,C|non_coding_transcript_exon_variant|MODIFIER|NPIPB5|ENSG00000243716|transcript|ENST00000415654|processed_transcript|23/23|n.2928G>C||||||WARNING_REF_DOES_NOT_MATCH_GENOME,C|non_coding_transcript_exon_variant|MODIFIER|NPIPB5|ENSG00000243716|transcript|ENST00000442450|retained_intron|3/3|n.773G>C||||||WARNING_REF_DOES_NOT_MATCH_GENOME |
| GRAPL | rs369169614 | 37 | 17 | 19061983 | 19061983 | + | INS | A | A | AT | SRR8586409 | Frame_Shift_Ins | 0/1:31,33:64:99:1138,0,1044 | 64;ANN=AT|3_prime_UTR_variant|MODIFIER|GRAPL|ENSG00000189152|transcript|ENST00000344415|protein_coding|4/4|c.*295dupT|||||296|INFO_REALIGN_3_PRIME,AT|3_prime_UTR_variant|MODIFIER|GRAPL|ENSG00000189152|transcript|ENST00000574324|protein_coding|3/3|c.*295dupT|||||296|WARNING_TRANSCRIPT_NO_START_CODON&INFO_REALIGN_3_PRIME,AT|3_prime_UTR_variant|MODIFIER|GRAPL|ENSG00000189152|transcript|ENST00000583540|nonsense_mediated_decay|3/3|c.*408dupT|||||14557|WARNING_TRANSCRIPT_NO_START_CODON&INFO_REALIGN_3_PRIME,AT|upstream_gene_variant|MODIFIER|RP11-160E2.11|ENSG00000262292|transcript|ENST00000572818|lincRNA||n.-2119_-2118insT|||||2118|,AT|intron_variant|MODIFIER|AC007952.6|ENSG00000197665|transcript|ENST00000399083|protein_coding|1/1|c.68-51dupA||||||,AT|intron_variant|MODIFIER|AC007952.6|ENSG00000197665|transcript|ENST00000399087|protein_coding|1/2|c.68-51dupA||||||,AT|intron_variant|MODIFIER|AC007952.6|ENSG00000197665|transcript|ENST00000436381|protein_coding|1/2|c.116-51dupA|||||| |
| GRAPL | rs373466781 | 37 | 17 | 19062028 | 19062028 | + | SNP | C | C | T | SRR8586409 | Nonstop_Mutation | 0/1:63,30:93:99:770,0,1706 | 93;ANN=T|3_prime_UTR_variant|MODIFIER|GRAPL|ENSG00000189152|transcript|ENST00000344415|protein_coding|4/4|c.*339C>T|||||339|WARNING_REF_DOES_NOT_MATCH_GENOME,T|3_prime_UTR_variant|MODIFIER|GRAPL|ENSG00000189152|transcript|ENST00000574324|protein_coding|3/3|c.*339C>T|||||339|WARNING_TRANSCRIPT_NO_START_CODON&WARNING_REF_DOES_NOT_MATCH_GENOME,T|3_prime_UTR_variant|MODIFIER|GRAPL|ENSG00000189152|transcript|ENST00000583540|nonsense_mediated_decay|3/3|c.*452C>T|||||14600|WARNING_TRANSCRIPT_NO_START_CODON&WARNING_REF_DOES_NOT_MATCH_GENOME,T|upstream_gene_variant|MODIFIER|RP11-160E2.11|ENSG00000262292|transcript|ENST00000572818|lincRNA||n.-2074C>T|||||2074|,T|intron_variant|MODIFIER|AC007952.6|ENSG00000197665|transcript|ENST00000399083|protein_coding|1/1|c.67+64G>A||||||,T|intron_variant|MODIFIER|AC007952.6|ENSG00000197665|transcript|ENST00000399087|protein_coding|1/2|c.67+64G>A||||||,T|intron_variant|MODIFIER|AC007952.6|ENSG00000197665|transcript|ENST00000436381|protein_coding|1/2|c.115+64G>A|||||| |
| GRAPL | rs201695897 | 37 | 17 | 19062050 | 19062050 | + | SNP | T | T | C | SRR8586409 | Nonstop_Mutation | 0/1:69,30:99:99:575,0,2004 | 99;ANN=C|3_prime_UTR_variant|MODIFIER|GRAPL|ENSG00000189152|transcript|ENST00000344415|protein_coding|4/4|c.*361T>C|||||361|WARNING_REF_DOES_NOT_MATCH_GENOME,C|3_prime_UTR_variant|MODIFIER|GRAPL|ENSG00000189152|transcript|ENST00000574324|protein_coding|3/3|c.*361T>C|||||361|WARNING_TRANSCRIPT_NO_START_CODON&WARNING_REF_DOES_NOT_MATCH_GENOME,C|3_prime_UTR_variant|MODIFIER|GRAPL|ENSG00000189152|transcript|ENST00000583540|nonsense_mediated_decay|3/3|c.*474T>C|||||14622|WARNING_TRANSCRIPT_NO_START_CODON&WARNING_REF_DOES_NOT_MATCH_GENOME,C|upstream_gene_variant|MODIFIER|RP11-160E2.11|ENSG00000262292|transcript|ENST00000572818|lincRNA||n.-2052T>C|||||2052|,C|intron_variant|MODIFIER|AC007952.6|ENSG00000197665|transcript|ENST00000399083|protein_coding|1/1|c.67+42A>G||||||,C|intron_variant|MODIFIER|AC007952.6|ENSG00000197665|transcript|ENST00000399087|protein_coding|1/2|c.67+42A>G||||||,C|intron_variant|MODIFIER|AC007952.6|ENSG00000197665|transcript|ENST00000436381|protein_coding|1/2|c.115+42A>G|||||| |
| RPS8P3-RP11-25O3.1 | rs7233791 | 37 | 18 | 49783514 | 49783514 | + | SNP | G | G | C | SRR8586409 | Missense_Mutation | 0/1:62,50:112:99:2220,0,3570 | 112;ANN=C|intergenic_region|MODIFIER|RPS8P3-RP11-25O3.1|ENSG00000215457-ENSG00000266335|intergenic_region|ENSG00000215457-ENSG00000266335|||n.49783514G>C|||||| |
| PDE4C | rs199580670 | 37 | 19 | 18364937 | 18364937 | + | DEL | TGGCGCGGGGGGCTCAAAACGGG | TGGCGCGGGGGGCTCAAAACGGG | T | SRR8586409 | In_Frame_Del | 0/1:9,7:16:99:268,0,356 | 22;ANN=T|upstream_gene_variant|MODIFIER|PDE4C|ENSG00000105650|transcript|ENST00000600667|retained_intron||n.-1718_-1697delCCCGTTTTGAGCCCCCCGCGCC|||||1697|,T|downstream_gene_variant|MODIFIER|KIAA1683|ENSG00000130518|transcript|ENST00000392413|protein_coding||c.*3031_*3052delCCCGTTTTGAGCCCCCCGCGCC|||||2970|,T|downstream_gene_variant|MODIFIER|KIAA1683|ENSG00000130518|transcript|ENST00000600328|protein_coding||c.*3031_*3052delCCCGTTTTGAGCCCCCCGCGCC|||||2970|,T|downstream_gene_variant|MODIFIER|KIAA1683|ENSG00000130518|transcript|ENST00000600359|protein_coding||c.*3031_*3052delCCCGTTTTGAGCCCCCCGCGCC|||||2970|,T|downstream_gene_variant|MODIFIER|KIAA1683|ENSG00000130518|transcript|ENST00000599638|retained_intron||n.*2950_*2971delCCCGTTTTGAGCCCCCCGCGCC|||||2971|,T|intron_variant|MODIFIER|PDE4C|ENSG00000105650|transcript|ENST00000355502|protein_coding|1/18|c.-673+1071_-673+1092delCCCGTTTTGAGCCCCCCGCGCC||||||,T|intron_variant|MODIFIER|PDE4C|ENSG00000105650|transcript|ENST00000596647|processed_transcript|1/3|n.132+1071_132+1092delCCCGTTTTGAGCCCCCCGCGCC|||||| |
| SLC8A1-AS1 | rs61486102 | 37 | 2 | 40269398 | 40269398 | + | SNP | T | T | C | SRR8586409 | Missense_Mutation | 0/1:102,16:118:99:194,0,4135 | 118;ANN=C|upstream_gene_variant|MODIFIER|SLC8A1-AS1|ENSG00000227028|transcript|ENST00000439606|antisense||n.-92T>C|||||92|,C|intron_variant|MODIFIER|SLC8A1-AS1|ENSG00000227028|transcript|ENST00000599740|antisense|1/1|n.74-209426T>C||||||,C|intron_variant|MODIFIER|SLC8A1-AS1|ENSG00000227028|transcript|ENST00000418854|antisense|2/2|n.133-62651T>C|||||| |
| PCBP1-AS1 | rs774795480 | 37 | 2 | 70271856 | 70271856 | + | DEL | TTTTC | TTTTC | T | SRR8586409 | In_Frame_Del | 0/1:4,49:53:39:2046,0,39 | 55;ANN=T|downstream_gene_variant|MODIFIER|PCBP1-AS1|ENSG00000179818|transcript|ENST00000458698|antisense||n.*531_*534delGAAA|||||534|,T|downstream_gene_variant|MODIFIER|PCBP1-AS1|ENSG00000179818|transcript|ENST00000603347|antisense||n.*1840_*1843delGAAA|||||1843|,T|downstream_gene_variant|MODIFIER|PCBP1-AS1|ENSG00000179818|transcript|ENST00000604346|antisense||n.*1886_*1889delGAAA|||||1889|,T|downstream_gene_variant|MODIFIER|PCBP1-AS1|ENSG00000179818|transcript|ENST00000413791|antisense||n.*3959_*3962delGAAA|||||3962|,T|downstream_gene_variant|MODIFIER|PCBP1-AS1|ENSG00000179818|transcript|ENST00000416506|antisense||n.*3963_*3966delGAAA|||||3966|,T|downstream_gene_variant|MODIFIER|PCBP1-AS1|ENSG00000179818|transcript|ENST00000439670|antisense||n.*4317_*4320delGAAA|||||4320|,T|intron_variant|MODIFIER|PCBP1-AS1|ENSG00000179818|transcript|ENST00000416395|antisense|3/4|n.394+6475_394+6478delGAAA||||||,T|intron_variant|MODIFIER|PCBP1-AS1|ENSG00000179818|transcript|ENST00000435880|antisense|6/10|n.528+6475_528+6478delGAAA||||||,T|intron_variant|MODIFIER|PCBP1-AS1|ENSG00000179818|transcript|ENST00000457076|antisense|3/3|n.429+6475_429+6478delGAAA||||||,T|intron_variant|MODIFIER|PCBP1-AS1|ENSG00000179818|transcript|ENST00000413436|antisense|4/6|n.244+6475_244+6478delGAAA||||||,T|intron_variant|MODIFIER|PCBP1-AS1|ENSG00000179818|transcript|ENST00000418564|antisense|3/4|n.331+6475_331+6478delGAAA||||||,T|intron_variant|MODIFIER|PCBP1-AS1|ENSG00000179818|transcript|ENST00000415222|antisense|3/4|n.244+6475_244+6478delGAAA||||||,T|intron_variant|MODIFIER|PCBP1-AS1|ENSG00000179818|transcript|ENST00000594376|antisense|2/3|n.158+6475_158+6478delGAAA||||||,T|intron_variant|MODIFIER|PCBP1-AS1|ENSG00000179818|transcript|ENST00000456161|antisense|4/4|n.677+6475_677+6478delGAAA||||||,T|intron_variant|MODIFIER|PCBP1-AS1|ENSG00000179818|transcript|ENST00000419963|antisense|4/4|n.369+6475_369+6478delGAAA||||||,T|intron_variant|MODIFIER|PCBP1-AS1|ENSG00000179818|transcript|ENST00000599673|antisense|4/5|n.391+6475_391+6478delGAAA||||||,T|intron_variant|MODIFIER|PCBP1-AS1|ENSG00000179818|transcript|ENST00000418308|antisense|3/4|n.331+6475_331+6478delGAAA||||||,T|intron_variant|MODIFIER|PCBP1-AS1|ENSG00000179818|transcript|ENST00000596028|antisense|6/6|n.730+6475_730+6478delGAAA||||||,T|intron_variant|MODIFIER|PCBP1-AS1|ENSG00000179818|transcript|ENST00000601431|antisense|2/3|n.141+6475_141+6478delGAAA||||||,T|intron_variant|MODIFIER|PCBP1-AS1|ENSG00000179818|transcript|ENST00000452431|antisense|4/4|n.456+6475_456+6478delGAAA||||||,T|intron_variant|MODIFIER|PCBP1-AS1|ENSG00000179818|transcript|ENST00000423402|antisense|4/4|n.482+6475_482+6478delGAAA||||||,T|intron_variant|MODIFIER|PCBP1-AS1|ENSG00000179818|transcript|ENST00000411429|antisense|4/4|n.481+6475_481+6478delGAAA||||||,T|intron_variant|MODIFIER|PCBP1-AS1|ENSG00000179818|transcript|ENST00000420309|antisense|4/4|n.514+6475_514+6478delGAAA||||||,T|intron_variant|MODIFIER|PCBP1-AS1|ENSG00000179818|transcript|ENST00000434781|antisense|3/4|n.424+6475_424+6478delGAAA||||||,T|intron_variant|MODIFIER|PCBP1-AS1|ENSG00000179818|transcript|ENST00000415742|antisense|4/5|n.406+6475_406+6478delGAAA||||||,T|intron_variant|MODIFIER|PCBP1-AS1|ENSG00000179818|transcript|ENST00000457770|antisense|4/4|n.482+6475_482+6478delGAAA||||||,T|intron_variant|MODIFIER|PCBP1-AS1|ENSG00000179818|transcript|ENST00000366234|antisense|4/4|n.385+6475_385+6478delGAAA||||||,T|intron_variant|MODIFIER|PCBP1-AS1|ENSG00000179818|transcript|ENST00000599427|antisense|2/3|n.236+6475_236+6478delGAAA||||||,T|intron_variant|MODIFIER|PCBP1-AS1|ENSG00000179818|transcript|ENST00000597318|antisense|4/5|n.242+6475_242+6478delGAAA||||||,T|intron_variant|MODIFIER|PCBP1-AS1|ENSG00000179818|transcript|ENST00000425333|antisense|3/4|n.244+6475_244+6478delGAAA||||||,T|intron_variant|MODIFIER|PCBP1-AS1|ENSG00000179818|transcript|ENST00000425601|antisense|3/4|n.331+6475_331+6478delGAAA||||||,T|intron_variant|MODIFIER|PCBP1-AS1|ENSG00000179818|transcript|ENST00000429599|antisense|4/5|n.820+6475_820+6478delGAAA||||||,T|intron_variant|MODIFIER|PCBP1-AS1|ENSG00000179818|transcript|ENST00000449178|antisense|4/5|n.369+6475_369+6478delGAAA||||||,T|intron_variant|MODIFIER|PCBP1-AS1|ENSG00000179818|transcript|ENST00000458686|antisense|7/7|n.902+6475_902+6478delGAAA||||||,T|intron_variant|MODIFIER|PCBP1-AS1|ENSG00000179818|transcript|ENST00000421843|antisense|4/4|n.546+6475_546+6478delGAAA||||||,T|intron_variant|MODIFIER|PCBP1-AS1|ENSG00000179818|transcript|ENST00000432604|antisense|5/5|n.451+6475_451+6478delGAAA||||||,T|intron_variant|MODIFIER|PCBP1-AS1|ENSG00000179818|transcript|ENST00000421255|antisense|6/6|n.577+6475_577+6478delGAAA||||||,T|intron_variant|MODIFIER|PCBP1-AS1|ENSG00000179818|transcript|ENST00000422515|antisense|4/4|n.477+6475_477+6478delGAAA||||||,T|intron_variant|MODIFIER|PCBP1-AS1|ENSG00000179818|transcript|ENST00000413069|antisense|5/5|n.505+6475_505+6478delGAAA||||||,T|intron_variant|MODIFIER|PCBP1-AS1|ENSG00000179818|transcript|ENST00000444410|antisense|6/6|n.528+6475_528+6478delGAAA|||||| |
| NCKAP5 | rs16829774 | 37 | 2 | 133929113 | 133929113 | + | SNP | G | G | A | SRR8586409 | Missense_Mutation | 0/1:20,25:45:99:718,0,551 | 45;ANN=A|intron_variant|MODIFIER|NCKAP5|ENSG00000176771|transcript|ENST00000317721|protein_coding|3/17|c.208-41430C>T||||||,A|intron_variant|MODIFIER|NCKAP5|ENSG00000176771|transcript|ENST00000409261|protein_coding|5/19|c.208-41430C>T||||||,A|intron_variant|MODIFIER|NCKAP5|ENSG00000176771|transcript|ENST00000409213|protein_coding|5/17|c.208-41430C>T||||||,A|intron_variant|MODIFIER|NCKAP5|ENSG00000176771|transcript|ENST00000405974|protein_coding|3/15|c.208-41430C>T||||||,A|intron_variant|MODIFIER|NCKAP5|ENSG00000176771|transcript|ENST00000427594|protein_coding|3/4|c.193-41430C>T||||||WARNING_TRANSCRIPT_NO_START_CODON |
| PDE11A | rs146313925 | 37 | 2 | 178662395 | 178662395 | + | SNP | T | T | C | SRR8586409 | Missense_Mutation | 0/1:223,50:273:99:705,0,5864 | 273;ANN=C|intron_variant|MODIFIER|PDE11A|ENSG00000128655|transcript|ENST00000286063|protein_coding|9/19|c.1737+19161A>G||||||,C|intron_variant|MODIFIER|PDE11A|ENSG00000128655|transcript|ENST00000358450|protein_coding|10/20|c.987+19161A>G||||||,C|intron_variant|MODIFIER|PDE11A|ENSG00000128655|transcript|ENST00000409504|protein_coding|8/19|c.663+19161A>G||||||,C|intron_variant|MODIFIER|PDE11A|ENSG00000128655|transcript|ENST00000389683|protein_coding|6/16|c.405+19161A>G||||||,C|intron_variant|MODIFIER|PDE11A|ENSG00000128655|transcript|ENST00000449286|protein_coding|8/18|c.663+19161A>G||||||,C|intron_variant|MODIFIER|PDE11A|ENSG00000128655|transcript|ENST00000433879|protein_coding|6/13|c.558+20190A>G||||||WARNING_TRANSCRIPT_NO_START_CODON,C|intron_variant|MODIFIER|PDE11A|ENSG00000128655|transcript|ENST00000497003|processed_transcript|8/14|n.779+19161A>G||||||,C|intron_variant|MODIFIER|PDE11A|ENSG00000128655|transcript|ENST00000492761|processed_transcript|6/6|n.594-448A>G|||||| |
| PDE11A | rs12994774 | 37 | 2 | 178663311 | 178663311 | + | SNP | A | A | C | SRR8586409 | Missense_Mutation | 0/1:268,102:370:99:2660,0,7393 | 370;ANN=C|intron_variant|MODIFIER|PDE11A|ENSG00000128655|transcript|ENST00000286063|protein_coding|9/19|c.1737+18245T>G||||||,C|intron_variant|MODIFIER|PDE11A|ENSG00000128655|transcript|ENST00000358450|protein_coding|10/20|c.987+18245T>G||||||,C|intron_variant|MODIFIER|PDE11A|ENSG00000128655|transcript|ENST00000409504|protein_coding|8/19|c.663+18245T>G||||||,C|intron_variant|MODIFIER|PDE11A|ENSG00000128655|transcript|ENST00000389683|protein_coding|6/16|c.405+18245T>G||||||,C|intron_variant|MODIFIER|PDE11A|ENSG00000128655|transcript|ENST00000449286|protein_coding|8/18|c.663+18245T>G||||||,C|intron_variant|MODIFIER|PDE11A|ENSG00000128655|transcript|ENST00000433879|protein_coding|6/13|c.558+19274T>G||||||WARNING_TRANSCRIPT_NO_START_CODON,C|intron_variant|MODIFIER|PDE11A|ENSG00000128655|transcript|ENST00000497003|processed_transcript|8/14|n.779+18245T>G||||||,C|intron_variant|MODIFIER|PDE11A|ENSG00000128655|transcript|ENST00000492761|processed_transcript|6/6|n.594-1364T>G|||||| |
| PDE11A | rs12998857 | 37 | 2 | 178668882 | 178668882 | + | SNP | A | A | C | SRR8586409 | Missense_Mutation | 0/1:165,107:272:99:2464,0,4304 | 279;ANN=C|intron_variant|MODIFIER|PDE11A|ENSG00000128655|transcript|ENST00000286063|protein_coding|9/19|c.1737+12674T>G||||||,C|intron_variant|MODIFIER|PDE11A|ENSG00000128655|transcript|ENST00000358450|protein_coding|10/20|c.987+12674T>G||||||,C|intron_variant|MODIFIER|PDE11A|ENSG00000128655|transcript|ENST00000409504|protein_coding|8/19|c.663+12674T>G||||||,C|intron_variant|MODIFIER|PDE11A|ENSG00000128655|transcript|ENST00000389683|protein_coding|6/16|c.405+12674T>G||||||,C|intron_variant|MODIFIER|PDE11A|ENSG00000128655|transcript|ENST00000449286|protein_coding|8/18|c.663+12674T>G||||||,C|intron_variant|MODIFIER|PDE11A|ENSG00000128655|transcript|ENST00000433879|protein_coding|6/13|c.558+13703T>G||||||WARNING_TRANSCRIPT_NO_START_CODON,C|intron_variant|MODIFIER|PDE11A|ENSG00000128655|transcript|ENST00000497003|processed_transcript|8/14|n.779+12674T>G||||||,C|intron_variant|MODIFIER|PDE11A|ENSG00000128655|transcript|ENST00000492761|processed_transcript|6/6|n.594-6935T>G|||||| |
| KCTD18 | rs12623282 | 37 | 2 | 201382734 | 201382734 | + | SNP | G | G | C | SRR8586409 | Missense_Mutation | 0/1:13,15:28:99:393,0,348 | 28;ANN=C|intron_variant|MODIFIER|KCTD18|ENSG00000155729|transcript|ENST00000468413|processed_transcript|1/5|n.87+1687C>G||||||,C|intron_variant|MODIFIER|SGOL2|ENSG00000163535|transcript|ENST00000418045|protein_coding|1/3|c.-120+7745G>C||||||WARNING_TRANSCRIPT_INCOMPLETE |
| AC007563.5 | rs3796032 | 37 | 2 | 217848213 | 217848213 | + | SNP | C | C | T | SRR8586409 | Missense_Mutation | 0/1:304,141:445:99:4261,0,13050 | 446;ANN=T|intron_variant|MODIFIER|AC007563.5|ENSG00000236886|transcript|ENST00000447289|antisense|3/3|n.511-10468C>T||||||,T|intron_variant|MODIFIER|AC007563.5|ENSG00000236886|transcript|ENST00000607591|antisense|2/2|n.272+1361C>T|||||| |
|  | rs538653835 | 37 | 20 | 63488235 | 63488235 | + | DEL | GC | G | G | SRR8586409 | Frame_Shift_Del | 1/1:9,484:493:99:21446,1293,0 | 521;ANN=G||MODIFIER|||||||||||||ERROR_OUT_OF_OMOSOME_RANGE |
| NFKBIZ | rs771150914 | 37 | 3 | 101576663 | 101576663 | + | SNP | A | A | G | SRR8586409 | Nonsense_Mutation | 0/1:31,74:105:99:2141,0,756 | 106;ANN=G|downstream_gene_variant|MODIFIER|NFKBIZ|ENSG00000144802|transcript|ENST00000483180|protein_coding||c.*360A>G|||||360|WARNING_TRANSCRIPT_NO_STOP_CODON,G|downstream_gene_variant|MODIFIER|NFKBIZ|ENSG00000144802|transcript|ENST00000491281|protein_coding||c.*4604A>G|||||4604|WARNING_TRANSCRIPT_INCOMPLETE,G|downstream_gene_variant|MODIFIER|NFKBIZ|ENSG00000144802|transcript|ENST00000465476|retained_intron||n.*1802A>G|||||1802|,G|intron_variant|MODIFIER|NFKBIZ|ENSG00000144802|transcript|ENST00000326172|protein_coding|11/11|c.2103+360A>G||||||,G|intron_variant|MODIFIER|NFKBIZ|ENSG00000144802|transcript|ENST00000394054|protein_coding|12/12|c.1803+360A>G||||||,G|intron_variant|MODIFIER|NFKBIZ|ENSG00000144802|transcript|ENST00000326151|protein_coding|12/12|c.1737+360A>G||||||,G|intron_variant|MODIFIER|NFKBIZ|ENSG00000144802|transcript|ENST00000477601|protein_coding|2/2|c.336+360A>G||||||,G|intron_variant|MODIFIER|NFKBIZ|ENSG00000144802|transcript|ENST00000495089|retained_intron|2/2|n.671+360A>G||||||,G|intron_variant|MODIFIER|NFKBIZ|ENSG00000144802|transcript|ENST00000495719|retained_intron|1/1|n.260+360A>G|||||| |
| RP11-12K22.1 | rs13122622 | 37 | 4 | 64351087 | 64351087 | + | SNP | T | T | A | SRR8586409 | Missense_Mutation | 0/1:77,22:99:99:487,0,2075 | 99;ANN=A|intron_variant|MODIFIER|RP11-12K22.1|ENSG00000250775|transcript|ENST00000508825|lincRNA|1/2|n.58+8852A>T|||||| |
| GRIA2 | rs566305935 | 37 | 4 | 158171636 | 158171636 | + | SNP | A | A | G | SRR8586409 | Missense_Mutation | 0/1:31,88:119:99:3641,0,1112 | 119;ANN=G|intron_variant|MODIFIER|GRIA2|ENSG00000120251|transcript|ENST00000264426|protein_coding|2/15|c.229+28677A>G||||||,G|intron_variant|MODIFIER|GRIA2|ENSG00000120251|transcript|ENST00000504801|processed_transcript|1/1|n.254+46049A>G||||||,G|intron_variant|MODIFIER|GRIA2|ENSG00000120251|transcript|ENST00000507898|protein_coding|2/15|c.88+28677A>G||||||,G|intron_variant|MODIFIER|GRIA2|ENSG00000120251|transcript|ENST00000393815|protein_coding|2/15|c.88+28677A>G||||||,G|intron_variant|MODIFIER|GRIA2|ENSG00000120251|transcript|ENST00000509417|protein_coding|3/3|c.229+28677A>G||||||WARNING_TRANSCRIPT_INCOMPLETE,G|intron_variant|MODIFIER|GRIA2|ENSG00000120251|transcript|ENST00000296526|protein_coding|2/15|c.229+28677A>G||||||,G|intron_variant|MODIFIER|GRIA2|ENSG00000120251|transcript|ENST00000471736|retained_intron|2/14|n.548+28677A>G||||||,G|intron_variant|MODIFIER|GRIA2|ENSG00000120251|transcript|ENST00000506284|protein_coding|2/2|c.88+28677A>G||||||WARNING_TRANSCRIPT_NO_STOP_CODON,G|intron_variant|MODIFIER|GRIA2|ENSG00000120251|transcript|ENST00000323661|nonsense_mediated_decay|2/16|c.88+28677A>G||||||,G|intron_variant|MODIFIER|GRIA2|ENSG00000120251|transcript|ENST00000505888|protein_coding|1/2|c.88+28677A>G||||||WARNING_TRANSCRIPT_INCOMPLETE,G|intron_variant|MODIFIER|GRIA2|ENSG00000120251|transcript|ENST00000449365|protein_coding|1/14|c.88+28677A>G||||||,G|intron_variant|MODIFIER|GRIA2|ENSG00000120251|transcript|ENST00000503437|protein_coding|2/4|c.-153+631A>G||||||WARNING_TRANSCRIPT_INCOMPLETE |
| CENPU | rs763448406 | 37 | 4 | 185639036 | 185639036 | + | SNP | C | C | T | SRR8586409 | Missense_Mutation | 0/1:118,40:158:99:144,0,3275 | 158;ANN=T|upstream_gene_variant|MODIFIER|CENPU|ENSG00000151725|transcript|ENST00000506535|processed_transcript||n.-3941G>A|||||3941|,T|intron_variant|MODIFIER|CENPU|ENSG00000151725|transcript|ENST00000281453|protein_coding|4/12|c.321-686G>A||||||,T|intron_variant|MODIFIER|CENPU|ENSG00000151725|transcript|ENST00000510146|nonsense_mediated_decay|4/11|c.321-686G>A||||||,T|intron_variant|MODIFIER|CENPU|ENSG00000151725|transcript|ENST00000541971|protein_coding|4/11|c.321-686G>A||||||,T|intron_variant|MODIFIER|CENPU|ENSG00000151725|transcript|ENST00000514781|protein_coding|4/5|c.234-686G>A||||||WARNING_TRANSCRIPT_NO_STOP_CODON |
| SLC1A3 | rs145798494 | 37 | 5 | 36649326 | 36649326 | + | DEL | CAACAAAAGCAAAACTCCATCTCAAAAAAAAAAAAA | CAACAAAAGCAAAACTCCATCTCAAAAAAAAAAAAA | C | SRR8586409 | In_Frame_Del | 0/1:30,27:57:99:890,0,1131 | 58;ANN=C|intron_variant|MODIFIER|SLC1A3|ENSG00000079215|transcript|ENST00000265113|protein_coding|3/9|c.319+19638_319+19672delAACAAAAGCAAAACTCCATCTCAAAAAAAAAAAAA||||||,C|intron_variant|MODIFIER|SLC1A3|ENSG00000079215|transcript|ENST00000381918|protein_coding|2/7|c.319+19638_319+19672delAACAAAAGCAAAACTCCATCTCAAAAAAAAAAAAA||||||,C|intron_variant|MODIFIER|SLC1A3|ENSG00000079215|transcript|ENST00000514563|processed_transcript|2/2|n.384+19638_384+19672delAACAAAAGCAAAACTCCATCTCAAAAAAAAAAAAA||||||,C|intron_variant|MODIFIER|SLC1A3|ENSG00000079215|transcript|ENST00000509272|processed_transcript|2/2|n.339+19638_339+19672delAACAAAAGCAAAACTCCATCTCAAAAAAAAAAAAA|||||| |
| CTC-321K16.1 | rs75879538 | 37 | 5 | 134927699 | 134927699 | + | SNP | A | A | G | SRR8586409 | Missense_Mutation | 0/1:6,21:27:99:671,0,121 | 28;ANN=G|intron_variant|MODIFIER|CTC-321K16.1|ENSG00000250167|transcript|ENST00000514446|antisense|1/2|n.413+12412A>G||||||,G|intron_variant|MODIFIER|CTC-321K16.1|ENSG00000250167|transcript|ENST00000509372|antisense|1/3|n.75-9041A>G|||||| |
| CTC-321K16.1 | rs78447151 | 37 | 5 | 134927713 | 134927713 | + | SNP | C | C | T | SRR8586409 | Missense_Mutation | 0/1:6,24:30:99:727,0,112 | 33;ANN=T|intron_variant|MODIFIER|CTC-321K16.1|ENSG00000250167|transcript|ENST00000514446|antisense|1/2|n.413+12426C>T||||||,T|intron_variant|MODIFIER|CTC-321K16.1|ENSG00000250167|transcript|ENST00000509372|antisense|1/3|n.75-9027C>T|||||| |
| 7-Sep | rs148964270 | 37 | 7 | 35903153 | 35903153 | + | SNP | C | C | T | SRR8586409 | Missense_Mutation | 0/1:209,74:283:99:1682,0,5386 | 283;ANN=T|upstream_gene_variant|MODIFIER|SEPT7|ENSG00000122545|transcript|ENST00000473201|retained_intron||n.-9C>T|||||9|,T|intron_variant|MODIFIER|SEPT7|ENSG00000122545|transcript|ENST00000399034|protein_coding|3/13|c.173-9C>T||||||,T|intron_variant|MODIFIER|SEPT7|ENSG00000122545|transcript|ENST00000435235|protein_coding|2/12|c.11-9C>T||||||,T|intron_variant|MODIFIER|SEPT7|ENSG00000122545|transcript|ENST00000469679|protein_coding|3/8|c.167-9C>T||||||WARNING_TRANSCRIPT_NO_STOP_CODON,T|intron_variant|MODIFIER|SEPT7|ENSG00000122545|transcript|ENST00000350320|protein_coding|2/12|c.167-9C>T||||||,T|intron_variant|MODIFIER|SEPT7|ENSG00000122545|transcript|ENST00000399035|protein_coding|3/13|c.167-9C>T||||||,T|intron_variant|MODIFIER|SEPT7|ENSG00000122545|transcript|ENST00000475109|processed_transcript|2/6|n.149-9C>T||||||,T|intron_variant|MODIFIER|SEPT7|ENSG00000122545|transcript|ENST00000494488|protein_coding|2/11|c.128-9C>T||||||WARNING_TRANSCRIPT_NO_START_CODON,T|intron_variant|MODIFIER|SEPT7|ENSG00000122545|transcript|ENST00000425198|nonsense_mediated_decay|1/10|c.104-9C>T||||||WARNING_TRANSCRIPT_NO_START_CODON |
| CALN1 | rs61737661 | 37 | 7 | 71421007 | 71421007 | + | SNP | C | C | T | SRR8586409 | Missense_Mutation | 0/1:50,30:80:99:781,0,1382 | 80;ANN=T|intron_variant|MODIFIER|CALN1|ENSG00000183166|transcript|ENST00000395275|protein_coding|5/6|c.501+67635G>A||||||,T|intron_variant|MODIFIER|CALN1|ENSG00000183166|transcript|ENST00000329008|protein_coding|4/5|c.375+67635G>A||||||,T|intron_variant|MODIFIER|CALN1|ENSG00000183166|transcript|ENST00000395276|protein_coding|5/6|c.375+67635G>A||||||,T|intron_variant|MODIFIER|CALN1|ENSG00000183166|transcript|ENST00000405452|protein_coding|3/4|c.375+67635G>A||||||,T|intron_variant|MODIFIER|CALN1|ENSG00000183166|transcript|ENST00000412588|protein_coding|4/5|c.501+67635G>A||||||,T|intron_variant|MODIFIER|CALN1|ENSG00000183166|transcript|ENST00000431984|protein_coding|4/5|c.375+67635G>A|||||| |
| SEMA3C-AC005008.2 | rs12706950 | 37 | 7 | 80678186 | 80678186 | + | SNP | G | G | A | SRR8586409 | Missense_Mutation | 0/1:121,107:228:99:2992,0,3031 | 228;ANN=A|intergenic_region|MODIFIER|SEMA3C-AC005008.2|ENSG00000075223-ENSG00000237896|intergenic_region|ENSG00000075223-ENSG00000237896|||n.80678186G>A|||||| |
| FLNC | rs2896399 | 37 | 7 | 128479325 | 128479325 | + | SNP | T | T | C | SRR8586409 | Missense_Mutation | 0/1:88,16:104:99:436,0,4936 | 105;ANN=C|upstream_gene_variant|MODIFIER|FLNC|ENSG00000128591|transcript|ENST00000388853|retained_intron||n.-2060T>C|||||2060|,C|intron_variant|MODIFIER|FLNC|ENSG00000128591|transcript|ENST00000325888|protein_coding|8/47|c.1411+468T>C||||||,C|intron_variant|MODIFIER|FLNC|ENSG00000128591|transcript|ENST00000346177|protein_coding|8/46|c.1411+468T>C|||||| |
| FLNC | rs1053120 | 37 | 7 | 128479326 | 128479326 | + | SNP | G | G | C | SRR8586409 | Missense_Mutation | 0/1:90,17:107:99:436,0,4936 | 108;ANN=C|upstream_gene_variant|MODIFIER|FLNC|ENSG00000128591|transcript|ENST00000388853|retained_intron||n.-2059G>C|||||2059|,C|intron_variant|MODIFIER|FLNC|ENSG00000128591|transcript|ENST00000325888|protein_coding|8/47|c.1411+469G>C||||||,C|intron_variant|MODIFIER|FLNC|ENSG00000128591|transcript|ENST00000346177|protein_coding|8/46|c.1411+469G>C|||||| |
| EXOC4 | rs12671131 | 37 | 7 | 133034625 | 133034625 | + | SNP | G | G | C | SRR8586409 | Missense_Mutation | 0/1:21,27:48:99:668,0,453 | 50;ANN=C|intron_variant|MODIFIER|EXOC4|ENSG00000131558|transcript|ENST00000253861|protein_coding|5/17|c.764-6459G>C||||||,C|intron_variant|MODIFIER|EXOC4|ENSG00000131558|transcript|ENST00000393161|protein_coding|5/9|c.764-6459G>C||||||,C|intron_variant|MODIFIER|EXOC4|ENSG00000131558|transcript|ENST00000486013|retained_intron|5/9|n.793-6459G>C||||||,C|intron_variant|MODIFIER|EXOC4|ENSG00000131558|transcript|ENST00000462055|retained_intron|5/8|n.771-6459G>C||||||,C|intron_variant|MODIFIER|EXOC4|ENSG00000131558|transcript|ENST00000539845|protein_coding|5/17|c.461-6459G>C|||||| |
| EXOC4 | rs12673323 | 37 | 7 | 133034703 | 133034703 | + | SNP | T | T | G | SRR8586409 | Missense_Mutation | 0/1:20,57:77:99:1369,0,402 | 79;ANN=G|intron_variant|MODIFIER|EXOC4|ENSG00000131558|transcript|ENST00000253861|protein_coding|5/17|c.764-6381T>G||||||,G|intron_variant|MODIFIER|EXOC4|ENSG00000131558|transcript|ENST00000393161|protein_coding|5/9|c.764-6381T>G||||||,G|intron_variant|MODIFIER|EXOC4|ENSG00000131558|transcript|ENST00000486013|retained_intron|5/9|n.793-6381T>G||||||,G|intron_variant|MODIFIER|EXOC4|ENSG00000131558|transcript|ENST00000462055|retained_intron|5/8|n.771-6381T>G||||||,G|intron_variant|MODIFIER|EXOC4|ENSG00000131558|transcript|ENST00000539845|protein_coding|5/17|c.461-6381T>G|||||| |
| MYOM2 | rs2294062 | 37 | 8 | 2090052 | 2090052 | + | SNP | G | A | A | SRR8586409 | Missense_Mutation | 1/1:18,209:227:99:6723,136,0 | 229;ANN=A|upstream_gene_variant|MODIFIER|MYOM2|ENSG00000036448|transcript|ENST00000518513|retained_intron||n.-1221G>A|||||1221|,A|downstream_gene_variant|MODIFIER|MYOM2|ENSG00000036448|transcript|ENST00000520072|processed_transcript||n.*1311G>A|||||1311|,A|downstream_gene_variant|MODIFIER|MYOM2|ENSG00000036448|transcript|ENST00000519631|processed_transcript||n.*1303G>A|||||1303|,A|intron_variant|MODIFIER|MYOM2|ENSG00000036448|transcript|ENST00000262113|protein_coding|34/36|c.4002-248G>A||||||,A|intron_variant|MODIFIER|MYOM2|ENSG00000036448|transcript|ENST00000523438|protein_coding|21/23|c.2277-248G>A||||||,A|intron_variant|MODIFIER|MYOM2|ENSG00000036448|transcript|ENST00000523595|processed_transcript|4/6|n.455-248G>A||||||,A|intron_variant|MODIFIER|MYOM2|ENSG00000036448|transcript|ENST00000519268|processed_transcript|3/5|n.356-248G>A||||||,A|intron_variant|MODIFIER|MYOM2|ENSG00000036448|transcript|ENST00000520298|processed_transcript|1/2|n.144+1243G>A||||||,A|intron_variant|MODIFIER|MYOM2|ENSG00000036448|transcript|ENST00000520779|processed_transcript|2/4|n.76-248G>A|||||| |
| MYOM2 | rs968381 | 37 | 8 | 2101041 | 2101041 | + | SNP | G | G | A | SRR8586409 | Missense_Mutation | 0/1:123,30:153:99:526,0,3130 | 180;ANN=A|intron_variant|MODIFIER|MYOM2|ENSG00000036448|transcript|ENST00000520779|processed_transcript|4/4|n.154+9661G>A|||||| |
| MYOM2 | rs11774879 | 37 | 8 | 2103604 | 2103604 | + | SNP | A | A | G | SRR8586409 | Missense_Mutation | 0/1:85,29:114:99:713,0,3190 | 120;ANN=G|intron_variant|MODIFIER|MYOM2|ENSG00000036448|transcript|ENST00000520779|processed_transcript|4/4|n.155-9472A>G|||||| |
| NOTCH2 | rs1055339 | 37 | 1 | 120489320 | 120489320 | + | SNP | A | A | G | SRR8586410 | Nonsense_Mutation | 0/1:220,30:250:99:341,0,6238 | 252;ANN=G|downstream_gene_variant|MODIFIER|NOTCH2|ENSG00000134250|transcript|ENST00000479412|retained_intron||n.*3423T>C|||||3423|,G|intron_variant|MODIFIER|NOTCH2|ENSG00000134250|transcript|ENST00000256646|protein_coding|17/33|c.2752+1717T>C|||||| |
| NOTCH2 | rs1699761 | 37 | 1 | 120489416 | 120489416 | + | SNP | C | C | A | SRR8586410 | Nonsense_Mutation | 0/1:238,146:384:99:3775,0,6063 | 389;ANN=A|downstream_gene_variant|MODIFIER|NOTCH2|ENSG00000134250|transcript|ENST00000479412|retained_intron||n.*3327G>T|||||3327|,A|intron_variant|MODIFIER|NOTCH2|ENSG00000134250|transcript|ENST00000256646|protein_coding|17/33|c.2752+1621G>T|||||| |
| NOTCH2 | rs113514538 | 37 | 1 | 120489584 | 120489584 | + | SNP | T | T | G | SRR8586410 | Nonsense_Mutation | 0/1:200,100:300:99:2356,0,5850 | 300;ANN=G|downstream_gene_variant|MODIFIER|NOTCH2|ENSG00000134250|transcript|ENST00000479412|retained_intron||n.*3159A>C|||||3159|,G|intron_variant|MODIFIER|NOTCH2|ENSG00000134250|transcript|ENST00000256646|protein_coding|17/33|c.2752+1453A>C|||||| |
| NOTCH2 | rs74872612 | 37 | 1 | 120489620 | 120489620 | + | SNP | G | G | T | SRR8586410 | Nonsense_Mutation | 0/1:223,67:290:99:1210,0,6471 | 290;ANN=T|downstream_gene_variant|MODIFIER|NOTCH2|ENSG00000134250|transcript|ENST00000479412|retained_intron||n.*3123C>A|||||3123|,T|intron_variant|MODIFIER|NOTCH2|ENSG00000134250|transcript|ENST00000256646|protein_coding|17/33|c.2752+1417C>A|||||| |
| NOTCH2 | rs75842891 | 37 | 1 | 120489652 | 120489652 | + | SNP | C | C | T | SRR8586410 | Nonsense_Mutation | 0/1:215,20:235:99:185,0,10049 | 235;ANN=T|downstream_gene_variant|MODIFIER|NOTCH2|ENSG00000134250|transcript|ENST00000479412|retained_intron||n.*3091G>A|||||3091|,T|intron_variant|MODIFIER|NOTCH2|ENSG00000134250|transcript|ENST00000256646|protein_coding|17/33|c.2752+1385G>A|||||| |
| NOTCH2 | rs78034674 | 37 | 1 | 120489653 | 120489653 | + | SNP | G | G | C | SRR8586410 | Nonsense_Mutation | 0/1:215,20:235:99:185,0,10049 | 235;ANN=C|downstream_gene_variant|MODIFIER|NOTCH2|ENSG00000134250|transcript|ENST00000479412|retained_intron||n.*3090C>G|||||3090|,C|intron_variant|MODIFIER|NOTCH2|ENSG00000134250|transcript|ENST00000256646|protein_coding|17/33|c.2752+1384C>G|||||| |
| NOTCH2 | rs1698582 | 37 | 1 | 120493284 | 120493284 | + | SNP | G | G | A | SRR8586410 | Missense_Mutation | 0/1:190,185:375:99:5334,0,5407 | 375;ANN=A|intron_variant|MODIFIER|NOTCH2|ENSG00000134250|transcript|ENST00000256646|protein_coding|15/33|c.2479+63C>T||||||,A|non_coding_transcript_exon_variant|MODIFIER|NOTCH2|ENSG00000134250|transcript|ENST00000479412|retained_intron|14/14|n.2680C>T||||||WARNING_REF_DOES_NOT_MATCH_GENOME |
| RP11-14N7.2 | rs1778562 | 37 | 1 | 148932939 | 148932939 | + | SNP | A | A | C | SRR8586410 | Nonsense_Mutation | 0/1:48,175:223:99:4756,0,1074 | 223;ANN=C|downstream_gene_variant|MODIFIER|RP11-14N7.2|ENSG00000232527|transcript|ENST00000420597|lincRNA||n.*19A>C|||||19|,C|intron_variant|MODIFIER|RP11-14N7.2|ENSG00000232527|transcript|ENST00000539543|lincRNA|2/3|n.176+19A>C||||||,C|intron_variant|MODIFIER|RP11-14N7.2|ENSG00000232527|transcript|ENST00000452399|lincRNA|2/2|n.199+19A>C||||||,C|intron_variant|MODIFIER|RP11-14N7.2|ENSG00000232527|transcript|ENST00000294715|lincRNA|2/2|n.190+19A>C||||||,C|intron_variant|MODIFIER|RP11-14N7.2|ENSG00000232527|transcript|ENST00000457390|lincRNA|1/1|n.136+19A>C|||||| |
| RP11-14N7.2 | rs3124680 | 37 | 1 | 148933084 | 148933084 | + | SNP | T | T | A | SRR8586410 | Nonsense_Mutation | 0/1:62,130:192:99:3920,0,1559 | 192;ANN=A|downstream_gene_variant|MODIFIER|RP11-14N7.2|ENSG00000232527|transcript|ENST00000420597|lincRNA||n.*164T>A|||||164|,A|intron_variant|MODIFIER|RP11-14N7.2|ENSG00000232527|transcript|ENST00000539543|lincRNA|2/3|n.176+164T>A||||||,A|intron_variant|MODIFIER|RP11-14N7.2|ENSG00000232527|transcript|ENST00000452399|lincRNA|2/2|n.199+164T>A||||||,A|intron_variant|MODIFIER|RP11-14N7.2|ENSG00000232527|transcript|ENST00000294715|lincRNA|2/2|n.190+164T>A||||||,A|intron_variant|MODIFIER|RP11-14N7.2|ENSG00000232527|transcript|ENST00000457390|lincRNA|1/1|n.136+164T>A|||||| |
| RP11-14N7.2 | rs622753 | 37 | 1 | 148933432 | 148933432 | + | SNP | A | A | G | SRR8586410 | Nonsense_Mutation | 0/1:31,69:100:99:1846,0,670 | 100;ANN=G|downstream_gene_variant|MODIFIER|RP11-14N7.2|ENSG00000232527|transcript|ENST00000420597|lincRNA||n.*512A>G|||||512|,G|downstream_gene_variant|MODIFIER|RP11-14N7.2|ENSG00000232527|transcript|ENST00000294715|lincRNA||n.*64A>G|||||64|,G|intron_variant|MODIFIER|RP11-14N7.2|ENSG00000232527|transcript|ENST00000539543|lincRNA|3/3|n.254+64A>G||||||,G|intron_variant|MODIFIER|RP11-14N7.2|ENSG00000232527|transcript|ENST00000452399|lincRNA|2/2|n.199+512A>G||||||,G|non_coding_transcript_exon_variant|MODIFIER|RP11-14N7.2|ENSG00000232527|transcript|ENST00000457390|lincRNA|2/2|n.278A>G||||||WARNING_REF_DOES_NOT_MATCH_GENOME |
| RP11-14N7.2 | rs511710 | 37 | 1 | 148933685 | 148933685 | + | SNP | T | T | C | SRR8586410 | Nonsense_Mutation | 0/1:39,21:60:99:570,0,1101 | 60;ANN=C|downstream_gene_variant|MODIFIER|RP11-14N7.2|ENSG00000232527|transcript|ENST00000420597|lincRNA||n.*765T>C|||||765|,C|downstream_gene_variant|MODIFIER|RP11-14N7.2|ENSG00000232527|transcript|ENST00000294715|lincRNA||n.*317T>C|||||317|,C|downstream_gene_variant|MODIFIER|RP11-14N7.2|ENSG00000232527|transcript|ENST00000457390|lincRNA||n.*106T>C|||||106|,C|intron_variant|MODIFIER|RP11-14N7.2|ENSG00000232527|transcript|ENST00000539543|lincRNA|3/3|n.254+317T>C||||||,C|intron_variant|MODIFIER|RP11-14N7.2|ENSG00000232527|transcript|ENST00000452399|lincRNA|2/2|n.199+765T>C|||||| |
| RP11-14N7.2 | rs512570 | 37 | 1 | 148933778 | 148933778 | + | SNP | A | A | G | SRR8586410 | Nonsense_Mutation | 0/1:52,110:162:99:3293,0,1192 | 162;ANN=G|downstream_gene_variant|MODIFIER|RP11-14N7.2|ENSG00000232527|transcript|ENST00000420597|lincRNA||n.*858A>G|||||858|,G|downstream_gene_variant|MODIFIER|RP11-14N7.2|ENSG00000232527|transcript|ENST00000294715|lincRNA||n.*410A>G|||||410|,G|downstream_gene_variant|MODIFIER|RP11-14N7.2|ENSG00000232527|transcript|ENST00000457390|lincRNA||n.*199A>G|||||199|,G|intron_variant|MODIFIER|RP11-14N7.2|ENSG00000232527|transcript|ENST00000539543|lincRNA|3/3|n.254+410A>G||||||,G|intron_variant|MODIFIER|RP11-14N7.2|ENSG00000232527|transcript|ENST00000452399|lincRNA|2/2|n.199+858A>G|||||| |
| SNAPIN | rs1128894 | 37 | 1 | 153631946 | 153631946 | + | SNP | T | T | C | SRR8586410 | Nonsense_Mutation | 0/1:1147,480:1627:99:9981,0,34906 | 1635;ANN=C|synonymous_variant|LOW|SNAPIN|ENSG00000143553|transcript|ENST00000368685|protein_coding|3/4|c.213T>C|p.Asp71Asp|303/1030|213/411|71/136||,C|downstream_gene_variant|MODIFIER|ILF2|ENSG00000143621|transcript|ENST00000361891|protein_coding||c.*2926A>G|||||2566|,C|downstream_gene_variant|MODIFIER|ILF2|ENSG00000143621|transcript|ENST00000480213|processed_transcript||n.*2595A>G|||||2595|,C|non_coding_transcript_exon_variant|MODIFIER|SNAPIN|ENSG00000143553|transcript|ENST00000474959|processed_transcript|2/3|n.496T>C||||||,C|non_coding_transcript_exon_variant|MODIFIER|SNAPIN|ENSG00000143553|transcript|ENST00000462880|processed_transcript|2/3|n.196T>C||||||,C|non_coding_transcript_exon_variant|MODIFIER|SNAPIN|ENSG00000143553|transcript|ENST00000478558|processed_transcript|1/2|n.744T>C|||||| |
| RP11-385F5.5 | rs71559972 | 37 | 1 | 236695563 | 236695563 | + | INS | T | T | TC | SRR8586410 | Frame_Shift_Ins | 0/1:69,29:98:99:936,0,4041 | 113;ANN=TC|downstream_gene_variant|MODIFIER|RP11-385F5.5|ENSG00000273058|transcript|ENST00000608547|antisense||n.*3909_*3910insG|||||3909|,TC|intron_variant|MODIFIER|LGALS8|ENSG00000116977|transcript|ENST00000352231|protein_coding|3/11|c.46-5234dupC||||||,TC|intron_variant|MODIFIER|LGALS8|ENSG00000116977|transcript|ENST00000481485|protein_coding|3/4|c.46-5234dupC||||||WARNING_TRANSCRIPT_INCOMPLETE,TC|intron_variant|MODIFIER|LGALS8|ENSG00000116977|transcript|ENST00000454943|protein_coding|4/7|c.46-5234dupC||||||WARNING_TRANSCRIPT_INCOMPLETE,TC|intron_variant|MODIFIER|LGALS8|ENSG00000116977|transcript|ENST00000527974|protein_coding|2/10|c.46-5234dupC||||||,TC|intron_variant|MODIFIER|LGALS8|ENSG00000116977|transcript|ENST00000430527|protein_coding|3/5|c.46-5234dupC||||||WARNING_TRANSCRIPT_INCOMPLETE,TC|intron_variant|MODIFIER|LGALS8|ENSG00000116977|transcript|ENST00000406509|protein_coding|5/10|c.46-5234dupC||||||WARNING_TRANSCRIPT_INCOMPLETE,TC|intron_variant|MODIFIER|LGALS8|ENSG00000116977|transcript|ENST00000526589|protein_coding|5/13|c.46-5234dupC||||||,TC|intron_variant|MODIFIER|LGALS8|ENSG00000116977|transcript|ENST00000529489|protein_coding|3/3|c.46-5234dupC||||||WARNING_TRANSCRIPT_NO_STOP_CODON,TC|intron_variant|MODIFIER|LGALS8|ENSG00000116977|transcript|ENST00000341872|protein_coding|3/10|c.46-5234dupC||||||,TC|intron_variant|MODIFIER|LGALS8|ENSG00000116977|transcript|ENST00000450372|protein_coding|3/11|c.46-5234dupC||||||,TC|intron_variant|MODIFIER|LGALS8|ENSG00000116977|transcript|ENST00000366584|protein_coding|2/9|c.46-5234dupC||||||,TC|intron_variant|MODIFIER|LGALS8|ENSG00000116977|transcript|ENST00000238181|protein_coding|2/6|c.46-5234dupC||||||WARNING_TRANSCRIPT_INCOMPLETE,TC|intron_variant|MODIFIER|LGALS8|ENSG00000116977|transcript|ENST00000532826|retained_intron|2/3|n.226-5234dupC||||||,TC|intron_variant|MODIFIER|LGALS8|ENSG00000116977|transcript|ENST00000528782|retained_intron|2/4|n.226-5234dupC||||||,TC|intron_variant|MODIFIER|LGALS8|ENSG00000116977|transcript|ENST00000366583|retained_intron|2/6|n.222-5234dupC||||||,TC|intron_variant|MODIFIER|LGALS8|ENSG00000116977|transcript|ENST00000442397|nonsense_mediated_decay|2/4|c.46-5234dupC||||||,TC|intron_variant|MODIFIER|LGALS8|ENSG00000116977|transcript|ENST00000434231|nonsense_mediated_decay|2/4|c.45+6148dupC||||||,TC|intron_variant|MODIFIER|LGALS8|ENSG00000116977|transcript|ENST00000416919|protein_coding|2/8|c.46-5234dupC||||||,TC|intron_variant|MODIFIER|LGALS8|ENSG00000116977|transcript|ENST00000323938|protein_coding|2/9|c.46-5234dupC||||||,TC|intron_variant|MODIFIER|LGALS8|ENSG00000116977|transcript|ENST00000526634|protein_coding|2/9|c.46-5234dupC||||||,TC|intron_variant|MODIFIER|LGALS8|ENSG00000116977|transcript|ENST00000525042|protein_coding|1/7|c.46-5234dupC|||||| |
| RP11-385F5.5 | rs56333540 | 37 | 1 | 236695573 | 236695573 | + | SNP | T | T | C | SRR8586410 | Nonsense_Mutation | 0/1:74,32:106:99:1038,0,4127 | 106;ANN=C|downstream_gene_variant|MODIFIER|RP11-385F5.5|ENSG00000273058|transcript|ENST00000608547|antisense||n.*3900A>G|||||3900|,C|intron_variant|MODIFIER|LGALS8|ENSG00000116977|transcript|ENST00000352231|protein_coding|3/11|c.46-5224T>C||||||,C|intron_variant|MODIFIER|LGALS8|ENSG00000116977|transcript|ENST00000481485|protein_coding|3/4|c.46-5224T>C||||||WARNING_TRANSCRIPT_INCOMPLETE,C|intron_variant|MODIFIER|LGALS8|ENSG00000116977|transcript|ENST00000454943|protein_coding|4/7|c.46-5224T>C||||||WARNING_TRANSCRIPT_INCOMPLETE,C|intron_variant|MODIFIER|LGALS8|ENSG00000116977|transcript|ENST00000527974|protein_coding|2/10|c.46-5224T>C||||||,C|intron_variant|MODIFIER|LGALS8|ENSG00000116977|transcript|ENST00000430527|protein_coding|3/5|c.46-5224T>C||||||WARNING_TRANSCRIPT_INCOMPLETE,C|intron_variant|MODIFIER|LGALS8|ENSG00000116977|transcript|ENST00000406509|protein_coding|5/10|c.46-5224T>C||||||WARNING_TRANSCRIPT_INCOMPLETE,C|intron_variant|MODIFIER|LGALS8|ENSG00000116977|transcript|ENST00000526589|protein_coding|5/13|c.46-5224T>C||||||,C|intron_variant|MODIFIER|LGALS8|ENSG00000116977|transcript|ENST00000529489|protein_coding|3/3|c.46-5224T>C||||||WARNING_TRANSCRIPT_NO_STOP_CODON,C|intron_variant|MODIFIER|LGALS8|ENSG00000116977|transcript|ENST00000341872|protein_coding|3/10|c.46-5224T>C||||||,C|intron_variant|MODIFIER|LGALS8|ENSG00000116977|transcript|ENST00000450372|protein_coding|3/11|c.46-5224T>C||||||,C|intron_variant|MODIFIER|LGALS8|ENSG00000116977|transcript|ENST00000366584|protein_coding|2/9|c.46-5224T>C||||||,C|intron_variant|MODIFIER|LGALS8|ENSG00000116977|transcript|ENST00000238181|protein_coding|2/6|c.46-5224T>C||||||WARNING_TRANSCRIPT_INCOMPLETE,C|intron_variant|MODIFIER|LGALS8|ENSG00000116977|transcript|ENST00000532826|retained_intron|2/3|n.226-5224T>C||||||,C|intron_variant|MODIFIER|LGALS8|ENSG00000116977|transcript|ENST00000528782|retained_intron|2/4|n.226-5224T>C||||||,C|intron_variant|MODIFIER|LGALS8|ENSG00000116977|transcript|ENST00000366583|retained_intron|2/6|n.222-5224T>C||||||,C|intron_variant|MODIFIER|LGALS8|ENSG00000116977|transcript|ENST00000442397|nonsense_mediated_decay|2/4|c.46-5224T>C||||||,C|intron_variant|MODIFIER|LGALS8|ENSG00000116977|transcript|ENST00000434231|nonsense_mediated_decay|2/4|c.45+6158T>C||||||,C|intron_variant|MODIFIER|LGALS8|ENSG00000116977|transcript|ENST00000416919|protein_coding|2/8|c.46-5224T>C||||||,C|intron_variant|MODIFIER|LGALS8|ENSG00000116977|transcript|ENST00000323938|protein_coding|2/9|c.46-5224T>C||||||,C|intron_variant|MODIFIER|LGALS8|ENSG00000116977|transcript|ENST00000526634|protein_coding|2/9|c.46-5224T>C||||||,C|intron_variant|MODIFIER|LGALS8|ENSG00000116977|transcript|ENST00000525042|protein_coding|1/7|c.46-5224T>C|||||| |
| HEATR1 | rs754174970 | 37 | 1 | 236718012 | 236718012 | + | SNP | T | T | C | SRR8586410 | Missense_Mutation | 0/1:237,323:560:99:6496,0,9122 | 580;ANN=C|missense_variant|MODERATE|HEATR1|ENSG00000119285|transcript|ENST00000366582|protein_coding|42/45|c.5964A>G|p.Cys1988Trp|6079/8447|5964/6435|1988/2144||WARNING_REF_DOES_NOT_MATCH_GENOME,C|missense_variant|MODERATE|HEATR1|ENSG00000119285|transcript|ENST00000366581|protein_coding|41/44|c.5721A>G|p.Cys1907Trp|5836/6538|5721/6192|1907/2063||WARNING_REF_DOES_NOT_MATCH_GENOME,C|upstream_gene_variant|MODIFIER|RP11-385F5.4|ENSG00000230325|transcript|ENST00000433131|antisense||n.-4432A>G|||||4432|,C|downstream_gene_variant|MODIFIER|LGALS8|ENSG00000116977|transcript|ENST00000526589|protein_coding||c.*6551T>C|||||1731|,C|downstream_gene_variant|MODIFIER|LGALS8|ENSG00000116977|transcript|ENST00000366584|protein_coding||c.*6551T>C|||||3702| |
| MTR-RPL35P1 | rs58648144 | 37 | 1 | 237075366 | 237075366 | + | SNP | C | C | G | SRR8586410 | Missense_Mutation | 0/1:138,81:219:99:1846,0,3224 | 221;ANN=G|intergenic_region|MODIFIER|MTR-RPL35P1|ENSG00000116984-ENSG00000237991|intergenic_region|ENSG00000116984-ENSG00000237991|||n.237075366C>G|||||| |
| MTR-RPL35P1 | rs75035402 | 37 | 1 | 237078806 | 237078806 | + | SNP | T | T | C | SRR8586410 | Missense_Mutation | 0/1:169,179:348:99:3921,0,4568 | 385;ANN=C|intergenic_region|MODIFIER|MTR-RPL35P1|ENSG00000116984-ENSG00000237991|intergenic_region|ENSG00000116984-ENSG00000237991|||n.237078806T>C|||||| |
| MTR-RPL35P1 | rs56102712 | 37 | 1 | 237080603 | 237080603 | + | SNP | C | C | T | SRR8586410 | Missense_Mutation | 0/1:284,26:310:99:275,0,15412 | 320;ANN=T|intergenic_region|MODIFIER|MTR-RPL35P1|ENSG00000116984-ENSG00000237991|intergenic_region|ENSG00000116984-ENSG00000237991|||n.237080603C>T|||||| |
| MTR-RPL35P1 | rs56114377 | 37 | 1 | 237080608 | 237080608 | + | SNP | G | G | A | SRR8586410 | Missense_Mutation | 0/1:264,31:295:99:451,0,15146 | 302;ANN=A|intergenic_region|MODIFIER|MTR-RPL35P1|ENSG00000116984-ENSG00000237991|intergenic_region|ENSG00000116984-ENSG00000237991|||n.237080608G>A|||||| |
| MTR-RPL35P1 | rs6686489 | 37 | 1 | 237091429 | 237091429 | + | SNP | G | G | T | SRR8586410 | Missense_Mutation | 0/1:39,99:138:99:2110,0,859 | 180;ANN=T|intergenic_region|MODIFIER|MTR-RPL35P1|ENSG00000116984-ENSG00000237991|intergenic_region|ENSG00000116984-ENSG00000237991|||n.237091429G>T|||||| |
| MTR-RPL35P1 | rs6686490 | 37 | 1 | 237091430 | 237091430 | + | SNP | G | G | T | SRR8586410 | Missense_Mutation | 0/1:29,102:131:99:2297,0,620 | 177;ANN=T|intergenic_region|MODIFIER|MTR-RPL35P1|ENSG00000116984-ENSG00000237991|intergenic_region|ENSG00000116984-ENSG00000237991|||n.237091430G>T|||||| |
| MTR-RPL35P1 | rs368474001 | 37 | 1 | 237091437 | 237091437 | + | SNP | C | C | G | SRR8586410 | Missense_Mutation | 0/1:107,21:128:99:124,0,2729 | 131;ANN=G|intergenic_region|MODIFIER|MTR-RPL35P1|ENSG00000116984-ENSG00000237991|intergenic_region|ENSG00000116984-ENSG00000237991|||n.237091437C>G|||||| |
| LIPA | rs1439093 | 37 | 10 | 90974560 | 90974560 | + | SNP | T | T | C | SRR8586410 | Nonstop_Mutation | 0/1:103,31:134:99:546,0,2865 | 134;ANN=C|3_prime_UTR_variant|MODIFIER|LIPA|ENSG00000107798|transcript|ENST00000336233|protein_coding|10/10|c.*25A>G|||||25|WARNING_REF_DOES_NOT_MATCH_GENOME,C|3_prime_UTR_variant|MODIFIER|LIPA|ENSG00000107798|transcript|ENST00000371837|protein_coding|9/9|c.*25A>G|||||25|WARNING_REF_DOES_NOT_MATCH_GENOME,C|3_prime_UTR_variant|MODIFIER|LIPA|ENSG00000107798|transcript|ENST00000456827|protein_coding|10/10|c.*25A>G|||||25|WARNING_REF_DOES_NOT_MATCH_GENOME |
| MAPKAPK5P1-RN7SKP278 | rs767193506 | 37 | 10 | 110648714 | 110648714 | + | INS | A | A | AG | SRR8586410 | Frame_Shift_Ins | 0/1:135,74:209:99:1475,0,3129 | 224;ANN=AG|intergenic_region|MODIFIER|MAPKAPK5P1-RN7SKP278|ENSG00000224498-ENSG00000222436|intergenic_region|ENSG00000224498-ENSG00000222436|||n.110648714_110648715insG|||||| |
| POLR2G | rs188468517 | 37 | 11 | 62527604 | 62527604 | + | SNP | G | G | A | SRR8586410 | Missense_Mutation | 0/1:1141,137:1278:99:2251,0,47765 | 1362;ANN=A|upstream_gene_variant|MODIFIER|POLR2G|ENSG00000168002|transcript|ENST00000301788|protein_coding||c.-1517G>A|||||1412|,A|upstream_gene_variant|MODIFIER|POLR2G|ENSG00000168002|transcript|ENST00000533442|protein_coding||c.-5077G>A|||||1433|WARNING_TRANSCRIPT_INCOMPLETE,A|upstream_gene_variant|MODIFIER|POLR2G|ENSG00000168002|transcript|ENST00000525455|nonsense_mediated_decay||c.-1517G>A|||||1448|,A|upstream_gene_variant|MODIFIER|POLR2G|ENSG00000168002|transcript|ENST00000524819|nonsense_mediated_decay||c.-1517G>A|||||1451|,A|upstream_gene_variant|MODIFIER|POLR2G|ENSG00000168002|transcript|ENST00000531944|nonsense_mediated_decay||c.-1517G>A|||||1485|,A|upstream_gene_variant|MODIFIER|POLR2G|ENSG00000168002|transcript|ENST00000527435|retained_intron||n.-1505G>A|||||1505|,A|upstream_gene_variant|MODIFIER|POLR2G|ENSG00000168002|transcript|ENST00000531996|nonsense_mediated_decay||c.-1663G>A|||||1663|WARNING_TRANSCRIPT_NO_START_CODON,A|upstream_gene_variant|MODIFIER|POLR2G|ENSG00000168002|transcript|ENST00000526368|retained_intron||n.-2622G>A|||||2622|,A|intergenic_region|MODIFIER|ZBTB3-POLR2G|ENSG00000185670-ENSG00000168002|intergenic_region|ENSG00000185670-ENSG00000168002|||n.62527604G>A|||||| |
| RP11-681H10.1-RP11-693N9.1 | rs376479171 | 37 | 11 | 104599786 | 104599786 | + | SNP | G | G | A | SRR8586410 | Missense_Mutation | 0/1:11,7:18:79:79,0,357 | 18;ANN=A|intergenic_region|MODIFIER|RP11-681H10.1-RP11-693N9.1|ENSG00000270449-ENSG00000254569|intergenic_region|ENSG00000270449-ENSG00000254569|||n.104599786G>A|||||| |
| TSPAN9 | rs58837502 | 37 | 12 | 3211762 | 3211762 | + | SNP | T | T | C | SRR8586410 | Missense_Mutation | 0/1:61,117:178:99:3374,0,1416 | 181;ANN=C|intron_variant|MODIFIER|TSPAN9|ENSG00000011105|transcript|ENST00000537971|protein_coding|1/7|c.-18+25143T>C||||||,C|intron_variant|MODIFIER|TSPAN9|ENSG00000011105|transcript|ENST00000444315|nonsense_mediated_decay|2/5|c.-18+18877T>C||||||,C|intron_variant|MODIFIER|TSPAN9|ENSG00000011105|transcript|ENST00000011898|protein_coding|2/8|c.-18+18877T>C|||||| |
| ADCY6 | rs754280693 | 37 | 12 | 49185703 | 49185703 | + | SNP | A | A | G | SRR8586410 | Missense_Mutation | 0/1:363,42:405:99:540,0,18959 | 409;ANN=G|upstream_gene_variant|MODIFIER|ADCY6|ENSG00000174233|transcript|ENST00000550422|protein_coding||c.-8486T>C|||||2883|,G|downstream_gene_variant|MODIFIER|RP11-579D7.4|ENSG00000257660|transcript|ENST00000549864|lincRNA||n.*1385A>G|||||1385|,G|downstream_gene_variant|MODIFIER|RP11-579D7.4|ENSG00000257660|transcript|ENST00000547774|lincRNA||n.*1385A>G|||||1385|,G|intergenic_region|MODIFIER|RP11-579D7.4-RP11-579D7.8|ENSG00000257660-ENSG00000271547|intergenic_region|ENSG00000257660-ENSG00000271547|||n.49185703A>G|||||| |
| ADCY6 | rs146680997 | 37 | 12 | 49185709 | 49185709 | + | SNP | A | A | G | SRR8586410 | Missense_Mutation | 0/1:391,42:433:99:588,0,18235 | 437;ANN=G|upstream_gene_variant|MODIFIER|ADCY6|ENSG00000174233|transcript|ENST00000550422|protein_coding||c.-8492T>C|||||2889|,G|downstream_gene_variant|MODIFIER|RP11-579D7.4|ENSG00000257660|transcript|ENST00000549864|lincRNA||n.*1391A>G|||||1391|,G|downstream_gene_variant|MODIFIER|RP11-579D7.4|ENSG00000257660|transcript|ENST00000547774|lincRNA||n.*1391A>G|||||1391|,G|intergenic_region|MODIFIER|RP11-579D7.4-RP11-579D7.8|ENSG00000257660-ENSG00000271547|intergenic_region|ENSG00000257660-ENSG00000271547|||n.49185709A>G|||||| |
| SNORD116-LINC00332 | rs1413482 | 37 | 13 | 40644314 | 40644314 | + | SNP | C | C | T | SRR8586410 | Missense_Mutation | 0/1:10,9:19:99:226,0,311 | 19;ANN=T|intergenic_region|MODIFIER|SNORD116-LINC00332|ENSG00000212553-ENSG00000230710|intergenic_region|ENSG00000212553-ENSG00000230710|||n.40644314C>T|||||| |
| PRMT5-AS1 | rs753335327 | 37 | 14 | 23384640 | 23384640 | + | SNP | C | C | T | SRR8586410 | Missense_Mutation | 0/1:349,225:574:99:5710,0,17581 | 574;ANN=T|upstream_gene_variant|MODIFIER|PRMT5-AS1|ENSG00000237054|transcript|ENST00000424245|antisense||n.-4025C>T|||||4025|,T|upstream_gene_variant|MODIFIER|RBM23|ENSG00000100461|transcript|ENST00000555691|protein_coding||c.-4038G>A|||||3639|WARNING_TRANSCRIPT_INCOMPLETE,T|upstream_gene_variant|MODIFIER|PRMT5-AS1|ENSG00000237054|transcript|ENST00000609885|antisense||n.-4190C>T|||||4190|,T|downstream_gene_variant|MODIFIER|RBM23|ENSG00000100461|transcript|ENST00000553777|processed_transcript||n.*2133G>A|||||2133|,T|intron_variant|MODIFIER|RBM23|ENSG00000100461|transcript|ENST00000359890|protein_coding|1/13|c.-11+3568G>A||||||,T|intron_variant|MODIFIER|RBM23|ENSG00000100461|transcript|ENST00000555209|protein_coding|1/10|c.-493+3568G>A||||||,T|intron_variant|MODIFIER|RBM23|ENSG00000100461|transcript|ENST00000557667|retained_intron|1/13|n.138+3568G>A||||||,T|intron_variant|MODIFIER|RBM23|ENSG00000100461|transcript|ENST00000399922|protein_coding|1/12|c.-11+3568G>A||||||,T|intron_variant|MODIFIER|RBM23|ENSG00000100461|transcript|ENST00000346528|protein_coding|1/11|c.-11+3568G>A||||||,T|intron_variant|MODIFIER|RBM23|ENSG00000100461|transcript|ENST00000542016|protein_coding|1/11|c.-360+3568G>A||||||,T|intron_variant|MODIFIER|RBM23|ENSG00000100461|transcript|ENST00000307814|retained_intron|1/10|n.151+3568G>A||||||,T|intron_variant|MODIFIER|RBM23|ENSG00000100461|transcript|ENST00000557403|protein_coding|1/5|c.-343+3568G>A||||||WARNING_TRANSCRIPT_INCOMPLETE,T|intron_variant|MODIFIER|RBM23|ENSG00000100461|transcript|ENST00000555722|protein_coding|1/4|c.-284+3568G>A||||||WARNING_TRANSCRIPT_INCOMPLETE,T|intron_variant|MODIFIER|RBM23|ENSG00000100461|transcript|ENST00000553920|processed_transcript|1/4|n.138+3568G>A||||||,T|intron_variant|MODIFIER|RBM23|ENSG00000100461|transcript|ENST00000557227|protein_coding|1/3|c.-306+3568G>A||||||WARNING_TRANSCRIPT_NO_STOP_CODON,T|intron_variant|MODIFIER|RBM23|ENSG00000100461|transcript|ENST00000555714|processed_transcript|1/5|n.151+3568G>A||||||,T|intron_variant|MODIFIER|RBM23|ENSG00000100461|transcript|ENST00000554256|protein_coding|1/5|c.-11+3568G>A||||||WARNING_TRANSCRIPT_NO_STOP_CODON,T|intron_variant|MODIFIER|RBM23|ENSG00000100461|transcript|ENST00000556687|retained_intron|1/3|n.151+3568G>A||||||,T|intron_variant|MODIFIER|RBM23|ENSG00000100461|transcript|ENST00000553902|retained_intron|1/2|n.151+3568G>A||||||,T|intron_variant|MODIFIER|RBM23|ENSG00000100461|transcript|ENST00000554955|retained_intron|1/4|n.112+3568G>A||||||,T|intron_variant|MODIFIER|RBM23|ENSG00000100461|transcript|ENST00000556984|processed_transcript|1/4|n.151+3568G>A||||||,T|intron_variant|MODIFIER|RBM23|ENSG00000100461|transcript|ENST00000557549|protein_coding|1/4|c.-128-3528G>A||||||WARNING_TRANSCRIPT_NO_STOP_CODON,T|intron_variant|MODIFIER|RBM23|ENSG00000100461|transcript|ENST00000555676|protein_coding|1/4|c.-132-1586G>A||||||WARNING_TRANSCRIPT_NO_STOP_CODON,T|intron_variant|MODIFIER|RBM23|ENSG00000100461|transcript|ENST00000557571|protein_coding|2/4|c.-11+1597G>A||||||WARNING_TRANSCRIPT_NO_STOP_CODON,T|intron_variant|MODIFIER|RBM23|ENSG00000100461|transcript|ENST00000556862|protein_coding|1/5|c.-132-1586G>A||||||WARNING_TRANSCRIPT_INCOMPLETE,T|intron_variant|MODIFIER|RBM23|ENSG00000100461|transcript|ENST00000557464|protein_coding|1/4|c.-108-1610G>A||||||WARNING_TRANSCRIPT_NO_STOP_CODON,T|intron_variant|MODIFIER|RBM23|ENSG00000100461|transcript|ENST00000554618|protein_coding|1/5|c.-210-1415G>A||||||WARNING_TRANSCRIPT_INCOMPLETE,T|intron_variant|MODIFIER|RBM23|ENSG00000100461|transcript|ENST00000556365|processed_transcript|1/3|n.151+3568G>A||||||,T|intron_variant|MODIFIER|RBM23|ENSG00000100461|transcript|ENST00000553876|protein_coding|1/2|c.-11+2048G>A||||||WARNING_TRANSCRIPT_NO_STOP_CODON |
| PRMT5-AS1 | rs370121893 | 37 | 14 | 23386023 | 23386023 | + | SNP | G | G | A | SRR8586410 | Missense_Mutation | 0/1:232,32:264:99:495,0,7106 | 265;ANN=A|upstream_gene_variant|MODIFIER|PRMT5-AS1|ENSG00000237054|transcript|ENST00000424245|antisense||n.-2642G>A|||||2642|,A|upstream_gene_variant|MODIFIER|PRMT5-AS1|ENSG00000237054|transcript|ENST00000609885|antisense||n.-2807G>A|||||2807|,A|upstream_gene_variant|MODIFIER|PRMT5-AS1|ENSG00000237054|transcript|ENST00000457443|antisense||n.-3825G>A|||||3825|,A|upstream_gene_variant|MODIFIER|PRMT5-AS1|ENSG00000237054|transcript|ENST00000599580|antisense||n.-4097G>A|||||4097|,A|upstream_gene_variant|MODIFIER|PRMT5-AS1|ENSG00000237054|transcript|ENST00000595662|antisense||n.-4224G>A|||||4224|,A|upstream_gene_variant|MODIFIER|PRMT5-AS1|ENSG00000237054|transcript|ENST00000587245|antisense||n.-4224G>A|||||4224|,A|upstream_gene_variant|MODIFIER|PRMT5-AS1|ENSG00000237054|transcript|ENST00000590290|antisense||n.-4227G>A|||||4227|,A|downstream_gene_variant|MODIFIER|PRMT5|ENSG00000100462|transcript|ENST00000324366|protein_coding||c.*4090C>T|||||3697|,A|downstream_gene_variant|MODIFIER|RBM23|ENSG00000100461|transcript|ENST00000553777|processed_transcript||n.*750C>T|||||750|,A|downstream_gene_variant|MODIFIER|PRMT5|ENSG00000100462|transcript|ENST00000397441|protein_coding||c.*4090C>T|||||3711|,A|downstream_gene_variant|MODIFIER|PRMT5|ENSG00000100462|transcript|ENST00000557443|protein_coding||c.*3867C>T|||||3715|WARNING_TRANSCRIPT_NO_START_CODON,A|downstream_gene_variant|MODIFIER|PRMT5|ENSG00000100462|transcript|ENST00000476175|processed_transcript||n.*3715C>T|||||3715|,A|downstream_gene_variant|MODIFIER|PRMT5|ENSG00000100462|transcript|ENST00000397440|protein_coding||c.*4090C>T|||||3715|,A|downstream_gene_variant|MODIFIER|PRMT5|ENSG00000100462|transcript|ENST00000216350|protein_coding||c.*4090C>T|||||3716|,A|downstream_gene_variant|MODIFIER|PRMT5|ENSG00000100462|transcript|ENST00000555454|protein_coding||c.*4233C>T|||||3996|WARNING_TRANSCRIPT_NO_START_CODON,A|downstream_gene_variant|MODIFIER|PRMT5|ENSG00000100462|transcript|ENST00000454731|protein_coding||c.*4090C>T|||||4011|WARNING_TRANSCRIPT_NO_START_CODON,A|downstream_gene_variant|MODIFIER|PRMT5|ENSG00000100462|transcript|ENST00000553915|nonsense_mediated_decay||c.*10782C>T|||||4043|,A|downstream_gene_variant|MODIFIER|PRMT5|ENSG00000100462|transcript|ENST00000538452|protein_coding||c.*4090C>T|||||4043|,A|downstream_gene_variant|MODIFIER|PRMT5|ENSG00000100462|transcript|ENST00000553897|protein_coding||c.*4090C>T|||||4062|,A|intron_variant|MODIFIER|RBM23|ENSG00000100461|transcript|ENST00000359890|protein_coding|1/13|c.-11+2185C>T||||||,A|intron_variant|MODIFIER|RBM23|ENSG00000100461|transcript|ENST00000555209|protein_coding|1/10|c.-493+2185C>T||||||,A|intron_variant|MODIFIER|RBM23|ENSG00000100461|transcript|ENST00000557667|retained_intron|1/13|n.138+2185C>T||||||,A|intron_variant|MODIFIER|RBM23|ENSG00000100461|transcript|ENST00000399922|protein_coding|1/12|c.-11+2185C>T||||||,A|intron_variant|MODIFIER|RBM23|ENSG00000100461|transcript|ENST00000346528|protein_coding|1/11|c.-11+2185C>T||||||,A|intron_variant|MODIFIER|RBM23|ENSG00000100461|transcript|ENST00000542016|protein_coding|1/11|c.-360+2185C>T||||||,A|intron_variant|MODIFIER|RBM23|ENSG00000100461|transcript|ENST00000307814|retained_intron|1/10|n.151+2185C>T||||||,A|intron_variant|MODIFIER|RBM23|ENSG00000100461|transcript|ENST00000557403|protein_coding|1/5|c.-343+2185C>T||||||WARNING_TRANSCRIPT_INCOMPLETE,A|intron_variant|MODIFIER|RBM23|ENSG00000100461|transcript|ENST00000555722|protein_coding|1/4|c.-284+2185C>T||||||WARNING_TRANSCRIPT_INCOMPLETE,A|intron_variant|MODIFIER|RBM23|ENSG00000100461|transcript|ENST00000553920|processed_transcript|1/4|n.138+2185C>T||||||,A|intron_variant|MODIFIER|RBM23|ENSG00000100461|transcript|ENST00000557227|protein_coding|1/3|c.-306+2185C>T||||||WARNING_TRANSCRIPT_NO_STOP_CODON,A|intron_variant|MODIFIER|RBM23|ENSG00000100461|transcript|ENST00000555714|processed_transcript|1/5|n.151+2185C>T||||||,A|intron_variant|MODIFIER|RBM23|ENSG00000100461|transcript|ENST00000554256|protein_coding|1/5|c.-11+2185C>T||||||WARNING_TRANSCRIPT_NO_STOP_CODON,A|intron_variant|MODIFIER|RBM23|ENSG00000100461|transcript|ENST00000556687|retained_intron|1/3|n.151+2185C>T||||||,A|intron_variant|MODIFIER|RBM23|ENSG00000100461|transcript|ENST00000553902|retained_intron|1/2|n.151+2185C>T||||||,A|intron_variant|MODIFIER|RBM23|ENSG00000100461|transcript|ENST00000554955|retained_intron|1/4|n.112+2185C>T||||||,A|intron_variant|MODIFIER|RBM23|ENSG00000100461|transcript|ENST00000556984|processed_transcript|1/4|n.151+2185C>T||||||,A|intron_variant|MODIFIER|RBM23|ENSG00000100461|transcript|ENST00000557549|protein_coding|1/4|c.-129+2185C>T||||||WARNING_TRANSCRIPT_NO_STOP_CODON,A|intron_variant|MODIFIER|RBM23|ENSG00000100461|transcript|ENST00000555676|protein_coding|1/4|c.-133+2185C>T||||||WARNING_TRANSCRIPT_NO_STOP_CODON,A|intron_variant|MODIFIER|RBM23|ENSG00000100461|transcript|ENST00000557571|protein_coding|2/4|c.-11+214C>T||||||WARNING_TRANSCRIPT_NO_STOP_CODON,A|intron_variant|MODIFIER|RBM23|ENSG00000100461|transcript|ENST00000556862|protein_coding|1/5|c.-133+2185C>T||||||WARNING_TRANSCRIPT_INCOMPLETE,A|intron_variant|MODIFIER|RBM23|ENSG00000100461|transcript|ENST00000557464|protein_coding|1/4|c.-109+2185C>T||||||WARNING_TRANSCRIPT_NO_STOP_CODON,A|intron_variant|MODIFIER|RBM23|ENSG00000100461|transcript|ENST00000554618|protein_coding|1/5|c.-211+2185C>T||||||WARNING_TRANSCRIPT_INCOMPLETE,A|intron_variant|MODIFIER|RBM23|ENSG00000100461|transcript|ENST00000556365|processed_transcript|1/3|n.151+2185C>T||||||,A|intron_variant|MODIFIER|RBM23|ENSG00000100461|transcript|ENST00000553876|protein_coding|1/2|c.-11+665C>T||||||WARNING_TRANSCRIPT_NO_STOP_CODON |
| PRMT5-AS1 | rs45574136 | 37 | 14 | 23386496 | 23386496 | + | SNP | T | T | A | SRR8586410 | Missense_Mutation | 0/1:385,312:697:99:9327,0,14260 | 697;ANN=A|upstream_gene_variant|MODIFIER|PRMT5-AS1|ENSG00000237054|transcript|ENST00000424245|antisense||n.-2169T>A|||||2169|,A|upstream_gene_variant|MODIFIER|PRMT5-AS1|ENSG00000237054|transcript|ENST00000609885|antisense||n.-2334T>A|||||2334|,A|upstream_gene_variant|MODIFIER|PRMT5-AS1|ENSG00000237054|transcript|ENST00000457443|antisense||n.-3352T>A|||||3352|,A|upstream_gene_variant|MODIFIER|PRMT5-AS1|ENSG00000237054|transcript|ENST00000599580|antisense||n.-3624T>A|||||3624|,A|upstream_gene_variant|MODIFIER|PRMT5-AS1|ENSG00000237054|transcript|ENST00000595662|antisense||n.-3751T>A|||||3751|,A|upstream_gene_variant|MODIFIER|PRMT5-AS1|ENSG00000237054|transcript|ENST00000587245|antisense||n.-3751T>A|||||3751|,A|upstream_gene_variant|MODIFIER|PRMT5-AS1|ENSG00000237054|transcript|ENST00000590290|antisense||n.-3754T>A|||||3754|,A|downstream_gene_variant|MODIFIER|PRMT5|ENSG00000100462|transcript|ENST00000324366|protein_coding||c.*3617A>T|||||3224|,A|downstream_gene_variant|MODIFIER|RBM23|ENSG00000100461|transcript|ENST00000553777|processed_transcript||n.*277A>T|||||277|,A|downstream_gene_variant|MODIFIER|PRMT5|ENSG00000100462|transcript|ENST00000397441|protein_coding||c.*3617A>T|||||3238|,A|downstream_gene_variant|MODIFIER|PRMT5|ENSG00000100462|transcript|ENST00000557443|protein_coding||c.*3394A>T|||||3242|WARNING_TRANSCRIPT_NO_START_CODON,A|downstream_gene_variant|MODIFIER|PRMT5|ENSG00000100462|transcript|ENST00000476175|processed_transcript||n.*3242A>T|||||3242|,A|downstream_gene_variant|MODIFIER|PRMT5|ENSG00000100462|transcript|ENST00000397440|protein_coding||c.*3617A>T|||||3242|,A|downstream_gene_variant|MODIFIER|PRMT5|ENSG00000100462|transcript|ENST00000216350|protein_coding||c.*3617A>T|||||3243|,A|downstream_gene_variant|MODIFIER|PRMT5|ENSG00000100462|transcript|ENST00000555454|protein_coding||c.*3760A>T|||||3523|WARNING_TRANSCRIPT_NO_START_CODON,A|downstream_gene_variant|MODIFIER|PRMT5|ENSG00000100462|transcript|ENST00000454731|protein_coding||c.*3617A>T|||||3538|WARNING_TRANSCRIPT_NO_START_CODON,A|downstream_gene_variant|MODIFIER|PRMT5|ENSG00000100462|transcript|ENST00000553915|nonsense_mediated_decay||c.*10309A>T|||||3570|,A|downstream_gene_variant|MODIFIER|PRMT5|ENSG00000100462|transcript|ENST00000538452|protein_coding||c.*3617A>T|||||3570|,A|downstream_gene_variant|MODIFIER|PRMT5|ENSG00000100462|transcript|ENST00000553897|protein_coding||c.*3617A>T|||||3589|,A|downstream_gene_variant|MODIFIER|PRMT5|ENSG00000100462|transcript|ENST00000557758|retained_intron||n.*4845A>T|||||4845|,A|intron_variant|MODIFIER|RBM23|ENSG00000100461|transcript|ENST00000359890|protein_coding|1/13|c.-11+1712A>T||||||,A|intron_variant|MODIFIER|RBM23|ENSG00000100461|transcript|ENST00000555209|protein_coding|1/10|c.-493+1712A>T||||||,A|intron_variant|MODIFIER|RBM23|ENSG00000100461|transcript|ENST00000557667|retained_intron|1/13|n.138+1712A>T||||||,A|intron_variant|MODIFIER|RBM23|ENSG00000100461|transcript|ENST00000399922|protein_coding|1/12|c.-11+1712A>T||||||,A|intron_variant|MODIFIER|RBM23|ENSG00000100461|transcript|ENST00000346528|protein_coding|1/11|c.-11+1712A>T||||||,A|intron_variant|MODIFIER|RBM23|ENSG00000100461|transcript|ENST00000542016|protein_coding|1/11|c.-360+1712A>T||||||,A|intron_variant|MODIFIER|RBM23|ENSG00000100461|transcript|ENST00000307814|retained_intron|1/10|n.151+1712A>T||||||,A|intron_variant|MODIFIER|RBM23|ENSG00000100461|transcript|ENST00000557403|protein_coding|1/5|c.-343+1712A>T||||||WARNING_TRANSCRIPT_INCOMPLETE,A|intron_variant|MODIFIER|RBM23|ENSG00000100461|transcript|ENST00000555722|protein_coding|1/4|c.-284+1712A>T||||||WARNING_TRANSCRIPT_INCOMPLETE,A|intron_variant|MODIFIER|RBM23|ENSG00000100461|transcript|ENST00000553920|processed_transcript|1/4|n.138+1712A>T||||||,A|intron_variant|MODIFIER|RBM23|ENSG00000100461|transcript|ENST00000557227|protein_coding|1/3|c.-306+1712A>T||||||WARNING_TRANSCRIPT_NO_STOP_CODON,A|intron_variant|MODIFIER|RBM23|ENSG00000100461|transcript|ENST00000555714|processed_transcript|1/5|n.151+1712A>T||||||,A|intron_variant|MODIFIER|RBM23|ENSG00000100461|transcript|ENST00000554256|protein_coding|1/5|c.-11+1712A>T||||||WARNING_TRANSCRIPT_NO_STOP_CODON,A|intron_variant|MODIFIER|RBM23|ENSG00000100461|transcript|ENST00000556687|retained_intron|1/3|n.151+1712A>T||||||,A|intron_variant|MODIFIER|RBM23|ENSG00000100461|transcript|ENST00000553902|retained_intron|1/2|n.151+1712A>T||||||,A|intron_variant|MODIFIER|RBM23|ENSG00000100461|transcript|ENST00000554955|retained_intron|1/4|n.112+1712A>T||||||,A|intron_variant|MODIFIER|RBM23|ENSG00000100461|transcript|ENST00000556984|processed_transcript|1/4|n.151+1712A>T||||||,A|intron_variant|MODIFIER|RBM23|ENSG00000100461|transcript|ENST00000557549|protein_coding|1/4|c.-129+1712A>T||||||WARNING_TRANSCRIPT_NO_STOP_CODON,A|intron_variant|MODIFIER|RBM23|ENSG00000100461|transcript|ENST00000555676|protein_coding|1/4|c.-133+1712A>T||||||WARNING_TRANSCRIPT_NO_STOP_CODON,A|intron_variant|MODIFIER|RBM23|ENSG00000100461|transcript|ENST00000557571|protein_coding|1/4|c.-149-121A>T||||||WARNING_TRANSCRIPT_NO_STOP_CODON,A|intron_variant|MODIFIER|RBM23|ENSG00000100461|transcript|ENST00000556862|protein_coding|1/5|c.-133+1712A>T||||||WARNING_TRANSCRIPT_INCOMPLETE,A|intron_variant|MODIFIER|RBM23|ENSG00000100461|transcript|ENST00000557464|protein_coding|1/4|c.-109+1712A>T||||||WARNING_TRANSCRIPT_NO_STOP_CODON,A|intron_variant|MODIFIER|RBM23|ENSG00000100461|transcript|ENST00000554618|protein_coding|1/5|c.-211+1712A>T||||||WARNING_TRANSCRIPT_INCOMPLETE,A|intron_variant|MODIFIER|RBM23|ENSG00000100461|transcript|ENST00000556365|processed_transcript|1/3|n.151+1712A>T||||||,A|intron_variant|MODIFIER|RBM23|ENSG00000100461|transcript|ENST00000553876|protein_coding|1/2|c.-11+192A>T||||||WARNING_TRANSCRIPT_NO_STOP_CODON |
| RBM23 | rs780653498 | 37 | 14 | 23388975 | 23388975 | + | SNP | T | T | C | SRR8586410 | Missense_Mutation | 0/1:352,187:539:99:4579,0,13920 | 539;ANN=C|upstream_gene_variant|MODIFIER|RBM23|ENSG00000100461|transcript|ENST00000359890|protein_coding||c.-8373A>G|||||582|,C|upstream_gene_variant|MODIFIER|RBM23|ENSG00000100461|transcript|ENST00000555209|protein_coding||c.-14778A>G|||||637|,C|upstream_gene_variant|MODIFIER|RBM23|ENSG00000100461|transcript|ENST00000557667|retained_intron||n.-630A>G|||||630|,C|upstream_gene_variant|MODIFIER|RBM23|ENSG00000100461|transcript|ENST00000399922|protein_coding||c.-8373A>G|||||584|,C|upstream_gene_variant|MODIFIER|RBM23|ENSG00000100461|transcript|ENST00000346528|protein_coding||c.-8373A>G|||||588|,C|upstream_gene_variant|MODIFIER|RBM23|ENSG00000100461|transcript|ENST00000542016|protein_coding||c.-14368A>G|||||588|,C|upstream_gene_variant|MODIFIER|RBM23|ENSG00000100461|transcript|ENST00000307814|retained_intron||n.-617A>G|||||617|,C|upstream_gene_variant|MODIFIER|RBM23|ENSG00000100461|transcript|ENST00000557403|protein_coding||c.-14368A>G|||||628|WARNING_TRANSCRIPT_INCOMPLETE,C|upstream_gene_variant|MODIFIER|RBM23|ENSG00000100461|transcript|ENST00000555722|protein_coding||c.-14368A>G|||||601|WARNING_TRANSCRIPT_INCOMPLETE,C|upstream_gene_variant|MODIFIER|RBM23|ENSG00000100461|transcript|ENST00000553920|processed_transcript||n.-630A>G|||||630|,C|upstream_gene_variant|MODIFIER|RBM23|ENSG00000100461|transcript|ENST00000557227|protein_coding||c.-14368A>G|||||584|WARNING_TRANSCRIPT_NO_STOP_CODON,C|upstream_gene_variant|MODIFIER|RBM23|ENSG00000100461|transcript|ENST00000555714|processed_transcript||n.-617A>G|||||617|,C|upstream_gene_variant|MODIFIER|RBM23|ENSG00000100461|transcript|ENST00000554256|protein_coding||c.-8373A>G|||||629|WARNING_TRANSCRIPT_NO_STOP_CODON,C|upstream_gene_variant|MODIFIER|RBM23|ENSG00000100461|transcript|ENST00000556687|retained_intron||n.-617A>G|||||617|,C|upstream_gene_variant|MODIFIER|RBM23|ENSG00000100461|transcript|ENST00000553902|retained_intron||n.-617A>G|||||617|,C|upstream_gene_variant|MODIFIER|RBM23|ENSG00000100461|transcript|ENST00000554955|retained_intron||n.-656A>G|||||656|,C|upstream_gene_variant|MODIFIER|RBM23|ENSG00000100461|transcript|ENST00000556984|processed_transcript||n.-617A>G|||||617|,C|upstream_gene_variant|MODIFIER|RBM23|ENSG00000100461|transcript|ENST00000557549|protein_coding||c.-8373A>G|||||617|WARNING_TRANSCRIPT_NO_STOP_CODON,C|upstream_gene_variant|MODIFIER|RBM23|ENSG00000100461|transcript|ENST00000555676|protein_coding||c.-8373A>G|||||617|WARNING_TRANSCRIPT_NO_STOP_CODON,C|upstream_gene_variant|MODIFIER|RBM23|ENSG00000100461|transcript|ENST00000557571|protein_coding||c.-8373A>G|||||617|WARNING_TRANSCRIPT_NO_STOP_CODON,C|upstream_gene_variant|MODIFIER|RBM23|ENSG00000100461|transcript|ENST00000556862|protein_coding||c.-8373A>G|||||609|WARNING_TRANSCRIPT_INCOMPLETE,C|upstream_gene_variant|MODIFIER|RBM23|ENSG00000100461|transcript|ENST00000557464|protein_coding||c.-8373A>G|||||617|WARNING_TRANSCRIPT_NO_STOP_CODON,C|upstream_gene_variant|MODIFIER|RBM23|ENSG00000100461|transcript|ENST00000554618|protein_coding||c.-8373A>G|||||617|WARNING_TRANSCRIPT_INCOMPLETE,C|upstream_gene_variant|MODIFIER|RBM23|ENSG00000100461|transcript|ENST00000556365|processed_transcript||n.-617A>G|||||617|,C|upstream_gene_variant|MODIFIER|RBM23|ENSG00000100461|transcript|ENST00000553876|protein_coding||c.-8373A>G|||||2210|WARNING_TRANSCRIPT_NO_STOP_CODON,C|upstream_gene_variant|MODIFIER|RBM23|ENSG00000100461|transcript|ENST00000553777|processed_transcript||n.-679A>G|||||679|,C|upstream_gene_variant|MODIFIER|PRMT5-AS1|ENSG00000237054|transcript|ENST00000457443|antisense||n.-873T>C|||||873|,C|upstream_gene_variant|MODIFIER|PRMT5-AS1|ENSG00000237054|transcript|ENST00000599580|antisense||n.-1145T>C|||||1145|,C|upstream_gene_variant|MODIFIER|PRMT5-AS1|ENSG00000237054|transcript|ENST00000595662|antisense||n.-1272T>C|||||1272|,C|upstream_gene_variant|MODIFIER|PRMT5-AS1|ENSG00000237054|transcript|ENST00000587245|antisense||n.-1272T>C|||||1272|,C|upstream_gene_variant|MODIFIER|PRMT5-AS1|ENSG00000237054|transcript|ENST00000590290|antisense||n.-1275T>C|||||1275|,C|downstream_gene_variant|MODIFIER|PRMT5|ENSG00000100462|transcript|ENST00000324366|protein_coding||c.*1138A>G|||||745|,C|downstream_gene_variant|MODIFIER|PRMT5|ENSG00000100462|transcript|ENST00000397441|protein_coding||c.*1138A>G|||||759|,C|downstream_gene_variant|MODIFIER|PRMT5|ENSG00000100462|transcript|ENST00000557443|protein_coding||c.*915A>G|||||763|WARNING_TRANSCRIPT_NO_START_CODON,C|downstream_gene_variant|MODIFIER|PRMT5|ENSG00000100462|transcript|ENST00000476175|processed_transcript||n.*763A>G|||||763|,C|downstream_gene_variant|MODIFIER|PRMT5|ENSG00000100462|transcript|ENST00000397440|protein_coding||c.*1138A>G|||||763|,C|downstream_gene_variant|MODIFIER|PRMT5|ENSG00000100462|transcript|ENST00000216350|protein_coding||c.*1138A>G|||||764|,C|downstream_gene_variant|MODIFIER|PRMT5|ENSG00000100462|transcript|ENST00000555454|protein_coding||c.*1281A>G|||||1044|WARNING_TRANSCRIPT_NO_START_CODON,C|downstream_gene_variant|MODIFIER|PRMT5|ENSG00000100462|transcript|ENST00000454731|protein_coding||c.*1138A>G|||||1059|WARNING_TRANSCRIPT_NO_START_CODON,C|downstream_gene_variant|MODIFIER|PRMT5|ENSG00000100462|transcript|ENST00000553915|nonsense_mediated_decay||c.*7830A>G|||||1091|,C|downstream_gene_variant|MODIFIER|PRMT5|ENSG00000100462|transcript|ENST00000538452|protein_coding||c.*1138A>G|||||1091|,C|downstream_gene_variant|MODIFIER|PRMT5|ENSG00000100462|transcript|ENST00000553897|protein_coding||c.*1138A>G|||||1110|,C|downstream_gene_variant|MODIFIER|PRMT5|ENSG00000100462|transcript|ENST00000557758|retained_intron||n.*2366A>G|||||2366|,C|downstream_gene_variant|MODIFIER|PRMT5|ENSG00000100462|transcript|ENST00000553502|protein_coding||c.*4396A>G|||||4396|WARNING_TRANSCRIPT_NO_START_CODON,C|downstream_gene_variant|MODIFIER|PRMT5|ENSG00000100462|transcript|ENST00000555530|protein_coding||c.*4551A>G|||||4551|WARNING_TRANSCRIPT_NO_START_CODON,C|downstream_gene_variant|MODIFIER|PRMT5|ENSG00000100462|transcript|ENST00000556043|protein_coding||c.*4733A>G|||||4733|WARNING_TRANSCRIPT_NO_STOP_CODON,C|downstream_gene_variant|MODIFIER|PRMT5|ENSG00000100462|transcript|ENST00000553550|protein_coding||c.*4872A>G|||||4872|WARNING_TRANSCRIPT_NO_STOP_CODON,C|downstream_gene_variant|MODIFIER|PRMT5|ENSG00000100462|transcript|ENST00000554716|processed_transcript||n.*4897A>G|||||4897|,C|non_coding_transcript_exon_variant|MODIFIER|PRMT5-AS1|ENSG00000237054|transcript|ENST00000424245|antisense|1/2|n.311T>C||||||,C|non_coding_transcript_exon_variant|MODIFIER|PRMT5-AS1|ENSG00000237054|transcript|ENST00000609885|antisense|1/2|n.146T>C|||||| |
| RBM23 | rs750020902 | 37 | 14 | 23393008 | 23393008 | + | SNP | C | C | T | SRR8586410 | Missense_Mutation | 0/1:312,37:349:99:109,0,8683 | 349;ANN=T|upstream_gene_variant|MODIFIER|RBM23|ENSG00000100461|transcript|ENST00000359890|protein_coding||c.-12406G>A|||||4615|,T|upstream_gene_variant|MODIFIER|RBM23|ENSG00000100461|transcript|ENST00000555209|protein_coding||c.-18811G>A|||||4670|,T|upstream_gene_variant|MODIFIER|RBM23|ENSG00000100461|transcript|ENST00000557667|retained_intron||n.-4663G>A|||||4663|,T|upstream_gene_variant|MODIFIER|RBM23|ENSG00000100461|transcript|ENST00000399922|protein_coding||c.-12406G>A|||||4617|,T|upstream_gene_variant|MODIFIER|RBM23|ENSG00000100461|transcript|ENST00000346528|protein_coding||c.-12406G>A|||||4621|,T|upstream_gene_variant|MODIFIER|RBM23|ENSG00000100461|transcript|ENST00000542016|protein_coding||c.-18401G>A|||||4621|,T|upstream_gene_variant|MODIFIER|RBM23|ENSG00000100461|transcript|ENST00000307814|retained_intron||n.-4650G>A|||||4650|,T|upstream_gene_variant|MODIFIER|RBM23|ENSG00000100461|transcript|ENST00000557403|protein_coding||c.-18401G>A|||||4661|WARNING_TRANSCRIPT_INCOMPLETE,T|upstream_gene_variant|MODIFIER|RBM23|ENSG00000100461|transcript|ENST00000555722|protein_coding||c.-18401G>A|||||4634|WARNING_TRANSCRIPT_INCOMPLETE,T|upstream_gene_variant|MODIFIER|RBM23|ENSG00000100461|transcript|ENST00000553920|processed_transcript||n.-4663G>A|||||4663|,T|upstream_gene_variant|MODIFIER|RBM23|ENSG00000100461|transcript|ENST00000557227|protein_coding||c.-18401G>A|||||4617|WARNING_TRANSCRIPT_NO_STOP_CODON,T|upstream_gene_variant|MODIFIER|RBM23|ENSG00000100461|transcript|ENST00000555714|processed_transcript||n.-4650G>A|||||4650|,T|upstream_gene_variant|MODIFIER|RBM23|ENSG00000100461|transcript|ENST00000554256|protein_coding||c.-12406G>A|||||4662|WARNING_TRANSCRIPT_NO_STOP_CODON,T|upstream_gene_variant|MODIFIER|RBM23|ENSG00000100461|transcript|ENST00000556687|retained_intron||n.-4650G>A|||||4650|,T|upstream_gene_variant|MODIFIER|RBM23|ENSG00000100461|transcript|ENST00000553902|retained_intron||n.-4650G>A|||||4650|,T|upstream_gene_variant|MODIFIER|RBM23|ENSG00000100461|transcript|ENST00000554955|retained_intron||n.-4689G>A|||||4689|,T|upstream_gene_variant|MODIFIER|RBM23|ENSG00000100461|transcript|ENST00000556984|processed_transcript||n.-4650G>A|||||4650|,T|upstream_gene_variant|MODIFIER|RBM23|ENSG00000100461|transcript|ENST00000557549|protein_coding||c.-12406G>A|||||4650|WARNING_TRANSCRIPT_NO_STOP_CODON,T|upstream_gene_variant|MODIFIER|RBM23|ENSG00000100461|transcript|ENST00000555676|protein_coding||c.-12406G>A|||||4650|WARNING_TRANSCRIPT_NO_STOP_CODON,T|upstream_gene_variant|MODIFIER|RBM23|ENSG00000100461|transcript|ENST00000557571|protein_coding||c.-12406G>A|||||4650|WARNING_TRANSCRIPT_NO_STOP_CODON,T|upstream_gene_variant|MODIFIER|RBM23|ENSG00000100461|transcript|ENST00000556862|protein_coding||c.-12406G>A|||||4642|WARNING_TRANSCRIPT_INCOMPLETE,T|upstream_gene_variant|MODIFIER|RBM23|ENSG00000100461|transcript|ENST00000557464|protein_coding||c.-12406G>A|||||4650|WARNING_TRANSCRIPT_NO_STOP_CODON,T|upstream_gene_variant|MODIFIER|RBM23|ENSG00000100461|transcript|ENST00000554618|protein_coding||c.-12406G>A|||||4650|WARNING_TRANSCRIPT_INCOMPLETE,T|upstream_gene_variant|MODIFIER|RBM23|ENSG00000100461|transcript|ENST00000556365|processed_transcript||n.-4650G>A|||||4650|,T|upstream_gene_variant|MODIFIER|RBM23|ENSG00000100461|transcript|ENST00000553777|processed_transcript||n.-4712G>A|||||4712|,T|upstream_gene_variant|MODIFIER|PRMT5|ENSG00000100462|transcript|ENST00000557443|protein_coding||c.-1561G>A|||||1559|WARNING_TRANSCRIPT_NO_START_CODON,T|upstream_gene_variant|MODIFIER|PRMT5|ENSG00000100462|transcript|ENST00000476175|processed_transcript||n.-978G>A|||||978|,T|upstream_gene_variant|MODIFIER|PRMT5|ENSG00000100462|transcript|ENST00000454731|protein_coding||c.-638G>A|||||637|WARNING_TRANSCRIPT_NO_START_CODON,T|upstream_gene_variant|MODIFIER|PRMT5|ENSG00000100462|transcript|ENST00000557758|retained_intron||n.-889G>A|||||889|,T|downstream_gene_variant|MODIFIER|PRMT5-AS1|ENSG00000237054|transcript|ENST00000424245|antisense||n.*392C>T|||||392|,T|downstream_gene_variant|MODIFIER|PRMT5-AS1|ENSG00000237054|transcript|ENST00000609885|antisense||n.*548C>T|||||548|,T|downstream_gene_variant|MODIFIER|PRMT5-AS1|ENSG00000237054|transcript|ENST00000457443|antisense||n.*396C>T|||||396|,T|downstream_gene_variant|MODIFIER|PRMT5|ENSG00000100462|transcript|ENST00000553502|protein_coding||c.*363G>A|||||363|WARNING_TRANSCRIPT_NO_START_CODON,T|downstream_gene_variant|MODIFIER|PRMT5|ENSG00000100462|transcript|ENST00000555530|protein_coding||c.*518G>A|||||518|WARNING_TRANSCRIPT_NO_START_CODON,T|downstream_gene_variant|MODIFIER|PRMT5|ENSG00000100462|transcript|ENST00000556043|protein_coding||c.*700G>A|||||700|WARNING_TRANSCRIPT_NO_STOP_CODON,T|downstream_gene_variant|MODIFIER|PRMT5|ENSG00000100462|transcript|ENST00000553550|protein_coding||c.*839G>A|||||839|WARNING_TRANSCRIPT_NO_STOP_CODON,T|downstream_gene_variant|MODIFIER|PRMT5|ENSG00000100462|transcript|ENST00000554716|processed_transcript||n.*864G>A|||||864|,T|downstream_gene_variant|MODIFIER|PRMT5|ENSG00000100462|transcript|ENST00000553787|nonsense_mediated_decay||c.*3797G>A|||||1201|,T|downstream_gene_variant|MODIFIER|PRMT5|ENSG00000100462|transcript|ENST00000553641|processed_transcript||n.*2334G>A|||||2334|,T|downstream_gene_variant|MODIFIER|PRMT5|ENSG00000100462|transcript|ENST00000554867|protein_coding||c.*2436G>A|||||2436|WARNING_TRANSCRIPT_NO_STOP_CODON,T|downstream_gene_variant|MODIFIER|PRMT5|ENSG00000100462|transcript|ENST00000557415|nonsense_mediated_decay||c.*3797G>A|||||2439|,T|downstream_gene_variant|MODIFIER|PRMT5|ENSG00000100462|transcript|ENST00000556616|protein_coding||c.*2441G>A|||||2441|WARNING_TRANSCRIPT_INCOMPLETE,T|downstream_gene_variant|MODIFIER|PRMT5|ENSG00000100462|transcript|ENST00000554910|protein_coding||c.*2476G>A|||||2476|WARNING_TRANSCRIPT_INCOMPLETE,T|downstream_gene_variant|MODIFIER|PRMT5|ENSG00000100462|transcript|ENST00000553417|retained_intron||n.*2682G>A|||||2682|,T|downstream_gene_variant|MODIFIER|PRMT5|ENSG00000100462|transcript|ENST00000421938|protein_coding||c.*2938G>A|||||2938|WARNING_TRANSCRIPT_NO_STOP_CODON,T|downstream_gene_variant|MODIFIER|PRMT5|ENSG00000100462|transcript|ENST00000556032|retained_intron||n.*3727G>A|||||3727|,T|downstream_gene_variant|MODIFIER|PRMT5|ENSG00000100462|transcript|ENST00000557015|retained_intron||n.*4332G>A|||||4332|,T|downstream_gene_variant|MODIFIER|PRMT5|ENSG00000100462|transcript|ENST00000556426|processed_transcript||n.*4464G>A|||||4464|,T|intron_variant|MODIFIER|PRMT5|ENSG00000100462|transcript|ENST00000324366|protein_coding|12/16|c.1375+209G>A||||||,T|intron_variant|MODIFIER|PRMT5|ENSG00000100462|transcript|ENST00000397441|protein_coding|12/16|c.1324+209G>A||||||,T|intron_variant|MODIFIER|PRMT5|ENSG00000100462|transcript|ENST00000397440|protein_coding|8/12|c.862+209G>A||||||,T|intron_variant|MODIFIER|PRMT5|ENSG00000100462|transcript|ENST00000216350|protein_coding|11/15|c.1192+209G>A||||||,T|intron_variant|MODIFIER|PRMT5|ENSG00000100462|transcript|ENST00000555454|protein_coding|1/5|c.172+209G>A||||||WARNING_TRANSCRIPT_NO_START_CODON,T|intron_variant|MODIFIER|PRMT5|ENSG00000100462|transcript|ENST00000553915|nonsense_mediated_decay|11/15|c.*995+209G>A||||||,T|intron_variant|MODIFIER|PRMT5|ENSG00000100462|transcript|ENST00000538452|protein_coding|11/15|c.1057+209G>A||||||,T|intron_variant|MODIFIER|PRMT5|ENSG00000100462|transcript|ENST00000553897|protein_coding|11/15|c.1243+209G>A||||||,T|intron_variant|MODIFIER|PRMT5-AS1|ENSG00000237054|transcript|ENST00000599580|antisense|2/3|n.337-502C>T||||||,T|intron_variant|MODIFIER|PRMT5-AS1|ENSG00000237054|transcript|ENST00000595662|antisense|2/3|n.320-502C>T||||||,T|intron_variant|MODIFIER|PRMT5-AS1|ENSG00000237054|transcript|ENST00000587245|antisense|2/2|n.316+595C>T||||||,T|intron_variant|MODIFIER|PRMT5-AS1|ENSG00000237054|transcript|ENST00000590290|antisense|1/2|n.81-502C>T|||||| |
| RP11-298I3.1 | rs765316931 | 37 | 14 | 23397004 | 23397004 | + | SNP | C | C | T | SRR8586410 | Missense_Mutation | 0/1:139,210:349:99:6571,0,3454 | 350;ANN=T|upstream_gene_variant|MODIFIER|RP11-298I3.1|ENSG00000257285|transcript|ENST00000548322|antisense||n.-1850C>T|||||1850|,T|upstream_gene_variant|MODIFIER|PRMT5|ENSG00000100462|transcript|ENST00000476175|processed_transcript||n.-4974G>A|||||4974|,T|upstream_gene_variant|MODIFIER|PRMT5|ENSG00000100462|transcript|ENST00000555454|protein_coding||c.-3616G>A|||||3616|WARNING_TRANSCRIPT_NO_START_CODON,T|upstream_gene_variant|MODIFIER|PRMT5|ENSG00000100462|transcript|ENST00000454731|protein_coding||c.-4634G>A|||||4633|WARNING_TRANSCRIPT_NO_START_CODON,T|upstream_gene_variant|MODIFIER|PRMT5|ENSG00000100462|transcript|ENST00000557758|retained_intron||n.-4885G>A|||||4885|,T|upstream_gene_variant|MODIFIER|PRMT5|ENSG00000100462|transcript|ENST00000553502|protein_coding||c.-2749G>A|||||2749|WARNING_TRANSCRIPT_NO_START_CODON,T|upstream_gene_variant|MODIFIER|PRMT5|ENSG00000100462|transcript|ENST00000556043|protein_coding||c.-1630G>A|||||1339|WARNING_TRANSCRIPT_NO_STOP_CODON,T|upstream_gene_variant|MODIFIER|PRMT5|ENSG00000100462|transcript|ENST00000553417|retained_intron||n.-199G>A|||||199|,T|upstream_gene_variant|MODIFIER|RP11-298I3.1|ENSG00000257285|transcript|ENST00000548819|antisense||n.-1814C>T|||||1814|,T|downstream_gene_variant|MODIFIER|PRMT5-AS1|ENSG00000237054|transcript|ENST00000424245|antisense||n.*4388C>T|||||4388|,T|downstream_gene_variant|MODIFIER|PRMT5-AS1|ENSG00000237054|transcript|ENST00000609885|antisense||n.*4544C>T|||||4544|,T|downstream_gene_variant|MODIFIER|PRMT5-AS1|ENSG00000237054|transcript|ENST00000457443|antisense||n.*4392C>T|||||4392|,T|downstream_gene_variant|MODIFIER|PRMT5-AS1|ENSG00000237054|transcript|ENST00000599580|antisense||n.*965C>T|||||965|,T|downstream_gene_variant|MODIFIER|PRMT5-AS1|ENSG00000237054|transcript|ENST00000595662|antisense||n.*1176C>T|||||1176|,T|downstream_gene_variant|MODIFIER|PRMT5-AS1|ENSG00000237054|transcript|ENST00000587245|antisense||n.*970C>T|||||970|,T|downstream_gene_variant|MODIFIER|PRMT5-AS1|ENSG00000237054|transcript|ENST00000590290|antisense||n.*899C>T|||||899|,T|downstream_gene_variant|MODIFIER|PRMT5|ENSG00000100462|transcript|ENST00000557015|retained_intron||n.*336G>A|||||336|,T|downstream_gene_variant|MODIFIER|PRMT5|ENSG00000100462|transcript|ENST00000556426|processed_transcript||n.*468G>A|||||468|,T|intron_variant|MODIFIER|PRMT5|ENSG00000100462|transcript|ENST00000324366|protein_coding|3/16|c.316-135G>A||||||,T|intron_variant|MODIFIER|PRMT5|ENSG00000100462|transcript|ENST00000397441|protein_coding|3/16|c.265-135G>A||||||,T|intron_variant|MODIFIER|PRMT5|ENSG00000100462|transcript|ENST00000397440|protein_coding|3/12|c.264+331G>A||||||,T|intron_variant|MODIFIER|PRMT5|ENSG00000100462|transcript|ENST00000216350|protein_coding|2/15|c.179-181G>A||||||,T|intron_variant|MODIFIER|PRMT5|ENSG00000100462|transcript|ENST00000553915|nonsense_mediated_decay|2/15|c.146-135G>A||||||,T|intron_variant|MODIFIER|PRMT5|ENSG00000100462|transcript|ENST00000538452|protein_coding|2/15|c.-3-135G>A||||||,T|intron_variant|MODIFIER|PRMT5|ENSG00000100462|transcript|ENST00000553897|protein_coding|2/15|c.230-181G>A||||||,T|intron_variant|MODIFIER|PRMT5|ENSG00000100462|transcript|ENST00000555530|protein_coding|1/8|c.19-135G>A||||||WARNING_TRANSCRIPT_NO_START_CODON,T|intron_variant|MODIFIER|PRMT5|ENSG00000100462|transcript|ENST00000553550|protein_coding|3/4|c.315+331G>A||||||WARNING_TRANSCRIPT_NO_STOP_CODON,T|intron_variant|MODIFIER|PRMT5|ENSG00000100462|transcript|ENST00000554716|processed_transcript|3/4|n.352+331G>A||||||,T|intron_variant|MODIFIER|PRMT5|ENSG00000100462|transcript|ENST00000553787|nonsense_mediated_decay|1/5|c.111-181G>A||||||,T|intron_variant|MODIFIER|PRMT5|ENSG00000100462|transcript|ENST00000553641|processed_transcript|3/6|n.339-135G>A||||||,T|intron_variant|MODIFIER|PRMT5|ENSG00000100462|transcript|ENST00000554867|protein_coding|3/5|c.315+331G>A||||||WARNING_TRANSCRIPT_NO_STOP_CODON,T|intron_variant|MODIFIER|PRMT5|ENSG00000100462|transcript|ENST00000557415|nonsense_mediated_decay|2/5|c.197-135G>A||||||,T|intron_variant|MODIFIER|PRMT5|ENSG00000100462|transcript|ENST00000556616|protein_coding|2/5|c.202-135G>A||||||WARNING_TRANSCRIPT_INCOMPLETE,T|intron_variant|MODIFIER|PRMT5|ENSG00000100462|transcript|ENST00000554910|protein_coding|3/6|c.190-135G>A||||||WARNING_TRANSCRIPT_INCOMPLETE,T|intron_variant|MODIFIER|PRMT5|ENSG00000100462|transcript|ENST00000421938|protein_coding|3/4|c.346-135G>A||||||WARNING_TRANSCRIPT_NO_STOP_CODON,T|intron_variant|MODIFIER|PRMT5|ENSG00000100462|transcript|ENST00000556032|retained_intron|2/2|n.509-135G>A|||||| |
| PRMT5 | rs181613656 | 37 | 14 | 23400352 | 23400352 | + | SNP | G | G | A | SRR8586410 | Missense_Mutation | 0/1:180,45:225:99:1040,0,4911 | 225;ANN=A|upstream_gene_variant|MODIFIER|PRMT5|ENSG00000100462|transcript|ENST00000324366|protein_coding||c.-1782C>T|||||1558|,A|upstream_gene_variant|MODIFIER|PRMT5|ENSG00000100462|transcript|ENST00000397441|protein_coding||c.-1918C>T|||||1742|,A|upstream_gene_variant|MODIFIER|PRMT5|ENSG00000100462|transcript|ENST00000397440|protein_coding||c.-1918C>T|||||1787|,A|upstream_gene_variant|MODIFIER|PRMT5|ENSG00000100462|transcript|ENST00000216350|protein_coding||c.-1918C>T|||||1752|,A|upstream_gene_variant|MODIFIER|PRMT5|ENSG00000100462|transcript|ENST00000553915|nonsense_mediated_decay||c.-1918C>T|||||1778|,A|upstream_gene_variant|MODIFIER|PRMT5|ENSG00000100462|transcript|ENST00000538452|protein_coding||c.-3486C>T|||||1776|,A|upstream_gene_variant|MODIFIER|PRMT5|ENSG00000100462|transcript|ENST00000553897|protein_coding||c.-1782C>T|||||1735|,A|upstream_gene_variant|MODIFIER|PRMT5|ENSG00000100462|transcript|ENST00000555530|protein_coding||c.-3000C>T|||||3000|WARNING_TRANSCRIPT_NO_START_CODON,A|upstream_gene_variant|MODIFIER|PRMT5|ENSG00000100462|transcript|ENST00000556043|protein_coding||c.-4978C>T|||||4687|WARNING_TRANSCRIPT_NO_STOP_CODON,A|upstream_gene_variant|MODIFIER|PRMT5|ENSG00000100462|transcript|ENST00000553550|protein_coding||c.-1782C>T|||||1751|WARNING_TRANSCRIPT_NO_STOP_CODON,A|upstream_gene_variant|MODIFIER|PRMT5|ENSG00000100462|transcript|ENST00000554716|processed_transcript||n.-1778C>T|||||1778|,A|upstream_gene_variant|MODIFIER|PRMT5|ENSG00000100462|transcript|ENST00000553787|nonsense_mediated_decay||c.-1782C>T|||||1770|,A|upstream_gene_variant|MODIFIER|PRMT5|ENSG00000100462|transcript|ENST00000553641|processed_transcript||n.-1792C>T|||||1792|,A|upstream_gene_variant|MODIFIER|PRMT5|ENSG00000100462|transcript|ENST00000554867|protein_coding||c.-1782C>T|||||1767|WARNING_TRANSCRIPT_NO_STOP_CODON,A|upstream_gene_variant|MODIFIER|PRMT5|ENSG00000100462|transcript|ENST00000557415|nonsense_mediated_decay||c.-1782C>T|||||1751|,A|upstream_gene_variant|MODIFIER|PRMT5|ENSG00000100462|transcript|ENST00000556616|protein_coding||c.-1782C>T|||||1767|WARNING_TRANSCRIPT_INCOMPLETE,A|upstream_gene_variant|MODIFIER|PRMT5|ENSG00000100462|transcript|ENST00000554910|protein_coding||c.-2544C>T|||||2062|WARNING_TRANSCRIPT_INCOMPLETE,A|upstream_gene_variant|MODIFIER|PRMT5|ENSG00000100462|transcript|ENST00000553417|retained_intron||n.-3547C>T|||||3547|,A|upstream_gene_variant|MODIFIER|PRMT5|ENSG00000100462|transcript|ENST00000421938|protein_coding||c.-1782C>T|||||1775|WARNING_TRANSCRIPT_NO_STOP_CODON,A|upstream_gene_variant|MODIFIER|PRMT5|ENSG00000100462|transcript|ENST00000556032|retained_intron||n.-1792C>T|||||1792|,A|upstream_gene_variant|MODIFIER|PRMT5|ENSG00000100462|transcript|ENST00000557015|retained_intron||n.-1749C>T|||||1749|,A|upstream_gene_variant|MODIFIER|PRMT5|ENSG00000100462|transcript|ENST00000556426|processed_transcript||n.-1767C>T|||||1767|,A|downstream_gene_variant|MODIFIER|PRMT5-AS1|ENSG00000237054|transcript|ENST00000599580|antisense||n.*4313G>A|||||4313|,A|downstream_gene_variant|MODIFIER|PRMT5-AS1|ENSG00000237054|transcript|ENST00000595662|antisense||n.*4524G>A|||||4524|,A|downstream_gene_variant|MODIFIER|PRMT5-AS1|ENSG00000237054|transcript|ENST00000587245|antisense||n.*4318G>A|||||4318|,A|downstream_gene_variant|MODIFIER|PRMT5-AS1|ENSG00000237054|transcript|ENST00000590290|antisense||n.*4247G>A|||||4247|,A|intron_variant|MODIFIER|RP11-298I3.1|ENSG00000257285|transcript|ENST00000548322|antisense|1/2|n.108+1391G>A||||||,A|intron_variant|MODIFIER|RP11-298I3.1|ENSG00000257285|transcript|ENST00000548819|antisense|1/2|n.144+1391G>A|||||| |
| WDR59 | rs764190356 | 37 | 16 | 74903925 | 74903925 | + | SNP | A | A | T | SRR8586410 | Nonsense_Mutation | 0/1:18,9:27:99:366,0,726 | 27;ANN=T|downstream_gene_variant|MODIFIER|WDR59|ENSG00000103091|transcript|ENST00000262144|protein_coding||c.*4182T>A|||||3543|,T|downstream_gene_variant|MODIFIER|WDR59|ENSG00000103091|transcript|ENST00000569183|retained_intron||n.*4052T>A|||||4052|,T|downstream_gene_variant|MODIFIER|WDR59|ENSG00000103091|transcript|ENST00000569968|retained_intron||n.*4368T>A|||||4368|,T|downstream_gene_variant|MODIFIER|WDR59|ENSG00000103091|transcript|ENST00000563797|protein_coding||c.*4399T>A|||||4399|WARNING_TRANSCRIPT_INCOMPLETE,T|intergenic_region|MODIFIER|RP11-787D11.1-WDR59|ENSG00000261458-ENSG00000103091|intergenic_region|ENSG00000261458-ENSG00000103091|||n.74903925A>T|||||| |
| GRAPL | rs373466781 | 37 | 17 | 19062028 | 19062028 | + | SNP | C | C | T | SRR8586410 | Nonstop_Mutation | 0/1:30,34:64:99:1008,0,736 | 64;ANN=T|3_prime_UTR_variant|MODIFIER|GRAPL|ENSG00000189152|transcript|ENST00000344415|protein_coding|4/4|c.*339C>T|||||339|WARNING_REF_DOES_NOT_MATCH_GENOME,T|3_prime_UTR_variant|MODIFIER|GRAPL|ENSG00000189152|transcript|ENST00000574324|protein_coding|3/3|c.*339C>T|||||339|WARNING_TRANSCRIPT_NO_START_CODON&WARNING_REF_DOES_NOT_MATCH_GENOME,T|3_prime_UTR_variant|MODIFIER|GRAPL|ENSG00000189152|transcript|ENST00000583540|nonsense_mediated_decay|3/3|c.*452C>T|||||14600|WARNING_TRANSCRIPT_NO_START_CODON&WARNING_REF_DOES_NOT_MATCH_GENOME,T|upstream_gene_variant|MODIFIER|RP11-160E2.11|ENSG00000262292|transcript|ENST00000572818|lincRNA||n.-2074C>T|||||2074|,T|intron_variant|MODIFIER|AC007952.6|ENSG00000197665|transcript|ENST00000399083|protein_coding|1/1|c.67+64G>A||||||,T|intron_variant|MODIFIER|AC007952.6|ENSG00000197665|transcript|ENST00000399087|protein_coding|1/2|c.67+64G>A||||||,T|intron_variant|MODIFIER|AC007952.6|ENSG00000197665|transcript|ENST00000436381|protein_coding|1/2|c.115+64G>A|||||| |
| GRAPL | rs201695897 | 37 | 17 | 19062050 | 19062050 | + | SNP | T | T | C | SRR8586410 | Nonstop_Mutation | 0/1:32,35:67:99:847,0,897 | 67;ANN=C|3_prime_UTR_variant|MODIFIER|GRAPL|ENSG00000189152|transcript|ENST00000344415|protein_coding|4/4|c.*361T>C|||||361|WARNING_REF_DOES_NOT_MATCH_GENOME,C|3_prime_UTR_variant|MODIFIER|GRAPL|ENSG00000189152|transcript|ENST00000574324|protein_coding|3/3|c.*361T>C|||||361|WARNING_TRANSCRIPT_NO_START_CODON&WARNING_REF_DOES_NOT_MATCH_GENOME,C|3_prime_UTR_variant|MODIFIER|GRAPL|ENSG00000189152|transcript|ENST00000583540|nonsense_mediated_decay|3/3|c.*474T>C|||||14622|WARNING_TRANSCRIPT_NO_START_CODON&WARNING_REF_DOES_NOT_MATCH_GENOME,C|upstream_gene_variant|MODIFIER|RP11-160E2.11|ENSG00000262292|transcript|ENST00000572818|lincRNA||n.-2052T>C|||||2052|,C|intron_variant|MODIFIER|AC007952.6|ENSG00000197665|transcript|ENST00000399083|protein_coding|1/1|c.67+42A>G||||||,C|intron_variant|MODIFIER|AC007952.6|ENSG00000197665|transcript|ENST00000399087|protein_coding|1/2|c.67+42A>G||||||,C|intron_variant|MODIFIER|AC007952.6|ENSG00000197665|transcript|ENST00000436381|protein_coding|1/2|c.115+42A>G|||||| |
| FSTL3 | rs2057713 | 37 | 19 | 682234 | 682234 | + | SNP | G | G | A | SRR8586410 | Nonstop_Mutation | 0/1:32,166:198:30:4553,0,30 | 199;ANN=A|3_prime_UTR_variant|MODIFIER|FSTL3|ENSG00000070404|transcript|ENST00000166139|protein_coding|5/5|c.*526G>A|||||526|,A|downstream_gene_variant|MODIFIER|PRSS57|ENSG00000185198|transcript|ENST00000329267|protein_coding||c.*3482C>T|||||3287|,A|downstream_gene_variant|MODIFIER|FSTL3|ENSG00000070404|transcript|ENST00000606071|processed_transcript||n.*1901G>A|||||1901|,A|downstream_gene_variant|MODIFIER|FSTL3|ENSG00000070404|transcript|ENST00000591552|protein_coding||c.*424G>A|||||180|WARNING_TRANSCRIPT_NO_START_CODON,A|downstream_gene_variant|MODIFIER|FSTL3|ENSG00000070404|transcript|ENST00000588773|nonsense_mediated_decay||c.*892G>A|||||440|WARNING_TRANSCRIPT_NO_START_CODON,A|downstream_gene_variant|MODIFIER|FSTL3|ENSG00000070404|transcript|ENST00000589185|retained_intron||n.*478G>A|||||478|,A|downstream_gene_variant|MODIFIER|FSTL3|ENSG00000070404|transcript|ENST00000592058|processed_transcript||n.*291G>A|||||291|,A|downstream_gene_variant|MODIFIER|FSTL3|ENSG00000070404|transcript|ENST00000592947|processed_transcript||n.*29G>A|||||29|,A|downstream_gene_variant|MODIFIER|FSTL3|ENSG00000070404|transcript|ENST00000591573|retained_intron||n.*463G>A|||||463|,A|downstream_gene_variant|MODIFIER|FSTL3|ENSG00000070404|transcript|ENST00000605925|processed_transcript||n.*425G>A|||||425| |
| PLIN4 | rs4807595 | 37 | 19 | 4513131 | 4513131 | + | SNP | C | C | T | SRR8586410 | Missense_Mutation | 0/1:38,107:145:99:3908,0,1801 | 149;ANN=T|missense_variant|MODERATE|PLIN4|ENSG00000167676|transcript|ENST00000301286|protein_coding|3/6|c.799G>A|p.Val267Met|799/6341|799/4074|267/1357|| |
| PLIN4 | rs4807596 | 37 | 19 | 4513132 | 4513132 | + | SNP | A | A | G | SRR8586410 | Nonsense_Mutation | 0/1:38,108:146:99:3908,0,1801 | 150;ANN=G|synonymous_variant|LOW|PLIN4|ENSG00000167676|transcript|ENST00000301286|protein_coding|3/6|c.798T>C|p.Asn266Asn|798/6341|798/4074|266/1357|| |
| RFX1 | rs759743296 | 37 | 19 | 14093077 | 14093077 | + | SNP | G | G | A | SRR8586410 | Nonsense_Mutation | 0/1:167,13:180:73:73,0,9366 | 180;ANN=A|downstream_gene_variant|MODIFIER|RFX1|ENSG00000132005|transcript|ENST00000589937|retained_intron||n.*763C>T|||||763|,A|downstream_gene_variant|MODIFIER|RFX1|ENSG00000132005|transcript|ENST00000589760|retained_intron||n.*804C>T|||||804|,A|downstream_gene_variant|MODIFIER|RFX1|ENSG00000132005|transcript|ENST00000588885|protein_coding||c.*1048C>T|||||1048|WARNING_TRANSCRIPT_NO_STOP_CODON,A|intron_variant|MODIFIER|RFX1|ENSG00000132005|transcript|ENST00000254325|protein_coding|4/20|c.514-37C>T||||||,A|intron_variant|MODIFIER|RFX1|ENSG00000132005|transcript|ENST00000589239|retained_intron|4/9|n.786-37C>T|||||| |
| ARHGAP33 | rs199987073 | 37 | 19 | 36270392 | 36270392 | + | SNP | C | C | A | SRR8586410 | Missense_Mutation | 0/1:39,9:48:99:180,0,1250 | 48;ANN=A|upstream_gene_variant|MODIFIER|ARHGAP33|ENSG00000004777|transcript|ENST00000601474|retained_intron||n.-1680C>A|||||1680|,A|upstream_gene_variant|MODIFIER|ARHGAP33|ENSG00000004777|transcript|ENST00000588248|protein_coding||c.-2884C>A|||||2883|WARNING_TRANSCRIPT_NO_START_CODON,A|upstream_gene_variant|MODIFIER|ARHGAP33|ENSG00000004777|transcript|ENST00000591438|processed_transcript||n.-2983C>A|||||2983|,A|upstream_gene_variant|MODIFIER|ARHGAP33|ENSG00000004777|transcript|ENST00000586918|retained_intron||n.-3135C>A|||||3135|,A|upstream_gene_variant|MODIFIER|ARHGAP33|ENSG00000004777|transcript|ENST00000587447|protein_coding||c.-3152C>A|||||3151|WARNING_TRANSCRIPT_NO_START_CODON,A|downstream_gene_variant|MODIFIER|ARHGAP33|ENSG00000004777|transcript|ENST00000589133|processed_transcript||n.*941C>A|||||941|,A|intron_variant|MODIFIER|ARHGAP33|ENSG00000004777|transcript|ENST00000007510|protein_coding|6/20|c.501+366C>A||||||,A|intron_variant|MODIFIER|ARHGAP33|ENSG00000004777|transcript|ENST00000378944|protein_coding|5/20|c.93+366C>A||||||,A|intron_variant|MODIFIER|ARHGAP33|ENSG00000004777|transcript|ENST00000590893|nonsense_mediated_decay|6/6|c.*111+366C>A||||||,A|intron_variant|MODIFIER|ARHGAP33|ENSG00000004777|transcript|ENST00000314737|protein_coding|6/20|c.501+366C>A||||||,A|intron_variant|MODIFIER|ARHGAP33|ENSG00000004777|transcript|ENST00000221905|processed_transcript|5/5|n.601+320C>A|||||| |
| ALK-AC016907.3 | rs13389106 | 37 | 2 | 30257884 | 30257884 | + | SNP | T | T | G | SRR8586410 | Missense_Mutation | 0/1:71,21:92:99:688,0,4389 | 92;ANN=G|intergenic_region|MODIFIER|ALK-AC016907.3|ENSG00000171094-ENSG00000233862|intergenic_region|ENSG00000171094-ENSG00000233862|||n.30257884T>G|||||| |
| ALK-AC016907.3 | rs13386353 | 37 | 2 | 30257890 | 30257890 | + | SNP | A | A | G | SRR8586410 | Missense_Mutation | 0/1:73,24:97:99:709,0,4470 | 97;ANN=G|intergenic_region|MODIFIER|ALK-AC016907.3|ENSG00000171094-ENSG00000233862|intergenic_region|ENSG00000171094-ENSG00000233862|||n.30257890A>G|||||| |
| PDE11A | rs3866706 | 37 | 2 | 178655278 | 178655278 | + | SNP | A | A | G | SRR8586410 | Missense_Mutation | 0/1:308,162:470:99:3712,0,7992 | 470;ANN=G|intron_variant|MODIFIER|PDE11A|ENSG00000128655|transcript|ENST00000286063|protein_coding|9/19|c.1738-21177T>C||||||,G|intron_variant|MODIFIER|PDE11A|ENSG00000128655|transcript|ENST00000358450|protein_coding|10/20|c.988-21177T>C||||||,G|intron_variant|MODIFIER|PDE11A|ENSG00000128655|transcript|ENST00000409504|protein_coding|8/19|c.664-21177T>C||||||,G|intron_variant|MODIFIER|PDE11A|ENSG00000128655|transcript|ENST00000389683|protein_coding|6/16|c.406-21177T>C||||||,G|intron_variant|MODIFIER|PDE11A|ENSG00000128655|transcript|ENST00000449286|protein_coding|8/18|c.664-21177T>C||||||,G|intron_variant|MODIFIER|PDE11A|ENSG00000128655|transcript|ENST00000433879|protein_coding|6/13|c.559-21177T>C||||||WARNING_TRANSCRIPT_NO_START_CODON,G|intron_variant|MODIFIER|PDE11A|ENSG00000128655|transcript|ENST00000497003|processed_transcript|8/14|n.780-21177T>C|||||| |
| PDE11A | rs770610620 | 37 | 2 | 178658393 | 178658393 | + | SNP | T | T | C | SRR8586410 | Nonsense_Mutation | 0/1:441,54:495:99:154,0,12975 | 495;ANN=C|downstream_gene_variant|MODIFIER|PDE11A|ENSG00000128655|transcript|ENST00000492761|processed_transcript||n.*3406A>G|||||3406|,C|intron_variant|MODIFIER|PDE11A|ENSG00000128655|transcript|ENST00000286063|protein_coding|9/19|c.1737+23163A>G||||||,C|intron_variant|MODIFIER|PDE11A|ENSG00000128655|transcript|ENST00000358450|protein_coding|10/20|c.987+23163A>G||||||,C|intron_variant|MODIFIER|PDE11A|ENSG00000128655|transcript|ENST00000409504|protein_coding|8/19|c.663+23163A>G||||||,C|intron_variant|MODIFIER|PDE11A|ENSG00000128655|transcript|ENST00000389683|protein_coding|6/16|c.405+23163A>G||||||,C|intron_variant|MODIFIER|PDE11A|ENSG00000128655|transcript|ENST00000449286|protein_coding|8/18|c.663+23163A>G||||||,C|intron_variant|MODIFIER|PDE11A|ENSG00000128655|transcript|ENST00000433879|protein_coding|6/13|c.558+24192A>G||||||WARNING_TRANSCRIPT_NO_START_CODON,C|intron_variant|MODIFIER|PDE11A|ENSG00000128655|transcript|ENST00000497003|processed_transcript|8/14|n.779+23163A>G|||||| |
| PDE11A | rs146313925 | 37 | 2 | 178662395 | 178662395 | + | SNP | T | T | C | SRR8586410 | Missense_Mutation | 0/1:230,63:293:99:1016,0,6108 | 293;ANN=C|intron_variant|MODIFIER|PDE11A|ENSG00000128655|transcript|ENST00000286063|protein_coding|9/19|c.1737+19161A>G||||||,C|intron_variant|MODIFIER|PDE11A|ENSG00000128655|transcript|ENST00000358450|protein_coding|10/20|c.987+19161A>G||||||,C|intron_variant|MODIFIER|PDE11A|ENSG00000128655|transcript|ENST00000409504|protein_coding|8/19|c.663+19161A>G||||||,C|intron_variant|MODIFIER|PDE11A|ENSG00000128655|transcript|ENST00000389683|protein_coding|6/16|c.405+19161A>G||||||,C|intron_variant|MODIFIER|PDE11A|ENSG00000128655|transcript|ENST00000449286|protein_coding|8/18|c.663+19161A>G||||||,C|intron_variant|MODIFIER|PDE11A|ENSG00000128655|transcript|ENST00000433879|protein_coding|6/13|c.558+20190A>G||||||WARNING_TRANSCRIPT_NO_START_CODON,C|intron_variant|MODIFIER|PDE11A|ENSG00000128655|transcript|ENST00000497003|processed_transcript|8/14|n.779+19161A>G||||||,C|intron_variant|MODIFIER|PDE11A|ENSG00000128655|transcript|ENST00000492761|processed_transcript|6/6|n.594-448A>G|||||| |
| PDE11A | rs12998857 | 37 | 2 | 178668882 | 178668882 | + | SNP | A | A | C | SRR8586410 | Missense_Mutation | 0/1:222,150:372:99:2929,0,6160 | 385;ANN=C|intron_variant|MODIFIER|PDE11A|ENSG00000128655|transcript|ENST00000286063|protein_coding|9/19|c.1737+12674T>G||||||,C|intron_variant|MODIFIER|PDE11A|ENSG00000128655|transcript|ENST00000358450|protein_coding|10/20|c.987+12674T>G||||||,C|intron_variant|MODIFIER|PDE11A|ENSG00000128655|transcript|ENST00000409504|protein_coding|8/19|c.663+12674T>G||||||,C|intron_variant|MODIFIER|PDE11A|ENSG00000128655|transcript|ENST00000389683|protein_coding|6/16|c.405+12674T>G||||||,C|intron_variant|MODIFIER|PDE11A|ENSG00000128655|transcript|ENST00000449286|protein_coding|8/18|c.663+12674T>G||||||,C|intron_variant|MODIFIER|PDE11A|ENSG00000128655|transcript|ENST00000433879|protein_coding|6/13|c.558+13703T>G||||||WARNING_TRANSCRIPT_NO_START_CODON,C|intron_variant|MODIFIER|PDE11A|ENSG00000128655|transcript|ENST00000497003|processed_transcript|8/14|n.779+12674T>G||||||,C|intron_variant|MODIFIER|PDE11A|ENSG00000128655|transcript|ENST00000492761|processed_transcript|6/6|n.594-6935T>G|||||| |
| PDE11A | rs776747375 | 37 | 2 | 178782599 | 178782599 | + | SNP | G | G | T | SRR8586410 | Missense_Mutation | 0/1:219,142:361:99:3167,0,7523 | 407;ANN=T|intron_variant|MODIFIER|PDE11A|ENSG00000128655|transcript|ENST00000286063|protein_coding|2/19|c.1072-12685C>A||||||,T|intron_variant|MODIFIER|PDE11A|ENSG00000128655|transcript|ENST00000358450|protein_coding|3/20|c.322-12685C>A||||||,T|intron_variant|MODIFIER|PDE11A|ENSG00000128655|transcript|ENST00000409504|protein_coding|1/19|c.-4+4836C>A||||||,T|intron_variant|MODIFIER|PDE11A|ENSG00000128655|transcript|ENST00000449286|protein_coding|1/18|c.-4+4836C>A||||||,T|intron_variant|MODIFIER|PDE11A|ENSG00000128655|transcript|ENST00000497003|processed_transcript|1/14|n.113+9025C>A||||||,T|intron_variant|MODIFIER|PDE11A|ENSG00000128655|transcript|ENST00000427127|nonsense_mediated_decay|2/4|c.-4+3859C>A|||||| |
| TBC1D5 | rs371256943 | 37 | 3 | 17860355 | 17860355 | + | SNP | C | C | T | SRR8586410 | Missense_Mutation | 0/1:5,18:23:99:586,0,112 | 23;ANN=T|intron_variant|MODIFIER|TBC1D5|ENSG00000131374|transcript|ENST00000414318|processed_transcript|1/8|n.212-510729G>A|||||| |
| AC124944.5 | rs369497400 | 37 | 3 | 195568833 | 195568833 | + | INS | G | G | GC | SRR8586410 | Frame_Shift_Ins | 0/1:130,250:380:99:7075,0,3519 | 381;ANN=GC|upstream_gene_variant|MODIFIER|AC124944.5|ENSG00000223783|transcript|ENST00000413586|lincRNA||n.-4918_-4917insG|||||4918|,GC|upstream_gene_variant|MODIFIER|AC124944.4|ENSG00000235836|transcript|ENST00000454055|processed_pseudogene||n.-2024_-2023insC|||||2023|,GC|intron_variant|MODIFIER|AC124944.5|ENSG00000223783|transcript|ENST00000444346|lincRNA|1/1|n.97-5348_97-5347insG||||||,GC|intron_variant|MODIFIER|AC124944.5|ENSG00000223783|transcript|ENST00000429834|lincRNA|1/1|n.133-5348_133-5347insG|||||| |
| ANKH | rs562391245 | 37 | 5 | 14797376 | 14797376 | + | SNP | G | G | A | SRR8586410 | Missense_Mutation | 0/1:17,16:33:99:621,0,1525 | 33;ANN=A|intron_variant|MODIFIER|ANKH|ENSG00000154122|transcript|ENST00000284268|protein_coding|1/11|c.97-28076C>T||||||,A|intron_variant|MODIFIER|ANKH|ENSG00000154122|transcript|ENST00000513115|retained_intron|1/1|n.122-28076C>T||||||,A|non_coding_transcript_exon_variant|MODIFIER|RBBP4P1|ENSG00000249485|transcript|ENST00000502424|processed_pseudogene|1/1|n.1134C>T||||||WARNING_REF_DOES_NOT_MATCH_GENOME |
| DCTN4 | rs13164723 | 37 | 5 | 150094860 | 150094860 | + | SNP | G | A | A | SRR8586410 | Nonsense_Mutation | 1/1:3,55:58:99:1909,103,0 | 58;ANN=A|downstream_gene_variant|MODIFIER|DCTN4|ENSG00000132912|transcript|ENST00000518909|retained_intron||n.*2978C>T|||||2978|,A|intron_variant|MODIFIER|DCTN4|ENSG00000132912|transcript|ENST00000446090|protein_coding|13/13|c.1190+267C>T||||||,A|intron_variant|MODIFIER|DCTN4|ENSG00000132912|transcript|ENST00000447998|protein_coding|12/12|c.1169+267C>T||||||,A|intron_variant|MODIFIER|DCTN4|ENSG00000132912|transcript|ENST00000424236|protein_coding|12/12|c.998+267C>T|||||| |
| RBM24 | rs373325663 | 37 | 6 | 17282608 | 17282608 | + | DEL | GCCC | GCCC | G | SRR8586410 | In_Frame_Del | 0/1:4,26:30:7:944,0,7 | 34;ANN=G|conservative_inframe_deletion|MODERATE|RBM24|ENSG00000112183|transcript|ENST00000503965|protein_coding|1/4|c.40_42delCCC|p.Gly14del|42/622|40/603|14/200||WARNING_TRANSCRIPT_NO_START_CODON,G|upstream_gene_variant|MODIFIER|RBM24|ENSG00000112183|transcript|ENST00000318204|protein_coding||c.-394_-392delCCC|||||323|,G|upstream_gene_variant|MODIFIER|RBM24|ENSG00000112183|transcript|ENST00000504055|processed_transcript||n.-666_-664delCCC|||||666|,G|intron_variant|MODIFIER|RBM24|ENSG00000112183|transcript|ENST00000379052|protein_coding|1/3|c.169-427_169-425delCCC||||||,G|intron_variant|MODIFIER|RBM24|ENSG00000112183|transcript|ENST00000509686|protein_coding|1/4|c.46-427_46-425delCCC||||||WARNING_TRANSCRIPT_NO_START_CODON,G|intron_variant|MODIFIER|RBM24|ENSG00000112183|transcript|ENST00000425446|protein_coding|1/3|c.-7+159_-7+161delCCC|||||| |
| INMT-FAM188B | rs571614381 | 37 | 7 | 30923681 | 30923681 | + | INS | A | A | AGGGAG | SRR8586410 | In_Frame_Ins | 0/1:73,44:117:99:1663,0,3633 | 124;ANN=AGGGAG|intron_variant|MODIFIER|INMT-FAM188B|ENSG00000254959|transcript|ENST00000458257|nonsense_mediated_decay|19/19|c.*2312+1073_*2312+1074insGGGAG||||||,AGGGAG|intron_variant|MODIFIER|FAM188B|ENSG00000106125|transcript|ENST00000265299|protein_coding|17/17|c.2225+1073_2225+1074insGGGAG||||||,AGGGAG|intron_variant|MODIFIER|AQP1|ENSG00000240583|transcript|ENST00000434909|protein_coding|6/9|c.263+1073_263+1074insGGGAG||||||,AGGGAG|intron_variant|MODIFIER|AQP1|ENSG00000250424|transcript|ENST00000509504|protein_coding|6/9|c.614+1073_614+1074insGGGAG||||||WARNING_TRANSCRIPT_NO_START_CODON,AGGGAG|intron_variant|MODIFIER|FAM188B|ENSG00000106125|transcript|ENST00000409881|retained_intron|5/5|n.2058+1073_2058+1074insGGGAG|||||| |
| AC004980.1 | rs560593469 | 37 | 7 | 76327668 | 76327668 | + | DEL | GCCC | G | GCC | SRR8586410 | Missense_Mutation | 1/2:6,48,35:89:99:2790,859,1212,1692,0,1562 | 137;ANN="G|upstream_gene_variant|MODIFIER|AC004980.1|ENSG00000221249|transcript|ENST00000408322|miRNA||n.-700_-697delGGGCinsC"|||||697|,GCC"|upstream_gene_variant|MODIFIER|AC004980.1|ENSG00000221249|transcript|ENST00000408322|miRNA||n.-700G>"|||||700|,"G|intron_variant|MODIFIER|UPK3B|ENSG00000243566|transcript|ENST00000419923|protein_coding|5/5|c.*220+51547_*220+51550delGCCCins"G||||||,GCC"|intron_variant|MODIFIER|UPK3B|ENSG00000243566|transcript|ENST00000419923|protein_coding|5/5|c.*220+51550C>"||||||,"G|intron_variant|MODIFIER|UPK3B|ENSG00000243566|transcript|ENST00000443097|protein_coding|7/7|c.*267+51547_*267+51550delGCCCins"G||||||,GCC"|intron_variant|MODIFIER|UPK3B|ENSG00000243566|transcript|ENST00000443097|protein_coding|7/7|c.*267+51550C>"|||||| |
| EXOC4 | rs12673323 | 37 | 7 | 133034703 | 133034703 | + | SNP | T | T | G | SRR8586410 | Missense_Mutation | 0/1:14,74:88:99:1859,0,231 | 96;ANN=G|intron_variant|MODIFIER|EXOC4|ENSG00000131558|transcript|ENST00000253861|protein_coding|5/17|c.764-6381T>G||||||,G|intron_variant|MODIFIER|EXOC4|ENSG00000131558|transcript|ENST00000393161|protein_coding|5/9|c.764-6381T>G||||||,G|intron_variant|MODIFIER|EXOC4|ENSG00000131558|transcript|ENST00000486013|retained_intron|5/9|n.793-6381T>G||||||,G|intron_variant|MODIFIER|EXOC4|ENSG00000131558|transcript|ENST00000462055|retained_intron|5/8|n.771-6381T>G||||||,G|intron_variant|MODIFIER|EXOC4|ENSG00000131558|transcript|ENST00000539845|protein_coding|5/17|c.461-6381T>G|||||| |
| EXOC4 | rs12674366 | 37 | 7 | 133035836 | 133035836 | + | SNP | T | T | C | SRR8586410 | Missense_Mutation | 0/1:11,105:116:7:3087,0,7 | 118;ANN=C|intron_variant|MODIFIER|EXOC4|ENSG00000131558|transcript|ENST00000253861|protein_coding|5/17|c.764-5248T>C||||||,C|intron_variant|MODIFIER|EXOC4|ENSG00000131558|transcript|ENST00000393161|protein_coding|5/9|c.764-5248T>C||||||,C|intron_variant|MODIFIER|EXOC4|ENSG00000131558|transcript|ENST00000486013|retained_intron|5/9|n.793-5248T>C||||||,C|intron_variant|MODIFIER|EXOC4|ENSG00000131558|transcript|ENST00000462055|retained_intron|5/8|n.771-5248T>C||||||,C|intron_variant|MODIFIER|EXOC4|ENSG00000131558|transcript|ENST00000539845|protein_coding|5/17|c.461-5248T>C|||||| |
| RP11-632K21.6 | rs17173702 | 37 | 7 | 150371516 | 150371516 | + | SNP | G | G | A | SRR8586410 | Nonsense_Mutation | 0/1:66,58:124:99:2157,0,3824 | 124;ANN=A|downstream_gene_variant|MODIFIER|RP11-632K21.6|ENSG00000270990|transcript|ENST00000605154|processed_pseudogene||n.*2405C>T|||||2405|,A|intergenic_region|MODIFIER|GIMAP6-RP11-632K21.6|ENSG00000133561-ENSG00000270990|intergenic_region|ENSG00000133561-ENSG00000270990|||n.150371516G>A|||||| |
| RP11-632K21.6 | rs17173703 | 37 | 7 | 150371531 | 150371531 | + | SNP | C | C | T | SRR8586410 | Nonsense_Mutation | 0/1:68,59:127:99:2220,0,3843 | 127;ANN=T|downstream_gene_variant|MODIFIER|RP11-632K21.6|ENSG00000270990|transcript|ENST00000605154|processed_pseudogene||n.*2390G>A|||||2390|,T|intergenic_region|MODIFIER|GIMAP6-RP11-632K21.6|ENSG00000133561-ENSG00000270990|intergenic_region|ENSG00000133561-ENSG00000270990|||n.150371531C>T|||||| |
| MYOM2 | rs3779840 | 37 | 8 | 2100690 | 2100690 | + | SNP | G | G | A | SRR8586410 | Missense_Mutation | 0/1:16,42:58:99:1289,0,325 | 59;ANN=A|intron_variant|MODIFIER|MYOM2|ENSG00000036448|transcript|ENST00000520779|processed_transcript|4/4|n.154+9310G>A|||||| |
| MYOM2 | rs3837173 | 37 | 8 | 2100702 | 2100702 | + | INS | A | A | AC | SRR8586410 | Frame_Shift_Ins | 0/1:21,37:58:99:1064,0,537 | 60;ANN=AC|intron_variant|MODIFIER|MYOM2|ENSG00000036448|transcript|ENST00000520779|processed_transcript|4/4|n.154+9322dupC|||||| |
| MYOM2 | rs147135468 | 37 | 8 | 2100716 | 2100716 | + | SNP | G | G | C | SRR8586410 | Missense_Mutation | 0/1:38,28:66:99:650,0,1056 | 67;ANN=C|intron_variant|MODIFIER|MYOM2|ENSG00000036448|transcript|ENST00000520779|processed_transcript|4/4|n.154+9336G>C|||||| |
| MYOM2 | rs968381 | 37 | 8 | 2101041 | 2101041 | + | SNP | G | G | A | SRR8586410 | Missense_Mutation | 0/1:159,44:203:99:969,0,4234 | 231;ANN=A|intron_variant|MODIFIER|MYOM2|ENSG00000036448|transcript|ENST00000520779|processed_transcript|4/4|n.154+9661G>A|||||| |
| TRPM3 | rs113775121 | 37 | 9 | 73455323 | 73455323 | + | SNP | T | T | C | SRR8586410 | Nonsense_Mutation | 0/1:7,11:18:99:419,0,473 | 18;ANN=C|downstream_gene_variant|MODIFIER|TRPM3|ENSG00000083067|transcript|ENST00000377097|protein_coding||c.*2566A>G|||||2512|,C|intron_variant|MODIFIER|TRPM3|ENSG00000083067|transcript|ENST00000423814|protein_coding|5/25|c.807+2596A>G||||||,C|intron_variant|MODIFIER|TRPM3|ENSG00000083067|transcript|ENST00000377110|protein_coding|5/24|c.801+2596A>G||||||,C|intron_variant|MODIFIER|TRPM3|ENSG00000083067|transcript|ENST00000377111|protein_coding|5/25|c.801+2596A>G||||||,C|intron_variant|MODIFIER|TRPM3|ENSG00000083067|transcript|ENST00000360823|protein_coding|5/24|c.342+2596A>G||||||,C|intron_variant|MODIFIER|TRPM3|ENSG00000083067|transcript|ENST00000377105|protein_coding|5/25|c.342+2596A>G||||||,C|intron_variant|MODIFIER|TRPM3|ENSG00000083067|transcript|ENST00000377106|protein_coding|5/25|c.342+2596A>G||||||,C|intron_variant|MODIFIER|TRPM3|ENSG00000083067|transcript|ENST00000357533|protein_coding|5/24|c.807+2596A>G||||||,C|intron_variant|MODIFIER|TRPM3|ENSG00000083067|transcript|ENST00000358082|protein_coding|3/22|c.342+2596A>G||||||,C|intron_variant|MODIFIER|TRPM3|ENSG00000083067|transcript|ENST00000396280|protein_coding|3/22|c.342+2596A>G||||||,C|intron_variant|MODIFIER|TRPM3|ENSG00000083067|transcript|ENST00000396285|protein_coding|3/22|c.342+2596A>G||||||,C|intron_variant|MODIFIER|TRPM3|ENSG00000083067|transcript|ENST00000396292|protein_coding|3/23|c.342+2596A>G||||||,C|intron_variant|MODIFIER|TRPM3|ENSG00000083067|transcript|ENST00000408909|protein_coding|3/23|c.342+2596A>G||||||,C|intron_variant|MODIFIER|TRPM3|ENSG00000083067|transcript|ENST00000377101|protein_coding|5/7|c.342+2596A>G||||||,C|intron_variant|MODIFIER|TRPM3|ENSG00000083067|transcript|ENST00000396283|protein_coding|5/8|c.342+2596A>G||||||,C|intron_variant|MODIFIER|TRPM3|ENSG00000083067|transcript|ENST00000361823|protein_coding|5/6|c.342+2596A>G||||||,C|intron_variant|MODIFIER|TRPM3|ENSG00000083067|transcript|ENST00000354500|retained_intron|5/5|n.876+2596A>G|||||| |
| RP5-857K21.4 | rs7518996 | 37 | 1 | 631862 | 631862 | + | SNP | G | G | A | SRR8586411 | Missense_Mutation | 0/1:50,338:388:83:8625,0,83 | 398;ANN=A|intron_variant|MODIFIER|RP5-857K21.4|ENSG00000230021|transcript|ENST00000440200|lincRNA|1/2|n.169+23550C>T|||||| |
| ALPL | rs781400739 | 37 | 1 | 21890706 | 21890706 | + | SNP | A | A | C | SRR8586411 | Nonsense_Mutation | 0/1:13,84:97:99:3356,0,10317 | 97;ANN=C|synonymous_variant|LOW|ALPL|ENSG00000162551|transcript|ENST00000374832|protein_coding|6/12|c.645A>C|p.Ile215Ile|899/2193|645/1575|215/524||WARNING_REF_DOES_NOT_MATCH_GENOME,C|synonymous_variant|LOW|ALPL|ENSG00000162551|transcript|ENST00000539907|protein_coding|4/10|c.414A>C|p.Ile138Ile|621/2312|414/1344|138/447||WARNING_REF_DOES_NOT_MATCH_GENOME,C|synonymous_variant|LOW|ALPL|ENSG00000162551|transcript|ENST00000540617|protein_coding|5/11|c.480A>C|p.Ile160Ile|737/2428|480/1410|160/469||WARNING_REF_DOES_NOT_MATCH_GENOME,C|synonymous_variant|LOW|ALPL|ENSG00000162551|transcript|ENST00000374840|protein_coding|6/12|c.645A>C|p.Ile215Ile|895/2589|645/1575|215/524||WARNING_REF_DOES_NOT_MATCH_GENOME,C|synonymous_variant|LOW|ALPL|ENSG00000162551|transcript|ENST00000425315|protein_coding|5/11|c.645A>C|p.Ile215Ile|645/2336|645/1575|215/524||WARNING_REF_DOES_NOT_MATCH_GENOME,C|downstream_gene_variant|MODIFIER|ALPL|ENSG00000162551|transcript|ENST00000468526|processed_transcript||n.*2A>C|||||2| |
| NOTCH2 | rs1699761 | 37 | 1 | 120489416 | 120489416 | + | SNP | C | C | A | SRR8586411 | Nonsense_Mutation | 0/1:156,51:207:99:1015,0,4012 | 207;ANN=A|downstream_gene_variant|MODIFIER|NOTCH2|ENSG00000134250|transcript|ENST00000479412|retained_intron||n.*3327G>T|||||3327|,A|intron_variant|MODIFIER|NOTCH2|ENSG00000134250|transcript|ENST00000256646|protein_coding|17/33|c.2752+1621G>T|||||| |
| NOTCH2 | rs113514538 | 37 | 1 | 120489584 | 120489584 | + | SNP | T | T | G | SRR8586411 | Nonsense_Mutation | 0/1:159,30:189:99:384,0,4380 | 189;ANN=G|downstream_gene_variant|MODIFIER|NOTCH2|ENSG00000134250|transcript|ENST00000479412|retained_intron||n.*3159A>C|||||3159|,G|intron_variant|MODIFIER|NOTCH2|ENSG00000134250|transcript|ENST00000256646|protein_coding|17/33|c.2752+1453A>C|||||| |
| RP11-14N7.2-AL732363.1 | rs1064044 | 37 | 1 | 149017809 | 149017809 | + | SNP | A | A | G | SRR8586411 | Missense_Mutation | 0/1:176,91:267:99:2263,0,5402 | 267;ANN=G|intergenic_region|MODIFIER|RP11-14N7.2-AL732363.1|ENSG00000232527-ENSG00000264022|intergenic_region|ENSG00000232527-ENSG00000264022|||n.149017809A>G|||||| |
| RNU1-92P | rs11805879 | 37 | 1 | 149216812 | 149216812 | + | SNP | A | A | C | SRR8586411 | Missense_Mutation | 0/1:31,12:43:99:300,0,901 | 43;ANN=C|upstream_gene_variant|MODIFIER|RNU1-92P|ENSG00000252826|transcript|ENST00000517017|snRNA||n.-1512T>G|||||1512|,C|intergenic_region|MODIFIER|RNU1-92P-RNVU1-18|ENSG00000252826-ENSG00000206737|intergenic_region|ENSG00000252826-ENSG00000206737|||n.149216812A>C|||||| |
| MTR-RPL35P1 | rs6686489 | 37 | 1 | 237091429 | 237091429 | + | SNP | G | G | T | SRR8586411 | Missense_Mutation | 0/1:66,107:173:99:2375,0,1426 | 222;ANN=T|intergenic_region|MODIFIER|MTR-RPL35P1|ENSG00000116984-ENSG00000237991|intergenic_region|ENSG00000116984-ENSG00000237991|||n.237091429G>T|||||| |
| MTR-RPL35P1 | rs6686490 | 37 | 1 | 237091430 | 237091430 | + | SNP | G | G | T | SRR8586411 | Missense_Mutation | 0/1:50,114:164:99:2589,0,1167 | 215;ANN=T|intergenic_region|MODIFIER|MTR-RPL35P1|ENSG00000116984-ENSG00000237991|intergenic_region|ENSG00000116984-ENSG00000237991|||n.237091430G>T|||||| |
| MTR-RPL35P1 | rs368474001 | 37 | 1 | 237091437 | 237091437 | + | SNP | C | C | G | SRR8586411 | Missense_Mutation | 0/1:125,39:164:99:248,0,3404 | 164;ANN=G|intergenic_region|MODIFIER|MTR-RPL35P1|ENSG00000116984-ENSG00000237991|intergenic_region|ENSG00000116984-ENSG00000237991|||n.237091437C>G|||||| |
| RYR2 | rs34151790 | 37 | 1 | 237367594 | 237367594 | + | SNP | C | C | T | SRR8586411 | Missense_Mutation | 0/1:175,33:208:99:929,0,7151 | 208;ANN=T|intron_variant|MODIFIER|RYR2|ENSG00000198626|transcript|ENST00000366574|protein_coding|1/104|c.49-66203C>T|||||| |
| AMBRA1 | rs568863082 | 37 | 11 | 46428617 | 46428617 | + | SNP | A | G | G | SRR8586411 | Missense_Mutation | 1/1:5,102:107:99:3110,132,0 | 107;ANN=G|intron_variant|MODIFIER|AMBRA1|ENSG00000110497|transcript|ENST00000458649|protein_coding|17/17|c.3403+1446T>C||||||,G|intron_variant|MODIFIER|AMBRA1|ENSG00000110497|transcript|ENST00000298834|protein_coding|16/16|c.3223+1446T>C||||||,G|intron_variant|MODIFIER|AMBRA1|ENSG00000110497|transcript|ENST00000314845|protein_coding|18/18|c.3133+1446T>C||||||,G|intron_variant|MODIFIER|AMBRA1|ENSG00000110497|transcript|ENST00000426438|protein_coding|16/16|c.3316+1446T>C||||||,G|intron_variant|MODIFIER|AMBRA1|ENSG00000110497|transcript|ENST00000533727|protein_coding|17/17|c.3046+1446T>C||||||,G|intron_variant|MODIFIER|AMBRA1|ENSG00000110497|transcript|ENST00000534300|protein_coding|16/16|c.3223+1446T>C||||||,G|intron_variant|MODIFIER|AMBRA1|ENSG00000110497|transcript|ENST00000526545|protein_coding|2/2|c.277+1446T>C||||||WARNING_TRANSCRIPT_NO_START_CODON,G|intron_variant|MODIFIER|AMBRA1|ENSG00000110497|transcript|ENST00000528950|protein_coding|16/16|c.3316+1446T>C|||||| |
| ZBTB3 | rs12801348 | 37 | 11 | 62520659 | 62520659 | + | SNP | T | T | C | SRR8586411 | Nonsense_Mutation | 0/1:939,97:1036:99:1206,0,26460 | 1036;ANN=C|synonymous_variant|LOW|ZBTB3|ENSG00000185670|transcript|ENST00000394807|protein_coding|2/2|c.628A>G|p.Ala210Ala|754/2978|628/1725|210/574||WARNING_REF_DOES_NOT_MATCH_GENOME,C|upstream_gene_variant|MODIFIER|ZBTB3|ENSG00000185670|transcript|ENST00000530112|nonsense_mediated_decay||c.-1089A>G|||||1088|WARNING_TRANSCRIPT_NO_START_CODON,C|downstream_gene_variant|MODIFIER|ZBTB3|ENSG00000185670|transcript|ENST00000527994|protein_coding||c.*131A>G|||||131|WARNING_TRANSCRIPT_INCOMPLETE |
| ZBTB3 | rs12796082 | 37 | 11 | 62520661 | 62520661 | + | SNP | A | A | G | SRR8586411 | Nonsense_Mutation | 0/1:935,94:1029:74:74,0,46704 | 1029;ANN=G|synonymous_variant|LOW|ZBTB3|ENSG00000185670|transcript|ENST00000394807|protein_coding|2/2|c.626T>C|p.Ser209Ser|752/2978|626/1725|209/574||WARNING_REF_DOES_NOT_MATCH_GENOME,G|upstream_gene_variant|MODIFIER|ZBTB3|ENSG00000185670|transcript|ENST00000530112|nonsense_mediated_decay||c.-1091T>C|||||1090|WARNING_TRANSCRIPT_NO_START_CODON,G|downstream_gene_variant|MODIFIER|ZBTB3|ENSG00000185670|transcript|ENST00000527994|protein_coding||c.*129T>C|||||129|WARNING_TRANSCRIPT_INCOMPLETE |
| POLR2G | rs543137707 | 37 | 11 | 62528461 | 62528461 | + | SNP | T | T | C | SRR8586411 | Missense_Mutation | 0/1:1161,130:1291:99:1414,0,31113 | 1331;ANN=C|upstream_gene_variant|MODIFIER|POLR2G|ENSG00000168002|transcript|ENST00000301788|protein_coding||c.-660T>C|||||555|,C|upstream_gene_variant|MODIFIER|POLR2G|ENSG00000168002|transcript|ENST00000533442|protein_coding||c.-4220T>C|||||576|WARNING_TRANSCRIPT_INCOMPLETE,C|upstream_gene_variant|MODIFIER|POLR2G|ENSG00000168002|transcript|ENST00000525455|nonsense_mediated_decay||c.-660T>C|||||591|,C|upstream_gene_variant|MODIFIER|POLR2G|ENSG00000168002|transcript|ENST00000524819|nonsense_mediated_decay||c.-660T>C|||||594|,C|upstream_gene_variant|MODIFIER|POLR2G|ENSG00000168002|transcript|ENST00000531944|nonsense_mediated_decay||c.-660T>C|||||628|,C|upstream_gene_variant|MODIFIER|POLR2G|ENSG00000168002|transcript|ENST00000527435|retained_intron||n.-648T>C|||||648|,C|upstream_gene_variant|MODIFIER|POLR2G|ENSG00000168002|transcript|ENST00000531996|nonsense_mediated_decay||c.-806T>C|||||806|WARNING_TRANSCRIPT_NO_START_CODON,C|upstream_gene_variant|MODIFIER|POLR2G|ENSG00000168002|transcript|ENST00000526368|retained_intron||n.-1765T>C|||||1765|,C|intergenic_region|MODIFIER|ZBTB3-POLR2G|ENSG00000185670-ENSG00000168002|intergenic_region|ENSG00000185670-ENSG00000168002|||n.62528461T>C|||||| |
| C11orf80 | rs13897 | 37 | 11 | 66563698 | 66563698 | + | SNP | C | C | T | SRR8586411 | Missense_Mutation | 0/1:36,36:72:99:1042,0,890 | 72;ANN=T|intron_variant|MODIFIER|C11orf80|ENSG00000173715|transcript|ENST00000360962|protein_coding|5/16|c.634-54C>T||||||,T|intron_variant|MODIFIER|C11orf80|ENSG00000173715|transcript|ENST00000524551|protein_coding|2/5|c.-24-54C>T||||||WARNING_TRANSCRIPT_INCOMPLETE,T|intron_variant|MODIFIER|C11orf80|ENSG00000173715|transcript|ENST00000525908|protein_coding|5/8|c.487-54C>T||||||WARNING_TRANSCRIPT_INCOMPLETE,T|intron_variant|MODIFIER|C11orf80|ENSG00000173715|transcript|ENST00000346672|protein_coding|5/15|c.169-54C>T||||||,T|intron_variant|MODIFIER|C11orf80|ENSG00000173715|transcript|ENST00000527634|protein_coding|2/13|c.-24-54C>T||||||,T|intron_variant|MODIFIER|C11orf80|ENSG00000173715|transcript|ENST00000527368|processed_transcript|4/5|n.683-54C>T||||||,T|intron_variant|MODIFIER|C11orf80|ENSG00000173715|transcript|ENST00000540737|protein_coding|3/14|c.136-54C>T||||||,T|intron_variant|MODIFIER|C11orf80|ENSG00000173715|transcript|ENST00000531400|nonsense_mediated_decay|3/4|c.136-4414C>T||||||WARNING_TRANSCRIPT_INCOMPLETE,T|intron_variant|MODIFIER|C11orf80|ENSG00000173715|transcript|ENST00000532565|protein_coding|5/16|c.-24-54C>T||||||,T|intron_variant|MODIFIER|C11orf80|ENSG00000173715|transcript|ENST00000525449|protein_coding|4/14|c.169-54C>T||||||,T|intron_variant|MODIFIER|C11orf80|ENSG00000173715|transcript|ENST00000532727|nonsense_mediated_decay|2/13|c.140-54C>T||||||,T|intron_variant|MODIFIER|C11orf80|ENSG00000173715|transcript|ENST00000526260|nonsense_mediated_decay|1/4|c.109-54C>T||||||WARNING_TRANSCRIPT_NO_START_CODON,T|intron_variant|MODIFIER|C11orf80|ENSG00000173715|transcript|ENST00000532089|protein_coding|1/3|c.109-54C>T||||||WARNING_TRANSCRIPT_INCOMPLETE,T|non_coding_transcript_exon_variant|MODIFIER|C11orf80|ENSG00000173715|transcript|ENST00000527352|retained_intron|1/4|n.331C>T||||||WARNING_REF_DOES_NOT_MATCH_GENOME |
| CACNA1C | rs527407680 | 37 | 12 | 2232253 | 2232253 | + | SNP | G | G | T | SRR8586411 | Missense_Mutation | 0/1:62,9:71:43:43,0,2757 | 71;ANN=T|intron_variant|MODIFIER|CACNA1C|ENSG00000151067|transcript|ENST00000399634|protein_coding|3/47|c.477+2657G>T||||||,T|intron_variant|MODIFIER|CACNA1C|ENSG00000151067|transcript|ENST00000335762|protein_coding|3/47|c.477+2657G>T||||||,T|intron_variant|MODIFIER|CACNA1C|ENSG00000151067|transcript|ENST00000399655|protein_coding|3/46|c.477+2657G>T||||||,T|intron_variant|MODIFIER|CACNA1C|ENSG00000151067|transcript|ENST00000480911|protein_coding|3/28|c.477+2657G>T||||||,T|intron_variant|MODIFIER|CACNA1C|ENSG00000151067|transcript|ENST00000327702|protein_coding|3/47|c.477+2657G>T||||||,T|intron_variant|MODIFIER|CACNA1C|ENSG00000151067|transcript|ENST00000344100|protein_coding|3/46|c.477+2657G>T||||||,T|intron_variant|MODIFIER|CACNA1C|ENSG00000151067|transcript|ENST00000347598|protein_coding|3/48|c.477+2657G>T||||||,T|intron_variant|MODIFIER|CACNA1C|ENSG00000151067|transcript|ENST00000399591|protein_coding|3/45|c.477+2657G>T||||||,T|intron_variant|MODIFIER|CACNA1C|ENSG00000151067|transcript|ENST00000399595|protein_coding|3/45|c.477+2657G>T||||||,T|intron_variant|MODIFIER|CACNA1C|ENSG00000151067|transcript|ENST00000399597|protein_coding|3/46|c.477+2657G>T||||||,T|intron_variant|MODIFIER|CACNA1C|ENSG00000151067|transcript|ENST00000399601|protein_coding|3/46|c.477+2657G>T||||||,T|intron_variant|MODIFIER|CACNA1C|ENSG00000151067|transcript|ENST00000399606|protein_coding|3/47|c.477+2657G>T||||||,T|intron_variant|MODIFIER|CACNA1C|ENSG00000151067|transcript|ENST00000399621|protein_coding|3/46|c.477+2657G>T||||||,T|intron_variant|MODIFIER|CACNA1C|ENSG00000151067|transcript|ENST00000399629|protein_coding|3/46|c.477+2657G>T||||||,T|intron_variant|MODIFIER|CACNA1C|ENSG00000151067|transcript|ENST00000399637|protein_coding|3/46|c.477+2657G>T||||||,T|intron_variant|MODIFIER|CACNA1C|ENSG00000151067|transcript|ENST00000399638|protein_coding|3/47|c.477+2657G>T||||||,T|intron_variant|MODIFIER|CACNA1C|ENSG00000151067|transcript|ENST00000399641|protein_coding|3/46|c.477+2657G>T||||||,T|intron_variant|MODIFIER|CACNA1C|ENSG00000151067|transcript|ENST00000399644|protein_coding|3/46|c.477+2657G>T||||||,T|intron_variant|MODIFIER|CACNA1C|ENSG00000151067|transcript|ENST00000399649|protein_coding|3/45|c.477+2657G>T||||||,T|intron_variant|MODIFIER|CACNA1C|ENSG00000151067|transcript|ENST00000402845|protein_coding|3/46|c.477+2657G>T||||||,T|intron_variant|MODIFIER|CACNA1C|ENSG00000151067|transcript|ENST00000399603|protein_coding|3/46|c.477+2657G>T||||||,T|intron_variant|MODIFIER|CACNA1C|ENSG00000151067|transcript|ENST00000399617|protein_coding|3/47|c.477+2657G>T||||||,T|intron_variant|MODIFIER|CACNA1C|ENSG00000151067|transcript|ENST00000406454|protein_coding|3/47|c.477+2657G>T|||||| |
| TSPAN9 | rs58837502 | 37 | 12 | 3211762 | 3211762 | + | SNP | T | T | C | SRR8586411 | Missense_Mutation | 0/1:29,92:121:99:2713,0,525 | 121;ANN=C|intron_variant|MODIFIER|TSPAN9|ENSG00000011105|transcript|ENST00000537971|protein_coding|1/7|c.-18+25143T>C||||||,C|intron_variant|MODIFIER|TSPAN9|ENSG00000011105|transcript|ENST00000444315|nonsense_mediated_decay|2/5|c.-18+18877T>C||||||,C|intron_variant|MODIFIER|TSPAN9|ENSG00000011105|transcript|ENST00000011898|protein_coding|2/8|c.-18+18877T>C|||||| |
| LINC00935 | rs1057548 | 37 | 12 | 49128822 | 49128822 | + | SNP | C | T | T | SRR8586411 | Missense_Mutation | 1/1:17,292:309:99:8569,160,0 | 310;ANN=T|intron_variant|MODIFIER|LINC00935|ENSG00000257987|transcript|ENST00000548380|protein_coding|1/3|c.79+7480C>T||||||,T|intron_variant|MODIFIER|LINC00935|ENSG00000257987|transcript|ENST00000548054|nonsense_mediated_decay|1/4|c.80-1049C>T|||||| |
| ADCY6 | rs566021671 | 37 | 12 | 49185874 | 49185874 | + | SNP | C | C | T | SRR8586411 | Missense_Mutation | 0/1:232,20:252:99:123,0,9624 | 252;ANN=T|upstream_gene_variant|MODIFIER|ADCY6|ENSG00000174233|transcript|ENST00000550422|protein_coding||c.-8657G>A|||||3054|,T|downstream_gene_variant|MODIFIER|RP11-579D7.4|ENSG00000257660|transcript|ENST00000549864|lincRNA||n.*1556C>T|||||1556|,T|downstream_gene_variant|MODIFIER|RP11-579D7.4|ENSG00000257660|transcript|ENST00000547774|lincRNA||n.*1556C>T|||||1556|,T|intergenic_region|MODIFIER|RP11-579D7.4-RP11-579D7.8|ENSG00000257660-ENSG00000271547|intergenic_region|ENSG00000257660-ENSG00000271547|||n.49185874C>T|||||| |
| PRMT5-AS1 | rs370121893 | 37 | 14 | 23386023 | 23386023 | + | SNP | G | G | A | SRR8586411 | Missense_Mutation | 0/1:133,30:163:99:654,0,3506 | 164;ANN=A|upstream_gene_variant|MODIFIER|PRMT5-AS1|ENSG00000237054|transcript|ENST00000424245|antisense||n.-2642G>A|||||2642|,A|upstream_gene_variant|MODIFIER|PRMT5-AS1|ENSG00000237054|transcript|ENST00000609885|antisense||n.-2807G>A|||||2807|,A|upstream_gene_variant|MODIFIER|PRMT5-AS1|ENSG00000237054|transcript|ENST00000457443|antisense||n.-3825G>A|||||3825|,A|upstream_gene_variant|MODIFIER|PRMT5-AS1|ENSG00000237054|transcript|ENST00000599580|antisense||n.-4097G>A|||||4097|,A|upstream_gene_variant|MODIFIER|PRMT5-AS1|ENSG00000237054|transcript|ENST00000595662|antisense||n.-4224G>A|||||4224|,A|upstream_gene_variant|MODIFIER|PRMT5-AS1|ENSG00000237054|transcript|ENST00000587245|antisense||n.-4224G>A|||||4224|,A|upstream_gene_variant|MODIFIER|PRMT5-AS1|ENSG00000237054|transcript|ENST00000590290|antisense||n.-4227G>A|||||4227|,A|downstream_gene_variant|MODIFIER|PRMT5|ENSG00000100462|transcript|ENST00000324366|protein_coding||c.*4090C>T|||||3697|,A|downstream_gene_variant|MODIFIER|RBM23|ENSG00000100461|transcript|ENST00000553777|processed_transcript||n.*750C>T|||||750|,A|downstream_gene_variant|MODIFIER|PRMT5|ENSG00000100462|transcript|ENST00000397441|protein_coding||c.*4090C>T|||||3711|,A|downstream_gene_variant|MODIFIER|PRMT5|ENSG00000100462|transcript|ENST00000557443|protein_coding||c.*3867C>T|||||3715|WARNING_TRANSCRIPT_NO_START_CODON,A|downstream_gene_variant|MODIFIER|PRMT5|ENSG00000100462|transcript|ENST00000476175|processed_transcript||n.*3715C>T|||||3715|,A|downstream_gene_variant|MODIFIER|PRMT5|ENSG00000100462|transcript|ENST00000397440|protein_coding||c.*4090C>T|||||3715|,A|downstream_gene_variant|MODIFIER|PRMT5|ENSG00000100462|transcript|ENST00000216350|protein_coding||c.*4090C>T|||||3716|,A|downstream_gene_variant|MODIFIER|PRMT5|ENSG00000100462|transcript|ENST00000555454|protein_coding||c.*4233C>T|||||3996|WARNING_TRANSCRIPT_NO_START_CODON,A|downstream_gene_variant|MODIFIER|PRMT5|ENSG00000100462|transcript|ENST00000454731|protein_coding||c.*4090C>T|||||4011|WARNING_TRANSCRIPT_NO_START_CODON,A|downstream_gene_variant|MODIFIER|PRMT5|ENSG00000100462|transcript|ENST00000553915|nonsense_mediated_decay||c.*10782C>T|||||4043|,A|downstream_gene_variant|MODIFIER|PRMT5|ENSG00000100462|transcript|ENST00000538452|protein_coding||c.*4090C>T|||||4043|,A|downstream_gene_variant|MODIFIER|PRMT5|ENSG00000100462|transcript|ENST00000553897|protein_coding||c.*4090C>T|||||4062|,A|intron_variant|MODIFIER|RBM23|ENSG00000100461|transcript|ENST00000359890|protein_coding|1/13|c.-11+2185C>T||||||,A|intron_variant|MODIFIER|RBM23|ENSG00000100461|transcript|ENST00000555209|protein_coding|1/10|c.-493+2185C>T||||||,A|intron_variant|MODIFIER|RBM23|ENSG00000100461|transcript|ENST00000557667|retained_intron|1/13|n.138+2185C>T||||||,A|intron_variant|MODIFIER|RBM23|ENSG00000100461|transcript|ENST00000399922|protein_coding|1/12|c.-11+2185C>T||||||,A|intron_variant|MODIFIER|RBM23|ENSG00000100461|transcript|ENST00000346528|protein_coding|1/11|c.-11+2185C>T||||||,A|intron_variant|MODIFIER|RBM23|ENSG00000100461|transcript|ENST00000542016|protein_coding|1/11|c.-360+2185C>T||||||,A|intron_variant|MODIFIER|RBM23|ENSG00000100461|transcript|ENST00000307814|retained_intron|1/10|n.151+2185C>T||||||,A|intron_variant|MODIFIER|RBM23|ENSG00000100461|transcript|ENST00000557403|protein_coding|1/5|c.-343+2185C>T||||||WARNING_TRANSCRIPT_INCOMPLETE,A|intron_variant|MODIFIER|RBM23|ENSG00000100461|transcript|ENST00000555722|protein_coding|1/4|c.-284+2185C>T||||||WARNING_TRANSCRIPT_INCOMPLETE,A|intron_variant|MODIFIER|RBM23|ENSG00000100461|transcript|ENST00000553920|processed_transcript|1/4|n.138+2185C>T||||||,A|intron_variant|MODIFIER|RBM23|ENSG00000100461|transcript|ENST00000557227|protein_coding|1/3|c.-306+2185C>T||||||WARNING_TRANSCRIPT_NO_STOP_CODON,A|intron_variant|MODIFIER|RBM23|ENSG00000100461|transcript|ENST00000555714|processed_transcript|1/5|n.151+2185C>T||||||,A|intron_variant|MODIFIER|RBM23|ENSG00000100461|transcript|ENST00000554256|protein_coding|1/5|c.-11+2185C>T||||||WARNING_TRANSCRIPT_NO_STOP_CODON,A|intron_variant|MODIFIER|RBM23|ENSG00000100461|transcript|ENST00000556687|retained_intron|1/3|n.151+2185C>T||||||,A|intron_variant|MODIFIER|RBM23|ENSG00000100461|transcript|ENST00000553902|retained_intron|1/2|n.151+2185C>T||||||,A|intron_variant|MODIFIER|RBM23|ENSG00000100461|transcript|ENST00000554955|retained_intron|1/4|n.112+2185C>T||||||,A|intron_variant|MODIFIER|RBM23|ENSG00000100461|transcript|ENST00000556984|processed_transcript|1/4|n.151+2185C>T||||||,A|intron_variant|MODIFIER|RBM23|ENSG00000100461|transcript|ENST00000557549|protein_coding|1/4|c.-129+2185C>T||||||WARNING_TRANSCRIPT_NO_STOP_CODON,A|intron_variant|MODIFIER|RBM23|ENSG00000100461|transcript|ENST00000555676|protein_coding|1/4|c.-133+2185C>T||||||WARNING_TRANSCRIPT_NO_STOP_CODON,A|intron_variant|MODIFIER|RBM23|ENSG00000100461|transcript|ENST00000557571|protein_coding|2/4|c.-11+214C>T||||||WARNING_TRANSCRIPT_NO_STOP_CODON,A|intron_variant|MODIFIER|RBM23|ENSG00000100461|transcript|ENST00000556862|protein_coding|1/5|c.-133+2185C>T||||||WARNING_TRANSCRIPT_INCOMPLETE,A|intron_variant|MODIFIER|RBM23|ENSG00000100461|transcript|ENST00000557464|protein_coding|1/4|c.-109+2185C>T||||||WARNING_TRANSCRIPT_NO_STOP_CODON,A|intron_variant|MODIFIER|RBM23|ENSG00000100461|transcript|ENST00000554618|protein_coding|1/5|c.-211+2185C>T||||||WARNING_TRANSCRIPT_INCOMPLETE,A|intron_variant|MODIFIER|RBM23|ENSG00000100461|transcript|ENST00000556365|processed_transcript|1/3|n.151+2185C>T||||||,A|intron_variant|MODIFIER|RBM23|ENSG00000100461|transcript|ENST00000553876|protein_coding|1/2|c.-11+665C>T||||||WARNING_TRANSCRIPT_NO_STOP_CODON |
| PRMT5-AS1 | rs374702183 | 37 | 14 | 23386579 | 23386579 | + | SNP | T | T | C | SRR8586411 | Missense_Mutation | 0/1:75,277:352:99:8275,0,1103 | 352;ANN=C|upstream_gene_variant|MODIFIER|PRMT5-AS1|ENSG00000237054|transcript|ENST00000424245|antisense||n.-2086T>C|||||2086|,C|upstream_gene_variant|MODIFIER|PRMT5-AS1|ENSG00000237054|transcript|ENST00000609885|antisense||n.-2251T>C|||||2251|,C|upstream_gene_variant|MODIFIER|PRMT5-AS1|ENSG00000237054|transcript|ENST00000457443|antisense||n.-3269T>C|||||3269|,C|upstream_gene_variant|MODIFIER|PRMT5-AS1|ENSG00000237054|transcript|ENST00000599580|antisense||n.-3541T>C|||||3541|,C|upstream_gene_variant|MODIFIER|PRMT5-AS1|ENSG00000237054|transcript|ENST00000595662|antisense||n.-3668T>C|||||3668|,C|upstream_gene_variant|MODIFIER|PRMT5-AS1|ENSG00000237054|transcript|ENST00000587245|antisense||n.-3668T>C|||||3668|,C|upstream_gene_variant|MODIFIER|PRMT5-AS1|ENSG00000237054|transcript|ENST00000590290|antisense||n.-3671T>C|||||3671|,C|downstream_gene_variant|MODIFIER|PRMT5|ENSG00000100462|transcript|ENST00000324366|protein_coding||c.*3534A>G|||||3141|,C|downstream_gene_variant|MODIFIER|RBM23|ENSG00000100461|transcript|ENST00000553777|processed_transcript||n.*194A>G|||||194|,C|downstream_gene_variant|MODIFIER|PRMT5|ENSG00000100462|transcript|ENST00000397441|protein_coding||c.*3534A>G|||||3155|,C|downstream_gene_variant|MODIFIER|PRMT5|ENSG00000100462|transcript|ENST00000557443|protein_coding||c.*3311A>G|||||3159|WARNING_TRANSCRIPT_NO_START_CODON,C|downstream_gene_variant|MODIFIER|PRMT5|ENSG00000100462|transcript|ENST00000476175|processed_transcript||n.*3159A>G|||||3159|,C|downstream_gene_variant|MODIFIER|PRMT5|ENSG00000100462|transcript|ENST00000397440|protein_coding||c.*3534A>G|||||3159|,C|downstream_gene_variant|MODIFIER|PRMT5|ENSG00000100462|transcript|ENST00000216350|protein_coding||c.*3534A>G|||||3160|,C|downstream_gene_variant|MODIFIER|PRMT5|ENSG00000100462|transcript|ENST00000555454|protein_coding||c.*3677A>G|||||3440|WARNING_TRANSCRIPT_NO_START_CODON,C|downstream_gene_variant|MODIFIER|PRMT5|ENSG00000100462|transcript|ENST00000454731|protein_coding||c.*3534A>G|||||3455|WARNING_TRANSCRIPT_NO_START_CODON,C|downstream_gene_variant|MODIFIER|PRMT5|ENSG00000100462|transcript|ENST00000553915|nonsense_mediated_decay||c.*10226A>G|||||3487|,C|downstream_gene_variant|MODIFIER|PRMT5|ENSG00000100462|transcript|ENST00000538452|protein_coding||c.*3534A>G|||||3487|,C|downstream_gene_variant|MODIFIER|PRMT5|ENSG00000100462|transcript|ENST00000553897|protein_coding||c.*3534A>G|||||3506|,C|downstream_gene_variant|MODIFIER|PRMT5|ENSG00000100462|transcript|ENST00000557758|retained_intron||n.*4762A>G|||||4762|,C|intron_variant|MODIFIER|RBM23|ENSG00000100461|transcript|ENST00000359890|protein_coding|1/13|c.-11+1629A>G||||||,C|intron_variant|MODIFIER|RBM23|ENSG00000100461|transcript|ENST00000555209|protein_coding|1/10|c.-493+1629A>G||||||,C|intron_variant|MODIFIER|RBM23|ENSG00000100461|transcript|ENST00000557667|retained_intron|1/13|n.138+1629A>G||||||,C|intron_variant|MODIFIER|RBM23|ENSG00000100461|transcript|ENST00000399922|protein_coding|1/12|c.-11+1629A>G||||||,C|intron_variant|MODIFIER|RBM23|ENSG00000100461|transcript|ENST00000346528|protein_coding|1/11|c.-11+1629A>G||||||,C|intron_variant|MODIFIER|RBM23|ENSG00000100461|transcript|ENST00000542016|protein_coding|1/11|c.-360+1629A>G||||||,C|intron_variant|MODIFIER|RBM23|ENSG00000100461|transcript|ENST00000307814|retained_intron|1/10|n.151+1629A>G||||||,C|intron_variant|MODIFIER|RBM23|ENSG00000100461|transcript|ENST00000557403|protein_coding|1/5|c.-343+1629A>G||||||WARNING_TRANSCRIPT_INCOMPLETE,C|intron_variant|MODIFIER|RBM23|ENSG00000100461|transcript|ENST00000555722|protein_coding|1/4|c.-284+1629A>G||||||WARNING_TRANSCRIPT_INCOMPLETE,C|intron_variant|MODIFIER|RBM23|ENSG00000100461|transcript|ENST00000553920|processed_transcript|1/4|n.138+1629A>G||||||,C|intron_variant|MODIFIER|RBM23|ENSG00000100461|transcript|ENST00000557227|protein_coding|1/3|c.-306+1629A>G||||||WARNING_TRANSCRIPT_NO_STOP_CODON,C|intron_variant|MODIFIER|RBM23|ENSG00000100461|transcript|ENST00000555714|processed_transcript|1/5|n.151+1629A>G||||||,C|intron_variant|MODIFIER|RBM23|ENSG00000100461|transcript|ENST00000554256|protein_coding|1/5|c.-11+1629A>G||||||WARNING_TRANSCRIPT_NO_STOP_CODON,C|intron_variant|MODIFIER|RBM23|ENSG00000100461|transcript|ENST00000556687|retained_intron|1/3|n.151+1629A>G||||||,C|intron_variant|MODIFIER|RBM23|ENSG00000100461|transcript|ENST00000553902|retained_intron|1/2|n.151+1629A>G||||||,C|intron_variant|MODIFIER|RBM23|ENSG00000100461|transcript|ENST00000554955|retained_intron|1/4|n.112+1629A>G||||||,C|intron_variant|MODIFIER|RBM23|ENSG00000100461|transcript|ENST00000556984|processed_transcript|1/4|n.151+1629A>G||||||,C|intron_variant|MODIFIER|RBM23|ENSG00000100461|transcript|ENST00000557549|protein_coding|1/4|c.-129+1629A>G||||||WARNING_TRANSCRIPT_NO_STOP_CODON,C|intron_variant|MODIFIER|RBM23|ENSG00000100461|transcript|ENST00000555676|protein_coding|1/4|c.-133+1629A>G||||||WARNING_TRANSCRIPT_NO_STOP_CODON,C|intron_variant|MODIFIER|RBM23|ENSG00000100461|transcript|ENST00000557571|protein_coding|1/4|c.-149-204A>G||||||WARNING_TRANSCRIPT_NO_STOP_CODON,C|intron_variant|MODIFIER|RBM23|ENSG00000100461|transcript|ENST00000556862|protein_coding|1/5|c.-133+1629A>G||||||WARNING_TRANSCRIPT_INCOMPLETE,C|intron_variant|MODIFIER|RBM23|ENSG00000100461|transcript|ENST00000557464|protein_coding|1/4|c.-109+1629A>G||||||WARNING_TRANSCRIPT_NO_STOP_CODON,C|intron_variant|MODIFIER|RBM23|ENSG00000100461|transcript|ENST00000554618|protein_coding|1/5|c.-211+1629A>G||||||WARNING_TRANSCRIPT_INCOMPLETE,C|intron_variant|MODIFIER|RBM23|ENSG00000100461|transcript|ENST00000556365|processed_transcript|1/3|n.151+1629A>G||||||,C|intron_variant|MODIFIER|RBM23|ENSG00000100461|transcript|ENST00000553876|protein_coding|1/2|c.-11+109A>G||||||WARNING_TRANSCRIPT_NO_STOP_CODON |
| RBM23 | rs780653498 | 37 | 14 | 23388975 | 23388975 | + | SNP | T | T | C | SRR8586411 | Missense_Mutation | 0/1:168,144:312:99:3822,0,4065 | 312;ANN=C|upstream_gene_variant|MODIFIER|RBM23|ENSG00000100461|transcript|ENST00000359890|protein_coding||c.-8373A>G|||||582|,C|upstream_gene_variant|MODIFIER|RBM23|ENSG00000100461|transcript|ENST00000555209|protein_coding||c.-14778A>G|||||637|,C|upstream_gene_variant|MODIFIER|RBM23|ENSG00000100461|transcript|ENST00000557667|retained_intron||n.-630A>G|||||630|,C|upstream_gene_variant|MODIFIER|RBM23|ENSG00000100461|transcript|ENST00000399922|protein_coding||c.-8373A>G|||||584|,C|upstream_gene_variant|MODIFIER|RBM23|ENSG00000100461|transcript|ENST00000346528|protein_coding||c.-8373A>G|||||588|,C|upstream_gene_variant|MODIFIER|RBM23|ENSG00000100461|transcript|ENST00000542016|protein_coding||c.-14368A>G|||||588|,C|upstream_gene_variant|MODIFIER|RBM23|ENSG00000100461|transcript|ENST00000307814|retained_intron||n.-617A>G|||||617|,C|upstream_gene_variant|MODIFIER|RBM23|ENSG00000100461|transcript|ENST00000557403|protein_coding||c.-14368A>G|||||628|WARNING_TRANSCRIPT_INCOMPLETE,C|upstream_gene_variant|MODIFIER|RBM23|ENSG00000100461|transcript|ENST00000555722|protein_coding||c.-14368A>G|||||601|WARNING_TRANSCRIPT_INCOMPLETE,C|upstream_gene_variant|MODIFIER|RBM23|ENSG00000100461|transcript|ENST00000553920|processed_transcript||n.-630A>G|||||630|,C|upstream_gene_variant|MODIFIER|RBM23|ENSG00000100461|transcript|ENST00000557227|protein_coding||c.-14368A>G|||||584|WARNING_TRANSCRIPT_NO_STOP_CODON,C|upstream_gene_variant|MODIFIER|RBM23|ENSG00000100461|transcript|ENST00000555714|processed_transcript||n.-617A>G|||||617|,C|upstream_gene_variant|MODIFIER|RBM23|ENSG00000100461|transcript|ENST00000554256|protein_coding||c.-8373A>G|||||629|WARNING_TRANSCRIPT_NO_STOP_CODON,C|upstream_gene_variant|MODIFIER|RBM23|ENSG00000100461|transcript|ENST00000556687|retained_intron||n.-617A>G|||||617|,C|upstream_gene_variant|MODIFIER|RBM23|ENSG00000100461|transcript|ENST00000553902|retained_intron||n.-617A>G|||||617|,C|upstream_gene_variant|MODIFIER|RBM23|ENSG00000100461|transcript|ENST00000554955|retained_intron||n.-656A>G|||||656|,C|upstream_gene_variant|MODIFIER|RBM23|ENSG00000100461|transcript|ENST00000556984|processed_transcript||n.-617A>G|||||617|,C|upstream_gene_variant|MODIFIER|RBM23|ENSG00000100461|transcript|ENST00000557549|protein_coding||c.-8373A>G|||||617|WARNING_TRANSCRIPT_NO_STOP_CODON,C|upstream_gene_variant|MODIFIER|RBM23|ENSG00000100461|transcript|ENST00000555676|protein_coding||c.-8373A>G|||||617|WARNING_TRANSCRIPT_NO_STOP_CODON,C|upstream_gene_variant|MODIFIER|RBM23|ENSG00000100461|transcript|ENST00000557571|protein_coding||c.-8373A>G|||||617|WARNING_TRANSCRIPT_NO_STOP_CODON,C|upstream_gene_variant|MODIFIER|RBM23|ENSG00000100461|transcript|ENST00000556862|protein_coding||c.-8373A>G|||||609|WARNING_TRANSCRIPT_INCOMPLETE,C|upstream_gene_variant|MODIFIER|RBM23|ENSG00000100461|transcript|ENST00000557464|protein_coding||c.-8373A>G|||||617|WARNING_TRANSCRIPT_NO_STOP_CODON,C|upstream_gene_variant|MODIFIER|RBM23|ENSG00000100461|transcript|ENST00000554618|protein_coding||c.-8373A>G|||||617|WARNING_TRANSCRIPT_INCOMPLETE,C|upstream_gene_variant|MODIFIER|RBM23|ENSG00000100461|transcript|ENST00000556365|processed_transcript||n.-617A>G|||||617|,C|upstream_gene_variant|MODIFIER|RBM23|ENSG00000100461|transcript|ENST00000553876|protein_coding||c.-8373A>G|||||2210|WARNING_TRANSCRIPT_NO_STOP_CODON,C|upstream_gene_variant|MODIFIER|RBM23|ENSG00000100461|transcript|ENST00000553777|processed_transcript||n.-679A>G|||||679|,C|upstream_gene_variant|MODIFIER|PRMT5-AS1|ENSG00000237054|transcript|ENST00000457443|antisense||n.-873T>C|||||873|,C|upstream_gene_variant|MODIFIER|PRMT5-AS1|ENSG00000237054|transcript|ENST00000599580|antisense||n.-1145T>C|||||1145|,C|upstream_gene_variant|MODIFIER|PRMT5-AS1|ENSG00000237054|transcript|ENST00000595662|antisense||n.-1272T>C|||||1272|,C|upstream_gene_variant|MODIFIER|PRMT5-AS1|ENSG00000237054|transcript|ENST00000587245|antisense||n.-1272T>C|||||1272|,C|upstream_gene_variant|MODIFIER|PRMT5-AS1|ENSG00000237054|transcript|ENST00000590290|antisense||n.-1275T>C|||||1275|,C|downstream_gene_variant|MODIFIER|PRMT5|ENSG00000100462|transcript|ENST00000324366|protein_coding||c.*1138A>G|||||745|,C|downstream_gene_variant|MODIFIER|PRMT5|ENSG00000100462|transcript|ENST00000397441|protein_coding||c.*1138A>G|||||759|,C|downstream_gene_variant|MODIFIER|PRMT5|ENSG00000100462|transcript|ENST00000557443|protein_coding||c.*915A>G|||||763|WARNING_TRANSCRIPT_NO_START_CODON,C|downstream_gene_variant|MODIFIER|PRMT5|ENSG00000100462|transcript|ENST00000476175|processed_transcript||n.*763A>G|||||763|,C|downstream_gene_variant|MODIFIER|PRMT5|ENSG00000100462|transcript|ENST00000397440|protein_coding||c.*1138A>G|||||763|,C|downstream_gene_variant|MODIFIER|PRMT5|ENSG00000100462|transcript|ENST00000216350|protein_coding||c.*1138A>G|||||764|,C|downstream_gene_variant|MODIFIER|PRMT5|ENSG00000100462|transcript|ENST00000555454|protein_coding||c.*1281A>G|||||1044|WARNING_TRANSCRIPT_NO_START_CODON,C|downstream_gene_variant|MODIFIER|PRMT5|ENSG00000100462|transcript|ENST00000454731|protein_coding||c.*1138A>G|||||1059|WARNING_TRANSCRIPT_NO_START_CODON,C|downstream_gene_variant|MODIFIER|PRMT5|ENSG00000100462|transcript|ENST00000553915|nonsense_mediated_decay||c.*7830A>G|||||1091|,C|downstream_gene_variant|MODIFIER|PRMT5|ENSG00000100462|transcript|ENST00000538452|protein_coding||c.*1138A>G|||||1091|,C|downstream_gene_variant|MODIFIER|PRMT5|ENSG00000100462|transcript|ENST00000553897|protein_coding||c.*1138A>G|||||1110|,C|downstream_gene_variant|MODIFIER|PRMT5|ENSG00000100462|transcript|ENST00000557758|retained_intron||n.*2366A>G|||||2366|,C|downstream_gene_variant|MODIFIER|PRMT5|ENSG00000100462|transcript|ENST00000553502|protein_coding||c.*4396A>G|||||4396|WARNING_TRANSCRIPT_NO_START_CODON,C|downstream_gene_variant|MODIFIER|PRMT5|ENSG00000100462|transcript|ENST00000555530|protein_coding||c.*4551A>G|||||4551|WARNING_TRANSCRIPT_NO_START_CODON,C|downstream_gene_variant|MODIFIER|PRMT5|ENSG00000100462|transcript|ENST00000556043|protein_coding||c.*4733A>G|||||4733|WARNING_TRANSCRIPT_NO_STOP_CODON,C|downstream_gene_variant|MODIFIER|PRMT5|ENSG00000100462|transcript|ENST00000553550|protein_coding||c.*4872A>G|||||4872|WARNING_TRANSCRIPT_NO_STOP_CODON,C|downstream_gene_variant|MODIFIER|PRMT5|ENSG00000100462|transcript|ENST00000554716|processed_transcript||n.*4897A>G|||||4897|,C|non_coding_transcript_exon_variant|MODIFIER|PRMT5-AS1|ENSG00000237054|transcript|ENST00000424245|antisense|1/2|n.311T>C||||||,C|non_coding_transcript_exon_variant|MODIFIER|PRMT5-AS1|ENSG00000237054|transcript|ENST00000609885|antisense|1/2|n.146T>C|||||| |
| RP11-298I3.1 | rs765316931 | 37 | 14 | 23397004 | 23397004 | + | SNP | C | C | T | SRR8586411 | Missense_Mutation | 0/1:68,302:370:99:9533,0,1006 | 370;ANN=T|upstream_gene_variant|MODIFIER|RP11-298I3.1|ENSG00000257285|transcript|ENST00000548322|antisense||n.-1850C>T|||||1850|,T|upstream_gene_variant|MODIFIER|PRMT5|ENSG00000100462|transcript|ENST00000476175|processed_transcript||n.-4974G>A|||||4974|,T|upstream_gene_variant|MODIFIER|PRMT5|ENSG00000100462|transcript|ENST00000555454|protein_coding||c.-3616G>A|||||3616|WARNING_TRANSCRIPT_NO_START_CODON,T|upstream_gene_variant|MODIFIER|PRMT5|ENSG00000100462|transcript|ENST00000454731|protein_coding||c.-4634G>A|||||4633|WARNING_TRANSCRIPT_NO_START_CODON,T|upstream_gene_variant|MODIFIER|PRMT5|ENSG00000100462|transcript|ENST00000557758|retained_intron||n.-4885G>A|||||4885|,T|upstream_gene_variant|MODIFIER|PRMT5|ENSG00000100462|transcript|ENST00000553502|protein_coding||c.-2749G>A|||||2749|WARNING_TRANSCRIPT_NO_START_CODON,T|upstream_gene_variant|MODIFIER|PRMT5|ENSG00000100462|transcript|ENST00000556043|protein_coding||c.-1630G>A|||||1339|WARNING_TRANSCRIPT_NO_STOP_CODON,T|upstream_gene_variant|MODIFIER|PRMT5|ENSG00000100462|transcript|ENST00000553417|retained_intron||n.-199G>A|||||199|,T|upstream_gene_variant|MODIFIER|RP11-298I3.1|ENSG00000257285|transcript|ENST00000548819|antisense||n.-1814C>T|||||1814|,T|downstream_gene_variant|MODIFIER|PRMT5-AS1|ENSG00000237054|transcript|ENST00000424245|antisense||n.*4388C>T|||||4388|,T|downstream_gene_variant|MODIFIER|PRMT5-AS1|ENSG00000237054|transcript|ENST00000609885|antisense||n.*4544C>T|||||4544|,T|downstream_gene_variant|MODIFIER|PRMT5-AS1|ENSG00000237054|transcript|ENST00000457443|antisense||n.*4392C>T|||||4392|,T|downstream_gene_variant|MODIFIER|PRMT5-AS1|ENSG00000237054|transcript|ENST00000599580|antisense||n.*965C>T|||||965|,T|downstream_gene_variant|MODIFIER|PRMT5-AS1|ENSG00000237054|transcript|ENST00000595662|antisense||n.*1176C>T|||||1176|,T|downstream_gene_variant|MODIFIER|PRMT5-AS1|ENSG00000237054|transcript|ENST00000587245|antisense||n.*970C>T|||||970|,T|downstream_gene_variant|MODIFIER|PRMT5-AS1|ENSG00000237054|transcript|ENST00000590290|antisense||n.*899C>T|||||899|,T|downstream_gene_variant|MODIFIER|PRMT5|ENSG00000100462|transcript|ENST00000557015|retained_intron||n.*336G>A|||||336|,T|downstream_gene_variant|MODIFIER|PRMT5|ENSG00000100462|transcript|ENST00000556426|processed_transcript||n.*468G>A|||||468|,T|intron_variant|MODIFIER|PRMT5|ENSG00000100462|transcript|ENST00000324366|protein_coding|3/16|c.316-135G>A||||||,T|intron_variant|MODIFIER|PRMT5|ENSG00000100462|transcript|ENST00000397441|protein_coding|3/16|c.265-135G>A||||||,T|intron_variant|MODIFIER|PRMT5|ENSG00000100462|transcript|ENST00000397440|protein_coding|3/12|c.264+331G>A||||||,T|intron_variant|MODIFIER|PRMT5|ENSG00000100462|transcript|ENST00000216350|protein_coding|2/15|c.179-181G>A||||||,T|intron_variant|MODIFIER|PRMT5|ENSG00000100462|transcript|ENST00000553915|nonsense_mediated_decay|2/15|c.146-135G>A||||||,T|intron_variant|MODIFIER|PRMT5|ENSG00000100462|transcript|ENST00000538452|protein_coding|2/15|c.-3-135G>A||||||,T|intron_variant|MODIFIER|PRMT5|ENSG00000100462|transcript|ENST00000553897|protein_coding|2/15|c.230-181G>A||||||,T|intron_variant|MODIFIER|PRMT5|ENSG00000100462|transcript|ENST00000555530|protein_coding|1/8|c.19-135G>A||||||WARNING_TRANSCRIPT_NO_START_CODON,T|intron_variant|MODIFIER|PRMT5|ENSG00000100462|transcript|ENST00000553550|protein_coding|3/4|c.315+331G>A||||||WARNING_TRANSCRIPT_NO_STOP_CODON,T|intron_variant|MODIFIER|PRMT5|ENSG00000100462|transcript|ENST00000554716|processed_transcript|3/4|n.352+331G>A||||||,T|intron_variant|MODIFIER|PRMT5|ENSG00000100462|transcript|ENST00000553787|nonsense_mediated_decay|1/5|c.111-181G>A||||||,T|intron_variant|MODIFIER|PRMT5|ENSG00000100462|transcript|ENST00000553641|processed_transcript|3/6|n.339-135G>A||||||,T|intron_variant|MODIFIER|PRMT5|ENSG00000100462|transcript|ENST00000554867|protein_coding|3/5|c.315+331G>A||||||WARNING_TRANSCRIPT_NO_STOP_CODON,T|intron_variant|MODIFIER|PRMT5|ENSG00000100462|transcript|ENST00000557415|nonsense_mediated_decay|2/5|c.197-135G>A||||||,T|intron_variant|MODIFIER|PRMT5|ENSG00000100462|transcript|ENST00000556616|protein_coding|2/5|c.202-135G>A||||||WARNING_TRANSCRIPT_INCOMPLETE,T|intron_variant|MODIFIER|PRMT5|ENSG00000100462|transcript|ENST00000554910|protein_coding|3/6|c.190-135G>A||||||WARNING_TRANSCRIPT_INCOMPLETE,T|intron_variant|MODIFIER|PRMT5|ENSG00000100462|transcript|ENST00000421938|protein_coding|3/4|c.346-135G>A||||||WARNING_TRANSCRIPT_NO_STOP_CODON,T|intron_variant|MODIFIER|PRMT5|ENSG00000100462|transcript|ENST00000556032|retained_intron|2/2|n.509-135G>A|||||| |
| AKAP5 | rs6745 | 37 | 14 | 64934977 | 64934977 | + | SNP | C | C | A | SRR8586411 | Translation_Start_Site | 0/1:68,51:119:99:1445,0,1570 | 119;ANN=A|5_prime_UTR_variant|MODIFIER|AKAP5|ENSG00000179841|transcript|ENST00000320636|protein_coding|1/1|c.-136C>A|||||136|WARNING_REF_DOES_NOT_MATCH_GENOME,A|5_prime_UTR_variant|MODIFIER|AKAP5|ENSG00000179841|transcript|ENST00000394718|protein_coding|2/2|c.-136C>A|||||136|WARNING_REF_DOES_NOT_MATCH_GENOME,A|downstream_gene_variant|MODIFIER|CTD-2555O16.3|ENSG00000272828|transcript|ENST00000608003|lincRNA||n.*4802C>A|||||4802|,A|intron_variant|MODIFIER|ZBTB25|ENSG00000089775|transcript|ENST00000555220|protein_coding|2/2|c.174-18621G>T||||||,A|intron_variant|MODIFIER|ZBTB25|ENSG00000089775|transcript|ENST00000555424|protein_coding|2/2|c.257-18621G>T|||||| |
| GOLGA8A | rs554219325 | 37 | 15 | 34794835 | 34794835 | + | SNP | A | A | G | SRR8586411 | Missense_Mutation | 0/1:107,107:214:99:6920,0,4860 | 214;ANN=G|intron_variant|MODIFIER|GOLGA8A|ENSG00000175265|transcript|ENST00000543376|protein_coding|8/22|c.-1598+34710T>C|||||| |
| TTC23 | rs12902459 | 37 | 15 | 99714808 | 99714808 | + | SNP | C | A | A | SRR8586411 | Missense_Mutation | 1/1:2,115:117:99:4431,313,0 | 117;ANN=A|intron_variant|MODIFIER|TTC23|ENSG00000103852|transcript|ENST00000262074|protein_coding|8/11|c.865+447G>T||||||,A|intron_variant|MODIFIER|TTC23|ENSG00000103852|transcript|ENST00000459771|nonsense_mediated_decay|9/12|c.865+447G>T||||||,A|intron_variant|MODIFIER|TTC23|ENSG00000103852|transcript|ENST00000394132|protein_coding|10/13|c.865+447G>T||||||,A|intron_variant|MODIFIER|TTC23|ENSG00000103852|transcript|ENST00000394136|protein_coding|9/12|c.865+447G>T||||||,A|intron_variant|MODIFIER|TTC23|ENSG00000103852|transcript|ENST00000558613|protein_coding|7/10|c.865+447G>T||||||,A|intron_variant|MODIFIER|TTC23|ENSG00000103852|transcript|ENST00000494567|retained_intron|4/7|n.869+447G>T||||||,A|intron_variant|MODIFIER|TTC23|ENSG00000103852|transcript|ENST00000558663|protein_coding|8/11|c.865+447G>T||||||,A|intron_variant|MODIFIER|TTC23|ENSG00000103852|transcript|ENST00000394135|protein_coding|8/11|c.865+447G>T||||||,A|intron_variant|MODIFIER|TTC23|ENSG00000103852|transcript|ENST00000434594|protein_coding|1/5|c.70+447G>T||||||WARNING_TRANSCRIPT_NO_START_CODON,A|intron_variant|MODIFIER|TTC23|ENSG00000103852|transcript|ENST00000394130|protein_coding|9/11|c.865+447G>T||||||,A|intron_variant|MODIFIER|TTC23|ENSG00000103852|transcript|ENST00000394129|protein_coding|10/11|c.865+447G>T|||||| |
| RN7SL367P | rs764861002 | 37 | 16 | 1945932 | 1945932 | + | SNP | T | T | A | SRR8586411 | Missense_Mutation | 0/1:33,250:283:99:7557,0,21252 | 283;ANN=A|upstream_gene_variant|MODIFIER|RN7SL367P|ENSG00000263769|transcript|ENST00000584097|misc_RNA||n.-995A>T|||||995|,A|intergenic_region|MODIFIER|RN7SL367P-HS3ST6|ENSG00000263769-ENSG00000162040|intergenic_region|ENSG00000263769-ENSG00000162040|||n.1945932T>A|||||| |
| RP11-160E2.16 | rs777872060 | 37 | 17 | 19112520 | 19112520 | + | SNP | G | G | A | SRR8586411 | Nonsense_Mutation | 0/1:229,40:269:99:648,0,5179 | 269;ANN=A|downstream_gene_variant|MODIFIER|RP11-160E2.16|ENSG00000236022|transcript|ENST00000447506|lincRNA||n.*2396G>A|||||2396|,A|intron_variant|MODIFIER|RP11-160E2.16|ENSG00000236022|transcript|ENST00000428348|lincRNA|2/2|n.272+2905G>A||||||,A|intron_variant|MODIFIER|RP11-160E2.16|ENSG00000236022|transcript|ENST00000424109|lincRNA|2/2|n.264+2905G>A||||||,A|non_coding_transcript_exon_variant|MODIFIER|KYNUP3|ENSG00000263206|transcript|ENST00000574386|transcribed_unprocessed_pseudogene|2/5|n.301G>A||||||WARNING_REF_DOES_NOT_MATCH_GENOME |
| CYTH1 | rs1065529 | 37 | 17 | 76679918 | 76679918 | + | SNP | C | C | A | SRR8586411 | Missense_Mutation | 0/1:115,594:709:99:15779,0,1775 | 711;ANN=A|upstream_gene_variant|MODIFIER|CYTH1|ENSG00000108669|transcript|ENST00000586430|retained_intron||n.-2770G>T|||||2770|,A|intron_variant|MODIFIER|CYTH1|ENSG00000108669|transcript|ENST00000361101|protein_coding|10/12|c.892-2794G>T||||||,A|intron_variant|MODIFIER|CYTH1|ENSG00000108669|transcript|ENST00000585509|protein_coding|11/13|c.715-2794G>T||||||,A|intron_variant|MODIFIER|CYTH1|ENSG00000108669|transcript|ENST00000589297|protein_coding|11/13|c.715-2794G>T||||||,A|intron_variant|MODIFIER|CYTH1|ENSG00000108669|transcript|ENST00000591455|protein_coding|10/12|c.889-2794G>T||||||,A|intron_variant|MODIFIER|CYTH1|ENSG00000108669|transcript|ENST00000446868|protein_coding|11/13|c.892-2794G>T||||||,A|intron_variant|MODIFIER|CYTH1|ENSG00000108669|transcript|ENST00000589296|protein_coding|3/3|c.171-7874G>T||||||,A|intron_variant|MODIFIER|CYTH1|ENSG00000108669|transcript|ENST00000586175|processed_transcript|1/4|n.78-2794G>T||||||,A|intron_variant|MODIFIER|CYTH1|ENSG00000108669|transcript|ENST00000591574|nonsense_mediated_decay|5/7|c.*365-2794G>T||||||,A|intron_variant|MODIFIER|CYTH1|ENSG00000108669|transcript|ENST00000590775|processed_transcript|3/4|n.503-3618G>T|||||| |
| PLIN4 | rs10423606 | 37 | 19 | 4511896 | 4511896 | + | SNP | C | C | T | SRR8586411 | Nonsense_Mutation | 0/1:37,166:203:99:6595,0,741 | 203;ANN=T|synonymous_variant|LOW|PLIN4|ENSG00000167676|transcript|ENST00000301286|protein_coding|3/6|c.2034G>A|p.Val678Val|2034/6341|2034/4074|678/1357|| |
| PLIN4 | rs28460600 | 37 | 19 | 4511908 | 4511908 | + | SNP | T | T | C | SRR8586411 | Nonsense_Mutation | 0/1:164,16:180:47:47,0,6889 | 180;ANN=C|synonymous_variant|LOW|PLIN4|ENSG00000167676|transcript|ENST00000301286|protein_coding|3/6|c.2022A>G|p.Thr674Thr|2022/6341|2022/4074|674/1357||WARNING_REF_DOES_NOT_MATCH_GENOME |
| ZNF576 | rs745665474 | 37 | 19 | 44106853 | 44106853 | + | SNP | G | G | A | SRR8586411 | Nonsense_Mutation | 0/1:60,10:70:99:233,0,2504 | 70;ANN=A|downstream_gene_variant|MODIFIER|ZNF576|ENSG00000124444|transcript|ENST00000336564|protein_coding||c.*3443G>A|||||1544|,A|downstream_gene_variant|MODIFIER|ZNF428|ENSG00000131116|transcript|ENST00000598676|protein_coding||c.*4927C>T|||||4927|WARNING_TRANSCRIPT_INCOMPLETE,A|downstream_gene_variant|MODIFIER|ZNF576|ENSG00000124444|transcript|ENST00000391965|protein_coding||c.*3443G>A|||||2697|,A|downstream_gene_variant|MODIFIER|ZNF576|ENSG00000124444|transcript|ENST00000525771|protein_coding||c.*3443G>A|||||2331|,A|downstream_gene_variant|MODIFIER|ZNF576|ENSG00000124444|transcript|ENST00000533118|protein_coding||c.*3443G>A|||||3207|,A|downstream_gene_variant|MODIFIER|ZNF576|ENSG00000124444|transcript|ENST00000528387|protein_coding||c.*3443G>A|||||3176|,A|downstream_gene_variant|MODIFIER|ZNF576|ENSG00000124444|transcript|ENST00000529930|protein_coding||c.*3443G>A|||||3287|,A|downstream_gene_variant|MODIFIER|ZNF428|ENSG00000131116|transcript|ENST00000300811|protein_coding||c.*4916C>T|||||4518|,A|intron_variant|MODIFIER|SRRM5|ENSG00000226763|transcript|ENST00000526798|protein_coding|2/2|c.-51+5508G>A||||||,A|intron_variant|MODIFIER|SRRM5|ENSG00000226763|transcript|ENST00000607544|protein_coding|2/2|c.-96+5508G>A|||||| |
| PDE11A | rs3866706 | 37 | 2 | 178655278 | 178655278 | + | SNP | A | A | G | SRR8586411 | Missense_Mutation | 0/1:229,86:315:99:1760,0,6055 | 317;ANN=G|intron_variant|MODIFIER|PDE11A|ENSG00000128655|transcript|ENST00000286063|protein_coding|9/19|c.1738-21177T>C||||||,G|intron_variant|MODIFIER|PDE11A|ENSG00000128655|transcript|ENST00000358450|protein_coding|10/20|c.988-21177T>C||||||,G|intron_variant|MODIFIER|PDE11A|ENSG00000128655|transcript|ENST00000409504|protein_coding|8/19|c.664-21177T>C||||||,G|intron_variant|MODIFIER|PDE11A|ENSG00000128655|transcript|ENST00000389683|protein_coding|6/16|c.406-21177T>C||||||,G|intron_variant|MODIFIER|PDE11A|ENSG00000128655|transcript|ENST00000449286|protein_coding|8/18|c.664-21177T>C||||||,G|intron_variant|MODIFIER|PDE11A|ENSG00000128655|transcript|ENST00000433879|protein_coding|6/13|c.559-21177T>C||||||WARNING_TRANSCRIPT_NO_START_CODON,G|intron_variant|MODIFIER|PDE11A|ENSG00000128655|transcript|ENST00000497003|processed_transcript|8/14|n.780-21177T>C|||||| |
| PDE11A | rs770610620 | 37 | 2 | 178658393 | 178658393 | + | SNP | T | T | C | SRR8586411 | Nonsense_Mutation | 0/1:386,39:425:99:248,0,15230 | 425;ANN=C|downstream_gene_variant|MODIFIER|PDE11A|ENSG00000128655|transcript|ENST00000492761|processed_transcript||n.*3406A>G|||||3406|,C|intron_variant|MODIFIER|PDE11A|ENSG00000128655|transcript|ENST00000286063|protein_coding|9/19|c.1737+23163A>G||||||,C|intron_variant|MODIFIER|PDE11A|ENSG00000128655|transcript|ENST00000358450|protein_coding|10/20|c.987+23163A>G||||||,C|intron_variant|MODIFIER|PDE11A|ENSG00000128655|transcript|ENST00000409504|protein_coding|8/19|c.663+23163A>G||||||,C|intron_variant|MODIFIER|PDE11A|ENSG00000128655|transcript|ENST00000389683|protein_coding|6/16|c.405+23163A>G||||||,C|intron_variant|MODIFIER|PDE11A|ENSG00000128655|transcript|ENST00000449286|protein_coding|8/18|c.663+23163A>G||||||,C|intron_variant|MODIFIER|PDE11A|ENSG00000128655|transcript|ENST00000433879|protein_coding|6/13|c.558+24192A>G||||||WARNING_TRANSCRIPT_NO_START_CODON,C|intron_variant|MODIFIER|PDE11A|ENSG00000128655|transcript|ENST00000497003|processed_transcript|8/14|n.779+23163A>G|||||| |
| PDE11A | rs146313925 | 37 | 2 | 178662395 | 178662395 | + | SNP | T | T | C | SRR8586411 | Missense_Mutation | 0/1:197,74:271:99:1296,0,4915 | 272;ANN=C|intron_variant|MODIFIER|PDE11A|ENSG00000128655|transcript|ENST00000286063|protein_coding|9/19|c.1737+19161A>G||||||,C|intron_variant|MODIFIER|PDE11A|ENSG00000128655|transcript|ENST00000358450|protein_coding|10/20|c.987+19161A>G||||||,C|intron_variant|MODIFIER|PDE11A|ENSG00000128655|transcript|ENST00000409504|protein_coding|8/19|c.663+19161A>G||||||,C|intron_variant|MODIFIER|PDE11A|ENSG00000128655|transcript|ENST00000389683|protein_coding|6/16|c.405+19161A>G||||||,C|intron_variant|MODIFIER|PDE11A|ENSG00000128655|transcript|ENST00000449286|protein_coding|8/18|c.663+19161A>G||||||,C|intron_variant|MODIFIER|PDE11A|ENSG00000128655|transcript|ENST00000433879|protein_coding|6/13|c.558+20190A>G||||||WARNING_TRANSCRIPT_NO_START_CODON,C|intron_variant|MODIFIER|PDE11A|ENSG00000128655|transcript|ENST00000497003|processed_transcript|8/14|n.779+19161A>G||||||,C|intron_variant|MODIFIER|PDE11A|ENSG00000128655|transcript|ENST00000492761|processed_transcript|6/6|n.594-448A>G|||||| |
| PDE11A | rs142156368 | 37 | 2 | 178663341 | 178663341 | + | SNP | C | C | A | SRR8586411 | Missense_Mutation | 0/1:138,251:389:99:6694,0,4218 | 390;ANN=A|intron_variant|MODIFIER|PDE11A|ENSG00000128655|transcript|ENST00000286063|protein_coding|9/19|c.1737+18215G>T||||||,A|intron_variant|MODIFIER|PDE11A|ENSG00000128655|transcript|ENST00000358450|protein_coding|10/20|c.987+18215G>T||||||,A|intron_variant|MODIFIER|PDE11A|ENSG00000128655|transcript|ENST00000409504|protein_coding|8/19|c.663+18215G>T||||||,A|intron_variant|MODIFIER|PDE11A|ENSG00000128655|transcript|ENST00000389683|protein_coding|6/16|c.405+18215G>T||||||,A|intron_variant|MODIFIER|PDE11A|ENSG00000128655|transcript|ENST00000449286|protein_coding|8/18|c.663+18215G>T||||||,A|intron_variant|MODIFIER|PDE11A|ENSG00000128655|transcript|ENST00000433879|protein_coding|6/13|c.558+19244G>T||||||WARNING_TRANSCRIPT_NO_START_CODON,A|intron_variant|MODIFIER|PDE11A|ENSG00000128655|transcript|ENST00000497003|processed_transcript|8/14|n.779+18215G>T||||||,A|intron_variant|MODIFIER|PDE11A|ENSG00000128655|transcript|ENST00000492761|processed_transcript|6/6|n.594-1394G>T|||||| |
| PDE11A | rs12998857 | 37 | 2 | 178668882 | 178668882 | + | SNP | A | A | C | SRR8586411 | Missense_Mutation | 0/1:107,92:199:99:2114,0,2724 | 203;ANN=C|intron_variant|MODIFIER|PDE11A|ENSG00000128655|transcript|ENST00000286063|protein_coding|9/19|c.1737+12674T>G||||||,C|intron_variant|MODIFIER|PDE11A|ENSG00000128655|transcript|ENST00000358450|protein_coding|10/20|c.987+12674T>G||||||,C|intron_variant|MODIFIER|PDE11A|ENSG00000128655|transcript|ENST00000409504|protein_coding|8/19|c.663+12674T>G||||||,C|intron_variant|MODIFIER|PDE11A|ENSG00000128655|transcript|ENST00000389683|protein_coding|6/16|c.405+12674T>G||||||,C|intron_variant|MODIFIER|PDE11A|ENSG00000128655|transcript|ENST00000449286|protein_coding|8/18|c.663+12674T>G||||||,C|intron_variant|MODIFIER|PDE11A|ENSG00000128655|transcript|ENST00000433879|protein_coding|6/13|c.558+13703T>G||||||WARNING_TRANSCRIPT_NO_START_CODON,C|intron_variant|MODIFIER|PDE11A|ENSG00000128655|transcript|ENST00000497003|processed_transcript|8/14|n.779+12674T>G||||||,C|intron_variant|MODIFIER|PDE11A|ENSG00000128655|transcript|ENST00000492761|processed_transcript|6/6|n.594-6935T>G|||||| |
| PDE11A | rs367648529 | 37 | 2 | 178759176 | 178759176 | + | SNP | C | C | T | SRR8586411 | Nonsense_Mutation | 0/1:51,58:109:99:599,0,1235 | 123;ANN=T|downstream_gene_variant|MODIFIER|PDE11A|ENSG00000128655|transcript|ENST00000427127|nonsense_mediated_decay||c.*5118G>A|||||3665|,T|intron_variant|MODIFIER|PDE11A|ENSG00000128655|transcript|ENST00000286063|protein_coding|4/19|c.1302+3609G>A||||||,T|intron_variant|MODIFIER|PDE11A|ENSG00000128655|transcript|ENST00000358450|protein_coding|5/20|c.552+3609G>A||||||,T|intron_variant|MODIFIER|PDE11A|ENSG00000128655|transcript|ENST00000409504|protein_coding|3/19|c.228+3609G>A||||||,T|intron_variant|MODIFIER|PDE11A|ENSG00000128655|transcript|ENST00000449286|protein_coding|3/18|c.228+3609G>A||||||,T|intron_variant|MODIFIER|PDE11A|ENSG00000128655|transcript|ENST00000433879|protein_coding|2/13|c.216+3609G>A||||||WARNING_TRANSCRIPT_NO_START_CODON,T|intron_variant|MODIFIER|PDE11A|ENSG00000128655|transcript|ENST00000497003|processed_transcript|3/14|n.344+3609G>A|||||| |
| SPATS2L | rs376105259 | 37 | 2 | 201166957 | 201166957 | + | SNP | G | G | A | SRR8586411 | Missense_Mutation | 0/1:37,29:66:99:1240,0,2217 | 66;ANN=A|upstream_gene_variant|MODIFIER|SPATS2L|ENSG00000196141|transcript|ENST00000439084|protein_coding||c.-87011G>A|||||3647|WARNING_TRANSCRIPT_NO_STOP_CODON,A|upstream_gene_variant|MODIFIER|SPATS2L|ENSG00000196141|transcript|ENST00000409718|protein_coding||c.-87011G>A|||||3659|,A|upstream_gene_variant|MODIFIER|SPATS2L|ENSG00000196141|transcript|ENST00000358677|protein_coding||c.-87011G>A|||||3752|,A|upstream_gene_variant|MODIFIER|SPATS2L|ENSG00000196141|transcript|ENST00000409988|protein_coding||c.-87011G>A|||||3917|,A|upstream_gene_variant|MODIFIER|SPATS2L|ENSG00000196141|transcript|ENST00000409385|protein_coding||c.-114177G>A|||||3925|,A|upstream_gene_variant|MODIFIER|SPATS2L|ENSG00000196141|transcript|ENST00000439395|protein_coding||c.-87011G>A|||||4042|WARNING_TRANSCRIPT_INCOMPLETE,A|upstream_gene_variant|MODIFIER|SPATS2L|ENSG00000196141|transcript|ENST00000444012|protein_coding||c.-87011G>A|||||4049|WARNING_TRANSCRIPT_INCOMPLETE,A|upstream_gene_variant|MODIFIER|SPATS2L|ENSG00000196141|transcript|ENST00000451764|protein_coding||c.-87011G>A|||||4142|,A|upstream_gene_variant|MODIFIER|SPATS2L|ENSG00000196141|transcript|ENST00000360760|protein_coding||c.-87011G>A|||||4290|,A|upstream_gene_variant|MODIFIER|SPATS2L|ENSG00000196141|transcript|ENST00000423749|protein_coding||c.-87011G>A|||||4319|WARNING_TRANSCRIPT_INCOMPLETE,A|upstream_gene_variant|MODIFIER|SPATS2L|ENSG00000196141|transcript|ENST00000428692|protein_coding||c.-87011G>A|||||4320|WARNING_TRANSCRIPT_NO_STOP_CODON,A|upstream_gene_variant|MODIFIER|SPATS2L|ENSG00000196141|transcript|ENST00000471601|retained_intron||n.-4320G>A|||||4320|,A|upstream_gene_variant|MODIFIER|SPATS2L|ENSG00000196141|transcript|ENST00000457757|protein_coding||c.-87011G>A|||||4320|WARNING_TRANSCRIPT_NO_STOP_CODON,A|upstream_gene_variant|MODIFIER|SPATS2L|ENSG00000196141|transcript|ENST00000453663|protein_coding||c.-87011G>A|||||4334|WARNING_TRANSCRIPT_INCOMPLETE,A|upstream_gene_variant|MODIFIER|SPATS2L|ENSG00000196141|transcript|ENST00000409140|protein_coding||c.-87011G>A|||||4474|,A|upstream_gene_variant|MODIFIER|SPATS2L|ENSG00000196141|transcript|ENST00000459656|retained_intron||n.-4484G>A|||||4484|,A|upstream_gene_variant|MODIFIER|SPATS2L|ENSG00000196141|transcript|ENST00000471533|processed_transcript||n.-4493G>A|||||4493|,A|upstream_gene_variant|MODIFIER|SPATS2L|ENSG00000196141|transcript|ENST00000409397|protein_coding||c.-87011G>A|||||4638|WARNING_TRANSCRIPT_INCOMPLETE,A|intergenic_region|MODIFIER|C2orf47-SPATS2L|ENSG00000162972-ENSG00000196141|intergenic_region|ENSG00000162972-ENSG00000196141|||n.201166957G>A|||||| |
| SPATS2L | rs370375013 | 37 | 2 | 201166960 | 201166960 | + | SNP | G | G | A | SRR8586411 | Missense_Mutation | 0/1:41,28:69:99:1094,0,2151 | 69;ANN=A|upstream_gene_variant|MODIFIER|SPATS2L|ENSG00000196141|transcript|ENST00000439084|protein_coding||c.-87008G>A|||||3644|WARNING_TRANSCRIPT_NO_STOP_CODON,A|upstream_gene_variant|MODIFIER|SPATS2L|ENSG00000196141|transcript|ENST00000409718|protein_coding||c.-87008G>A|||||3656|,A|upstream_gene_variant|MODIFIER|SPATS2L|ENSG00000196141|transcript|ENST00000358677|protein_coding||c.-87008G>A|||||3749|,A|upstream_gene_variant|MODIFIER|SPATS2L|ENSG00000196141|transcript|ENST00000409988|protein_coding||c.-87008G>A|||||3914|,A|upstream_gene_variant|MODIFIER|SPATS2L|ENSG00000196141|transcript|ENST00000409385|protein_coding||c.-114174G>A|||||3922|,A|upstream_gene_variant|MODIFIER|SPATS2L|ENSG00000196141|transcript|ENST00000439395|protein_coding||c.-87008G>A|||||4039|WARNING_TRANSCRIPT_INCOMPLETE,A|upstream_gene_variant|MODIFIER|SPATS2L|ENSG00000196141|transcript|ENST00000444012|protein_coding||c.-87008G>A|||||4046|WARNING_TRANSCRIPT_INCOMPLETE,A|upstream_gene_variant|MODIFIER|SPATS2L|ENSG00000196141|transcript|ENST00000451764|protein_coding||c.-87008G>A|||||4139|,A|upstream_gene_variant|MODIFIER|SPATS2L|ENSG00000196141|transcript|ENST00000360760|protein_coding||c.-87008G>A|||||4287|,A|upstream_gene_variant|MODIFIER|SPATS2L|ENSG00000196141|transcript|ENST00000423749|protein_coding||c.-87008G>A|||||4316|WARNING_TRANSCRIPT_INCOMPLETE,A|upstream_gene_variant|MODIFIER|SPATS2L|ENSG00000196141|transcript|ENST00000428692|protein_coding||c.-87008G>A|||||4317|WARNING_TRANSCRIPT_NO_STOP_CODON,A|upstream_gene_variant|MODIFIER|SPATS2L|ENSG00000196141|transcript|ENST00000471601|retained_intron||n.-4317G>A|||||4317|,A|upstream_gene_variant|MODIFIER|SPATS2L|ENSG00000196141|transcript|ENST00000457757|protein_coding||c.-87008G>A|||||4317|WARNING_TRANSCRIPT_NO_STOP_CODON,A|upstream_gene_variant|MODIFIER|SPATS2L|ENSG00000196141|transcript|ENST00000453663|protein_coding||c.-87008G>A|||||4331|WARNING_TRANSCRIPT_INCOMPLETE,A|upstream_gene_variant|MODIFIER|SPATS2L|ENSG00000196141|transcript|ENST00000409140|protein_coding||c.-87008G>A|||||4471|,A|upstream_gene_variant|MODIFIER|SPATS2L|ENSG00000196141|transcript|ENST00000459656|retained_intron||n.-4481G>A|||||4481|,A|upstream_gene_variant|MODIFIER|SPATS2L|ENSG00000196141|transcript|ENST00000471533|processed_transcript||n.-4490G>A|||||4490|,A|upstream_gene_variant|MODIFIER|SPATS2L|ENSG00000196141|transcript|ENST00000409397|protein_coding||c.-87008G>A|||||4635|WARNING_TRANSCRIPT_INCOMPLETE,A|intergenic_region|MODIFIER|C2orf47-SPATS2L|ENSG00000162972-ENSG00000196141|intergenic_region|ENSG00000162972-ENSG00000196141|||n.201166960G>A|||||| |
| DLGAP4 | rs751905425 | 37 | 20 | 34996771 | 34996771 | + | SNP | G | G | A | SRR8586411 | Missense_Mutation | 0/1:21,23:44:99:903,0,7529 | 44;ANN=A|intron_variant|MODIFIER|DLGAP4|ENSG00000080845|transcript|ENST00000373913|protein_coding|2/12|c.-73+1093G>A||||||,A|intron_variant|MODIFIER|DLGAP4|ENSG00000080845|transcript|ENST00000401952|protein_coding|1/11|c.-73+1093G>A||||||,A|intron_variant|MODIFIER|DLGAP4|ENSG00000080845|transcript|ENST00000373907|protein_coding|1/11|c.-73+1093G>A|||||| |
| LRP5L | rs13058434 | 37 | 22 | 25768446 | 25768446 | + | SNP | C | C | T | SRR8586411 | Missense_Mutation | 0/1:159,215:374:99:5023,0,3890 | 381;ANN=T|intron_variant|MODIFIER|LRP5L|ENSG00000100068|transcript|ENST00000402859|protein_coding|2/5|c.-132+3334G>A||||||,T|intron_variant|MODIFIER|LRP5L|ENSG00000100068|transcript|ENST00000444995|protein_coding|3/6|c.-132+3334G>A||||||,T|intron_variant|MODIFIER|LRP5L|ENSG00000100068|transcript|ENST00000468442|protein_coding|2/3|c.-243+3334G>A||||||WARNING_TRANSCRIPT_INCOMPLETE |
| NFKBIZ | rs771150914 | 37 | 3 | 101576663 | 101576663 | + | SNP | A | A | G | SRR8586411 | Nonsense_Mutation | 0/1:18,85:103:99:2477,0,415 | 104;ANN=G|downstream_gene_variant|MODIFIER|NFKBIZ|ENSG00000144802|transcript|ENST00000483180|protein_coding||c.*360A>G|||||360|WARNING_TRANSCRIPT_NO_STOP_CODON,G|downstream_gene_variant|MODIFIER|NFKBIZ|ENSG00000144802|transcript|ENST00000491281|protein_coding||c.*4604A>G|||||4604|WARNING_TRANSCRIPT_INCOMPLETE,G|downstream_gene_variant|MODIFIER|NFKBIZ|ENSG00000144802|transcript|ENST00000465476|retained_intron||n.*1802A>G|||||1802|,G|intron_variant|MODIFIER|NFKBIZ|ENSG00000144802|transcript|ENST00000326172|protein_coding|11/11|c.2103+360A>G||||||,G|intron_variant|MODIFIER|NFKBIZ|ENSG00000144802|transcript|ENST00000394054|protein_coding|12/12|c.1803+360A>G||||||,G|intron_variant|MODIFIER|NFKBIZ|ENSG00000144802|transcript|ENST00000326151|protein_coding|12/12|c.1737+360A>G||||||,G|intron_variant|MODIFIER|NFKBIZ|ENSG00000144802|transcript|ENST00000477601|protein_coding|2/2|c.336+360A>G||||||,G|intron_variant|MODIFIER|NFKBIZ|ENSG00000144802|transcript|ENST00000495089|retained_intron|2/2|n.671+360A>G||||||,G|intron_variant|MODIFIER|NFKBIZ|ENSG00000144802|transcript|ENST00000495719|retained_intron|1/1|n.260+360A>G|||||| |
| FAM188B2 | rs14434 | 37 | 3 | 150584223 | 150584223 | + | SNP | C | C | T | SRR8586411 | Nonsense_Mutation | 0/1:17,23:40:99:603,0,434 | 40;ANN=T|downstream_gene_variant|MODIFIER|FAM188B2|ENSG00000214237|transcript|ENST00000397891|protein_coding||c.*4609G>A|||||4609|,T|downstream_gene_variant|MODIFIER|FAM188B2|ENSG00000214237|transcript|ENST00000465419|protein_coding||c.*4609G>A|||||4609|WARNING_TRANSCRIPT_NO_START_CODON,T|intron_variant|MODIFIER|CLRN1-AS1|ENSG00000239265|transcript|ENST00000476886|antisense|1/4|n.123+13830C>T|||||| |
| RBPJ | rs201279979 | 37 | 4 | 26431200 | 26431200 | + | SNP | A | A | G | SRR8586411 | Missense_Mutation | 0/1:33,25:58:99:506,0,707 | 59;ANN=G|upstream_gene_variant|MODIFIER|RBPJ|ENSG00000168214|transcript|ENST00000505727|retained_intron||n.-568A>G|||||568|,G|downstream_gene_variant|MODIFIER|RBPJ|ENSG00000168214|transcript|ENST00000506903|retained_intron||n.*4584A>G|||||4584|,G|intron_variant|MODIFIER|RBPJ|ENSG00000168214|transcript|ENST00000342295|protein_coding|9/11|c.928-320A>G||||||,G|intron_variant|MODIFIER|RBPJ|ENSG00000168214|transcript|ENST00000345843|protein_coding|8/10|c.883-320A>G||||||,G|intron_variant|MODIFIER|RBPJ|ENSG00000168214|transcript|ENST00000361572|protein_coding|8/10|c.928-320A>G||||||,G|intron_variant|MODIFIER|RBPJ|ENSG00000168214|transcript|ENST00000514380|retained_intron|8/8|n.960-320A>G||||||,G|intron_variant|MODIFIER|RBPJ|ENSG00000168214|transcript|ENST00000348160|protein_coding|8/10|c.889-320A>G||||||,G|intron_variant|MODIFIER|RBPJ|ENSG00000168214|transcript|ENST00000355476|protein_coding|9/11|c.886-320A>G||||||,G|intron_variant|MODIFIER|RBPJ|ENSG00000168214|transcript|ENST00000507561|protein_coding|8/10|c.823-320A>G||||||,G|intron_variant|MODIFIER|RBPJ|ENSG00000168214|transcript|ENST00000504907|protein_coding|8/9|c.886-320A>G||||||,G|intron_variant|MODIFIER|RBPJ|ENSG00000168214|transcript|ENST00000342320|protein_coding|8/10|c.886-320A>G||||||,G|intron_variant|MODIFIER|RBPJ|ENSG00000168214|transcript|ENST00000510725|retained_intron|1/1|n.249-320A>G||||||,G|intron_variant|MODIFIER|RBPJ|ENSG00000168214|transcript|ENST00000504423|protein_coding|1/2|c.141+718A>G||||||WARNING_TRANSCRIPT_NO_START_CODON |
| CENPU | rs768412056 | 37 | 4 | 185652732 | 185652732 | + | SNP | T | T | C | SRR8586411 | Missense_Mutation | 0/1:55,72:127:99:2852,0,2618 | 127;ANN=C|intron_variant|MODIFIER|CENPU|ENSG00000151725|transcript|ENST00000281453|protein_coding|1/12|c.48-610A>G||||||,C|intron_variant|MODIFIER|CENPU|ENSG00000151725|transcript|ENST00000510146|nonsense_mediated_decay|1/11|c.48-610A>G||||||,C|intron_variant|MODIFIER|CENPU|ENSG00000151725|transcript|ENST00000541971|protein_coding|1/11|c.48-610A>G||||||,C|intron_variant|MODIFIER|CENPU|ENSG00000151725|transcript|ENST00000514781|protein_coding|1/5|c.-40-610A>G||||||WARNING_TRANSCRIPT_NO_STOP_CODON |
| SLC1A3 | rs145798494 | 37 | 5 | 36649326 | 36649326 | + | DEL | CAACAAAAGCAAAACTCCATCTCAAAAAAAAAAAAA | CAACAAAAGCAAAACTCCATCTCAAAAAAAAAAAAA | C | SRR8586411 | In_Frame_Del | 0/1:23,35:58:99:1383,0,913 | 58;ANN=C|intron_variant|MODIFIER|SLC1A3|ENSG00000079215|transcript|ENST00000265113|protein_coding|3/9|c.319+19638_319+19672delAACAAAAGCAAAACTCCATCTCAAAAAAAAAAAAA||||||,C|intron_variant|MODIFIER|SLC1A3|ENSG00000079215|transcript|ENST00000381918|protein_coding|2/7|c.319+19638_319+19672delAACAAAAGCAAAACTCCATCTCAAAAAAAAAAAAA||||||,C|intron_variant|MODIFIER|SLC1A3|ENSG00000079215|transcript|ENST00000514563|processed_transcript|2/2|n.384+19638_384+19672delAACAAAAGCAAAACTCCATCTCAAAAAAAAAAAAA||||||,C|intron_variant|MODIFIER|SLC1A3|ENSG00000079215|transcript|ENST00000509272|processed_transcript|2/2|n.339+19638_339+19672delAACAAAAGCAAAACTCCATCTCAAAAAAAAAAAAA|||||| |
| FBXL17-RP11-120B7.1 | rs1368441 | 37 | 5 | 107859556 | 107859556 | + | SNP | C | C | A | SRR8586411 | Missense_Mutation | 0/1:23,8:31:99:256,0,1315 | 31;ANN=A|intergenic_region|MODIFIER|FBXL17-RP11-120B7.1|ENSG00000145743-ENSG00000244245|intergenic_region|ENSG00000145743-ENSG00000244245|||n.107859556C>A|||||| |
| FBXL17-RP11-120B7.1 | rs1054248 | 37 | 5 | 107859561 | 107859561 | + | SNP | C | C | A | SRR8586411 | Missense_Mutation | 0/1:22,8:30:99:259,0,1285 | 30;ANN=A|intergenic_region|MODIFIER|FBXL17-RP11-120B7.1|ENSG00000145743-ENSG00000244245|intergenic_region|ENSG00000145743-ENSG00000244245|||n.107859561C>A|||||| |
| XXbac-BPG248L24.13 | rs2308592 | 37 | 6 | 31271153 | 31271153 | + | SNP | A | A | C | SRR8586411 | Missense_Mutation | 0/1:90,51:141:99:1115,0,2783 | 141;ANN=C|upstream_gene_variant|MODIFIER|XXbac-BPG248L24.13|ENSG00000256166|transcript|ENST00000539514|lincRNA||n.-1734T>G|||||1734|,C|downstream_gene_variant|MODIFIER|XXbac-BPG248L24.10|ENSG00000229836|transcript|ENST00000421191|unprocessed_pseudogene||n.*4439T>G|||||4439|,C|intergenic_region|MODIFIER|XXbac-BPG248L24.13-XXbac-BPG248L24.10|ENSG00000256166-ENSG00000229836|intergenic_region|ENSG00000256166-ENSG00000229836|||n.31271153A>C|||||| |
| XXbac-BPG248L24.13 | rs2308590 | 37 | 6 | 31271165 | 31271165 | + | SNP | G | G | T | SRR8586411 | Missense_Mutation | 0/1:99,51:150:99:1126,0,2402 | 150;ANN=T|upstream_gene_variant|MODIFIER|XXbac-BPG248L24.13|ENSG00000256166|transcript|ENST00000539514|lincRNA||n.-1746C>A|||||1746|,T|downstream_gene_variant|MODIFIER|XXbac-BPG248L24.10|ENSG00000229836|transcript|ENST00000421191|unprocessed_pseudogene||n.*4427C>A|||||4427|,T|intergenic_region|MODIFIER|XXbac-BPG248L24.13-XXbac-BPG248L24.10|ENSG00000256166-ENSG00000229836|intergenic_region|ENSG00000256166-ENSG00000229836|||n.31271165G>T|||||| |
| RCAN2 | rs11760109 | 37 | 6 | 46446313 | 46446313 | + | SNP | T | T | G | SRR8586411 | Missense_Mutation | 0/1:28,23:51:99:608,0,772 | 51;ANN=G|intron_variant|MODIFIER|RCAN2|ENSG00000172348|transcript|ENST00000306764|protein_coding|1/4|c.-3+13294A>C||||||,G|intron_variant|MODIFIER|RCAN2|ENSG00000172348|transcript|ENST00000371374|protein_coding|1/4|c.-3+12597A>C|||||| |
| EXOC4 | rs12671131 | 37 | 7 | 133034625 | 133034625 | + | SNP | G | G | C | SRR8586411 | Missense_Mutation | 0/1:5,16:21:98:431,0,98 | 25;ANN=C|intron_variant|MODIFIER|EXOC4|ENSG00000131558|transcript|ENST00000253861|protein_coding|5/17|c.764-6459G>C||||||,C|intron_variant|MODIFIER|EXOC4|ENSG00000131558|transcript|ENST00000393161|protein_coding|5/9|c.764-6459G>C||||||,C|intron_variant|MODIFIER|EXOC4|ENSG00000131558|transcript|ENST00000486013|retained_intron|5/9|n.793-6459G>C||||||,C|intron_variant|MODIFIER|EXOC4|ENSG00000131558|transcript|ENST00000462055|retained_intron|5/8|n.771-6459G>C||||||,C|intron_variant|MODIFIER|EXOC4|ENSG00000131558|transcript|ENST00000539845|protein_coding|5/17|c.461-6459G>C|||||| |
| MYOM2 | rs3779840 | 37 | 8 | 2100690 | 2100690 | + | SNP | G | G | A | SRR8586411 | Missense_Mutation | 0/1:13,31:44:99:887,0,234 | 44;ANN=A|intron_variant|MODIFIER|MYOM2|ENSG00000036448|transcript|ENST00000520779|processed_transcript|4/4|n.154+9310G>A|||||| |
| MYOM2 | rs968381 | 37 | 8 | 2101041 | 2101041 | + | SNP | G | G | A | SRR8586411 | Missense_Mutation | 0/1:169,36:205:99:647,0,4189 | 222;ANN=A|intron_variant|MODIFIER|MYOM2|ENSG00000036448|transcript|ENST00000520779|processed_transcript|4/4|n.154+9661G>A|||||| |
| SNTG1 | rs118108043 | 37 | 8 | 51696568 | 51696568 | + | SNP | C | C | T | SRR8586411 | Missense_Mutation | 0/1:21,17:38:99:467,0,511 | 38;ANN=T|intron_variant|MODIFIER|SNTG1|ENSG00000147481|transcript|ENST00000518864|protein_coding|19/19|c.1396-8663C>T||||||,T|intron_variant|MODIFIER|SNTG1|ENSG00000147481|transcript|ENST00000522124|protein_coding|18/18|c.1396-8663C>T||||||,T|intron_variant|MODIFIER|SNTG1|ENSG00000147481|transcript|ENST00000517473|protein_coding|16/16|c.1285-8663C>T||||||,T|intron_variant|MODIFIER|SNTG1|ENSG00000147481|transcript|ENST00000520825|nonsense_mediated_decay|17/17|c.*375-8663C>T||||||,T|intron_variant|MODIFIER|SNTG1|ENSG00000147481|transcript|ENST00000276467|protein_coding|15/15|c.1285-8663C>T|||||| |
| NFIB | rs7029180 | 37 | 9 | 14220408 | 14220408 | + | SNP | T | T | C | SRR8586411 | Missense_Mutation | 0/1:12,16:28:99:377,0,359 | 28;ANN=C|intron_variant|MODIFIER|NFIB|ENSG00000147862|transcript|ENST00000397581|protein_coding|2/11|c.563-40629A>G||||||,C|intron_variant|MODIFIER|NFIB|ENSG00000147862|transcript|ENST00000380934|protein_coding|2/8|c.641-40629A>G||||||,C|intron_variant|MODIFIER|NFIB|ENSG00000147862|transcript|ENST00000380959|protein_coding|2/8|c.563-40629A>G||||||,C|intron_variant|MODIFIER|NFIB|ENSG00000147862|transcript|ENST00000380953|protein_coding|2/10|c.563-40629A>G||||||,C|intron_variant|MODIFIER|NFIB|ENSG00000147862|transcript|ENST00000397575|protein_coding|2/11|c.563-40629A>G||||||,C|intron_variant|MODIFIER|NFIB|ENSG00000147862|transcript|ENST00000397579|protein_coding|2/9|c.563-40629A>G|||||| |
| RNA5SP284 | rs443057 | 37 | 9 | 68405372 | 68405372 | + | SNP | C | C | G | SRR8586411 | Missense_Mutation | 0/1:10,25:35:99:652,0,243 | 35;ANN=G|upstream_gene_variant|MODIFIER|RNA5SP284|ENSG00000207277|transcript|ENST00000384547|rRNA||n.-3552C>G|||||3552|,G|intron_variant|MODIFIER|RP11-764K9.1|ENSG00000225411|transcript|ENST00000417843|lincRNA|2/4|n.224+1853G>C|||||| |
| TPT1P9-RP11-349E4.1 | rs528604896 | 37 | 9 | 121326672 | 121326672 | + | SNP | G | G | T | SRR8586411 | Missense_Mutation | 0/1:100,68:168:99:1346,0,2196 | 170;ANN=T|intergenic_region|MODIFIER|TPT1P9-RP11-349E4.1|ENSG00000234782-ENSG00000225050|intergenic_region|ENSG00000234782-ENSG00000225050|||n.121326672G>T|||||| |
| ERI3 | rs773928872 | 37 | 1 | 44777838 | 44777838 | + | SNP | A | A | T | SRR8586412 | Missense_Mutation | 0/1:7,57:64:25:2544,0,25 | 64;ANN=T|intron_variant|MODIFIER|ERI3|ENSG00000117419|transcript|ENST00000372257|protein_coding|5/8|c.666+1003T>A||||||,T|intron_variant|MODIFIER|ERI3|ENSG00000117419|transcript|ENST00000456170|protein_coding|2/6|c.183+1003T>A||||||WARNING_TRANSCRIPT_NO_START_CODON,T|intron_variant|MODIFIER|ERI3|ENSG00000117419|transcript|ENST00000372259|protein_coding|3/6|c.321+1003T>A||||||,T|intron_variant|MODIFIER|ERI3|ENSG00000117419|transcript|ENST00000537474|protein_coding|3/6|c.135+1003T>A||||||,T|intron_variant|MODIFIER|ERI3|ENSG00000117419|transcript|ENST00000452396|protein_coding|3/5|c.312+1003T>A||||||WARNING_TRANSCRIPT_INCOMPLETE,T|intron_variant|MODIFIER|ERI3|ENSG00000117419|transcript|ENST00000495828|processed_transcript|4/5|n.802+1003T>A||||||,T|intron_variant|MODIFIER|ERI3|ENSG00000117419|transcript|ENST00000457571|protein_coding|5/5|c.660+1003T>A||||||WARNING_TRANSCRIPT_INCOMPLETE |
| AK5 | rs754819624 | 37 | 1 | 77942201 | 77942201 | + | SNP | T | T | C | SRR8586412 | Missense_Mutation | 0/1:105,246:351:99:9762,0,2722 | 355;ANN=C|intron_variant|MODIFIER|AK5|ENSG00000154027|transcript|ENST00000354567|protein_coding|8/13|c.1060-6801T>C||||||,C|intron_variant|MODIFIER|AK5|ENSG00000154027|transcript|ENST00000344720|protein_coding|8/13|c.982-6801T>C||||||,C|intron_variant|MODIFIER|AK5|ENSG00000154027|transcript|ENST00000530826|processed_transcript|3/7|n.259-6801T>C||||||,C|intron_variant|MODIFIER|AK5|ENSG00000154027|transcript|ENST00000527263|nonsense_mediated_decay|1/5|c.22-6801T>C||||||WARNING_TRANSCRIPT_NO_START_CODON |
| NOTCH2 | rs3851873 | 37 | 1 | 120492014 | 120492014 | + | SNP | C | C | T | SRR8586412 | Nonsense_Mutation | 0/1:230,32:262:99:337,0,9613 | 263;ANN=T|downstream_gene_variant|MODIFIER|NOTCH2|ENSG00000134250|transcript|ENST00000479412|retained_intron||n.*729G>A|||||729|,T|intron_variant|MODIFIER|NOTCH2|ENSG00000134250|transcript|ENST00000256646|protein_coding|15/33|c.2480-265G>A|||||| |
| NOTCH2 | rs3863691 | 37 | 1 | 120492015 | 120492015 | + | SNP | T | T | C | SRR8586412 | Nonsense_Mutation | 0/1:229,35:264:99:710,0,8100 | 264;ANN=C|downstream_gene_variant|MODIFIER|NOTCH2|ENSG00000134250|transcript|ENST00000479412|retained_intron||n.*728A>G|||||728|,C|intron_variant|MODIFIER|NOTCH2|ENSG00000134250|transcript|ENST00000256646|protein_coding|15/33|c.2480-266A>G|||||| |
| RP11-533N14.3-GJA5 | rs587701389 | 37 | 1 | 147179457 | 147179457 | + | INS | A | A | AG | SRR8586412 | Frame_Shift_Ins | 0/1:21,8:29:99:208,0,687 | 31;ANN=AG|intergenic_region|MODIFIER|RP11-533N14.3-GJA5|ENSG00000227139-ENSG00000143140|intergenic_region|ENSG00000227139-ENSG00000143140|||n.147179457_147179458insG|||||| |
| RP11-763B22.9 | rs1664022 | 37 | 1 | 148889827 | 148889827 | + | SNP | G | G | T | SRR8586412 | Missense_Mutation | 0/1:252,35:287:99:444,0,6983 | 287;ANN=T|intron_variant|MODIFIER|RP11-763B22.9|ENSG00000231448|transcript|ENST00000444424|unprocessed_pseudogene|8/9|n.1017+138G>T|||||| |
| RP11-14N7.2 | rs619027 | 37 | 1 | 148932609 | 148932609 | + | SNP | T | A | A | SRR8586412 | Missense_Mutation | 1/1:20,195:215:18:6252,18,0 | 215;ANN=A|upstream_gene_variant|MODIFIER|RP11-14N7.2|ENSG00000232527|transcript|ENST00000457390|lincRNA||n.-176T>A|||||176|,A|intron_variant|MODIFIER|RP11-14N7.2|ENSG00000232527|transcript|ENST00000539543|lincRNA|1/3|n.42-177T>A||||||,A|intron_variant|MODIFIER|RP11-14N7.2|ENSG00000232527|transcript|ENST00000420597|lincRNA|1/1|n.65-177T>A||||||,A|intron_variant|MODIFIER|RP11-14N7.2|ENSG00000232527|transcript|ENST00000452399|lincRNA|1/2|n.65-177T>A||||||,A|intron_variant|MODIFIER|RP11-14N7.2|ENSG00000232527|transcript|ENST00000294715|lincRNA|1/2|n.56-177T>A|||||| |
| RP11-14N7.2 | rs3124680 | 37 | 1 | 148933084 | 148933084 | + | SNP | T | T | A | SRR8586412 | Nonsense_Mutation | 0/1:45,45:90:99:1277,0,1132 | 90;ANN=A|downstream_gene_variant|MODIFIER|RP11-14N7.2|ENSG00000232527|transcript|ENST00000420597|lincRNA||n.*164T>A|||||164|,A|intron_variant|MODIFIER|RP11-14N7.2|ENSG00000232527|transcript|ENST00000539543|lincRNA|2/3|n.176+164T>A||||||,A|intron_variant|MODIFIER|RP11-14N7.2|ENSG00000232527|transcript|ENST00000452399|lincRNA|2/2|n.199+164T>A||||||,A|intron_variant|MODIFIER|RP11-14N7.2|ENSG00000232527|transcript|ENST00000294715|lincRNA|2/2|n.190+164T>A||||||,A|intron_variant|MODIFIER|RP11-14N7.2|ENSG00000232527|transcript|ENST00000457390|lincRNA|1/1|n.136+164T>A|||||| |
| RNVU1-17 | rs75500033 | 37 | 1 | 149197768 | 149197768 | + | SNP | C | C | T | SRR8586412 | Missense_Mutation | 0/1:931,124:1055:99:1274,0,35675 | 1055;ANN=T|upstream_gene_variant|MODIFIER|RNVU1-17|ENSG00000207349|transcript|ENST00000384619|snRNA||n.-3499G>A|||||3499|,T|intergenic_region|MODIFIER|RNVU1-17-RNU1-92P|ENSG00000207349-ENSG00000252826|intergenic_region|ENSG00000207349-ENSG00000252826|||n.149197768C>T|||||| |
| RNVU1-17 | rs201921277 | 37 | 1 | 149197982 | 149197982 | + | SNP | C | C | G | SRR8586412 | Missense_Mutation | 0/1:216,67:283:99:1100,0,6490 | 285;ANN=G|upstream_gene_variant|MODIFIER|RNVU1-17|ENSG00000207349|transcript|ENST00000384619|snRNA||n.-3713G>C|||||3713|,G|intergenic_region|MODIFIER|RNVU1-17-RNU1-92P|ENSG00000207349-ENSG00000252826|intergenic_region|ENSG00000207349-ENSG00000252826|||n.149197982C>G|||||| |
| RIIAD1 | rs4617400 | 37 | 1 | 151696498 | 151696498 | + | SNP | T | T | G | SRR8586412 | Missense_Mutation | 0/1:20,23:43:99:474,0,743 | 49;ANN=G|upstream_gene_variant|MODIFIER|RIIAD1|ENSG00000178796|transcript|ENST00000451222|processed_transcript||n.-4349T>G|||||4349|,G|upstream_gene_variant|MODIFIER|RIIAD1|ENSG00000178796|transcript|ENST00000427205|processed_transcript||n.-4407T>G|||||4407|,G|intron_variant|MODIFIER|AL589765.1|ENSG00000240510|transcript|ENST00000442233|protein_coding|6/8|c.*1004+1860T>G||||||,G|intron_variant|MODIFIER|RIIAD1|ENSG00000178796|transcript|ENST00000326413|protein_coding|6/8|c.191+1860T>G||||||,G|intron_variant|MODIFIER|RIIAD1|ENSG00000178796|transcript|ENST00000479191|protein_coding|2/4|c.161+1860T>G||||||,G|intron_variant|MODIFIER|RIIAD1|ENSG00000178796|transcript|ENST00000451484|processed_transcript|2/4|n.178+1860T>G||||||,G|intron_variant|MODIFIER|RIIAD1|ENSG00000178796|transcript|ENST00000426175|processed_transcript|1/2|n.34+1860T>G|||||| |
| RP11-385F5.5 | rs56333540 | 37 | 1 | 236695573 | 236695573 | + | SNP | T | T | C | SRR8586412 | Nonsense_Mutation | 0/1:72,28:100:99:782,0,2043 | 100;ANN=C|downstream_gene_variant|MODIFIER|RP11-385F5.5|ENSG00000273058|transcript|ENST00000608547|antisense||n.*3900A>G|||||3900|,C|intron_variant|MODIFIER|LGALS8|ENSG00000116977|transcript|ENST00000352231|protein_coding|3/11|c.46-5224T>C||||||,C|intron_variant|MODIFIER|LGALS8|ENSG00000116977|transcript|ENST00000481485|protein_coding|3/4|c.46-5224T>C||||||WARNING_TRANSCRIPT_INCOMPLETE,C|intron_variant|MODIFIER|LGALS8|ENSG00000116977|transcript|ENST00000454943|protein_coding|4/7|c.46-5224T>C||||||WARNING_TRANSCRIPT_INCOMPLETE,C|intron_variant|MODIFIER|LGALS8|ENSG00000116977|transcript|ENST00000527974|protein_coding|2/10|c.46-5224T>C||||||,C|intron_variant|MODIFIER|LGALS8|ENSG00000116977|transcript|ENST00000430527|protein_coding|3/5|c.46-5224T>C||||||WARNING_TRANSCRIPT_INCOMPLETE,C|intron_variant|MODIFIER|LGALS8|ENSG00000116977|transcript|ENST00000406509|protein_coding|5/10|c.46-5224T>C||||||WARNING_TRANSCRIPT_INCOMPLETE,C|intron_variant|MODIFIER|LGALS8|ENSG00000116977|transcript|ENST00000526589|protein_coding|5/13|c.46-5224T>C||||||,C|intron_variant|MODIFIER|LGALS8|ENSG00000116977|transcript|ENST00000529489|protein_coding|3/3|c.46-5224T>C||||||WARNING_TRANSCRIPT_NO_STOP_CODON,C|intron_variant|MODIFIER|LGALS8|ENSG00000116977|transcript|ENST00000341872|protein_coding|3/10|c.46-5224T>C||||||,C|intron_variant|MODIFIER|LGALS8|ENSG00000116977|transcript|ENST00000450372|protein_coding|3/11|c.46-5224T>C||||||,C|intron_variant|MODIFIER|LGALS8|ENSG00000116977|transcript|ENST00000366584|protein_coding|2/9|c.46-5224T>C||||||,C|intron_variant|MODIFIER|LGALS8|ENSG00000116977|transcript|ENST00000238181|protein_coding|2/6|c.46-5224T>C||||||WARNING_TRANSCRIPT_INCOMPLETE,C|intron_variant|MODIFIER|LGALS8|ENSG00000116977|transcript|ENST00000532826|retained_intron|2/3|n.226-5224T>C||||||,C|intron_variant|MODIFIER|LGALS8|ENSG00000116977|transcript|ENST00000528782|retained_intron|2/4|n.226-5224T>C||||||,C|intron_variant|MODIFIER|LGALS8|ENSG00000116977|transcript|ENST00000366583|retained_intron|2/6|n.222-5224T>C||||||,C|intron_variant|MODIFIER|LGALS8|ENSG00000116977|transcript|ENST00000442397|nonsense_mediated_decay|2/4|c.46-5224T>C||||||,C|intron_variant|MODIFIER|LGALS8|ENSG00000116977|transcript|ENST00000434231|nonsense_mediated_decay|2/4|c.45+6158T>C||||||,C|intron_variant|MODIFIER|LGALS8|ENSG00000116977|transcript|ENST00000416919|protein_coding|2/8|c.46-5224T>C||||||,C|intron_variant|MODIFIER|LGALS8|ENSG00000116977|transcript|ENST00000323938|protein_coding|2/9|c.46-5224T>C||||||,C|intron_variant|MODIFIER|LGALS8|ENSG00000116977|transcript|ENST00000526634|protein_coding|2/9|c.46-5224T>C||||||,C|intron_variant|MODIFIER|LGALS8|ENSG00000116977|transcript|ENST00000525042|protein_coding|1/7|c.46-5224T>C|||||| |
| MTR-RPL35P1 | rs6686489 | 37 | 1 | 237091429 | 237091429 | + | SNP | G | G | T | SRR8586412 | Missense_Mutation | 0/1:34,73:107:99:1557,0,405 | 146;ANN=T|intergenic_region|MODIFIER|MTR-RPL35P1|ENSG00000116984-ENSG00000237991|intergenic_region|ENSG00000116984-ENSG00000237991|||n.237091429G>T|||||| |
| MTR-RPL35P1 | rs6686490 | 37 | 1 | 237091430 | 237091430 | + | SNP | G | G | T | SRR8586412 | Missense_Mutation | 0/1:41,64:105:99:1430,0,1016 | 144;ANN=T|intergenic_region|MODIFIER|MTR-RPL35P1|ENSG00000116984-ENSG00000237991|intergenic_region|ENSG00000116984-ENSG00000237991|||n.237091430G>T|||||| |
| MTR-RPL35P1 | rs368474001 | 37 | 1 | 237091437 | 237091437 | + | SNP | C | C | G | SRR8586412 | Missense_Mutation | 0/1:67,36:103:99:477,0,1658 | 110;ANN=G|intergenic_region|MODIFIER|MTR-RPL35P1|ENSG00000116984-ENSG00000237991|intergenic_region|ENSG00000116984-ENSG00000237991|||n.237091437C>G|||||| |
| ST8SIA6-PRPF38AP2 | rs35235387 | 37 | 10 | 17591242 | 17591242 | + | DEL | CT | CT | C | SRR8586412 | Frame_Shift_Del | 0/1:32,19:51:99:601,0,1116 | 51;ANN=C|intergenic_region|MODIFIER|ST8SIA6-PRPF38AP2|ENSG00000148488-ENSG00000236582|intergenic_region|ENSG00000148488-ENSG00000236582|||n.17591243delT|||||| |
| AC091487.1-RP11-181F12.1 | rs112448389 | 37 | 10 | 86679519 | 86679519 | + | SNP | G | G | A | SRR8586412 | Missense_Mutation | 0/1:49,44:93:99:928,0,3533 | 137;ANN=A|intergenic_region|MODIFIER|AC091487.1-RP11-181F12.1|ENSG00000238469-ENSG00000237267|intergenic_region|ENSG00000238469-ENSG00000237267|||n.86679519G>A|||||| |
| TSPAN9 | rs58837502 | 37 | 12 | 3211762 | 3211762 | + | SNP | T | T | C | SRR8586412 | Missense_Mutation | 0/1:27,105:132:99:3066,0,464 | 137;ANN=C|intron_variant|MODIFIER|TSPAN9|ENSG00000011105|transcript|ENST00000537971|protein_coding|1/7|c.-18+25143T>C||||||,C|intron_variant|MODIFIER|TSPAN9|ENSG00000011105|transcript|ENST00000444315|nonsense_mediated_decay|2/5|c.-18+18877T>C||||||,C|intron_variant|MODIFIER|TSPAN9|ENSG00000011105|transcript|ENST00000011898|protein_coding|2/8|c.-18+18877T>C|||||| |
| B3GALTL-RXFP2 | rs799526 | 37 | 13 | 31928498 | 31928498 | + | SNP | T | T | C | SRR8586412 | Missense_Mutation | 0/1:12,29:41:99:1182,0,727 | 41;ANN=C|intergenic_region|MODIFIER|B3GALTL-RXFP2|ENSG00000187676-ENSG00000133105|intergenic_region|ENSG00000187676-ENSG00000133105|||n.31928498T>C|||||| |
| PRMT5-AS1 | rs370121893 | 37 | 14 | 23386023 | 23386023 | + | SNP | G | G | A | SRR8586412 | Missense_Mutation | 0/1:157,21:178:99:238,0,4561 | 179;ANN=A|upstream_gene_variant|MODIFIER|PRMT5-AS1|ENSG00000237054|transcript|ENST00000424245|antisense||n.-2642G>A|||||2642|,A|upstream_gene_variant|MODIFIER|PRMT5-AS1|ENSG00000237054|transcript|ENST00000609885|antisense||n.-2807G>A|||||2807|,A|upstream_gene_variant|MODIFIER|PRMT5-AS1|ENSG00000237054|transcript|ENST00000457443|antisense||n.-3825G>A|||||3825|,A|upstream_gene_variant|MODIFIER|PRMT5-AS1|ENSG00000237054|transcript|ENST00000599580|antisense||n.-4097G>A|||||4097|,A|upstream_gene_variant|MODIFIER|PRMT5-AS1|ENSG00000237054|transcript|ENST00000595662|antisense||n.-4224G>A|||||4224|,A|upstream_gene_variant|MODIFIER|PRMT5-AS1|ENSG00000237054|transcript|ENST00000587245|antisense||n.-4224G>A|||||4224|,A|upstream_gene_variant|MODIFIER|PRMT5-AS1|ENSG00000237054|transcript|ENST00000590290|antisense||n.-4227G>A|||||4227|,A|downstream_gene_variant|MODIFIER|PRMT5|ENSG00000100462|transcript|ENST00000324366|protein_coding||c.*4090C>T|||||3697|,A|downstream_gene_variant|MODIFIER|RBM23|ENSG00000100461|transcript|ENST00000553777|processed_transcript||n.*750C>T|||||750|,A|downstream_gene_variant|MODIFIER|PRMT5|ENSG00000100462|transcript|ENST00000397441|protein_coding||c.*4090C>T|||||3711|,A|downstream_gene_variant|MODIFIER|PRMT5|ENSG00000100462|transcript|ENST00000557443|protein_coding||c.*3867C>T|||||3715|WARNING_TRANSCRIPT_NO_START_CODON,A|downstream_gene_variant|MODIFIER|PRMT5|ENSG00000100462|transcript|ENST00000476175|processed_transcript||n.*3715C>T|||||3715|,A|downstream_gene_variant|MODIFIER|PRMT5|ENSG00000100462|transcript|ENST00000397440|protein_coding||c.*4090C>T|||||3715|,A|downstream_gene_variant|MODIFIER|PRMT5|ENSG00000100462|transcript|ENST00000216350|protein_coding||c.*4090C>T|||||3716|,A|downstream_gene_variant|MODIFIER|PRMT5|ENSG00000100462|transcript|ENST00000555454|protein_coding||c.*4233C>T|||||3996|WARNING_TRANSCRIPT_NO_START_CODON,A|downstream_gene_variant|MODIFIER|PRMT5|ENSG00000100462|transcript|ENST00000454731|protein_coding||c.*4090C>T|||||4011|WARNING_TRANSCRIPT_NO_START_CODON,A|downstream_gene_variant|MODIFIER|PRMT5|ENSG00000100462|transcript|ENST00000553915|nonsense_mediated_decay||c.*10782C>T|||||4043|,A|downstream_gene_variant|MODIFIER|PRMT5|ENSG00000100462|transcript|ENST00000538452|protein_coding||c.*4090C>T|||||4043|,A|downstream_gene_variant|MODIFIER|PRMT5|ENSG00000100462|transcript|ENST00000553897|protein_coding||c.*4090C>T|||||4062|,A|intron_variant|MODIFIER|RBM23|ENSG00000100461|transcript|ENST00000359890|protein_coding|1/13|c.-11+2185C>T||||||,A|intron_variant|MODIFIER|RBM23|ENSG00000100461|transcript|ENST00000555209|protein_coding|1/10|c.-493+2185C>T||||||,A|intron_variant|MODIFIER|RBM23|ENSG00000100461|transcript|ENST00000557667|retained_intron|1/13|n.138+2185C>T||||||,A|intron_variant|MODIFIER|RBM23|ENSG00000100461|transcript|ENST00000399922|protein_coding|1/12|c.-11+2185C>T||||||,A|intron_variant|MODIFIER|RBM23|ENSG00000100461|transcript|ENST00000346528|protein_coding|1/11|c.-11+2185C>T||||||,A|intron_variant|MODIFIER|RBM23|ENSG00000100461|transcript|ENST00000542016|protein_coding|1/11|c.-360+2185C>T||||||,A|intron_variant|MODIFIER|RBM23|ENSG00000100461|transcript|ENST00000307814|retained_intron|1/10|n.151+2185C>T||||||,A|intron_variant|MODIFIER|RBM23|ENSG00000100461|transcript|ENST00000557403|protein_coding|1/5|c.-343+2185C>T||||||WARNING_TRANSCRIPT_INCOMPLETE,A|intron_variant|MODIFIER|RBM23|ENSG00000100461|transcript|ENST00000555722|protein_coding|1/4|c.-284+2185C>T||||||WARNING_TRANSCRIPT_INCOMPLETE,A|intron_variant|MODIFIER|RBM23|ENSG00000100461|transcript|ENST00000553920|processed_transcript|1/4|n.138+2185C>T||||||,A|intron_variant|MODIFIER|RBM23|ENSG00000100461|transcript|ENST00000557227|protein_coding|1/3|c.-306+2185C>T||||||WARNING_TRANSCRIPT_NO_STOP_CODON,A|intron_variant|MODIFIER|RBM23|ENSG00000100461|transcript|ENST00000555714|processed_transcript|1/5|n.151+2185C>T||||||,A|intron_variant|MODIFIER|RBM23|ENSG00000100461|transcript|ENST00000554256|protein_coding|1/5|c.-11+2185C>T||||||WARNING_TRANSCRIPT_NO_STOP_CODON,A|intron_variant|MODIFIER|RBM23|ENSG00000100461|transcript|ENST00000556687|retained_intron|1/3|n.151+2185C>T||||||,A|intron_variant|MODIFIER|RBM23|ENSG00000100461|transcript|ENST00000553902|retained_intron|1/2|n.151+2185C>T||||||,A|intron_variant|MODIFIER|RBM23|ENSG00000100461|transcript|ENST00000554955|retained_intron|1/4|n.112+2185C>T||||||,A|intron_variant|MODIFIER|RBM23|ENSG00000100461|transcript|ENST00000556984|processed_transcript|1/4|n.151+2185C>T||||||,A|intron_variant|MODIFIER|RBM23|ENSG00000100461|transcript|ENST00000557549|protein_coding|1/4|c.-129+2185C>T||||||WARNING_TRANSCRIPT_NO_STOP_CODON,A|intron_variant|MODIFIER|RBM23|ENSG00000100461|transcript|ENST00000555676|protein_coding|1/4|c.-133+2185C>T||||||WARNING_TRANSCRIPT_NO_STOP_CODON,A|intron_variant|MODIFIER|RBM23|ENSG00000100461|transcript|ENST00000557571|protein_coding|2/4|c.-11+214C>T||||||WARNING_TRANSCRIPT_NO_STOP_CODON,A|intron_variant|MODIFIER|RBM23|ENSG00000100461|transcript|ENST00000556862|protein_coding|1/5|c.-133+2185C>T||||||WARNING_TRANSCRIPT_INCOMPLETE,A|intron_variant|MODIFIER|RBM23|ENSG00000100461|transcript|ENST00000557464|protein_coding|1/4|c.-109+2185C>T||||||WARNING_TRANSCRIPT_NO_STOP_CODON,A|intron_variant|MODIFIER|RBM23|ENSG00000100461|transcript|ENST00000554618|protein_coding|1/5|c.-211+2185C>T||||||WARNING_TRANSCRIPT_INCOMPLETE,A|intron_variant|MODIFIER|RBM23|ENSG00000100461|transcript|ENST00000556365|processed_transcript|1/3|n.151+2185C>T||||||,A|intron_variant|MODIFIER|RBM23|ENSG00000100461|transcript|ENST00000553876|protein_coding|1/2|c.-11+665C>T||||||WARNING_TRANSCRIPT_NO_STOP_CODON |
| PRMT5-AS1 | rs45574136 | 37 | 14 | 23386496 | 23386496 | + | SNP | T | T | A | SRR8586412 | Missense_Mutation | 0/1:192,877:1069:99:34092,0,4994 | 1069;ANN=A|upstream_gene_variant|MODIFIER|PRMT5-AS1|ENSG00000237054|transcript|ENST00000424245|antisense||n.-2169T>A|||||2169|,A|upstream_gene_variant|MODIFIER|PRMT5-AS1|ENSG00000237054|transcript|ENST00000609885|antisense||n.-2334T>A|||||2334|,A|upstream_gene_variant|MODIFIER|PRMT5-AS1|ENSG00000237054|transcript|ENST00000457443|antisense||n.-3352T>A|||||3352|,A|upstream_gene_variant|MODIFIER|PRMT5-AS1|ENSG00000237054|transcript|ENST00000599580|antisense||n.-3624T>A|||||3624|,A|upstream_gene_variant|MODIFIER|PRMT5-AS1|ENSG00000237054|transcript|ENST00000595662|antisense||n.-3751T>A|||||3751|,A|upstream_gene_variant|MODIFIER|PRMT5-AS1|ENSG00000237054|transcript|ENST00000587245|antisense||n.-3751T>A|||||3751|,A|upstream_gene_variant|MODIFIER|PRMT5-AS1|ENSG00000237054|transcript|ENST00000590290|antisense||n.-3754T>A|||||3754|,A|downstream_gene_variant|MODIFIER|PRMT5|ENSG00000100462|transcript|ENST00000324366|protein_coding||c.*3617A>T|||||3224|,A|downstream_gene_variant|MODIFIER|RBM23|ENSG00000100461|transcript|ENST00000553777|processed_transcript||n.*277A>T|||||277|,A|downstream_gene_variant|MODIFIER|PRMT5|ENSG00000100462|transcript|ENST00000397441|protein_coding||c.*3617A>T|||||3238|,A|downstream_gene_variant|MODIFIER|PRMT5|ENSG00000100462|transcript|ENST00000557443|protein_coding||c.*3394A>T|||||3242|WARNING_TRANSCRIPT_NO_START_CODON,A|downstream_gene_variant|MODIFIER|PRMT5|ENSG00000100462|transcript|ENST00000476175|processed_transcript||n.*3242A>T|||||3242|,A|downstream_gene_variant|MODIFIER|PRMT5|ENSG00000100462|transcript|ENST00000397440|protein_coding||c.*3617A>T|||||3242|,A|downstream_gene_variant|MODIFIER|PRMT5|ENSG00000100462|transcript|ENST00000216350|protein_coding||c.*3617A>T|||||3243|,A|downstream_gene_variant|MODIFIER|PRMT5|ENSG00000100462|transcript|ENST00000555454|protein_coding||c.*3760A>T|||||3523|WARNING_TRANSCRIPT_NO_START_CODON,A|downstream_gene_variant|MODIFIER|PRMT5|ENSG00000100462|transcript|ENST00000454731|protein_coding||c.*3617A>T|||||3538|WARNING_TRANSCRIPT_NO_START_CODON,A|downstream_gene_variant|MODIFIER|PRMT5|ENSG00000100462|transcript|ENST00000553915|nonsense_mediated_decay||c.*10309A>T|||||3570|,A|downstream_gene_variant|MODIFIER|PRMT5|ENSG00000100462|transcript|ENST00000538452|protein_coding||c.*3617A>T|||||3570|,A|downstream_gene_variant|MODIFIER|PRMT5|ENSG00000100462|transcript|ENST00000553897|protein_coding||c.*3617A>T|||||3589|,A|downstream_gene_variant|MODIFIER|PRMT5|ENSG00000100462|transcript|ENST00000557758|retained_intron||n.*4845A>T|||||4845|,A|intron_variant|MODIFIER|RBM23|ENSG00000100461|transcript|ENST00000359890|protein_coding|1/13|c.-11+1712A>T||||||,A|intron_variant|MODIFIER|RBM23|ENSG00000100461|transcript|ENST00000555209|protein_coding|1/10|c.-493+1712A>T||||||,A|intron_variant|MODIFIER|RBM23|ENSG00000100461|transcript|ENST00000557667|retained_intron|1/13|n.138+1712A>T||||||,A|intron_variant|MODIFIER|RBM23|ENSG00000100461|transcript|ENST00000399922|protein_coding|1/12|c.-11+1712A>T||||||,A|intron_variant|MODIFIER|RBM23|ENSG00000100461|transcript|ENST00000346528|protein_coding|1/11|c.-11+1712A>T||||||,A|intron_variant|MODIFIER|RBM23|ENSG00000100461|transcript|ENST00000542016|protein_coding|1/11|c.-360+1712A>T||||||,A|intron_variant|MODIFIER|RBM23|ENSG00000100461|transcript|ENST00000307814|retained_intron|1/10|n.151+1712A>T||||||,A|intron_variant|MODIFIER|RBM23|ENSG00000100461|transcript|ENST00000557403|protein_coding|1/5|c.-343+1712A>T||||||WARNING_TRANSCRIPT_INCOMPLETE,A|intron_variant|MODIFIER|RBM23|ENSG00000100461|transcript|ENST00000555722|protein_coding|1/4|c.-284+1712A>T||||||WARNING_TRANSCRIPT_INCOMPLETE,A|intron_variant|MODIFIER|RBM23|ENSG00000100461|transcript|ENST00000553920|processed_transcript|1/4|n.138+1712A>T||||||,A|intron_variant|MODIFIER|RBM23|ENSG00000100461|transcript|ENST00000557227|protein_coding|1/3|c.-306+1712A>T||||||WARNING_TRANSCRIPT_NO_STOP_CODON,A|intron_variant|MODIFIER|RBM23|ENSG00000100461|transcript|ENST00000555714|processed_transcript|1/5|n.151+1712A>T||||||,A|intron_variant|MODIFIER|RBM23|ENSG00000100461|transcript|ENST00000554256|protein_coding|1/5|c.-11+1712A>T||||||WARNING_TRANSCRIPT_NO_STOP_CODON,A|intron_variant|MODIFIER|RBM23|ENSG00000100461|transcript|ENST00000556687|retained_intron|1/3|n.151+1712A>T||||||,A|intron_variant|MODIFIER|RBM23|ENSG00000100461|transcript|ENST00000553902|retained_intron|1/2|n.151+1712A>T||||||,A|intron_variant|MODIFIER|RBM23|ENSG00000100461|transcript|ENST00000554955|retained_intron|1/4|n.112+1712A>T||||||,A|intron_variant|MODIFIER|RBM23|ENSG00000100461|transcript|ENST00000556984|processed_transcript|1/4|n.151+1712A>T||||||,A|intron_variant|MODIFIER|RBM23|ENSG00000100461|transcript|ENST00000557549|protein_coding|1/4|c.-129+1712A>T||||||WARNING_TRANSCRIPT_NO_STOP_CODON,A|intron_variant|MODIFIER|RBM23|ENSG00000100461|transcript|ENST00000555676|protein_coding|1/4|c.-133+1712A>T||||||WARNING_TRANSCRIPT_NO_STOP_CODON,A|intron_variant|MODIFIER|RBM23|ENSG00000100461|transcript|ENST00000557571|protein_coding|1/4|c.-149-121A>T||||||WARNING_TRANSCRIPT_NO_STOP_CODON,A|intron_variant|MODIFIER|RBM23|ENSG00000100461|transcript|ENST00000556862|protein_coding|1/5|c.-133+1712A>T||||||WARNING_TRANSCRIPT_INCOMPLETE,A|intron_variant|MODIFIER|RBM23|ENSG00000100461|transcript|ENST00000557464|protein_coding|1/4|c.-109+1712A>T||||||WARNING_TRANSCRIPT_NO_STOP_CODON,A|intron_variant|MODIFIER|RBM23|ENSG00000100461|transcript|ENST00000554618|protein_coding|1/5|c.-211+1712A>T||||||WARNING_TRANSCRIPT_INCOMPLETE,A|intron_variant|MODIFIER|RBM23|ENSG00000100461|transcript|ENST00000556365|processed_transcript|1/3|n.151+1712A>T||||||,A|intron_variant|MODIFIER|RBM23|ENSG00000100461|transcript|ENST00000553876|protein_coding|1/2|c.-11+192A>T||||||WARNING_TRANSCRIPT_NO_STOP_CODON |
| PRMT5-AS1 | rs374702183 | 37 | 14 | 23386579 | 23386579 | + | SNP | T | T | C | SRR8586412 | Missense_Mutation | 0/1:57,313:370:99:9689,0,705 | 370;ANN=C|upstream_gene_variant|MODIFIER|PRMT5-AS1|ENSG00000237054|transcript|ENST00000424245|antisense||n.-2086T>C|||||2086|,C|upstream_gene_variant|MODIFIER|PRMT5-AS1|ENSG00000237054|transcript|ENST00000609885|antisense||n.-2251T>C|||||2251|,C|upstream_gene_variant|MODIFIER|PRMT5-AS1|ENSG00000237054|transcript|ENST00000457443|antisense||n.-3269T>C|||||3269|,C|upstream_gene_variant|MODIFIER|PRMT5-AS1|ENSG00000237054|transcript|ENST00000599580|antisense||n.-3541T>C|||||3541|,C|upstream_gene_variant|MODIFIER|PRMT5-AS1|ENSG00000237054|transcript|ENST00000595662|antisense||n.-3668T>C|||||3668|,C|upstream_gene_variant|MODIFIER|PRMT5-AS1|ENSG00000237054|transcript|ENST00000587245|antisense||n.-3668T>C|||||3668|,C|upstream_gene_variant|MODIFIER|PRMT5-AS1|ENSG00000237054|transcript|ENST00000590290|antisense||n.-3671T>C|||||3671|,C|downstream_gene_variant|MODIFIER|PRMT5|ENSG00000100462|transcript|ENST00000324366|protein_coding||c.*3534A>G|||||3141|,C|downstream_gene_variant|MODIFIER|RBM23|ENSG00000100461|transcript|ENST00000553777|processed_transcript||n.*194A>G|||||194|,C|downstream_gene_variant|MODIFIER|PRMT5|ENSG00000100462|transcript|ENST00000397441|protein_coding||c.*3534A>G|||||3155|,C|downstream_gene_variant|MODIFIER|PRMT5|ENSG00000100462|transcript|ENST00000557443|protein_coding||c.*3311A>G|||||3159|WARNING_TRANSCRIPT_NO_START_CODON,C|downstream_gene_variant|MODIFIER|PRMT5|ENSG00000100462|transcript|ENST00000476175|processed_transcript||n.*3159A>G|||||3159|,C|downstream_gene_variant|MODIFIER|PRMT5|ENSG00000100462|transcript|ENST00000397440|protein_coding||c.*3534A>G|||||3159|,C|downstream_gene_variant|MODIFIER|PRMT5|ENSG00000100462|transcript|ENST00000216350|protein_coding||c.*3534A>G|||||3160|,C|downstream_gene_variant|MODIFIER|PRMT5|ENSG00000100462|transcript|ENST00000555454|protein_coding||c.*3677A>G|||||3440|WARNING_TRANSCRIPT_NO_START_CODON,C|downstream_gene_variant|MODIFIER|PRMT5|ENSG00000100462|transcript|ENST00000454731|protein_coding||c.*3534A>G|||||3455|WARNING_TRANSCRIPT_NO_START_CODON,C|downstream_gene_variant|MODIFIER|PRMT5|ENSG00000100462|transcript|ENST00000553915|nonsense_mediated_decay||c.*10226A>G|||||3487|,C|downstream_gene_variant|MODIFIER|PRMT5|ENSG00000100462|transcript|ENST00000538452|protein_coding||c.*3534A>G|||||3487|,C|downstream_gene_variant|MODIFIER|PRMT5|ENSG00000100462|transcript|ENST00000553897|protein_coding||c.*3534A>G|||||3506|,C|downstream_gene_variant|MODIFIER|PRMT5|ENSG00000100462|transcript|ENST00000557758|retained_intron||n.*4762A>G|||||4762|,C|intron_variant|MODIFIER|RBM23|ENSG00000100461|transcript|ENST00000359890|protein_coding|1/13|c.-11+1629A>G||||||,C|intron_variant|MODIFIER|RBM23|ENSG00000100461|transcript|ENST00000555209|protein_coding|1/10|c.-493+1629A>G||||||,C|intron_variant|MODIFIER|RBM23|ENSG00000100461|transcript|ENST00000557667|retained_intron|1/13|n.138+1629A>G||||||,C|intron_variant|MODIFIER|RBM23|ENSG00000100461|transcript|ENST00000399922|protein_coding|1/12|c.-11+1629A>G||||||,C|intron_variant|MODIFIER|RBM23|ENSG00000100461|transcript|ENST00000346528|protein_coding|1/11|c.-11+1629A>G||||||,C|intron_variant|MODIFIER|RBM23|ENSG00000100461|transcript|ENST00000542016|protein_coding|1/11|c.-360+1629A>G||||||,C|intron_variant|MODIFIER|RBM23|ENSG00000100461|transcript|ENST00000307814|retained_intron|1/10|n.151+1629A>G||||||,C|intron_variant|MODIFIER|RBM23|ENSG00000100461|transcript|ENST00000557403|protein_coding|1/5|c.-343+1629A>G||||||WARNING_TRANSCRIPT_INCOMPLETE,C|intron_variant|MODIFIER|RBM23|ENSG00000100461|transcript|ENST00000555722|protein_coding|1/4|c.-284+1629A>G||||||WARNING_TRANSCRIPT_INCOMPLETE,C|intron_variant|MODIFIER|RBM23|ENSG00000100461|transcript|ENST00000553920|processed_transcript|1/4|n.138+1629A>G||||||,C|intron_variant|MODIFIER|RBM23|ENSG00000100461|transcript|ENST00000557227|protein_coding|1/3|c.-306+1629A>G||||||WARNING_TRANSCRIPT_NO_STOP_CODON,C|intron_variant|MODIFIER|RBM23|ENSG00000100461|transcript|ENST00000555714|processed_transcript|1/5|n.151+1629A>G||||||,C|intron_variant|MODIFIER|RBM23|ENSG00000100461|transcript|ENST00000554256|protein_coding|1/5|c.-11+1629A>G||||||WARNING_TRANSCRIPT_NO_STOP_CODON,C|intron_variant|MODIFIER|RBM23|ENSG00000100461|transcript|ENST00000556687|retained_intron|1/3|n.151+1629A>G||||||,C|intron_variant|MODIFIER|RBM23|ENSG00000100461|transcript|ENST00000553902|retained_intron|1/2|n.151+1629A>G||||||,C|intron_variant|MODIFIER|RBM23|ENSG00000100461|transcript|ENST00000554955|retained_intron|1/4|n.112+1629A>G||||||,C|intron_variant|MODIFIER|RBM23|ENSG00000100461|transcript|ENST00000556984|processed_transcript|1/4|n.151+1629A>G||||||,C|intron_variant|MODIFIER|RBM23|ENSG00000100461|transcript|ENST00000557549|protein_coding|1/4|c.-129+1629A>G||||||WARNING_TRANSCRIPT_NO_STOP_CODON,C|intron_variant|MODIFIER|RBM23|ENSG00000100461|transcript|ENST00000555676|protein_coding|1/4|c.-133+1629A>G||||||WARNING_TRANSCRIPT_NO_STOP_CODON,C|intron_variant|MODIFIER|RBM23|ENSG00000100461|transcript|ENST00000557571|protein_coding|1/4|c.-149-204A>G||||||WARNING_TRANSCRIPT_NO_STOP_CODON,C|intron_variant|MODIFIER|RBM23|ENSG00000100461|transcript|ENST00000556862|protein_coding|1/5|c.-133+1629A>G||||||WARNING_TRANSCRIPT_INCOMPLETE,C|intron_variant|MODIFIER|RBM23|ENSG00000100461|transcript|ENST00000557464|protein_coding|1/4|c.-109+1629A>G||||||WARNING_TRANSCRIPT_NO_STOP_CODON,C|intron_variant|MODIFIER|RBM23|ENSG00000100461|transcript|ENST00000554618|protein_coding|1/5|c.-211+1629A>G||||||WARNING_TRANSCRIPT_INCOMPLETE,C|intron_variant|MODIFIER|RBM23|ENSG00000100461|transcript|ENST00000556365|processed_transcript|1/3|n.151+1629A>G||||||,C|intron_variant|MODIFIER|RBM23|ENSG00000100461|transcript|ENST00000553876|protein_coding|1/2|c.-11+109A>G||||||WARNING_TRANSCRIPT_NO_STOP_CODON |
| PRMT5-AS1 | rs150081280 | 37 | 14 | 23387882 | 23387882 | + | SNP | A | A | C | SRR8586412 | Missense_Mutation | 0/1:223,95:318:99:3284,0,12669 | 318;ANN=C|upstream_gene_variant|MODIFIER|PRMT5-AS1|ENSG00000237054|transcript|ENST00000424245|antisense||n.-783A>C|||||783|,C|upstream_gene_variant|MODIFIER|RBM23|ENSG00000100461|transcript|ENST00000553876|protein_coding||c.-7280T>G|||||1117|WARNING_TRANSCRIPT_NO_STOP_CODON,C|upstream_gene_variant|MODIFIER|PRMT5-AS1|ENSG00000237054|transcript|ENST00000609885|antisense||n.-948A>C|||||948|,C|upstream_gene_variant|MODIFIER|PRMT5-AS1|ENSG00000237054|transcript|ENST00000457443|antisense||n.-1966A>C|||||1966|,C|upstream_gene_variant|MODIFIER|PRMT5-AS1|ENSG00000237054|transcript|ENST00000599580|antisense||n.-2238A>C|||||2238|,C|upstream_gene_variant|MODIFIER|PRMT5-AS1|ENSG00000237054|transcript|ENST00000595662|antisense||n.-2365A>C|||||2365|,C|upstream_gene_variant|MODIFIER|PRMT5-AS1|ENSG00000237054|transcript|ENST00000587245|antisense||n.-2365A>C|||||2365|,C|upstream_gene_variant|MODIFIER|PRMT5-AS1|ENSG00000237054|transcript|ENST00000590290|antisense||n.-2368A>C|||||2368|,C|downstream_gene_variant|MODIFIER|PRMT5|ENSG00000100462|transcript|ENST00000324366|protein_coding||c.*2231T>G|||||1838|,C|downstream_gene_variant|MODIFIER|PRMT5|ENSG00000100462|transcript|ENST00000397441|protein_coding||c.*2231T>G|||||1852|,C|downstream_gene_variant|MODIFIER|PRMT5|ENSG00000100462|transcript|ENST00000557443|protein_coding||c.*2008T>G|||||1856|WARNING_TRANSCRIPT_NO_START_CODON,C|downstream_gene_variant|MODIFIER|PRMT5|ENSG00000100462|transcript|ENST00000476175|processed_transcript||n.*1856T>G|||||1856|,C|downstream_gene_variant|MODIFIER|PRMT5|ENSG00000100462|transcript|ENST00000397440|protein_coding||c.*2231T>G|||||1856|,C|downstream_gene_variant|MODIFIER|PRMT5|ENSG00000100462|transcript|ENST00000216350|protein_coding||c.*2231T>G|||||1857|,C|downstream_gene_variant|MODIFIER|PRMT5|ENSG00000100462|transcript|ENST00000555454|protein_coding||c.*2374T>G|||||2137|WARNING_TRANSCRIPT_NO_START_CODON,C|downstream_gene_variant|MODIFIER|PRMT5|ENSG00000100462|transcript|ENST00000454731|protein_coding||c.*2231T>G|||||2152|WARNING_TRANSCRIPT_NO_START_CODON,C|downstream_gene_variant|MODIFIER|PRMT5|ENSG00000100462|transcript|ENST00000553915|nonsense_mediated_decay||c.*8923T>G|||||2184|,C|downstream_gene_variant|MODIFIER|PRMT5|ENSG00000100462|transcript|ENST00000538452|protein_coding||c.*2231T>G|||||2184|,C|downstream_gene_variant|MODIFIER|PRMT5|ENSG00000100462|transcript|ENST00000553897|protein_coding||c.*2231T>G|||||2203|,C|downstream_gene_variant|MODIFIER|PRMT5|ENSG00000100462|transcript|ENST00000557758|retained_intron||n.*3459T>G|||||3459|,C|intron_variant|MODIFIER|RBM23|ENSG00000100461|transcript|ENST00000359890|protein_coding|1/13|c.-11+326T>G||||||,C|intron_variant|MODIFIER|RBM23|ENSG00000100461|transcript|ENST00000555209|protein_coding|1/10|c.-493+326T>G||||||,C|intron_variant|MODIFIER|RBM23|ENSG00000100461|transcript|ENST00000557667|retained_intron|1/13|n.138+326T>G||||||,C|intron_variant|MODIFIER|RBM23|ENSG00000100461|transcript|ENST00000399922|protein_coding|1/12|c.-11+326T>G||||||,C|intron_variant|MODIFIER|RBM23|ENSG00000100461|transcript|ENST00000346528|protein_coding|1/11|c.-11+326T>G||||||,C|intron_variant|MODIFIER|RBM23|ENSG00000100461|transcript|ENST00000542016|protein_coding|1/11|c.-360+326T>G||||||,C|intron_variant|MODIFIER|RBM23|ENSG00000100461|transcript|ENST00000307814|retained_intron|1/10|n.151+326T>G||||||,C|intron_variant|MODIFIER|RBM23|ENSG00000100461|transcript|ENST00000557403|protein_coding|1/5|c.-343+326T>G||||||WARNING_TRANSCRIPT_INCOMPLETE,C|intron_variant|MODIFIER|RBM23|ENSG00000100461|transcript|ENST00000555722|protein_coding|1/4|c.-284+326T>G||||||WARNING_TRANSCRIPT_INCOMPLETE,C|intron_variant|MODIFIER|RBM23|ENSG00000100461|transcript|ENST00000553920|processed_transcript|1/4|n.138+326T>G||||||,C|intron_variant|MODIFIER|RBM23|ENSG00000100461|transcript|ENST00000557227|protein_coding|1/3|c.-306+326T>G||||||WARNING_TRANSCRIPT_NO_STOP_CODON,C|intron_variant|MODIFIER|RBM23|ENSG00000100461|transcript|ENST00000555714|processed_transcript|1/5|n.151+326T>G||||||,C|intron_variant|MODIFIER|RBM23|ENSG00000100461|transcript|ENST00000554256|protein_coding|1/5|c.-11+326T>G||||||WARNING_TRANSCRIPT_NO_STOP_CODON,C|intron_variant|MODIFIER|RBM23|ENSG00000100461|transcript|ENST00000556687|retained_intron|1/3|n.151+326T>G||||||,C|intron_variant|MODIFIER|RBM23|ENSG00000100461|transcript|ENST00000553902|retained_intron|1/2|n.151+326T>G||||||,C|intron_variant|MODIFIER|RBM23|ENSG00000100461|transcript|ENST00000554955|retained_intron|1/4|n.112+326T>G||||||,C|intron_variant|MODIFIER|RBM23|ENSG00000100461|transcript|ENST00000556984|processed_transcript|1/4|n.151+326T>G||||||,C|intron_variant|MODIFIER|RBM23|ENSG00000100461|transcript|ENST00000557549|protein_coding|1/4|c.-129+326T>G||||||WARNING_TRANSCRIPT_NO_STOP_CODON,C|intron_variant|MODIFIER|RBM23|ENSG00000100461|transcript|ENST00000555676|protein_coding|1/4|c.-133+326T>G||||||WARNING_TRANSCRIPT_NO_STOP_CODON,C|intron_variant|MODIFIER|RBM23|ENSG00000100461|transcript|ENST00000557571|protein_coding|1/4|c.-150+326T>G||||||WARNING_TRANSCRIPT_NO_STOP_CODON,C|intron_variant|MODIFIER|RBM23|ENSG00000100461|transcript|ENST00000556862|protein_coding|1/5|c.-133+326T>G||||||WARNING_TRANSCRIPT_INCOMPLETE,C|intron_variant|MODIFIER|RBM23|ENSG00000100461|transcript|ENST00000557464|protein_coding|1/4|c.-109+326T>G||||||WARNING_TRANSCRIPT_NO_STOP_CODON,C|intron_variant|MODIFIER|RBM23|ENSG00000100461|transcript|ENST00000554618|protein_coding|1/5|c.-211+326T>G||||||WARNING_TRANSCRIPT_INCOMPLETE,C|intron_variant|MODIFIER|RBM23|ENSG00000100461|transcript|ENST00000556365|processed_transcript|1/3|n.151+326T>G||||||,C|intron_variant|MODIFIER|RBM23|ENSG00000100461|transcript|ENST00000553777|processed_transcript|1/1|n.89+326T>G|||||| |
| RBM23 | rs780653498 | 37 | 14 | 23388975 | 23388975 | + | SNP | T | T | C | SRR8586412 | Missense_Mutation | 0/1:104,83:187:99:2239,0,2867 | 188;ANN=C|upstream_gene_variant|MODIFIER|RBM23|ENSG00000100461|transcript|ENST00000359890|protein_coding||c.-8373A>G|||||582|,C|upstream_gene_variant|MODIFIER|RBM23|ENSG00000100461|transcript|ENST00000555209|protein_coding||c.-14778A>G|||||637|,C|upstream_gene_variant|MODIFIER|RBM23|ENSG00000100461|transcript|ENST00000557667|retained_intron||n.-630A>G|||||630|,C|upstream_gene_variant|MODIFIER|RBM23|ENSG00000100461|transcript|ENST00000399922|protein_coding||c.-8373A>G|||||584|,C|upstream_gene_variant|MODIFIER|RBM23|ENSG00000100461|transcript|ENST00000346528|protein_coding||c.-8373A>G|||||588|,C|upstream_gene_variant|MODIFIER|RBM23|ENSG00000100461|transcript|ENST00000542016|protein_coding||c.-14368A>G|||||588|,C|upstream_gene_variant|MODIFIER|RBM23|ENSG00000100461|transcript|ENST00000307814|retained_intron||n.-617A>G|||||617|,C|upstream_gene_variant|MODIFIER|RBM23|ENSG00000100461|transcript|ENST00000557403|protein_coding||c.-14368A>G|||||628|WARNING_TRANSCRIPT_INCOMPLETE,C|upstream_gene_variant|MODIFIER|RBM23|ENSG00000100461|transcript|ENST00000555722|protein_coding||c.-14368A>G|||||601|WARNING_TRANSCRIPT_INCOMPLETE,C|upstream_gene_variant|MODIFIER|RBM23|ENSG00000100461|transcript|ENST00000553920|processed_transcript||n.-630A>G|||||630|,C|upstream_gene_variant|MODIFIER|RBM23|ENSG00000100461|transcript|ENST00000557227|protein_coding||c.-14368A>G|||||584|WARNING_TRANSCRIPT_NO_STOP_CODON,C|upstream_gene_variant|MODIFIER|RBM23|ENSG00000100461|transcript|ENST00000555714|processed_transcript||n.-617A>G|||||617|,C|upstream_gene_variant|MODIFIER|RBM23|ENSG00000100461|transcript|ENST00000554256|protein_coding||c.-8373A>G|||||629|WARNING_TRANSCRIPT_NO_STOP_CODON,C|upstream_gene_variant|MODIFIER|RBM23|ENSG00000100461|transcript|ENST00000556687|retained_intron||n.-617A>G|||||617|,C|upstream_gene_variant|MODIFIER|RBM23|ENSG00000100461|transcript|ENST00000553902|retained_intron||n.-617A>G|||||617|,C|upstream_gene_variant|MODIFIER|RBM23|ENSG00000100461|transcript|ENST00000554955|retained_intron||n.-656A>G|||||656|,C|upstream_gene_variant|MODIFIER|RBM23|ENSG00000100461|transcript|ENST00000556984|processed_transcript||n.-617A>G|||||617|,C|upstream_gene_variant|MODIFIER|RBM23|ENSG00000100461|transcript|ENST00000557549|protein_coding||c.-8373A>G|||||617|WARNING_TRANSCRIPT_NO_STOP_CODON,C|upstream_gene_variant|MODIFIER|RBM23|ENSG00000100461|transcript|ENST00000555676|protein_coding||c.-8373A>G|||||617|WARNING_TRANSCRIPT_NO_STOP_CODON,C|upstream_gene_variant|MODIFIER|RBM23|ENSG00000100461|transcript|ENST00000557571|protein_coding||c.-8373A>G|||||617|WARNING_TRANSCRIPT_NO_STOP_CODON,C|upstream_gene_variant|MODIFIER|RBM23|ENSG00000100461|transcript|ENST00000556862|protein_coding||c.-8373A>G|||||609|WARNING_TRANSCRIPT_INCOMPLETE,C|upstream_gene_variant|MODIFIER|RBM23|ENSG00000100461|transcript|ENST00000557464|protein_coding||c.-8373A>G|||||617|WARNING_TRANSCRIPT_NO_STOP_CODON,C|upstream_gene_variant|MODIFIER|RBM23|ENSG00000100461|transcript|ENST00000554618|protein_coding||c.-8373A>G|||||617|WARNING_TRANSCRIPT_INCOMPLETE,C|upstream_gene_variant|MODIFIER|RBM23|ENSG00000100461|transcript|ENST00000556365|processed_transcript||n.-617A>G|||||617|,C|upstream_gene_variant|MODIFIER|RBM23|ENSG00000100461|transcript|ENST00000553876|protein_coding||c.-8373A>G|||||2210|WARNING_TRANSCRIPT_NO_STOP_CODON,C|upstream_gene_variant|MODIFIER|RBM23|ENSG00000100461|transcript|ENST00000553777|processed_transcript||n.-679A>G|||||679|,C|upstream_gene_variant|MODIFIER|PRMT5-AS1|ENSG00000237054|transcript|ENST00000457443|antisense||n.-873T>C|||||873|,C|upstream_gene_variant|MODIFIER|PRMT5-AS1|ENSG00000237054|transcript|ENST00000599580|antisense||n.-1145T>C|||||1145|,C|upstream_gene_variant|MODIFIER|PRMT5-AS1|ENSG00000237054|transcript|ENST00000595662|antisense||n.-1272T>C|||||1272|,C|upstream_gene_variant|MODIFIER|PRMT5-AS1|ENSG00000237054|transcript|ENST00000587245|antisense||n.-1272T>C|||||1272|,C|upstream_gene_variant|MODIFIER|PRMT5-AS1|ENSG00000237054|transcript|ENST00000590290|antisense||n.-1275T>C|||||1275|,C|downstream_gene_variant|MODIFIER|PRMT5|ENSG00000100462|transcript|ENST00000324366|protein_coding||c.*1138A>G|||||745|,C|downstream_gene_variant|MODIFIER|PRMT5|ENSG00000100462|transcript|ENST00000397441|protein_coding||c.*1138A>G|||||759|,C|downstream_gene_variant|MODIFIER|PRMT5|ENSG00000100462|transcript|ENST00000557443|protein_coding||c.*915A>G|||||763|WARNING_TRANSCRIPT_NO_START_CODON,C|downstream_gene_variant|MODIFIER|PRMT5|ENSG00000100462|transcript|ENST00000476175|processed_transcript||n.*763A>G|||||763|,C|downstream_gene_variant|MODIFIER|PRMT5|ENSG00000100462|transcript|ENST00000397440|protein_coding||c.*1138A>G|||||763|,C|downstream_gene_variant|MODIFIER|PRMT5|ENSG00000100462|transcript|ENST00000216350|protein_coding||c.*1138A>G|||||764|,C|downstream_gene_variant|MODIFIER|PRMT5|ENSG00000100462|transcript|ENST00000555454|protein_coding||c.*1281A>G|||||1044|WARNING_TRANSCRIPT_NO_START_CODON,C|downstream_gene_variant|MODIFIER|PRMT5|ENSG00000100462|transcript|ENST00000454731|protein_coding||c.*1138A>G|||||1059|WARNING_TRANSCRIPT_NO_START_CODON,C|downstream_gene_variant|MODIFIER|PRMT5|ENSG00000100462|transcript|ENST00000553915|nonsense_mediated_decay||c.*7830A>G|||||1091|,C|downstream_gene_variant|MODIFIER|PRMT5|ENSG00000100462|transcript|ENST00000538452|protein_coding||c.*1138A>G|||||1091|,C|downstream_gene_variant|MODIFIER|PRMT5|ENSG00000100462|transcript|ENST00000553897|protein_coding||c.*1138A>G|||||1110|,C|downstream_gene_variant|MODIFIER|PRMT5|ENSG00000100462|transcript|ENST00000557758|retained_intron||n.*2366A>G|||||2366|,C|downstream_gene_variant|MODIFIER|PRMT5|ENSG00000100462|transcript|ENST00000553502|protein_coding||c.*4396A>G|||||4396|WARNING_TRANSCRIPT_NO_START_CODON,C|downstream_gene_variant|MODIFIER|PRMT5|ENSG00000100462|transcript|ENST00000555530|protein_coding||c.*4551A>G|||||4551|WARNING_TRANSCRIPT_NO_START_CODON,C|downstream_gene_variant|MODIFIER|PRMT5|ENSG00000100462|transcript|ENST00000556043|protein_coding||c.*4733A>G|||||4733|WARNING_TRANSCRIPT_NO_STOP_CODON,C|downstream_gene_variant|MODIFIER|PRMT5|ENSG00000100462|transcript|ENST00000553550|protein_coding||c.*4872A>G|||||4872|WARNING_TRANSCRIPT_NO_STOP_CODON,C|downstream_gene_variant|MODIFIER|PRMT5|ENSG00000100462|transcript|ENST00000554716|processed_transcript||n.*4897A>G|||||4897|,C|non_coding_transcript_exon_variant|MODIFIER|PRMT5-AS1|ENSG00000237054|transcript|ENST00000424245|antisense|1/2|n.311T>C||||||,C|non_coding_transcript_exon_variant|MODIFIER|PRMT5-AS1|ENSG00000237054|transcript|ENST00000609885|antisense|1/2|n.146T>C|||||| |
| PRMT5 | rs144050960 | 37 | 14 | 23396736 | 23396736 | + | SNP | A | A | G | SRR8586412 | Splice_Site | 0/1:92,23:115:99:697,0,5412 | 116;ANN=G|structural_interaction_variant|HIGH|PRMT5|ENSG00000100462|interaction|4GQB:A_122-A_150:ENST00000324366|protein_coding|4/17|c.449T>C||||||,G|structural_interaction_variant|HIGH|PRMT5|ENSG00000100462|interaction|4X61:A_120-A_150:ENST00000324366|protein_coding|4/17|c.449T>C||||||,G|missense_variant&splice_region_variant|MODERATE|PRMT5|ENSG00000100462|transcript|ENST00000324366|protein_coding|4/17|c.449T>C|p.Met150Thr|673/2531|449/1914|150/637||,G|missense_variant&splice_region_variant|MODERATE|PRMT5|ENSG00000100462|transcript|ENST00000397441|protein_coding|4/17|c.398T>C|p.Met133Thr|574/2418|398/1863|133/620||,G|missense_variant&splice_region_variant|MODERATE|PRMT5|ENSG00000100462|transcript|ENST00000216350|protein_coding|3/16|c.266T>C|p.Met89Thr|432/2271|266/1731|89/576||,G|missense_variant&splice_region_variant|MODERATE|PRMT5|ENSG00000100462|transcript|ENST00000538452|protein_coding|3/16|c.131T>C|p.Met44Thr|421/1933|131/1596|44/531||,G|missense_variant&splice_region_variant|MODERATE|PRMT5|ENSG00000100462|transcript|ENST00000553897|protein_coding|3/16|c.317T>C|p.Met106Thr|364/1857|317/1782|106/593||,G|missense_variant&splice_region_variant|MODERATE|PRMT5|ENSG00000100462|transcript|ENST00000555530|protein_coding|2/9|c.152T>C|p.Met51Thr|152/837|152/837|51/278||WARNING_TRANSCRIPT_NO_START_CODON,G|missense_variant&splice_region_variant|MODERATE|PRMT5|ENSG00000100462|transcript|ENST00000556616|protein_coding|3/6|c.335T>C|p.Met112Thr|350/571|335/556|112/184||WARNING_TRANSCRIPT_INCOMPLETE,G|missense_variant&splice_region_variant|MODERATE|PRMT5|ENSG00000100462|transcript|ENST00000554910|protein_coding|4/7|c.323T>C|p.Met108Thr|391/577|323/509|108/168||WARNING_TRANSCRIPT_INCOMPLETE,G|missense_variant&splice_region_variant|MODERATE|PRMT5|ENSG00000100462|transcript|ENST00000421938|protein_coding|4/5|c.479T>C|p.Met160Thr|486/565|479/558|160/185||WARNING_TRANSCRIPT_NO_STOP_CODON,G|splice_region_variant|LOW|PRMT5|ENSG00000100462|transcript|ENST00000553915|nonsense_mediated_decay|3/16|c.*69T>C||||||,G|splice_region_variant|LOW|PRMT5|ENSG00000100462|transcript|ENST00000553787|nonsense_mediated_decay|2/6|c.*69T>C||||||,G|splice_region_variant&non_coding_transcript_exon_variant|LOW|PRMT5|ENSG00000100462|transcript|ENST00000553641|processed_transcript|4/7|n.472T>C||||||,G|splice_region_variant|LOW|PRMT5|ENSG00000100462|transcript|ENST00000557415|nonsense_mediated_decay|3/6|c.*69T>C||||||,G|splice_region_variant&non_coding_transcript_exon_variant|LOW|PRMT5|ENSG00000100462|transcript|ENST00000553417|retained_intron|1/3|n.70T>C||||||,G|splice_region_variant&non_coding_transcript_exon_variant|LOW|PRMT5|ENSG00000100462|transcript|ENST00000556032|retained_intron|3/3|n.642T>C||||||,G|sequence_feature|LOW|PRMT5|ENSG00000100462|beta-strand:combinatorial_evidence_used_in_manual_assertion|ENST00000324366|protein_coding|4/17|c.449T>C||||||,G|sequence_feature|LOW|PRMT5|ENSG00000100462|beta-strand:combinatorial_evidence_used_in_manual_assertion|ENST00000397441|protein_coding|4/17|c.398T>C||||||,G|sequence_feature|LOW|PRMT5|ENSG00000100462|beta-strand:combinatorial_evidence_used_in_manual_assertion|ENST00000216350|protein_coding|3/16|c.266T>C||||||,G|sequence_feature|LOW|PRMT5|ENSG00000100462|beta-strand:combinatorial_evidence_used_in_manual_assertion|ENST00000553897|protein_coding|3/16|c.317T>C||||||,G|3_prime_UTR_variant|MODIFIER|PRMT5|ENSG00000100462|transcript|ENST00000553915|nonsense_mediated_decay|3/16|c.*69T>C|||||69|,G|3_prime_UTR_variant|MODIFIER|PRMT5|ENSG00000100462|transcript|ENST00000553787|nonsense_mediated_decay|2/6|c.*69T>C|||||69|,G|3_prime_UTR_variant|MODIFIER|PRMT5|ENSG00000100462|transcript|ENST00000557415|nonsense_mediated_decay|3/6|c.*69T>C|||||69|,G|upstream_gene_variant|MODIFIER|RP11-298I3.1|ENSG00000257285|transcript|ENST00000548322|antisense||n.-2118A>G|||||2118|,G|upstream_gene_variant|MODIFIER|PRMT5|ENSG00000100462|transcript|ENST00000476175|processed_transcript||n.-4706T>C|||||4706|,G|upstream_gene_variant|MODIFIER|PRMT5|ENSG00000100462|transcript|ENST00000555454|protein_coding||c.-3348T>C|||||3348|WARNING_TRANSCRIPT_NO_START_CODON,G|upstream_gene_variant|MODIFIER|PRMT5|ENSG00000100462|transcript|ENST00000454731|protein_coding||c.-4366T>C|||||4365|WARNING_TRANSCRIPT_NO_START_CODON,G|upstream_gene_variant|MODIFIER|PRMT5|ENSG00000100462|transcript|ENST00000557758|retained_intron||n.-4617T>C|||||4617|,G|upstream_gene_variant|MODIFIER|PRMT5|ENSG00000100462|transcript|ENST00000553502|protein_coding||c.-2481T>C|||||2481|WARNING_TRANSCRIPT_NO_START_CODON,G|upstream_gene_variant|MODIFIER|PRMT5|ENSG00000100462|transcript|ENST00000556043|protein_coding||c.-1362T>C|||||1071|WARNING_TRANSCRIPT_NO_STOP_CODON,G|upstream_gene_variant|MODIFIER|RP11-298I3.1|ENSG00000257285|transcript|ENST00000548819|antisense||n.-2082A>G|||||2082|,G|downstream_gene_variant|MODIFIER|PRMT5-AS1|ENSG00000237054|transcript|ENST00000424245|antisense||n.*4120A>G|||||4120|,G|downstream_gene_variant|MODIFIER|PRMT5-AS1|ENSG00000237054|transcript|ENST00000609885|antisense||n.*4276A>G|||||4276|,G|downstream_gene_variant|MODIFIER|PRMT5-AS1|ENSG00000237054|transcript|ENST00000457443|antisense||n.*4124A>G|||||4124|,G|downstream_gene_variant|MODIFIER|PRMT5-AS1|ENSG00000237054|transcript|ENST00000599580|antisense||n.*697A>G|||||697|,G|downstream_gene_variant|MODIFIER|PRMT5-AS1|ENSG00000237054|transcript|ENST00000595662|antisense||n.*908A>G|||||908|,G|downstream_gene_variant|MODIFIER|PRMT5-AS1|ENSG00000237054|transcript|ENST00000587245|antisense||n.*702A>G|||||702|,G|downstream_gene_variant|MODIFIER|PRMT5-AS1|ENSG00000237054|transcript|ENST00000590290|antisense||n.*631A>G|||||631|,G|downstream_gene_variant|MODIFIER|PRMT5|ENSG00000100462|transcript|ENST00000557015|retained_intron||n.*604T>C|||||604|,G|downstream_gene_variant|MODIFIER|PRMT5|ENSG00000100462|transcript|ENST00000556426|processed_transcript||n.*736T>C|||||736|,G|intron_variant|MODIFIER|PRMT5|ENSG00000100462|transcript|ENST00000397440|protein_coding|3/12|c.264+599T>C||||||,G|intron_variant|MODIFIER|PRMT5|ENSG00000100462|transcript|ENST00000553550|protein_coding|3/4|c.315+599T>C||||||WARNING_TRANSCRIPT_NO_STOP_CODON,G|intron_variant|MODIFIER|PRMT5|ENSG00000100462|transcript|ENST00000554716|processed_transcript|3/4|n.352+599T>C||||||,G|intron_variant|MODIFIER|PRMT5|ENSG00000100462|transcript|ENST00000554867|protein_coding|3/5|c.315+599T>C||||||WARNING_TRANSCRIPT_NO_STOP_CODON |
| RP11-298I3.1 | rs149734381 | 37 | 14 | 23397034 | 23397034 | + | SNP | G | G | A | SRR8586412 | Missense_Mutation | 0/1:84,137:221:99:4072,0,1920 | 222;ANN=A|upstream_gene_variant|MODIFIER|RP11-298I3.1|ENSG00000257285|transcript|ENST00000548322|antisense||n.-1820G>A|||||1820|,A|upstream_gene_variant|MODIFIER|PRMT5|ENSG00000100462|transcript|ENST00000555454|protein_coding||c.-3646C>T|||||3646|WARNING_TRANSCRIPT_NO_START_CODON,A|upstream_gene_variant|MODIFIER|PRMT5|ENSG00000100462|transcript|ENST00000454731|protein_coding||c.-4664C>T|||||4663|WARNING_TRANSCRIPT_NO_START_CODON,A|upstream_gene_variant|MODIFIER|PRMT5|ENSG00000100462|transcript|ENST00000557758|retained_intron||n.-4915C>T|||||4915|,A|upstream_gene_variant|MODIFIER|PRMT5|ENSG00000100462|transcript|ENST00000553502|protein_coding||c.-2779C>T|||||2779|WARNING_TRANSCRIPT_NO_START_CODON,A|upstream_gene_variant|MODIFIER|PRMT5|ENSG00000100462|transcript|ENST00000556043|protein_coding||c.-1660C>T|||||1369|WARNING_TRANSCRIPT_NO_STOP_CODON,A|upstream_gene_variant|MODIFIER|PRMT5|ENSG00000100462|transcript|ENST00000553417|retained_intron||n.-229C>T|||||229|,A|upstream_gene_variant|MODIFIER|RP11-298I3.1|ENSG00000257285|transcript|ENST00000548819|antisense||n.-1784G>A|||||1784|,A|downstream_gene_variant|MODIFIER|PRMT5-AS1|ENSG00000237054|transcript|ENST00000424245|antisense||n.*4418G>A|||||4418|,A|downstream_gene_variant|MODIFIER|PRMT5-AS1|ENSG00000237054|transcript|ENST00000609885|antisense||n.*4574G>A|||||4574|,A|downstream_gene_variant|MODIFIER|PRMT5-AS1|ENSG00000237054|transcript|ENST00000457443|antisense||n.*4422G>A|||||4422|,A|downstream_gene_variant|MODIFIER|PRMT5-AS1|ENSG00000237054|transcript|ENST00000599580|antisense||n.*995G>A|||||995|,A|downstream_gene_variant|MODIFIER|PRMT5-AS1|ENSG00000237054|transcript|ENST00000595662|antisense||n.*1206G>A|||||1206|,A|downstream_gene_variant|MODIFIER|PRMT5-AS1|ENSG00000237054|transcript|ENST00000587245|antisense||n.*1000G>A|||||1000|,A|downstream_gene_variant|MODIFIER|PRMT5-AS1|ENSG00000237054|transcript|ENST00000590290|antisense||n.*929G>A|||||929|,A|downstream_gene_variant|MODIFIER|PRMT5|ENSG00000100462|transcript|ENST00000557015|retained_intron||n.*306C>T|||||306|,A|downstream_gene_variant|MODIFIER|PRMT5|ENSG00000100462|transcript|ENST00000556426|processed_transcript||n.*438C>T|||||438|,A|intron_variant|MODIFIER|PRMT5|ENSG00000100462|transcript|ENST00000324366|protein_coding|3/16|c.316-165C>T||||||,A|intron_variant|MODIFIER|PRMT5|ENSG00000100462|transcript|ENST00000397441|protein_coding|3/16|c.265-165C>T||||||,A|intron_variant|MODIFIER|PRMT5|ENSG00000100462|transcript|ENST00000397440|protein_coding|3/12|c.264+301C>T||||||,A|intron_variant|MODIFIER|PRMT5|ENSG00000100462|transcript|ENST00000216350|protein_coding|2/15|c.179-211C>T||||||,A|intron_variant|MODIFIER|PRMT5|ENSG00000100462|transcript|ENST00000553915|nonsense_mediated_decay|2/15|c.146-165C>T||||||,A|intron_variant|MODIFIER|PRMT5|ENSG00000100462|transcript|ENST00000538452|protein_coding|2/15|c.-3-165C>T||||||,A|intron_variant|MODIFIER|PRMT5|ENSG00000100462|transcript|ENST00000553897|protein_coding|2/15|c.230-211C>T||||||,A|intron_variant|MODIFIER|PRMT5|ENSG00000100462|transcript|ENST00000555530|protein_coding|1/8|c.19-165C>T||||||WARNING_TRANSCRIPT_NO_START_CODON,A|intron_variant|MODIFIER|PRMT5|ENSG00000100462|transcript|ENST00000553550|protein_coding|3/4|c.315+301C>T||||||WARNING_TRANSCRIPT_NO_STOP_CODON,A|intron_variant|MODIFIER|PRMT5|ENSG00000100462|transcript|ENST00000554716|processed_transcript|3/4|n.352+301C>T||||||,A|intron_variant|MODIFIER|PRMT5|ENSG00000100462|transcript|ENST00000553787|nonsense_mediated_decay|1/5|c.111-211C>T||||||,A|intron_variant|MODIFIER|PRMT5|ENSG00000100462|transcript|ENST00000553641|processed_transcript|3/6|n.339-165C>T||||||,A|intron_variant|MODIFIER|PRMT5|ENSG00000100462|transcript|ENST00000554867|protein_coding|3/5|c.315+301C>T||||||WARNING_TRANSCRIPT_NO_STOP_CODON,A|intron_variant|MODIFIER|PRMT5|ENSG00000100462|transcript|ENST00000557415|nonsense_mediated_decay|2/5|c.197-165C>T||||||,A|intron_variant|MODIFIER|PRMT5|ENSG00000100462|transcript|ENST00000556616|protein_coding|2/5|c.202-165C>T||||||WARNING_TRANSCRIPT_INCOMPLETE,A|intron_variant|MODIFIER|PRMT5|ENSG00000100462|transcript|ENST00000554910|protein_coding|3/6|c.190-165C>T||||||WARNING_TRANSCRIPT_INCOMPLETE,A|intron_variant|MODIFIER|PRMT5|ENSG00000100462|transcript|ENST00000421938|protein_coding|3/4|c.346-165C>T||||||WARNING_TRANSCRIPT_NO_STOP_CODON,A|intron_variant|MODIFIER|PRMT5|ENSG00000100462|transcript|ENST00000556032|retained_intron|2/2|n.509-165C>T|||||| |
| GOLGA8A | rs111244139 | 37 | 15 | 34794833 | 34794833 | + | SNP | G | G | T | SRR8586412 | Missense_Mutation | 0/1:99,131:230:99:2147,0,7231 | 232;ANN=T|intron_variant|MODIFIER|GOLGA8A|ENSG00000175265|transcript|ENST00000543376|protein_coding|8/22|c.-1598+34712C>A|||||| |
| GOLGA8A | rs554219325 | 37 | 15 | 34794835 | 34794835 | + | SNP | A | A | G | SRR8586412 | Missense_Mutation | 0/1:96,72:168:99:2280,0,5588 | 168;ANN=G|intron_variant|MODIFIER|GOLGA8A|ENSG00000175265|transcript|ENST00000543376|protein_coding|8/22|c.-1598+34710T>C|||||| |
| RP11-160E2.16 | rs763617811 | 37 | 17 | 19112564 | 19112564 | + | INS | T | T | TA | SRR8586412 | Frame_Shift_Ins | 0/1:87,119:206:99:4122,0,2816 | 206;ANN=TA|downstream_gene_variant|MODIFIER|RP11-160E2.16|ENSG00000236022|transcript|ENST00000447506|lincRNA||n.*2440_*2441insA|||||2441|,TA|intron_variant|MODIFIER|RP11-160E2.16|ENSG00000236022|transcript|ENST00000428348|lincRNA|2/2|n.272+2950dupA||||||INFO_REALIGN_3_PRIME,TA|intron_variant|MODIFIER|RP11-160E2.16|ENSG00000236022|transcript|ENST00000424109|lincRNA|2/2|n.264+2950dupA||||||INFO_REALIGN_3_PRIME,TA|non_coding_transcript_exon_variant|MODIFIER|KYNUP3|ENSG00000263206|transcript|ENST00000574386|transcribed_unprocessed_pseudogene|2/5|n.346dupA||||||INFO_REALIGN_3_PRIME |
| CTIF | rs139399471 | 37 | 18 | 46084650 | 46084650 | + | SNP | G | G | A | SRR8586412 | Missense_Mutation | 0/1:47,97:144:99:3114,0,1367 | 148;ANN=A|intron_variant|MODIFIER|CTIF|ENSG00000134030|transcript|ENST00000382998|protein_coding|2/12|c.-29+18448G>A||||||,A|intron_variant|MODIFIER|CTIF|ENSG00000134030|transcript|ENST00000256413|protein_coding|1/11|c.-29+18967G>A||||||,A|intron_variant|MODIFIER|CTIF|ENSG00000134030|transcript|ENST00000591412|protein_coding|1/4|c.-29+18967G>A||||||WARNING_TRANSCRIPT_NO_STOP_CODON,A|intron_variant|MODIFIER|CTIF|ENSG00000134030|transcript|ENST00000591387|protein_coding|1/4|c.-29+18448G>A||||||WARNING_TRANSCRIPT_NO_STOP_CODON,A|intron_variant|MODIFIER|CTIF|ENSG00000134030|transcript|ENST00000587752|protein_coding|1/7|c.-29+18221G>A||||||WARNING_TRANSCRIPT_INCOMPLETE,A|intron_variant|MODIFIER|CTIF|ENSG00000134030|transcript|ENST00000588345|protein_coding|1/3|c.-29+17942G>A||||||WARNING_TRANSCRIPT_INCOMPLETE |
| LILRB1 | rs111247067 | 37 | 19 | 55146037 | 55146037 | + | SNP | C | C | G | SRR8586412 | Splice_Site | 0/1:32,22:54:99:563,0,811 | 54;ANN=G|splice_acceptor_variant&intron_variant|HIGH|LILRB1|ENSG00000104972|transcript|ENST00000462628|processed_transcript|3/7|n.205-2C>G||||||,G|upstream_gene_variant|MODIFIER|LILRB1|ENSG00000104972|transcript|ENST00000480375|retained_intron||n.-307C>G|||||307|,G|downstream_gene_variant|MODIFIER|AC009892.10|ENSG00000224730|transcript|ENST00000456337|protein_coding||c.*1335G>C|||||1136|,G|downstream_gene_variant|MODIFIER|LILRB1|ENSG00000104972|transcript|ENST00000480257|processed_transcript||n.*2531C>G|||||2531|,G|downstream_gene_variant|MODIFIER|LILRB1|ENSG00000104972|transcript|ENST00000473412|processed_transcript||n.*403C>G|||||403|,G|intron_variant|MODIFIER|LILRB1|ENSG00000104972|transcript|ENST00000427581|protein_coding|9/14|c.1511-55C>G||||||,G|intron_variant|MODIFIER|LILRB1|ENSG00000104972|transcript|ENST00000396321|protein_coding|8/13|c.1361-55C>G||||||,G|intron_variant|MODIFIER|LILRB1|ENSG00000104972|transcript|ENST00000418536|protein_coding|7/12|c.1313-55C>G||||||,G|intron_variant|MODIFIER|LILRB1|ENSG00000104972|transcript|ENST00000448689|protein_coding|8/12|c.1361-55C>G||||||,G|intron_variant|MODIFIER|LILRB1|ENSG00000104972|transcript|ENST00000396331|protein_coding|10/15|c.1361-55C>G||||||,G|intron_variant|MODIFIER|LILRB1|ENSG00000104972|transcript|ENST00000396327|protein_coding|9/14|c.1364-55C>G||||||,G|intron_variant|MODIFIER|LILRB1|ENSG00000104972|transcript|ENST00000324602|protein_coding|9/14|c.1364-55C>G||||||,G|intron_variant|MODIFIER|LILRB1|ENSG00000104972|transcript|ENST00000434867|protein_coding|9/14|c.1361-55C>G||||||,G|intron_variant|MODIFIER|LILRB1|ENSG00000104972|transcript|ENST00000396332|protein_coding|9/14|c.1361-55C>G||||||,G|intron_variant|MODIFIER|LILRB1|ENSG00000104972|transcript|ENST00000396315|protein_coding|8/13|c.1364-55C>G||||||,G|intron_variant|MODIFIER|LILRB1|ENSG00000104972|transcript|ENST00000396317|protein_coding|7/12|c.1313-55C>G||||||,G|intron_variant|MODIFIER|LILRB1|ENSG00000104972|transcript|ENST00000421584|nonsense_mediated_decay|8/12|c.1361-55C>G||||||,G|non_coding_transcript_exon_variant|MODIFIER|LILRB1|ENSG00000104972|transcript|ENST00000487425|retained_intron|1/4|n.216C>G||||||WARNING_REF_DOES_NOT_MATCH_GENOME |
| RAB10 | rs530599892 | 37 | 2 | 26285566 | 26285566 | + | SNP | G | G | A | SRR8586412 | Missense_Mutation | 0/1:146,47:193:99:1275,0,3841 | 204;ANN=A|intron_variant|MODIFIER|RAB10|ENSG00000084733|transcript|ENST00000264710|protein_coding|1/5|c.127+27962G>A||||||,A|intron_variant|MODIFIER|RAB10|ENSG00000084733|transcript|ENST00000495146|retained_intron|1/4|n.629+27962G>A|||||| |
| ALK-AC016907.3 | rs13389106 | 37 | 2 | 30257884 | 30257884 | + | SNP | T | T | G | SRR8586412 | Missense_Mutation | 0/1:51,26:77:99:958,0,2076 | 78;ANN=G|intergenic_region|MODIFIER|ALK-AC016907.3|ENSG00000171094-ENSG00000233862|intergenic_region|ENSG00000171094-ENSG00000233862|||n.30257884T>G|||||| |
| ALK-AC016907.3 | rs13386353 | 37 | 2 | 30257890 | 30257890 | + | SNP | A | A | G | SRR8586412 | Missense_Mutation | 0/1:56,29:85:99:993,0,2193 | 87;ANN=G|intergenic_region|MODIFIER|ALK-AC016907.3|ENSG00000171094-ENSG00000233862|intergenic_region|ENSG00000171094-ENSG00000233862|||n.30257890A>G|||||| |
| SLC8A1-AS1 | rs66532383 | 37 | 2 | 40219806 | 40219806 | + | DEL | CTTTGTTTGCTATGCATGAGATTGCTTGAAAAATGTGATTTAGCTCCAACAGAGCCTCCCTACTATAGGATTTTTTTTTTTTTTTTTTTTTTTTTTTTAGAAAATCAATACATCTTTCTGTCAAAATT | CTTTGTTTGCTATGCATGAGATTGCTTGAAAAATGTGATTTAGCTCCAACAGAGCCTCCCTACTATAGGATTTTTTTTTTTTTTTTTTTTTTTTTTTTAGAAAATCAATACATCTTTCTGTCAAAATT | C | SRR8586412 | In_Frame_Del | 0/1:20,38:58:99:1447,0,714 | 58;ANN=C|intron_variant|MODIFIER|SLC8A1-AS1|ENSG00000227028|transcript|ENST00000599740|antisense|1/1|n.73+206142_73+206268del||||||,C|intron_variant|MODIFIER|SLC8A1-AS1|ENSG00000227028|transcript|ENST00000418854|antisense|2/2|n.132+69778_132+69904del|||||| |
| PDE11A | rs149494189 | 37 | 2 | 178654576 | 178654576 | + | SNP | T | T | C | SRR8586412 | Missense_Mutation | 0/1:18,165:183:85:4725,0,85 | 183;ANN=C|intron_variant|MODIFIER|PDE11A|ENSG00000128655|transcript|ENST00000286063|protein_coding|9/19|c.1738-20475A>G||||||,C|intron_variant|MODIFIER|PDE11A|ENSG00000128655|transcript|ENST00000358450|protein_coding|10/20|c.988-20475A>G||||||,C|intron_variant|MODIFIER|PDE11A|ENSG00000128655|transcript|ENST00000409504|protein_coding|8/19|c.664-20475A>G||||||,C|intron_variant|MODIFIER|PDE11A|ENSG00000128655|transcript|ENST00000389683|protein_coding|6/16|c.406-20475A>G||||||,C|intron_variant|MODIFIER|PDE11A|ENSG00000128655|transcript|ENST00000449286|protein_coding|8/18|c.664-20475A>G||||||,C|intron_variant|MODIFIER|PDE11A|ENSG00000128655|transcript|ENST00000433879|protein_coding|6/13|c.559-20475A>G||||||WARNING_TRANSCRIPT_NO_START_CODON,C|intron_variant|MODIFIER|PDE11A|ENSG00000128655|transcript|ENST00000497003|processed_transcript|8/14|n.780-20475A>G|||||| |
| PDE11A | rs148207286 | 37 | 2 | 178654624 | 178654624 | + | SNP | T | T | C | SRR8586412 | Missense_Mutation | 0/1:78,30:108:99:937,0,3218 | 108;ANN=C|intron_variant|MODIFIER|PDE11A|ENSG00000128655|transcript|ENST00000286063|protein_coding|9/19|c.1738-20523A>G||||||,C|intron_variant|MODIFIER|PDE11A|ENSG00000128655|transcript|ENST00000358450|protein_coding|10/20|c.988-20523A>G||||||,C|intron_variant|MODIFIER|PDE11A|ENSG00000128655|transcript|ENST00000409504|protein_coding|8/19|c.664-20523A>G||||||,C|intron_variant|MODIFIER|PDE11A|ENSG00000128655|transcript|ENST00000389683|protein_coding|6/16|c.406-20523A>G||||||,C|intron_variant|MODIFIER|PDE11A|ENSG00000128655|transcript|ENST00000449286|protein_coding|8/18|c.664-20523A>G||||||,C|intron_variant|MODIFIER|PDE11A|ENSG00000128655|transcript|ENST00000433879|protein_coding|6/13|c.559-20523A>G||||||WARNING_TRANSCRIPT_NO_START_CODON,C|intron_variant|MODIFIER|PDE11A|ENSG00000128655|transcript|ENST00000497003|processed_transcript|8/14|n.780-20523A>G|||||| |
| PDE11A | rs58496397 | 37 | 2 | 178658087 | 178658087 | + | SNP | C | C | T | SRR8586412 | Nonsense_Mutation | 0/1:316,44:360:99:568,0,8089 | 360;ANN=T|downstream_gene_variant|MODIFIER|PDE11A|ENSG00000128655|transcript|ENST00000492761|processed_transcript||n.*3712G>A|||||3712|,T|intron_variant|MODIFIER|PDE11A|ENSG00000128655|transcript|ENST00000286063|protein_coding|9/19|c.1737+23469G>A||||||,T|intron_variant|MODIFIER|PDE11A|ENSG00000128655|transcript|ENST00000358450|protein_coding|10/20|c.987+23469G>A||||||,T|intron_variant|MODIFIER|PDE11A|ENSG00000128655|transcript|ENST00000409504|protein_coding|8/19|c.663+23469G>A||||||,T|intron_variant|MODIFIER|PDE11A|ENSG00000128655|transcript|ENST00000389683|protein_coding|6/16|c.405+23469G>A||||||,T|intron_variant|MODIFIER|PDE11A|ENSG00000128655|transcript|ENST00000449286|protein_coding|8/18|c.663+23469G>A||||||,T|intron_variant|MODIFIER|PDE11A|ENSG00000128655|transcript|ENST00000433879|protein_coding|6/13|c.559-23986G>A||||||WARNING_TRANSCRIPT_NO_START_CODON,T|intron_variant|MODIFIER|PDE11A|ENSG00000128655|transcript|ENST00000497003|processed_transcript|8/14|n.779+23469G>A|||||| |
| TBC1D5 | rs371256943 | 37 | 3 | 17860355 | 17860355 | + | SNP | C | C | T | SRR8586412 | Missense_Mutation | 0/1:9,15:24:99:476,0,333 | 24;ANN=T|intron_variant|MODIFIER|TBC1D5|ENSG00000131374|transcript|ENST00000414318|processed_transcript|1/8|n.212-510729G>A|||||| |
| NFKBIZ | rs771150914 | 37 | 3 | 101576663 | 101576663 | + | SNP | A | A | G | SRR8586412 | Nonsense_Mutation | 0/1:20,70:90:99:2072,0,483 | 91;ANN=G|downstream_gene_variant|MODIFIER|NFKBIZ|ENSG00000144802|transcript|ENST00000483180|protein_coding||c.*360A>G|||||360|WARNING_TRANSCRIPT_NO_STOP_CODON,G|downstream_gene_variant|MODIFIER|NFKBIZ|ENSG00000144802|transcript|ENST00000491281|protein_coding||c.*4604A>G|||||4604|WARNING_TRANSCRIPT_INCOMPLETE,G|downstream_gene_variant|MODIFIER|NFKBIZ|ENSG00000144802|transcript|ENST00000465476|retained_intron||n.*1802A>G|||||1802|,G|intron_variant|MODIFIER|NFKBIZ|ENSG00000144802|transcript|ENST00000326172|protein_coding|11/11|c.2103+360A>G||||||,G|intron_variant|MODIFIER|NFKBIZ|ENSG00000144802|transcript|ENST00000394054|protein_coding|12/12|c.1803+360A>G||||||,G|intron_variant|MODIFIER|NFKBIZ|ENSG00000144802|transcript|ENST00000326151|protein_coding|12/12|c.1737+360A>G||||||,G|intron_variant|MODIFIER|NFKBIZ|ENSG00000144802|transcript|ENST00000477601|protein_coding|2/2|c.336+360A>G||||||,G|intron_variant|MODIFIER|NFKBIZ|ENSG00000144802|transcript|ENST00000495089|retained_intron|2/2|n.671+360A>G||||||,G|intron_variant|MODIFIER|NFKBIZ|ENSG00000144802|transcript|ENST00000495719|retained_intron|1/1|n.260+360A>G|||||| |
| AC138951.1 | rs371964320 | 37 | 5 | 21491337 | 21491337 | + | SNP | G | G | T | SRR8586412 | Missense_Mutation | 0/1:23,22:45:99:663,0,535 | 50;ANN=T|upstream_gene_variant|MODIFIER|AC138951.1|ENSG00000203563|transcript|ENST00000366374|pseudogene||n.-2355G>T|||||2355|,T|intron_variant|MODIFIER|GUSBP1|ENSG00000183666|transcript|ENST00000607545|processed_transcript|3/4|n.180-10464G>T||||||,T|intron_variant|MODIFIER|GUSBP1|ENSG00000183666|transcript|ENST00000508260|processed_transcript|4/8|n.371-93G>T||||||,T|intron_variant|MODIFIER|GUSBP1|ENSG00000183666|transcript|ENST00000328346|transcribed_unprocessed_pseudogene|3/5|n.256-93G>T||||||,T|intron_variant|MODIFIER|GUSBP1|ENSG00000183666|transcript|ENST00000449061|transcribed_unprocessed_pseudogene|3/6|n.176-93G>T|||||| |
| XXbac-BPG248L24.13 | rs1071650 | 37 | 6 | 31271844 | 31271844 | + | SNP | T | T | A | SRR8586412 | Missense_Mutation | 0/1:34,6:40:99:144,0,2734 | 40;ANN=A|upstream_gene_variant|MODIFIER|XXbac-BPG248L24.13|ENSG00000256166|transcript|ENST00000539514|lincRNA||n.-2425A>T|||||2425|,A|downstream_gene_variant|MODIFIER|XXbac-BPG248L24.10|ENSG00000229836|transcript|ENST00000421191|unprocessed_pseudogene||n.*3748A>T|||||3748|,A|intergenic_region|MODIFIER|XXbac-BPG248L24.13-XXbac-BPG248L24.10|ENSG00000256166-ENSG00000229836|intergenic_region|ENSG00000256166-ENSG00000229836|||n.31271844T>A|||||| |
| XXbac-BPG248L24.13 | rs9264668 | 37 | 6 | 31271845 | 31271845 | + | SNP | C | C | A | SRR8586412 | Missense_Mutation | 0/1:33,6:39:99:144,0,2734 | 39;ANN=A|upstream_gene_variant|MODIFIER|XXbac-BPG248L24.13|ENSG00000256166|transcript|ENST00000539514|lincRNA||n.-2426G>T|||||2426|,A|downstream_gene_variant|MODIFIER|XXbac-BPG248L24.10|ENSG00000229836|transcript|ENST00000421191|unprocessed_pseudogene||n.*3747G>T|||||3747|,A|intergenic_region|MODIFIER|XXbac-BPG248L24.13-XXbac-BPG248L24.10|ENSG00000256166-ENSG00000229836|intergenic_region|ENSG00000256166-ENSG00000229836|||n.31271845C>A|||||| |
| AL391417.1-RP1-253B10.1 | rs398140 | 37 | 6 | 87341544 | 87341544 | + | SNP | T | T | C | SRR8586412 | Missense_Mutation | 0/1:10,13:23:99:350,0,303 | 24;ANN=C|intergenic_region|MODIFIER|AL391417.1-RP1-253B10.1|ENSG00000221101-ENSG00000218561|intergenic_region|ENSG00000221101-ENSG00000218561|||n.87341544T>C|||||| |
| MYOM2 | rs10108246 | 37 | 8 | 2060291 | 2060291 | + | SNP | A | A | G | SRR8586412 | Missense_Mutation | 0/1:89,31:120:99:667,0,2675 | 120;ANN=G|intron_variant|MODIFIER|MYOM2|ENSG00000036448|transcript|ENST00000262113|protein_coding|25/36|c.3180+2969A>G||||||,G|intron_variant|MODIFIER|MYOM2|ENSG00000036448|transcript|ENST00000523438|protein_coding|12/23|c.1455+2969A>G||||||,G|intron_variant|MODIFIER|MYOM2|ENSG00000036448|transcript|ENST00000523443|retained_intron|1/7|n.245+2969A>G|||||| |
| MYOM2 | rs968381 | 37 | 8 | 2101041 | 2101041 | + | SNP | G | G | A | SRR8586412 | Missense_Mutation | 0/1:119,30:149:99:617,0,3135 | 186;ANN=A|intron_variant|MODIFIER|MYOM2|ENSG00000036448|transcript|ENST00000520779|processed_transcript|4/4|n.154+9661G>A|||||| |
| CTD-2547L16.2 | rs35838806 | 37 | 8 | 18056822 | 18056822 | + | DEL | ACTTC | ACTTC | A | SRR8586412 | In_Frame_Del | 0/1:551,49:600:99:387,0,24470 | 609;ANN=A|downstream_gene_variant|MODIFIER|CTD-2547L16.2|ENSG00000254015|transcript|ENST00000518144|processed_pseudogene||n.*1607_*1610delGAAG|||||1610|,A|intron_variant|MODIFIER|NAT1|ENSG00000171428|transcript|ENST00000517441|processed_transcript|2/4|n.93-10467_93-10464delCTTC||||||,A|intron_variant|MODIFIER|NAT1|ENSG00000171428|transcript|ENST00000535084|protein_coding|1/3|c.-260-10467_-260-10464delCTTC|||||| |
| CTD-2547L16.2 | rs574114 | 37 | 8 | 18057350 | 18057350 | + | SNP | A | A | T | SRR8586412 | Nonsense_Mutation | 0/1:382,79:461:99:2143,0,15790 | 473;ANN=T|downstream_gene_variant|MODIFIER|CTD-2547L16.2|ENSG00000254015|transcript|ENST00000518144|processed_pseudogene||n.*1083T>A|||||1083|,T|intron_variant|MODIFIER|NAT1|ENSG00000171428|transcript|ENST00000517441|processed_transcript|2/4|n.93-9940A>T||||||,T|intron_variant|MODIFIER|NAT1|ENSG00000171428|transcript|ENST00000535084|protein_coding|1/3|c.-260-9940A>T|||||| |
| CTD-2547L16.2 | rs417661 | 37 | 8 | 18057410 | 18057410 | + | SNP | A | A | T | SRR8586412 | Nonsense_Mutation | 0/1:386,174:560:99:4474,0,10865 | 571;ANN=T|downstream_gene_variant|MODIFIER|CTD-2547L16.2|ENSG00000254015|transcript|ENST00000518144|processed_pseudogene||n.*1023T>A|||||1023|,T|intron_variant|MODIFIER|NAT1|ENSG00000171428|transcript|ENST00000517441|processed_transcript|2/4|n.93-9880A>T||||||,T|intron_variant|MODIFIER|NAT1|ENSG00000171428|transcript|ENST00000535084|protein_coding|1/3|c.-260-9880A>T|||||| |
| NOTCH2 | rs1699761 | 37 | 1 | 120489416 | 120489416 | + | SNP | C | C | A | SRR8586413 | Nonsense_Mutation | 0/1:118,41:159:99:855,0,3054 | 159;ANN=A|downstream_gene_variant|MODIFIER|NOTCH2|ENSG00000134250|transcript|ENST00000479412|retained_intron||n.*3327G>T|||||3327|,A|intron_variant|MODIFIER|NOTCH2|ENSG00000134250|transcript|ENST00000256646|protein_coding|17/33|c.2752+1621G>T|||||| |
| RP11-763B22.9 | rs1664022 | 37 | 1 | 148889827 | 148889827 | + | SNP | G | G | T | SRR8586413 | Missense_Mutation | 0/1:155,24:179:99:298,0,4029 | 181;ANN=T|intron_variant|MODIFIER|RP11-763B22.9|ENSG00000231448|transcript|ENST00000444424|unprocessed_pseudogene|8/9|n.1017+138G>T|||||| |
| RP11-14N7.2 | rs3124680 | 37 | 1 | 148933084 | 148933084 | + | SNP | T | T | A | SRR8586413 | Nonsense_Mutation | 0/1:25,36:61:99:1089,0,619 | 61;ANN=A|downstream_gene_variant|MODIFIER|RP11-14N7.2|ENSG00000232527|transcript|ENST00000420597|lincRNA||n.*164T>A|||||164|,A|intron_variant|MODIFIER|RP11-14N7.2|ENSG00000232527|transcript|ENST00000539543|lincRNA|2/3|n.176+164T>A||||||,A|intron_variant|MODIFIER|RP11-14N7.2|ENSG00000232527|transcript|ENST00000452399|lincRNA|2/2|n.199+164T>A||||||,A|intron_variant|MODIFIER|RP11-14N7.2|ENSG00000232527|transcript|ENST00000294715|lincRNA|2/2|n.190+164T>A||||||,A|intron_variant|MODIFIER|RP11-14N7.2|ENSG00000232527|transcript|ENST00000457390|lincRNA|1/1|n.136+164T>A|||||| |
| RP11-14N7.2 | rs512570 | 37 | 1 | 148933778 | 148933778 | + | SNP | A | A | G | SRR8586413 | Nonsense_Mutation | 0/1:32,29:61:99:794,0,826 | 61;ANN=G|downstream_gene_variant|MODIFIER|RP11-14N7.2|ENSG00000232527|transcript|ENST00000420597|lincRNA||n.*858A>G|||||858|,G|downstream_gene_variant|MODIFIER|RP11-14N7.2|ENSG00000232527|transcript|ENST00000294715|lincRNA||n.*410A>G|||||410|,G|downstream_gene_variant|MODIFIER|RP11-14N7.2|ENSG00000232527|transcript|ENST00000457390|lincRNA||n.*199A>G|||||199|,G|intron_variant|MODIFIER|RP11-14N7.2|ENSG00000232527|transcript|ENST00000539543|lincRNA|3/3|n.254+410A>G||||||,G|intron_variant|MODIFIER|RP11-14N7.2|ENSG00000232527|transcript|ENST00000452399|lincRNA|2/2|n.199+858A>G|||||| |
| RNVU1-17 | rs61810934 | 37 | 1 | 149196907 | 149196907 | + | SNP | G | G | C | SRR8586413 | Missense_Mutation | 0/1:263,137:400:99:3330,0,7182 | 400;ANN=C|upstream_gene_variant|MODIFIER|RNVU1-17|ENSG00000207349|transcript|ENST00000384619|snRNA||n.-2638C>G|||||2638|,C|intergenic_region|MODIFIER|RNVU1-17-RNU1-92P|ENSG00000207349-ENSG00000252826|intergenic_region|ENSG00000207349-ENSG00000252826|||n.149196907G>C|||||| |
| RNVU1-17 | rs61810935 | 37 | 1 | 149196923 | 149196923 | + | SNP | C | C | A | SRR8586413 | Missense_Mutation | 0/1:258,103:361:99:2247,0,7186 | 361;ANN=A|upstream_gene_variant|MODIFIER|RNVU1-17|ENSG00000207349|transcript|ENST00000384619|snRNA||n.-2654G>T|||||2654|,A|intergenic_region|MODIFIER|RNVU1-17-RNU1-92P|ENSG00000207349-ENSG00000252826|intergenic_region|ENSG00000207349-ENSG00000252826|||n.149196923C>A|||||| |
| RNVU1-17 | rs75500033 | 37 | 1 | 149197768 | 149197768 | + | SNP | C | C | T | SRR8586413 | Missense_Mutation | 0/1:511,95:606:99:1406,0,14088 | 606;ANN=T|upstream_gene_variant|MODIFIER|RNVU1-17|ENSG00000207349|transcript|ENST00000384619|snRNA||n.-3499G>A|||||3499|,T|intergenic_region|MODIFIER|RNVU1-17-RNU1-92P|ENSG00000207349-ENSG00000252826|intergenic_region|ENSG00000207349-ENSG00000252826|||n.149197768C>T|||||| |
| MTR-RPL35P1 | rs6686489 | 37 | 1 | 237091429 | 237091429 | + | SNP | G | G | T | SRR8586413 | Missense_Mutation | 0/1:49,112:161:99:2452,0,1050 | 209;ANN=T|intergenic_region|MODIFIER|MTR-RPL35P1|ENSG00000116984-ENSG00000237991|intergenic_region|ENSG00000116984-ENSG00000237991|||n.237091429G>T|||||| |
| MTR-RPL35P1 | rs6686490 | 37 | 1 | 237091430 | 237091430 | + | SNP | G | G | T | SRR8586413 | Missense_Mutation | 0/1:43,116:159:99:2608,0,940 | 206;ANN=T|intergenic_region|MODIFIER|MTR-RPL35P1|ENSG00000116984-ENSG00000237991|intergenic_region|ENSG00000116984-ENSG00000237991|||n.237091430G>T|||||| |
| MTR-RPL35P1 | rs368474001 | 37 | 1 | 237091437 | 237091437 | + | SNP | C | C | G | SRR8586413 | Missense_Mutation | 0/1:122,35:157:99:190,0,3288 | 157;ANN=G|intergenic_region|MODIFIER|MTR-RPL35P1|ENSG00000116984-ENSG00000237991|intergenic_region|ENSG00000116984-ENSG00000237991|||n.237091437C>G|||||| |
| RYR2 | rs11330863 | 37 | 1 | 237367587 | 237367587 | + | DEL | TA | TA | T | SRR8586413 | Frame_Shift_Del | 0/1:147,46:193:99:1463,0,6034 | 196;ANN=T|intron_variant|MODIFIER|RYR2|ENSG00000198626|transcript|ENST00000366574|protein_coding|1/104|c.49-66209delA|||||| |
| RYR2 | rs34151790 | 37 | 1 | 237367594 | 237367594 | + | SNP | C | C | T | SRR8586413 | Missense_Mutation | 0/1:149,45:194:99:1466,0,6061 | 194;ANN=T|intron_variant|MODIFIER|RYR2|ENSG00000198626|transcript|ENST00000366574|protein_coding|1/104|c.49-66203C>T|||||| |
| RYR2 | rs28725322 | 37 | 1 | 237623387 | 237623387 | + | SNP | T | T | C | SRR8586413 | Missense_Mutation | 0/1:14,4:18:99:126,0,831 | 18;ANN=C|intron_variant|MODIFIER|RYR2|ENSG00000198626|transcript|ENST00000360064|protein_coding|17/106|c.1606+3352T>C||||||,C|intron_variant|MODIFIER|RYR2|ENSG00000198626|transcript|ENST00000366574|protein_coding|16/104|c.1612+3352T>C||||||,C|intron_variant|MODIFIER|RYR2|ENSG00000198626|transcript|ENST00000542537|protein_coding|15/103|c.1564+3352T>C||||||WARNING_TRANSCRIPT_NO_START_CODON |
| RYR2 | rs2794826 | 37 | 1 | 237804191 | 237804191 | + | SNP | G | G | A | SRR8586413 | Splice_Site | 0/1:35,55:90:99:1263,0,856 | 90;ANN=A|splice_region_variant&intron_variant|LOW|RYR2|ENSG00000198626|transcript|ENST00000360064|protein_coding|47/106|c.7110-6G>A||||||,A|splice_region_variant&intron_variant|LOW|RYR2|ENSG00000198626|transcript|ENST00000366574|protein_coding|46/104|c.7116-6G>A||||||,A|splice_region_variant&intron_variant|LOW|RYR2|ENSG00000198626|transcript|ENST00000542537|protein_coding|45/103|c.7068-6G>A||||||WARNING_TRANSCRIPT_NO_START_CODON |
| H2AFY2 | rs7869 | 37 | 10 | 71816550 | 71816550 | + | SNP | C | T | T | SRR8586413 | Missense_Mutation | 1/1:18,298:316:99:9845,279,0 | 319;ANN=T|intron_variant|MODIFIER|H2AFY2|ENSG00000099284|transcript|ENST00000373255|protein_coding|1/8|c.-60+3794C>T||||||,T|intron_variant|MODIFIER|H2AFY2|ENSG00000099284|transcript|ENST00000455786|protein_coding|1/6|c.-60+3794C>T||||||WARNING_TRANSCRIPT_INCOMPLETE |
| AC091487.1-RP11-181F12.1 | rs76249835 | 37 | 10 | 86786935 | 86786935 | + | SNP | G | G | A | SRR8586413 | Missense_Mutation | 0/1:9,8:17:99:239,0,275 | 17;ANN=A|intergenic_region|MODIFIER|AC091487.1-RP11-181F12.1|ENSG00000238469-ENSG00000237267|intergenic_region|ENSG00000238469-ENSG00000237267|||n.86786935G>A|||||| |
| PDE6C | rs11188311 | 37 | 10 | 95394941 | 95394941 | + | SNP | G | G | A | SRR8586413 | Missense_Mutation | 0/1:76,40:116:99:1451,0,3509 | 116;ANN=A|intron_variant|MODIFIER|PDE6C|ENSG00000095464|transcript|ENST00000371447|protein_coding|9/21|c.1269+277G>A|||||| |
| ZBTB3 | rs144102956 | 37 | 11 | 62520491 | 62520491 | + | SNP | G | G | A | SRR8586413 | Missense_Mutation | 0/1:881,112:993:99:1997,0,37412 | 993;ANN=A|missense_variant|MODERATE|ZBTB3|ENSG00000185670|transcript|ENST00000394807|protein_coding|2/2|c.796C>T|p.Asp266Tyr|922/2978|796/1725|266/574||WARNING_REF_DOES_NOT_MATCH_GENOME,A|upstream_gene_variant|MODIFIER|ZBTB3|ENSG00000185670|transcript|ENST00000530112|nonsense_mediated_decay||c.-921C>T|||||920|WARNING_TRANSCRIPT_NO_START_CODON,A|downstream_gene_variant|MODIFIER|ZBTB3|ENSG00000185670|transcript|ENST00000527994|protein_coding||c.*299C>T|||||299|WARNING_TRANSCRIPT_INCOMPLETE |
| TSPAN9 | rs58837502 | 37 | 12 | 3211762 | 3211762 | + | SNP | T | T | C | SRR8586413 | Missense_Mutation | 0/1:37,76:113:99:2204,0,775 | 113;ANN=C|intron_variant|MODIFIER|TSPAN9|ENSG00000011105|transcript|ENST00000537971|protein_coding|1/7|c.-18+25143T>C||||||,C|intron_variant|MODIFIER|TSPAN9|ENSG00000011105|transcript|ENST00000444315|nonsense_mediated_decay|2/5|c.-18+18877T>C||||||,C|intron_variant|MODIFIER|TSPAN9|ENSG00000011105|transcript|ENST00000011898|protein_coding|2/8|c.-18+18877T>C|||||| |
| RP11-632B21.1-RP11-20L19.1 | rs80228231 | 37 | 12 | 91103708 | 91103708 | + | SNP | A | A | G | SRR8586413 | Missense_Mutation | 0/1:35,28:63:99:789,0,1058 | 63;ANN=G|intergenic_region|MODIFIER|RP11-632B21.1-RP11-20L19.1|ENSG00000257995-ENSG00000257787|intergenic_region|ENSG00000257995-ENSG00000257787|||n.91103708A>G|||||| |
| PRMT5-AS1 | rs200260229 | 37 | 14 | 23384530 | 23384530 | + | SNP | C | C | T | SRR8586413 | Missense_Mutation | 0/1:74,289:363:99:12071,0,2337 | 363;ANN=T|upstream_gene_variant|MODIFIER|PRMT5-AS1|ENSG00000237054|transcript|ENST00000424245|antisense||n.-4135C>T|||||4135|,T|upstream_gene_variant|MODIFIER|RBM23|ENSG00000100461|transcript|ENST00000555691|protein_coding||c.-3928G>A|||||3529|WARNING_TRANSCRIPT_INCOMPLETE,T|upstream_gene_variant|MODIFIER|PRMT5-AS1|ENSG00000237054|transcript|ENST00000609885|antisense||n.-4300C>T|||||4300|,T|downstream_gene_variant|MODIFIER|RBM23|ENSG00000100461|transcript|ENST00000553777|processed_transcript||n.*2243G>A|||||2243|,T|intron_variant|MODIFIER|RBM23|ENSG00000100461|transcript|ENST00000359890|protein_coding|1/13|c.-11+3678G>A||||||,T|intron_variant|MODIFIER|RBM23|ENSG00000100461|transcript|ENST00000555209|protein_coding|1/10|c.-493+3678G>A||||||,T|intron_variant|MODIFIER|RBM23|ENSG00000100461|transcript|ENST00000557667|retained_intron|1/13|n.138+3678G>A||||||,T|intron_variant|MODIFIER|RBM23|ENSG00000100461|transcript|ENST00000399922|protein_coding|1/12|c.-11+3678G>A||||||,T|intron_variant|MODIFIER|RBM23|ENSG00000100461|transcript|ENST00000346528|protein_coding|1/11|c.-11+3678G>A||||||,T|intron_variant|MODIFIER|RBM23|ENSG00000100461|transcript|ENST00000542016|protein_coding|1/11|c.-360+3678G>A||||||,T|intron_variant|MODIFIER|RBM23|ENSG00000100461|transcript|ENST00000307814|retained_intron|1/10|n.151+3678G>A||||||,T|intron_variant|MODIFIER|RBM23|ENSG00000100461|transcript|ENST00000557403|protein_coding|1/5|c.-343+3678G>A||||||WARNING_TRANSCRIPT_INCOMPLETE,T|intron_variant|MODIFIER|RBM23|ENSG00000100461|transcript|ENST00000555722|protein_coding|1/4|c.-284+3678G>A||||||WARNING_TRANSCRIPT_INCOMPLETE,T|intron_variant|MODIFIER|RBM23|ENSG00000100461|transcript|ENST00000553920|processed_transcript|1/4|n.138+3678G>A||||||,T|intron_variant|MODIFIER|RBM23|ENSG00000100461|transcript|ENST00000557227|protein_coding|1/3|c.-306+3678G>A||||||WARNING_TRANSCRIPT_NO_STOP_CODON,T|intron_variant|MODIFIER|RBM23|ENSG00000100461|transcript|ENST00000555714|processed_transcript|1/5|n.151+3678G>A||||||,T|intron_variant|MODIFIER|RBM23|ENSG00000100461|transcript|ENST00000554256|protein_coding|1/5|c.-11+3678G>A||||||WARNING_TRANSCRIPT_NO_STOP_CODON,T|intron_variant|MODIFIER|RBM23|ENSG00000100461|transcript|ENST00000556687|retained_intron|1/3|n.151+3678G>A||||||,T|intron_variant|MODIFIER|RBM23|ENSG00000100461|transcript|ENST00000553902|retained_intron|1/2|n.151+3678G>A||||||,T|intron_variant|MODIFIER|RBM23|ENSG00000100461|transcript|ENST00000554955|retained_intron|1/4|n.112+3678G>A||||||,T|intron_variant|MODIFIER|RBM23|ENSG00000100461|transcript|ENST00000556984|processed_transcript|1/4|n.151+3678G>A||||||,T|intron_variant|MODIFIER|RBM23|ENSG00000100461|transcript|ENST00000557549|protein_coding|1/4|c.-128-3418G>A||||||WARNING_TRANSCRIPT_NO_STOP_CODON,T|intron_variant|MODIFIER|RBM23|ENSG00000100461|transcript|ENST00000555676|protein_coding|1/4|c.-132-1476G>A||||||WARNING_TRANSCRIPT_NO_STOP_CODON,T|intron_variant|MODIFIER|RBM23|ENSG00000100461|transcript|ENST00000557571|protein_coding|2/4|c.-11+1707G>A||||||WARNING_TRANSCRIPT_NO_STOP_CODON,T|intron_variant|MODIFIER|RBM23|ENSG00000100461|transcript|ENST00000556862|protein_coding|1/5|c.-132-1476G>A||||||WARNING_TRANSCRIPT_INCOMPLETE,T|intron_variant|MODIFIER|RBM23|ENSG00000100461|transcript|ENST00000557464|protein_coding|1/4|c.-108-1500G>A||||||WARNING_TRANSCRIPT_NO_STOP_CODON,T|intron_variant|MODIFIER|RBM23|ENSG00000100461|transcript|ENST00000554618|protein_coding|1/5|c.-210-1305G>A||||||WARNING_TRANSCRIPT_INCOMPLETE,T|intron_variant|MODIFIER|RBM23|ENSG00000100461|transcript|ENST00000556365|processed_transcript|1/3|n.151+3678G>A||||||,T|intron_variant|MODIFIER|RBM23|ENSG00000100461|transcript|ENST00000553876|protein_coding|1/2|c.-11+2158G>A||||||WARNING_TRANSCRIPT_NO_STOP_CODON |
| PRMT5-AS1 | rs202141059 | 37 | 14 | 23384531 | 23384531 | + | SNP | C | C | T | SRR8586413 | Missense_Mutation | 0/1:76,290:366:99:12318,0,2359 | 366;ANN=T|upstream_gene_variant|MODIFIER|PRMT5-AS1|ENSG00000237054|transcript|ENST00000424245|antisense||n.-4134C>T|||||4134|,T|upstream_gene_variant|MODIFIER|RBM23|ENSG00000100461|transcript|ENST00000555691|protein_coding||c.-3929G>A|||||3530|WARNING_TRANSCRIPT_INCOMPLETE,T|upstream_gene_variant|MODIFIER|PRMT5-AS1|ENSG00000237054|transcript|ENST00000609885|antisense||n.-4299C>T|||||4299|,T|downstream_gene_variant|MODIFIER|RBM23|ENSG00000100461|transcript|ENST00000553777|processed_transcript||n.*2242G>A|||||2242|,T|intron_variant|MODIFIER|RBM23|ENSG00000100461|transcript|ENST00000359890|protein_coding|1/13|c.-11+3677G>A||||||,T|intron_variant|MODIFIER|RBM23|ENSG00000100461|transcript|ENST00000555209|protein_coding|1/10|c.-493+3677G>A||||||,T|intron_variant|MODIFIER|RBM23|ENSG00000100461|transcript|ENST00000557667|retained_intron|1/13|n.138+3677G>A||||||,T|intron_variant|MODIFIER|RBM23|ENSG00000100461|transcript|ENST00000399922|protein_coding|1/12|c.-11+3677G>A||||||,T|intron_variant|MODIFIER|RBM23|ENSG00000100461|transcript|ENST00000346528|protein_coding|1/11|c.-11+3677G>A||||||,T|intron_variant|MODIFIER|RBM23|ENSG00000100461|transcript|ENST00000542016|protein_coding|1/11|c.-360+3677G>A||||||,T|intron_variant|MODIFIER|RBM23|ENSG00000100461|transcript|ENST00000307814|retained_intron|1/10|n.151+3677G>A||||||,T|intron_variant|MODIFIER|RBM23|ENSG00000100461|transcript|ENST00000557403|protein_coding|1/5|c.-343+3677G>A||||||WARNING_TRANSCRIPT_INCOMPLETE,T|intron_variant|MODIFIER|RBM23|ENSG00000100461|transcript|ENST00000555722|protein_coding|1/4|c.-284+3677G>A||||||WARNING_TRANSCRIPT_INCOMPLETE,T|intron_variant|MODIFIER|RBM23|ENSG00000100461|transcript|ENST00000553920|processed_transcript|1/4|n.138+3677G>A||||||,T|intron_variant|MODIFIER|RBM23|ENSG00000100461|transcript|ENST00000557227|protein_coding|1/3|c.-306+3677G>A||||||WARNING_TRANSCRIPT_NO_STOP_CODON,T|intron_variant|MODIFIER|RBM23|ENSG00000100461|transcript|ENST00000555714|processed_transcript|1/5|n.151+3677G>A||||||,T|intron_variant|MODIFIER|RBM23|ENSG00000100461|transcript|ENST00000554256|protein_coding|1/5|c.-11+3677G>A||||||WARNING_TRANSCRIPT_NO_STOP_CODON,T|intron_variant|MODIFIER|RBM23|ENSG00000100461|transcript|ENST00000556687|retained_intron|1/3|n.151+3677G>A||||||,T|intron_variant|MODIFIER|RBM23|ENSG00000100461|transcript|ENST00000553902|retained_intron|1/2|n.151+3677G>A||||||,T|intron_variant|MODIFIER|RBM23|ENSG00000100461|transcript|ENST00000554955|retained_intron|1/4|n.112+3677G>A||||||,T|intron_variant|MODIFIER|RBM23|ENSG00000100461|transcript|ENST00000556984|processed_transcript|1/4|n.151+3677G>A||||||,T|intron_variant|MODIFIER|RBM23|ENSG00000100461|transcript|ENST00000557549|protein_coding|1/4|c.-128-3419G>A||||||WARNING_TRANSCRIPT_NO_STOP_CODON,T|intron_variant|MODIFIER|RBM23|ENSG00000100461|transcript|ENST00000555676|protein_coding|1/4|c.-132-1477G>A||||||WARNING_TRANSCRIPT_NO_STOP_CODON,T|intron_variant|MODIFIER|RBM23|ENSG00000100461|transcript|ENST00000557571|protein_coding|2/4|c.-11+1706G>A||||||WARNING_TRANSCRIPT_NO_STOP_CODON,T|intron_variant|MODIFIER|RBM23|ENSG00000100461|transcript|ENST00000556862|protein_coding|1/5|c.-132-1477G>A||||||WARNING_TRANSCRIPT_INCOMPLETE,T|intron_variant|MODIFIER|RBM23|ENSG00000100461|transcript|ENST00000557464|protein_coding|1/4|c.-108-1501G>A||||||WARNING_TRANSCRIPT_NO_STOP_CODON,T|intron_variant|MODIFIER|RBM23|ENSG00000100461|transcript|ENST00000554618|protein_coding|1/5|c.-210-1306G>A||||||WARNING_TRANSCRIPT_INCOMPLETE,T|intron_variant|MODIFIER|RBM23|ENSG00000100461|transcript|ENST00000556365|processed_transcript|1/3|n.151+3677G>A||||||,T|intron_variant|MODIFIER|RBM23|ENSG00000100461|transcript|ENST00000553876|protein_coding|1/2|c.-11+2157G>A||||||WARNING_TRANSCRIPT_NO_STOP_CODON |
| PRMT5-AS1 | rs149294569 | 37 | 14 | 23384580 | 23384580 | + | SNP | T | T | G | SRR8586413 | Missense_Mutation | 0/1:101,315:416:99:8252,0,3150 | 416;ANN=G|upstream_gene_variant|MODIFIER|PRMT5-AS1|ENSG00000237054|transcript|ENST00000424245|antisense||n.-4085T>G|||||4085|,G|upstream_gene_variant|MODIFIER|RBM23|ENSG00000100461|transcript|ENST00000555691|protein_coding||c.-3978A>C|||||3579|WARNING_TRANSCRIPT_INCOMPLETE,G|upstream_gene_variant|MODIFIER|PRMT5-AS1|ENSG00000237054|transcript|ENST00000609885|antisense||n.-4250T>G|||||4250|,G|downstream_gene_variant|MODIFIER|RBM23|ENSG00000100461|transcript|ENST00000553777|processed_transcript||n.*2193A>C|||||2193|,G|intron_variant|MODIFIER|RBM23|ENSG00000100461|transcript|ENST00000359890|protein_coding|1/13|c.-11+3628A>C||||||,G|intron_variant|MODIFIER|RBM23|ENSG00000100461|transcript|ENST00000555209|protein_coding|1/10|c.-493+3628A>C||||||,G|intron_variant|MODIFIER|RBM23|ENSG00000100461|transcript|ENST00000557667|retained_intron|1/13|n.138+3628A>C||||||,G|intron_variant|MODIFIER|RBM23|ENSG00000100461|transcript|ENST00000399922|protein_coding|1/12|c.-11+3628A>C||||||,G|intron_variant|MODIFIER|RBM23|ENSG00000100461|transcript|ENST00000346528|protein_coding|1/11|c.-11+3628A>C||||||,G|intron_variant|MODIFIER|RBM23|ENSG00000100461|transcript|ENST00000542016|protein_coding|1/11|c.-360+3628A>C||||||,G|intron_variant|MODIFIER|RBM23|ENSG00000100461|transcript|ENST00000307814|retained_intron|1/10|n.151+3628A>C||||||,G|intron_variant|MODIFIER|RBM23|ENSG00000100461|transcript|ENST00000557403|protein_coding|1/5|c.-343+3628A>C||||||WARNING_TRANSCRIPT_INCOMPLETE,G|intron_variant|MODIFIER|RBM23|ENSG00000100461|transcript|ENST00000555722|protein_coding|1/4|c.-284+3628A>C||||||WARNING_TRANSCRIPT_INCOMPLETE,G|intron_variant|MODIFIER|RBM23|ENSG00000100461|transcript|ENST00000553920|processed_transcript|1/4|n.138+3628A>C||||||,G|intron_variant|MODIFIER|RBM23|ENSG00000100461|transcript|ENST00000557227|protein_coding|1/3|c.-306+3628A>C||||||WARNING_TRANSCRIPT_NO_STOP_CODON,G|intron_variant|MODIFIER|RBM23|ENSG00000100461|transcript|ENST00000555714|processed_transcript|1/5|n.151+3628A>C||||||,G|intron_variant|MODIFIER|RBM23|ENSG00000100461|transcript|ENST00000554256|protein_coding|1/5|c.-11+3628A>C||||||WARNING_TRANSCRIPT_NO_STOP_CODON,G|intron_variant|MODIFIER|RBM23|ENSG00000100461|transcript|ENST00000556687|retained_intron|1/3|n.151+3628A>C||||||,G|intron_variant|MODIFIER|RBM23|ENSG00000100461|transcript|ENST00000553902|retained_intron|1/2|n.151+3628A>C||||||,G|intron_variant|MODIFIER|RBM23|ENSG00000100461|transcript|ENST00000554955|retained_intron|1/4|n.112+3628A>C||||||,G|intron_variant|MODIFIER|RBM23|ENSG00000100461|transcript|ENST00000556984|processed_transcript|1/4|n.151+3628A>C||||||,G|intron_variant|MODIFIER|RBM23|ENSG00000100461|transcript|ENST00000557549|protein_coding|1/4|c.-128-3468A>C||||||WARNING_TRANSCRIPT_NO_STOP_CODON,G|intron_variant|MODIFIER|RBM23|ENSG00000100461|transcript|ENST00000555676|protein_coding|1/4|c.-132-1526A>C||||||WARNING_TRANSCRIPT_NO_STOP_CODON,G|intron_variant|MODIFIER|RBM23|ENSG00000100461|transcript|ENST00000557571|protein_coding|2/4|c.-11+1657A>C||||||WARNING_TRANSCRIPT_NO_STOP_CODON,G|intron_variant|MODIFIER|RBM23|ENSG00000100461|transcript|ENST00000556862|protein_coding|1/5|c.-132-1526A>C||||||WARNING_TRANSCRIPT_INCOMPLETE,G|intron_variant|MODIFIER|RBM23|ENSG00000100461|transcript|ENST00000557464|protein_coding|1/4|c.-108-1550A>C||||||WARNING_TRANSCRIPT_NO_STOP_CODON,G|intron_variant|MODIFIER|RBM23|ENSG00000100461|transcript|ENST00000554618|protein_coding|1/5|c.-210-1355A>C||||||WARNING_TRANSCRIPT_INCOMPLETE,G|intron_variant|MODIFIER|RBM23|ENSG00000100461|transcript|ENST00000556365|processed_transcript|1/3|n.151+3628A>C||||||,G|intron_variant|MODIFIER|RBM23|ENSG00000100461|transcript|ENST00000553876|protein_coding|1/2|c.-11+2108A>C||||||WARNING_TRANSCRIPT_NO_STOP_CODON |
| PRMT5-AS1 | rs762151922 | 37 | 14 | 23384604 | 23384604 | + | SNP | C | C | T | SRR8586413 | Missense_Mutation | 0/1:85,318:403:99:13112,0,2176 | 403;ANN=T|upstream_gene_variant|MODIFIER|PRMT5-AS1|ENSG00000237054|transcript|ENST00000424245|antisense||n.-4061C>T|||||4061|,T|upstream_gene_variant|MODIFIER|RBM23|ENSG00000100461|transcript|ENST00000555691|protein_coding||c.-4002G>A|||||3603|WARNING_TRANSCRIPT_INCOMPLETE,T|upstream_gene_variant|MODIFIER|PRMT5-AS1|ENSG00000237054|transcript|ENST00000609885|antisense||n.-4226C>T|||||4226|,T|downstream_gene_variant|MODIFIER|RBM23|ENSG00000100461|transcript|ENST00000553777|processed_transcript||n.*2169G>A|||||2169|,T|intron_variant|MODIFIER|RBM23|ENSG00000100461|transcript|ENST00000359890|protein_coding|1/13|c.-11+3604G>A||||||,T|intron_variant|MODIFIER|RBM23|ENSG00000100461|transcript|ENST00000555209|protein_coding|1/10|c.-493+3604G>A||||||,T|intron_variant|MODIFIER|RBM23|ENSG00000100461|transcript|ENST00000557667|retained_intron|1/13|n.138+3604G>A||||||,T|intron_variant|MODIFIER|RBM23|ENSG00000100461|transcript|ENST00000399922|protein_coding|1/12|c.-11+3604G>A||||||,T|intron_variant|MODIFIER|RBM23|ENSG00000100461|transcript|ENST00000346528|protein_coding|1/11|c.-11+3604G>A||||||,T|intron_variant|MODIFIER|RBM23|ENSG00000100461|transcript|ENST00000542016|protein_coding|1/11|c.-360+3604G>A||||||,T|intron_variant|MODIFIER|RBM23|ENSG00000100461|transcript|ENST00000307814|retained_intron|1/10|n.151+3604G>A||||||,T|intron_variant|MODIFIER|RBM23|ENSG00000100461|transcript|ENST00000557403|protein_coding|1/5|c.-343+3604G>A||||||WARNING_TRANSCRIPT_INCOMPLETE,T|intron_variant|MODIFIER|RBM23|ENSG00000100461|transcript|ENST00000555722|protein_coding|1/4|c.-284+3604G>A||||||WARNING_TRANSCRIPT_INCOMPLETE,T|intron_variant|MODIFIER|RBM23|ENSG00000100461|transcript|ENST00000553920|processed_transcript|1/4|n.138+3604G>A||||||,T|intron_variant|MODIFIER|RBM23|ENSG00000100461|transcript|ENST00000557227|protein_coding|1/3|c.-306+3604G>A||||||WARNING_TRANSCRIPT_NO_STOP_CODON,T|intron_variant|MODIFIER|RBM23|ENSG00000100461|transcript|ENST00000555714|processed_transcript|1/5|n.151+3604G>A||||||,T|intron_variant|MODIFIER|RBM23|ENSG00000100461|transcript|ENST00000554256|protein_coding|1/5|c.-11+3604G>A||||||WARNING_TRANSCRIPT_NO_STOP_CODON,T|intron_variant|MODIFIER|RBM23|ENSG00000100461|transcript|ENST00000556687|retained_intron|1/3|n.151+3604G>A||||||,T|intron_variant|MODIFIER|RBM23|ENSG00000100461|transcript|ENST00000553902|retained_intron|1/2|n.151+3604G>A||||||,T|intron_variant|MODIFIER|RBM23|ENSG00000100461|transcript|ENST00000554955|retained_intron|1/4|n.112+3604G>A||||||,T|intron_variant|MODIFIER|RBM23|ENSG00000100461|transcript|ENST00000556984|processed_transcript|1/4|n.151+3604G>A||||||,T|intron_variant|MODIFIER|RBM23|ENSG00000100461|transcript|ENST00000557549|protein_coding|1/4|c.-128-3492G>A||||||WARNING_TRANSCRIPT_NO_STOP_CODON,T|intron_variant|MODIFIER|RBM23|ENSG00000100461|transcript|ENST00000555676|protein_coding|1/4|c.-132-1550G>A||||||WARNING_TRANSCRIPT_NO_STOP_CODON,T|intron_variant|MODIFIER|RBM23|ENSG00000100461|transcript|ENST00000557571|protein_coding|2/4|c.-11+1633G>A||||||WARNING_TRANSCRIPT_NO_STOP_CODON,T|intron_variant|MODIFIER|RBM23|ENSG00000100461|transcript|ENST00000556862|protein_coding|1/5|c.-132-1550G>A||||||WARNING_TRANSCRIPT_INCOMPLETE,T|intron_variant|MODIFIER|RBM23|ENSG00000100461|transcript|ENST00000557464|protein_coding|1/4|c.-108-1574G>A||||||WARNING_TRANSCRIPT_NO_STOP_CODON,T|intron_variant|MODIFIER|RBM23|ENSG00000100461|transcript|ENST00000554618|protein_coding|1/5|c.-210-1379G>A||||||WARNING_TRANSCRIPT_INCOMPLETE,T|intron_variant|MODIFIER|RBM23|ENSG00000100461|transcript|ENST00000556365|processed_transcript|1/3|n.151+3604G>A||||||,T|intron_variant|MODIFIER|RBM23|ENSG00000100461|transcript|ENST00000553876|protein_coding|1/2|c.-11+2084G>A||||||WARNING_TRANSCRIPT_NO_STOP_CODON |
| PRMT5-AS1 | rs753335327 | 37 | 14 | 23384640 | 23384640 | + | SNP | C | C | T | SRR8586413 | Missense_Mutation | 0/1:71,289:360:99:9100,0,1808 | 360;ANN=T|upstream_gene_variant|MODIFIER|PRMT5-AS1|ENSG00000237054|transcript|ENST00000424245|antisense||n.-4025C>T|||||4025|,T|upstream_gene_variant|MODIFIER|RBM23|ENSG00000100461|transcript|ENST00000555691|protein_coding||c.-4038G>A|||||3639|WARNING_TRANSCRIPT_INCOMPLETE,T|upstream_gene_variant|MODIFIER|PRMT5-AS1|ENSG00000237054|transcript|ENST00000609885|antisense||n.-4190C>T|||||4190|,T|downstream_gene_variant|MODIFIER|RBM23|ENSG00000100461|transcript|ENST00000553777|processed_transcript||n.*2133G>A|||||2133|,T|intron_variant|MODIFIER|RBM23|ENSG00000100461|transcript|ENST00000359890|protein_coding|1/13|c.-11+3568G>A||||||,T|intron_variant|MODIFIER|RBM23|ENSG00000100461|transcript|ENST00000555209|protein_coding|1/10|c.-493+3568G>A||||||,T|intron_variant|MODIFIER|RBM23|ENSG00000100461|transcript|ENST00000557667|retained_intron|1/13|n.138+3568G>A||||||,T|intron_variant|MODIFIER|RBM23|ENSG00000100461|transcript|ENST00000399922|protein_coding|1/12|c.-11+3568G>A||||||,T|intron_variant|MODIFIER|RBM23|ENSG00000100461|transcript|ENST00000346528|protein_coding|1/11|c.-11+3568G>A||||||,T|intron_variant|MODIFIER|RBM23|ENSG00000100461|transcript|ENST00000542016|protein_coding|1/11|c.-360+3568G>A||||||,T|intron_variant|MODIFIER|RBM23|ENSG00000100461|transcript|ENST00000307814|retained_intron|1/10|n.151+3568G>A||||||,T|intron_variant|MODIFIER|RBM23|ENSG00000100461|transcript|ENST00000557403|protein_coding|1/5|c.-343+3568G>A||||||WARNING_TRANSCRIPT_INCOMPLETE,T|intron_variant|MODIFIER|RBM23|ENSG00000100461|transcript|ENST00000555722|protein_coding|1/4|c.-284+3568G>A||||||WARNING_TRANSCRIPT_INCOMPLETE,T|intron_variant|MODIFIER|RBM23|ENSG00000100461|transcript|ENST00000553920|processed_transcript|1/4|n.138+3568G>A||||||,T|intron_variant|MODIFIER|RBM23|ENSG00000100461|transcript|ENST00000557227|protein_coding|1/3|c.-306+3568G>A||||||WARNING_TRANSCRIPT_NO_STOP_CODON,T|intron_variant|MODIFIER|RBM23|ENSG00000100461|transcript|ENST00000555714|processed_transcript|1/5|n.151+3568G>A||||||,T|intron_variant|MODIFIER|RBM23|ENSG00000100461|transcript|ENST00000554256|protein_coding|1/5|c.-11+3568G>A||||||WARNING_TRANSCRIPT_NO_STOP_CODON,T|intron_variant|MODIFIER|RBM23|ENSG00000100461|transcript|ENST00000556687|retained_intron|1/3|n.151+3568G>A||||||,T|intron_variant|MODIFIER|RBM23|ENSG00000100461|transcript|ENST00000553902|retained_intron|1/2|n.151+3568G>A||||||,T|intron_variant|MODIFIER|RBM23|ENSG00000100461|transcript|ENST00000554955|retained_intron|1/4|n.112+3568G>A||||||,T|intron_variant|MODIFIER|RBM23|ENSG00000100461|transcript|ENST00000556984|processed_transcript|1/4|n.151+3568G>A||||||,T|intron_variant|MODIFIER|RBM23|ENSG00000100461|transcript|ENST00000557549|protein_coding|1/4|c.-128-3528G>A||||||WARNING_TRANSCRIPT_NO_STOP_CODON,T|intron_variant|MODIFIER|RBM23|ENSG00000100461|transcript|ENST00000555676|protein_coding|1/4|c.-132-1586G>A||||||WARNING_TRANSCRIPT_NO_STOP_CODON,T|intron_variant|MODIFIER|RBM23|ENSG00000100461|transcript|ENST00000557571|protein_coding|2/4|c.-11+1597G>A||||||WARNING_TRANSCRIPT_NO_STOP_CODON,T|intron_variant|MODIFIER|RBM23|ENSG00000100461|transcript|ENST00000556862|protein_coding|1/5|c.-132-1586G>A||||||WARNING_TRANSCRIPT_INCOMPLETE,T|intron_variant|MODIFIER|RBM23|ENSG00000100461|transcript|ENST00000557464|protein_coding|1/4|c.-108-1610G>A||||||WARNING_TRANSCRIPT_NO_STOP_CODON,T|intron_variant|MODIFIER|RBM23|ENSG00000100461|transcript|ENST00000554618|protein_coding|1/5|c.-210-1415G>A||||||WARNING_TRANSCRIPT_INCOMPLETE,T|intron_variant|MODIFIER|RBM23|ENSG00000100461|transcript|ENST00000556365|processed_transcript|1/3|n.151+3568G>A||||||,T|intron_variant|MODIFIER|RBM23|ENSG00000100461|transcript|ENST00000553876|protein_coding|1/2|c.-11+2048G>A||||||WARNING_TRANSCRIPT_NO_STOP_CODON |
| PRMT5-AS1 | rs370121893 | 37 | 14 | 23386023 | 23386023 | + | SNP | G | G | A | SRR8586413 | Missense_Mutation | 0/1:133,35:168:99:719,0,4000 | 169;ANN=A|upstream_gene_variant|MODIFIER|PRMT5-AS1|ENSG00000237054|transcript|ENST00000424245|antisense||n.-2642G>A|||||2642|,A|upstream_gene_variant|MODIFIER|PRMT5-AS1|ENSG00000237054|transcript|ENST00000609885|antisense||n.-2807G>A|||||2807|,A|upstream_gene_variant|MODIFIER|PRMT5-AS1|ENSG00000237054|transcript|ENST00000457443|antisense||n.-3825G>A|||||3825|,A|upstream_gene_variant|MODIFIER|PRMT5-AS1|ENSG00000237054|transcript|ENST00000599580|antisense||n.-4097G>A|||||4097|,A|upstream_gene_variant|MODIFIER|PRMT5-AS1|ENSG00000237054|transcript|ENST00000595662|antisense||n.-4224G>A|||||4224|,A|upstream_gene_variant|MODIFIER|PRMT5-AS1|ENSG00000237054|transcript|ENST00000587245|antisense||n.-4224G>A|||||4224|,A|upstream_gene_variant|MODIFIER|PRMT5-AS1|ENSG00000237054|transcript|ENST00000590290|antisense||n.-4227G>A|||||4227|,A|downstream_gene_variant|MODIFIER|PRMT5|ENSG00000100462|transcript|ENST00000324366|protein_coding||c.*4090C>T|||||3697|,A|downstream_gene_variant|MODIFIER|RBM23|ENSG00000100461|transcript|ENST00000553777|processed_transcript||n.*750C>T|||||750|,A|downstream_gene_variant|MODIFIER|PRMT5|ENSG00000100462|transcript|ENST00000397441|protein_coding||c.*4090C>T|||||3711|,A|downstream_gene_variant|MODIFIER|PRMT5|ENSG00000100462|transcript|ENST00000557443|protein_coding||c.*3867C>T|||||3715|WARNING_TRANSCRIPT_NO_START_CODON,A|downstream_gene_variant|MODIFIER|PRMT5|ENSG00000100462|transcript|ENST00000476175|processed_transcript||n.*3715C>T|||||3715|,A|downstream_gene_variant|MODIFIER|PRMT5|ENSG00000100462|transcript|ENST00000397440|protein_coding||c.*4090C>T|||||3715|,A|downstream_gene_variant|MODIFIER|PRMT5|ENSG00000100462|transcript|ENST00000216350|protein_coding||c.*4090C>T|||||3716|,A|downstream_gene_variant|MODIFIER|PRMT5|ENSG00000100462|transcript|ENST00000555454|protein_coding||c.*4233C>T|||||3996|WARNING_TRANSCRIPT_NO_START_CODON,A|downstream_gene_variant|MODIFIER|PRMT5|ENSG00000100462|transcript|ENST00000454731|protein_coding||c.*4090C>T|||||4011|WARNING_TRANSCRIPT_NO_START_CODON,A|downstream_gene_variant|MODIFIER|PRMT5|ENSG00000100462|transcript|ENST00000553915|nonsense_mediated_decay||c.*10782C>T|||||4043|,A|downstream_gene_variant|MODIFIER|PRMT5|ENSG00000100462|transcript|ENST00000538452|protein_coding||c.*4090C>T|||||4043|,A|downstream_gene_variant|MODIFIER|PRMT5|ENSG00000100462|transcript|ENST00000553897|protein_coding||c.*4090C>T|||||4062|,A|intron_variant|MODIFIER|RBM23|ENSG00000100461|transcript|ENST00000359890|protein_coding|1/13|c.-11+2185C>T||||||,A|intron_variant|MODIFIER|RBM23|ENSG00000100461|transcript|ENST00000555209|protein_coding|1/10|c.-493+2185C>T||||||,A|intron_variant|MODIFIER|RBM23|ENSG00000100461|transcript|ENST00000557667|retained_intron|1/13|n.138+2185C>T||||||,A|intron_variant|MODIFIER|RBM23|ENSG00000100461|transcript|ENST00000399922|protein_coding|1/12|c.-11+2185C>T||||||,A|intron_variant|MODIFIER|RBM23|ENSG00000100461|transcript|ENST00000346528|protein_coding|1/11|c.-11+2185C>T||||||,A|intron_variant|MODIFIER|RBM23|ENSG00000100461|transcript|ENST00000542016|protein_coding|1/11|c.-360+2185C>T||||||,A|intron_variant|MODIFIER|RBM23|ENSG00000100461|transcript|ENST00000307814|retained_intron|1/10|n.151+2185C>T||||||,A|intron_variant|MODIFIER|RBM23|ENSG00000100461|transcript|ENST00000557403|protein_coding|1/5|c.-343+2185C>T||||||WARNING_TRANSCRIPT_INCOMPLETE,A|intron_variant|MODIFIER|RBM23|ENSG00000100461|transcript|ENST00000555722|protein_coding|1/4|c.-284+2185C>T||||||WARNING_TRANSCRIPT_INCOMPLETE,A|intron_variant|MODIFIER|RBM23|ENSG00000100461|transcript|ENST00000553920|processed_transcript|1/4|n.138+2185C>T||||||,A|intron_variant|MODIFIER|RBM23|ENSG00000100461|transcript|ENST00000557227|protein_coding|1/3|c.-306+2185C>T||||||WARNING_TRANSCRIPT_NO_STOP_CODON,A|intron_variant|MODIFIER|RBM23|ENSG00000100461|transcript|ENST00000555714|processed_transcript|1/5|n.151+2185C>T||||||,A|intron_variant|MODIFIER|RBM23|ENSG00000100461|transcript|ENST00000554256|protein_coding|1/5|c.-11+2185C>T||||||WARNING_TRANSCRIPT_NO_STOP_CODON,A|intron_variant|MODIFIER|RBM23|ENSG00000100461|transcript|ENST00000556687|retained_intron|1/3|n.151+2185C>T||||||,A|intron_variant|MODIFIER|RBM23|ENSG00000100461|transcript|ENST00000553902|retained_intron|1/2|n.151+2185C>T||||||,A|intron_variant|MODIFIER|RBM23|ENSG00000100461|transcript|ENST00000554955|retained_intron|1/4|n.112+2185C>T||||||,A|intron_variant|MODIFIER|RBM23|ENSG00000100461|transcript|ENST00000556984|processed_transcript|1/4|n.151+2185C>T||||||,A|intron_variant|MODIFIER|RBM23|ENSG00000100461|transcript|ENST00000557549|protein_coding|1/4|c.-129+2185C>T||||||WARNING_TRANSCRIPT_NO_STOP_CODON,A|intron_variant|MODIFIER|RBM23|ENSG00000100461|transcript|ENST00000555676|protein_coding|1/4|c.-133+2185C>T||||||WARNING_TRANSCRIPT_NO_STOP_CODON,A|intron_variant|MODIFIER|RBM23|ENSG00000100461|transcript|ENST00000557571|protein_coding|2/4|c.-11+214C>T||||||WARNING_TRANSCRIPT_NO_STOP_CODON,A|intron_variant|MODIFIER|RBM23|ENSG00000100461|transcript|ENST00000556862|protein_coding|1/5|c.-133+2185C>T||||||WARNING_TRANSCRIPT_INCOMPLETE,A|intron_variant|MODIFIER|RBM23|ENSG00000100461|transcript|ENST00000557464|protein_coding|1/4|c.-109+2185C>T||||||WARNING_TRANSCRIPT_NO_STOP_CODON,A|intron_variant|MODIFIER|RBM23|ENSG00000100461|transcript|ENST00000554618|protein_coding|1/5|c.-211+2185C>T||||||WARNING_TRANSCRIPT_INCOMPLETE,A|intron_variant|MODIFIER|RBM23|ENSG00000100461|transcript|ENST00000556365|processed_transcript|1/3|n.151+2185C>T||||||,A|intron_variant|MODIFIER|RBM23|ENSG00000100461|transcript|ENST00000553876|protein_coding|1/2|c.-11+665C>T||||||WARNING_TRANSCRIPT_NO_STOP_CODON |
| RBM23 | rs780653498 | 37 | 14 | 23388975 | 23388975 | + | SNP | T | T | C | SRR8586413 | Missense_Mutation | 0/1:224,169:393:99:4150,0,5658 | 408;ANN=C|upstream_gene_variant|MODIFIER|RBM23|ENSG00000100461|transcript|ENST00000359890|protein_coding||c.-8373A>G|||||582|,C|upstream_gene_variant|MODIFIER|RBM23|ENSG00000100461|transcript|ENST00000555209|protein_coding||c.-14778A>G|||||637|,C|upstream_gene_variant|MODIFIER|RBM23|ENSG00000100461|transcript|ENST00000557667|retained_intron||n.-630A>G|||||630|,C|upstream_gene_variant|MODIFIER|RBM23|ENSG00000100461|transcript|ENST00000399922|protein_coding||c.-8373A>G|||||584|,C|upstream_gene_variant|MODIFIER|RBM23|ENSG00000100461|transcript|ENST00000346528|protein_coding||c.-8373A>G|||||588|,C|upstream_gene_variant|MODIFIER|RBM23|ENSG00000100461|transcript|ENST00000542016|protein_coding||c.-14368A>G|||||588|,C|upstream_gene_variant|MODIFIER|RBM23|ENSG00000100461|transcript|ENST00000307814|retained_intron||n.-617A>G|||||617|,C|upstream_gene_variant|MODIFIER|RBM23|ENSG00000100461|transcript|ENST00000557403|protein_coding||c.-14368A>G|||||628|WARNING_TRANSCRIPT_INCOMPLETE,C|upstream_gene_variant|MODIFIER|RBM23|ENSG00000100461|transcript|ENST00000555722|protein_coding||c.-14368A>G|||||601|WARNING_TRANSCRIPT_INCOMPLETE,C|upstream_gene_variant|MODIFIER|RBM23|ENSG00000100461|transcript|ENST00000553920|processed_transcript||n.-630A>G|||||630|,C|upstream_gene_variant|MODIFIER|RBM23|ENSG00000100461|transcript|ENST00000557227|protein_coding||c.-14368A>G|||||584|WARNING_TRANSCRIPT_NO_STOP_CODON,C|upstream_gene_variant|MODIFIER|RBM23|ENSG00000100461|transcript|ENST00000555714|processed_transcript||n.-617A>G|||||617|,C|upstream_gene_variant|MODIFIER|RBM23|ENSG00000100461|transcript|ENST00000554256|protein_coding||c.-8373A>G|||||629|WARNING_TRANSCRIPT_NO_STOP_CODON,C|upstream_gene_variant|MODIFIER|RBM23|ENSG00000100461|transcript|ENST00000556687|retained_intron||n.-617A>G|||||617|,C|upstream_gene_variant|MODIFIER|RBM23|ENSG00000100461|transcript|ENST00000553902|retained_intron||n.-617A>G|||||617|,C|upstream_gene_variant|MODIFIER|RBM23|ENSG00000100461|transcript|ENST00000554955|retained_intron||n.-656A>G|||||656|,C|upstream_gene_variant|MODIFIER|RBM23|ENSG00000100461|transcript|ENST00000556984|processed_transcript||n.-617A>G|||||617|,C|upstream_gene_variant|MODIFIER|RBM23|ENSG00000100461|transcript|ENST00000557549|protein_coding||c.-8373A>G|||||617|WARNING_TRANSCRIPT_NO_STOP_CODON,C|upstream_gene_variant|MODIFIER|RBM23|ENSG00000100461|transcript|ENST00000555676|protein_coding||c.-8373A>G|||||617|WARNING_TRANSCRIPT_NO_STOP_CODON,C|upstream_gene_variant|MODIFIER|RBM23|ENSG00000100461|transcript|ENST00000557571|protein_coding||c.-8373A>G|||||617|WARNING_TRANSCRIPT_NO_STOP_CODON,C|upstream_gene_variant|MODIFIER|RBM23|ENSG00000100461|transcript|ENST00000556862|protein_coding||c.-8373A>G|||||609|WARNING_TRANSCRIPT_INCOMPLETE,C|upstream_gene_variant|MODIFIER|RBM23|ENSG00000100461|transcript|ENST00000557464|protein_coding||c.-8373A>G|||||617|WARNING_TRANSCRIPT_NO_STOP_CODON,C|upstream_gene_variant|MODIFIER|RBM23|ENSG00000100461|transcript|ENST00000554618|protein_coding||c.-8373A>G|||||617|WARNING_TRANSCRIPT_INCOMPLETE,C|upstream_gene_variant|MODIFIER|RBM23|ENSG00000100461|transcript|ENST00000556365|processed_transcript||n.-617A>G|||||617|,C|upstream_gene_variant|MODIFIER|RBM23|ENSG00000100461|transcript|ENST00000553876|protein_coding||c.-8373A>G|||||2210|WARNING_TRANSCRIPT_NO_STOP_CODON,C|upstream_gene_variant|MODIFIER|RBM23|ENSG00000100461|transcript|ENST00000553777|processed_transcript||n.-679A>G|||||679|,C|upstream_gene_variant|MODIFIER|PRMT5-AS1|ENSG00000237054|transcript|ENST00000457443|antisense||n.-873T>C|||||873|,C|upstream_gene_variant|MODIFIER|PRMT5-AS1|ENSG00000237054|transcript|ENST00000599580|antisense||n.-1145T>C|||||1145|,C|upstream_gene_variant|MODIFIER|PRMT5-AS1|ENSG00000237054|transcript|ENST00000595662|antisense||n.-1272T>C|||||1272|,C|upstream_gene_variant|MODIFIER|PRMT5-AS1|ENSG00000237054|transcript|ENST00000587245|antisense||n.-1272T>C|||||1272|,C|upstream_gene_variant|MODIFIER|PRMT5-AS1|ENSG00000237054|transcript|ENST00000590290|antisense||n.-1275T>C|||||1275|,C|downstream_gene_variant|MODIFIER|PRMT5|ENSG00000100462|transcript|ENST00000324366|protein_coding||c.*1138A>G|||||745|,C|downstream_gene_variant|MODIFIER|PRMT5|ENSG00000100462|transcript|ENST00000397441|protein_coding||c.*1138A>G|||||759|,C|downstream_gene_variant|MODIFIER|PRMT5|ENSG00000100462|transcript|ENST00000557443|protein_coding||c.*915A>G|||||763|WARNING_TRANSCRIPT_NO_START_CODON,C|downstream_gene_variant|MODIFIER|PRMT5|ENSG00000100462|transcript|ENST00000476175|processed_transcript||n.*763A>G|||||763|,C|downstream_gene_variant|MODIFIER|PRMT5|ENSG00000100462|transcript|ENST00000397440|protein_coding||c.*1138A>G|||||763|,C|downstream_gene_variant|MODIFIER|PRMT5|ENSG00000100462|transcript|ENST00000216350|protein_coding||c.*1138A>G|||||764|,C|downstream_gene_variant|MODIFIER|PRMT5|ENSG00000100462|transcript|ENST00000555454|protein_coding||c.*1281A>G|||||1044|WARNING_TRANSCRIPT_NO_START_CODON,C|downstream_gene_variant|MODIFIER|PRMT5|ENSG00000100462|transcript|ENST00000454731|protein_coding||c.*1138A>G|||||1059|WARNING_TRANSCRIPT_NO_START_CODON,C|downstream_gene_variant|MODIFIER|PRMT5|ENSG00000100462|transcript|ENST00000553915|nonsense_mediated_decay||c.*7830A>G|||||1091|,C|downstream_gene_variant|MODIFIER|PRMT5|ENSG00000100462|transcript|ENST00000538452|protein_coding||c.*1138A>G|||||1091|,C|downstream_gene_variant|MODIFIER|PRMT5|ENSG00000100462|transcript|ENST00000553897|protein_coding||c.*1138A>G|||||1110|,C|downstream_gene_variant|MODIFIER|PRMT5|ENSG00000100462|transcript|ENST00000557758|retained_intron||n.*2366A>G|||||2366|,C|downstream_gene_variant|MODIFIER|PRMT5|ENSG00000100462|transcript|ENST00000553502|protein_coding||c.*4396A>G|||||4396|WARNING_TRANSCRIPT_NO_START_CODON,C|downstream_gene_variant|MODIFIER|PRMT5|ENSG00000100462|transcript|ENST00000555530|protein_coding||c.*4551A>G|||||4551|WARNING_TRANSCRIPT_NO_START_CODON,C|downstream_gene_variant|MODIFIER|PRMT5|ENSG00000100462|transcript|ENST00000556043|protein_coding||c.*4733A>G|||||4733|WARNING_TRANSCRIPT_NO_STOP_CODON,C|downstream_gene_variant|MODIFIER|PRMT5|ENSG00000100462|transcript|ENST00000553550|protein_coding||c.*4872A>G|||||4872|WARNING_TRANSCRIPT_NO_STOP_CODON,C|downstream_gene_variant|MODIFIER|PRMT5|ENSG00000100462|transcript|ENST00000554716|processed_transcript||n.*4897A>G|||||4897|,C|non_coding_transcript_exon_variant|MODIFIER|PRMT5-AS1|ENSG00000237054|transcript|ENST00000424245|antisense|1/2|n.311T>C||||||,C|non_coding_transcript_exon_variant|MODIFIER|PRMT5-AS1|ENSG00000237054|transcript|ENST00000609885|antisense|1/2|n.146T>C|||||| |
| RP11-298I3.1 | rs765316931 | 37 | 14 | 23397004 | 23397004 | + | SNP | C | C | T | SRR8586413 | Missense_Mutation | 0/1:98,396:494:99:12729,0,1636 | 495;ANN=T|upstream_gene_variant|MODIFIER|RP11-298I3.1|ENSG00000257285|transcript|ENST00000548322|antisense||n.-1850C>T|||||1850|,T|upstream_gene_variant|MODIFIER|PRMT5|ENSG00000100462|transcript|ENST00000476175|processed_transcript||n.-4974G>A|||||4974|,T|upstream_gene_variant|MODIFIER|PRMT5|ENSG00000100462|transcript|ENST00000555454|protein_coding||c.-3616G>A|||||3616|WARNING_TRANSCRIPT_NO_START_CODON,T|upstream_gene_variant|MODIFIER|PRMT5|ENSG00000100462|transcript|ENST00000454731|protein_coding||c.-4634G>A|||||4633|WARNING_TRANSCRIPT_NO_START_CODON,T|upstream_gene_variant|MODIFIER|PRMT5|ENSG00000100462|transcript|ENST00000557758|retained_intron||n.-4885G>A|||||4885|,T|upstream_gene_variant|MODIFIER|PRMT5|ENSG00000100462|transcript|ENST00000553502|protein_coding||c.-2749G>A|||||2749|WARNING_TRANSCRIPT_NO_START_CODON,T|upstream_gene_variant|MODIFIER|PRMT5|ENSG00000100462|transcript|ENST00000556043|protein_coding||c.-1630G>A|||||1339|WARNING_TRANSCRIPT_NO_STOP_CODON,T|upstream_gene_variant|MODIFIER|PRMT5|ENSG00000100462|transcript|ENST00000553417|retained_intron||n.-199G>A|||||199|,T|upstream_gene_variant|MODIFIER|RP11-298I3.1|ENSG00000257285|transcript|ENST00000548819|antisense||n.-1814C>T|||||1814|,T|downstream_gene_variant|MODIFIER|PRMT5-AS1|ENSG00000237054|transcript|ENST00000424245|antisense||n.*4388C>T|||||4388|,T|downstream_gene_variant|MODIFIER|PRMT5-AS1|ENSG00000237054|transcript|ENST00000609885|antisense||n.*4544C>T|||||4544|,T|downstream_gene_variant|MODIFIER|PRMT5-AS1|ENSG00000237054|transcript|ENST00000457443|antisense||n.*4392C>T|||||4392|,T|downstream_gene_variant|MODIFIER|PRMT5-AS1|ENSG00000237054|transcript|ENST00000599580|antisense||n.*965C>T|||||965|,T|downstream_gene_variant|MODIFIER|PRMT5-AS1|ENSG00000237054|transcript|ENST00000595662|antisense||n.*1176C>T|||||1176|,T|downstream_gene_variant|MODIFIER|PRMT5-AS1|ENSG00000237054|transcript|ENST00000587245|antisense||n.*970C>T|||||970|,T|downstream_gene_variant|MODIFIER|PRMT5-AS1|ENSG00000237054|transcript|ENST00000590290|antisense||n.*899C>T|||||899|,T|downstream_gene_variant|MODIFIER|PRMT5|ENSG00000100462|transcript|ENST00000557015|retained_intron||n.*336G>A|||||336|,T|downstream_gene_variant|MODIFIER|PRMT5|ENSG00000100462|transcript|ENST00000556426|processed_transcript||n.*468G>A|||||468|,T|intron_variant|MODIFIER|PRMT5|ENSG00000100462|transcript|ENST00000324366|protein_coding|3/16|c.316-135G>A||||||,T|intron_variant|MODIFIER|PRMT5|ENSG00000100462|transcript|ENST00000397441|protein_coding|3/16|c.265-135G>A||||||,T|intron_variant|MODIFIER|PRMT5|ENSG00000100462|transcript|ENST00000397440|protein_coding|3/12|c.264+331G>A||||||,T|intron_variant|MODIFIER|PRMT5|ENSG00000100462|transcript|ENST00000216350|protein_coding|2/15|c.179-181G>A||||||,T|intron_variant|MODIFIER|PRMT5|ENSG00000100462|transcript|ENST00000553915|nonsense_mediated_decay|2/15|c.146-135G>A||||||,T|intron_variant|MODIFIER|PRMT5|ENSG00000100462|transcript|ENST00000538452|protein_coding|2/15|c.-3-135G>A||||||,T|intron_variant|MODIFIER|PRMT5|ENSG00000100462|transcript|ENST00000553897|protein_coding|2/15|c.230-181G>A||||||,T|intron_variant|MODIFIER|PRMT5|ENSG00000100462|transcript|ENST00000555530|protein_coding|1/8|c.19-135G>A||||||WARNING_TRANSCRIPT_NO_START_CODON,T|intron_variant|MODIFIER|PRMT5|ENSG00000100462|transcript|ENST00000553550|protein_coding|3/4|c.315+331G>A||||||WARNING_TRANSCRIPT_NO_STOP_CODON,T|intron_variant|MODIFIER|PRMT5|ENSG00000100462|transcript|ENST00000554716|processed_transcript|3/4|n.352+331G>A||||||,T|intron_variant|MODIFIER|PRMT5|ENSG00000100462|transcript|ENST00000553787|nonsense_mediated_decay|1/5|c.111-181G>A||||||,T|intron_variant|MODIFIER|PRMT5|ENSG00000100462|transcript|ENST00000553641|processed_transcript|3/6|n.339-135G>A||||||,T|intron_variant|MODIFIER|PRMT5|ENSG00000100462|transcript|ENST00000554867|protein_coding|3/5|c.315+331G>A||||||WARNING_TRANSCRIPT_NO_STOP_CODON,T|intron_variant|MODIFIER|PRMT5|ENSG00000100462|transcript|ENST00000557415|nonsense_mediated_decay|2/5|c.197-135G>A||||||,T|intron_variant|MODIFIER|PRMT5|ENSG00000100462|transcript|ENST00000556616|protein_coding|2/5|c.202-135G>A||||||WARNING_TRANSCRIPT_INCOMPLETE,T|intron_variant|MODIFIER|PRMT5|ENSG00000100462|transcript|ENST00000554910|protein_coding|3/6|c.190-135G>A||||||WARNING_TRANSCRIPT_INCOMPLETE,T|intron_variant|MODIFIER|PRMT5|ENSG00000100462|transcript|ENST00000421938|protein_coding|3/4|c.346-135G>A||||||WARNING_TRANSCRIPT_NO_STOP_CODON,T|intron_variant|MODIFIER|PRMT5|ENSG00000100462|transcript|ENST00000556032|retained_intron|2/2|n.509-135G>A|||||| |
| HAUS4 | rs773795845 | 37 | 14 | 23415266 | 23415266 | + | SNP | G | G | A | SRR8586413 | Nonsense_Mutation | 0/1:428,416:844:99:11970,0,23173 | 845;ANN=A|downstream_gene_variant|MODIFIER|HAUS4|ENSG00000092036|transcript|ENST00000206474|protein_coding||c.*468C>T|||||171|,A|downstream_gene_variant|MODIFIER|RP11-298I3.5|ENSG00000259132|transcript|ENST00000555074|protein_coding||c.*468C>T|||||213|WARNING_TRANSCRIPT_NO_START_CODON,A|downstream_gene_variant|MODIFIER|HAUS4|ENSG00000092036|transcript|ENST00000490506|protein_coding||c.*468C>T|||||171|,A|downstream_gene_variant|MODIFIER|HAUS4|ENSG00000092036|transcript|ENST00000554446|processed_transcript||n.*172C>T|||||172|,A|downstream_gene_variant|MODIFIER|HAUS4|ENSG00000092036|transcript|ENST00000541587|protein_coding||c.*468C>T|||||172|,A|downstream_gene_variant|MODIFIER|HAUS4|ENSG00000092036|transcript|ENST00000397409|protein_coding||c.*468C>T|||||179|,A|downstream_gene_variant|MODIFIER|HAUS4|ENSG00000092036|transcript|ENST00000342454|protein_coding||c.*468C>T|||||179|,A|downstream_gene_variant|MODIFIER|HAUS4|ENSG00000092036|transcript|ENST00000347758|protein_coding||c.*468C>T|||||179|,A|downstream_gene_variant|MODIFIER|HAUS4|ENSG00000092036|transcript|ENST00000555367|protein_coding||c.*468C>T|||||204|,A|downstream_gene_variant|MODIFIER|HAUS4|ENSG00000092036|transcript|ENST00000555986|protein_coding||c.*468C>T|||||213|,A|downstream_gene_variant|MODIFIER|HAUS4|ENSG00000092036|transcript|ENST00000556421|retained_intron||n.*504C>T|||||504|,A|downstream_gene_variant|MODIFIER|HAUS4|ENSG00000092036|transcript|ENST00000553859|nonsense_mediated_decay||c.*1550C>T|||||560|WARNING_TRANSCRIPT_NO_START_CODON,A|downstream_gene_variant|MODIFIER|HAUS4|ENSG00000092036|transcript|ENST00000554063|retained_intron||n.*1252C>T|||||1252|,A|downstream_gene_variant|MODIFIER|HAUS4|ENSG00000092036|transcript|ENST00000555040|protein_coding||c.*1544C>T|||||1544|WARNING_TRANSCRIPT_INCOMPLETE,A|downstream_gene_variant|MODIFIER|HAUS4|ENSG00000092036|transcript|ENST00000556915|protein_coding||c.*1645C>T|||||1645|WARNING_TRANSCRIPT_INCOMPLETE,A|downstream_gene_variant|MODIFIER|HAUS4|ENSG00000092036|transcript|ENST00000554349|processed_transcript||n.*1811C>T|||||1811|,A|downstream_gene_variant|MODIFIER|HAUS4|ENSG00000092036|transcript|ENST00000553794|processed_transcript||n.*1830C>T|||||1830|,A|downstream_gene_variant|MODIFIER|HAUS4|ENSG00000092036|transcript|ENST00000554373|processed_transcript||n.*1863C>T|||||1863|,A|downstream_gene_variant|MODIFIER|HAUS4|ENSG00000092036|transcript|ENST00000554651|processed_transcript||n.*1904C>T|||||1904|,A|downstream_gene_variant|MODIFIER|HAUS4|ENSG00000092036|transcript|ENST00000554516|protein_coding||c.*4257C>T|||||4257|WARNING_TRANSCRIPT_INCOMPLETE,A|downstream_gene_variant|MODIFIER|HAUS4|ENSG00000092036|transcript|ENST00000557591|protein_coding||c.*4338C>T|||||4338|WARNING_TRANSCRIPT_INCOMPLETE,A|intron_variant|MODIFIER|RP11-298I3.1|ENSG00000257285|transcript|ENST00000548322|antisense|2/2|n.159-9073G>A||||||,A|intron_variant|MODIFIER|RP11-298I3.1|ENSG00000257285|transcript|ENST00000548819|antisense|2/2|n.195-8274G>A|||||| |
| RP11-298I3.5 | rs2069540 | 37 | 14 | 23433544 | 23433544 | + | SNP | G | G | A | SRR8586413 | Missense_Mutation | 0/1:173,217:390:99:6525,0,4037 | 391;ANN=A|intron_variant|MODIFIER|RP11-298I3.5|ENSG00000259132|transcript|ENST00000555074|protein_coding|1/4|c.50-16322C>T||||||WARNING_TRANSCRIPT_NO_START_CODON |
| ATP5C1P1-CDKN3 | rs1307289 | 37 | 14 | 54575897 | 54575897 | + | SNP | G | G | A | SRR8586413 | Missense_Mutation | 0/1:13,26:39:99:672,0,332 | 39;ANN=A|intergenic_region|MODIFIER|ATP5C1P1-CDKN3|ENSG00000224004-ENSG00000100526|intergenic_region|ENSG00000224004-ENSG00000100526|||n.54575897G>A|||||| |
| GOSR2 | rs375919215 | 37 | 17 | 45023648 | 45023648 | + | SNP | T | T | A | SRR8586413 | Missense_Mutation | 0/1:19,15:34:99:194,0,344 | 36;ANN=A|intron_variant|MODIFIER|GOSR2|ENSG00000108433|transcript|ENST00000439730|protein_coding|6/8|c.583+7578T>A||||||,A|intron_variant|MODIFIER|RP11-156P1.2|ENSG00000262633|transcript|ENST00000571841|nonsense_mediated_decay|6/9|c.583+7578T>A||||||,A|intron_variant|MODIFIER|GOSR2|ENSG00000108433|transcript|ENST00000572403|protein_coding|6/6|c.388+7578T>A||||||WARNING_TRANSCRIPT_INCOMPLETE,A|intron_variant|MODIFIER|GOSR2|ENSG00000108433|transcript|ENST00000573224|protein_coding|1/1|c.106+7578T>A||||||WARNING_TRANSCRIPT_NO_START_CODON |
| DNAH17 | rs60002062 | 37 | 17 | 76563725 | 76563725 | + | DEL | GT | GT | G | SRR8586413 | Frame_Shift_Del | 0/1:25,74:99:99:1767,0,400 | 103;ANN=G|intron_variant|MODIFIER|DNAH17|ENSG00000187775|transcript|ENST00000389840|protein_coding|9/80|c.1285-478delA||||||,G|intron_variant|MODIFIER|DNAH17|ENSG00000187775|transcript|ENST00000585328|protein_coding|9/80|c.1285-478delA||||||,G|intron_variant|MODIFIER|DNAH17|ENSG00000187775|transcript|ENST00000589793|retained_intron|5/12|n.497-478delA|||||| |
| PDE11A | rs146313925 | 37 | 2 | 178662395 | 178662395 | + | SNP | T | T | C | SRR8586413 | Missense_Mutation | 0/1:230,54:284:99:725,0,5972 | 284;ANN=C|intron_variant|MODIFIER|PDE11A|ENSG00000128655|transcript|ENST00000286063|protein_coding|9/19|c.1737+19161A>G||||||,C|intron_variant|MODIFIER|PDE11A|ENSG00000128655|transcript|ENST00000358450|protein_coding|10/20|c.987+19161A>G||||||,C|intron_variant|MODIFIER|PDE11A|ENSG00000128655|transcript|ENST00000409504|protein_coding|8/19|c.663+19161A>G||||||,C|intron_variant|MODIFIER|PDE11A|ENSG00000128655|transcript|ENST00000389683|protein_coding|6/16|c.405+19161A>G||||||,C|intron_variant|MODIFIER|PDE11A|ENSG00000128655|transcript|ENST00000449286|protein_coding|8/18|c.663+19161A>G||||||,C|intron_variant|MODIFIER|PDE11A|ENSG00000128655|transcript|ENST00000433879|protein_coding|6/13|c.558+20190A>G||||||WARNING_TRANSCRIPT_NO_START_CODON,C|intron_variant|MODIFIER|PDE11A|ENSG00000128655|transcript|ENST00000497003|processed_transcript|8/14|n.779+19161A>G||||||,C|intron_variant|MODIFIER|PDE11A|ENSG00000128655|transcript|ENST00000492761|processed_transcript|6/6|n.594-448A>G|||||| |
| PDE11A | rs12994774 | 37 | 2 | 178663311 | 178663311 | + | SNP | A | A | C | SRR8586413 | Missense_Mutation | 0/1:265,81:346:99:1738,0,7566 | 347;ANN=C|intron_variant|MODIFIER|PDE11A|ENSG00000128655|transcript|ENST00000286063|protein_coding|9/19|c.1737+18245T>G||||||,C|intron_variant|MODIFIER|PDE11A|ENSG00000128655|transcript|ENST00000358450|protein_coding|10/20|c.987+18245T>G||||||,C|intron_variant|MODIFIER|PDE11A|ENSG00000128655|transcript|ENST00000409504|protein_coding|8/19|c.663+18245T>G||||||,C|intron_variant|MODIFIER|PDE11A|ENSG00000128655|transcript|ENST00000389683|protein_coding|6/16|c.405+18245T>G||||||,C|intron_variant|MODIFIER|PDE11A|ENSG00000128655|transcript|ENST00000449286|protein_coding|8/18|c.663+18245T>G||||||,C|intron_variant|MODIFIER|PDE11A|ENSG00000128655|transcript|ENST00000433879|protein_coding|6/13|c.558+19274T>G||||||WARNING_TRANSCRIPT_NO_START_CODON,C|intron_variant|MODIFIER|PDE11A|ENSG00000128655|transcript|ENST00000497003|processed_transcript|8/14|n.779+18245T>G||||||,C|intron_variant|MODIFIER|PDE11A|ENSG00000128655|transcript|ENST00000492761|processed_transcript|6/6|n.594-1364T>G|||||| |
| PDE11A | rs12998857 | 37 | 2 | 178668882 | 178668882 | + | SNP | A | A | C | SRR8586413 | Missense_Mutation | 0/1:140,59:199:99:919,0,5122 | 203;ANN=C|intron_variant|MODIFIER|PDE11A|ENSG00000128655|transcript|ENST00000286063|protein_coding|9/19|c.1737+12674T>G||||||,C|intron_variant|MODIFIER|PDE11A|ENSG00000128655|transcript|ENST00000358450|protein_coding|10/20|c.987+12674T>G||||||,C|intron_variant|MODIFIER|PDE11A|ENSG00000128655|transcript|ENST00000409504|protein_coding|8/19|c.663+12674T>G||||||,C|intron_variant|MODIFIER|PDE11A|ENSG00000128655|transcript|ENST00000389683|protein_coding|6/16|c.405+12674T>G||||||,C|intron_variant|MODIFIER|PDE11A|ENSG00000128655|transcript|ENST00000449286|protein_coding|8/18|c.663+12674T>G||||||,C|intron_variant|MODIFIER|PDE11A|ENSG00000128655|transcript|ENST00000433879|protein_coding|6/13|c.558+13703T>G||||||WARNING_TRANSCRIPT_NO_START_CODON,C|intron_variant|MODIFIER|PDE11A|ENSG00000128655|transcript|ENST00000497003|processed_transcript|8/14|n.779+12674T>G||||||,C|intron_variant|MODIFIER|PDE11A|ENSG00000128655|transcript|ENST00000492761|processed_transcript|6/6|n.594-6935T>G|||||| |
|  | rs538653835 | 37 | 20 | 63488235 | 63488235 | + | DEL | GC | G | G | SRR8586413 | Frame_Shift_Del | 1/1:4,374:378:99:16794,1077,0 | 396;ANN=G||MODIFIER|||||||||||||ERROR_OUT_OF_OMOSOME_RANGE |
| AC124944.5 | rs369497400 | 37 | 3 | 195568833 | 195568833 | + | SNP | G | G | GC | SRR8586413 | Missense_Mutation | 0/1:98,151:249:99:3968,0,3465 | 249;ANN=GC|upstream_gene_variant|MODIFIER|AC124944.5|ENSG00000223783|transcript|ENST00000413586|lincRNA||n.-4918_-4917insG|||||4918|,GC|upstream_gene_variant|MODIFIER|AC124944.4|ENSG00000235836|transcript|ENST00000454055|processed_pseudogene||n.-2024_-2023insC|||||2023|,GC|intron_variant|MODIFIER|AC124944.5|ENSG00000223783|transcript|ENST00000444346|lincRNA|1/1|n.97-5348_97-5347insG||||||,GC|intron_variant|MODIFIER|AC124944.5|ENSG00000223783|transcript|ENST00000429834|lincRNA|1/1|n.133-5348_133-5347insG|||||| |
| GRIA2 | rs566305935 | 37 | 4 | 158171636 | 158171636 | + | SNP | A | A | G | SRR8586413 | Missense_Mutation | 0/1:13,46:59:99:2060,0,431 | 61;ANN=G|intron_variant|MODIFIER|GRIA2|ENSG00000120251|transcript|ENST00000264426|protein_coding|2/15|c.229+28677A>G||||||,G|intron_variant|MODIFIER|GRIA2|ENSG00000120251|transcript|ENST00000504801|processed_transcript|1/1|n.254+46049A>G||||||,G|intron_variant|MODIFIER|GRIA2|ENSG00000120251|transcript|ENST00000507898|protein_coding|2/15|c.88+28677A>G||||||,G|intron_variant|MODIFIER|GRIA2|ENSG00000120251|transcript|ENST00000393815|protein_coding|2/15|c.88+28677A>G||||||,G|intron_variant|MODIFIER|GRIA2|ENSG00000120251|transcript|ENST00000509417|protein_coding|3/3|c.229+28677A>G||||||WARNING_TRANSCRIPT_INCOMPLETE,G|intron_variant|MODIFIER|GRIA2|ENSG00000120251|transcript|ENST00000296526|protein_coding|2/15|c.229+28677A>G||||||,G|intron_variant|MODIFIER|GRIA2|ENSG00000120251|transcript|ENST00000471736|retained_intron|2/14|n.548+28677A>G||||||,G|intron_variant|MODIFIER|GRIA2|ENSG00000120251|transcript|ENST00000506284|protein_coding|2/2|c.88+28677A>G||||||WARNING_TRANSCRIPT_NO_STOP_CODON,G|intron_variant|MODIFIER|GRIA2|ENSG00000120251|transcript|ENST00000323661|nonsense_mediated_decay|2/16|c.88+28677A>G||||||,G|intron_variant|MODIFIER|GRIA2|ENSG00000120251|transcript|ENST00000505888|protein_coding|1/2|c.88+28677A>G||||||WARNING_TRANSCRIPT_INCOMPLETE,G|intron_variant|MODIFIER|GRIA2|ENSG00000120251|transcript|ENST00000449365|protein_coding|1/14|c.88+28677A>G||||||,G|intron_variant|MODIFIER|GRIA2|ENSG00000120251|transcript|ENST00000503437|protein_coding|2/4|c.-153+631A>G||||||WARNING_TRANSCRIPT_INCOMPLETE |
| SLC1A3 | rs71604837 | 37 | 5 | 36636045 | 36636045 | + | SNP | T | T | TTG | SRR8586413 | Missense_Mutation | 0/1:12,22:34:99:571,0,352 | 55;ANN=TTG|intron_variant|MODIFIER|SLC1A3|ENSG00000079215|transcript|ENST00000265113|protein_coding|3/9|c.319+6355_319+6356dupTG||||||,TTG|intron_variant|MODIFIER|SLC1A3|ENSG00000079215|transcript|ENST00000381918|protein_coding|2/7|c.319+6355_319+6356dupTG||||||,TTG|intron_variant|MODIFIER|SLC1A3|ENSG00000079215|transcript|ENST00000514563|processed_transcript|2/2|n.384+6355_384+6356dupTG||||||,TTG|intron_variant|MODIFIER|SLC1A3|ENSG00000079215|transcript|ENST00000509272|processed_transcript|2/2|n.339+6355_339+6356dupTG|||||| |
| SLC1A3 | rs145798494 | 37 | 5 | 36649326 | 36649326 | + | DEL | CAACAAAAGCAAAACTCCATCTCAAAAAAAAAAAAA | CAACAAAAGCAAAACTCCATCTCAAAAAAAAAAAAA | C | SRR8586413 | In_Frame_Del | 0/1:19,7:26:99:233,0,753 | 26;ANN=C|intron_variant|MODIFIER|SLC1A3|ENSG00000079215|transcript|ENST00000265113|protein_coding|3/9|c.319+19638_319+19672delAACAAAAGCAAAACTCCATCTCAAAAAAAAAAAAA||||||,C|intron_variant|MODIFIER|SLC1A3|ENSG00000079215|transcript|ENST00000381918|protein_coding|2/7|c.319+19638_319+19672delAACAAAAGCAAAACTCCATCTCAAAAAAAAAAAAA||||||,C|intron_variant|MODIFIER|SLC1A3|ENSG00000079215|transcript|ENST00000514563|processed_transcript|2/2|n.384+19638_384+19672delAACAAAAGCAAAACTCCATCTCAAAAAAAAAAAAA||||||,C|intron_variant|MODIFIER|SLC1A3|ENSG00000079215|transcript|ENST00000509272|processed_transcript|2/2|n.339+19638_339+19672delAACAAAAGCAAAACTCCATCTCAAAAAAAAAAAAA|||||| |
| UPK3B | rs537392004 | 37 | 7 | 76304269 | 76304269 | + | DEL | CAAAT | CAAAT | C | SRR8586413 | In_Frame_Del | 0/1:215,213:428:99:8156,0,12299 | 438;ANN=C|intron_variant|MODIFIER|UPK3B|ENSG00000243566|transcript|ENST00000419923|protein_coding|5/5|c.*220+28149_*220+28152delAAAT||||||,C|intron_variant|MODIFIER|UPK3B|ENSG00000243566|transcript|ENST00000443097|protein_coding|7/7|c.*267+28149_*267+28152delAAAT|||||| |
| SEMA3C-AC005008.2 | rs12706950 | 37 | 7 | 80678186 | 80678186 | + | SNP | G | G | A | SRR8586413 | Missense_Mutation | 0/1:125,86:211:99:2398,0,3169 | 211;ANN=A|intergenic_region|MODIFIER|SEMA3C-AC005008.2|ENSG00000075223-ENSG00000237896|intergenic_region|ENSG00000075223-ENSG00000237896|||n.80678186G>A|||||| |
| EXOC4 | rs12673323 | 37 | 7 | 133034703 | 133034703 | + | SNP | T | T | G | SRR8586413 | Missense_Mutation | 0/1:11,35:46:99:836,0,162 | 56;ANN=G|intron_variant|MODIFIER|EXOC4|ENSG00000131558|transcript|ENST00000253861|protein_coding|5/17|c.764-6381T>G||||||,G|intron_variant|MODIFIER|EXOC4|ENSG00000131558|transcript|ENST00000393161|protein_coding|5/9|c.764-6381T>G||||||,G|intron_variant|MODIFIER|EXOC4|ENSG00000131558|transcript|ENST00000486013|retained_intron|5/9|n.793-6381T>G||||||,G|intron_variant|MODIFIER|EXOC4|ENSG00000131558|transcript|ENST00000462055|retained_intron|5/8|n.771-6381T>G||||||,G|intron_variant|MODIFIER|EXOC4|ENSG00000131558|transcript|ENST00000539845|protein_coding|5/17|c.461-6381T>G|||||| |
| MYOM2 | rs2294062 | 37 | 8 | 2090052 | 2090052 | + | SNP | G | A | A | SRR8586413 | Missense_Mutation | 1/1:12,215:227:99:7227,313,0 | 228;ANN=A|upstream_gene_variant|MODIFIER|MYOM2|ENSG00000036448|transcript|ENST00000518513|retained_intron||n.-1221G>A|||||1221|,A|downstream_gene_variant|MODIFIER|MYOM2|ENSG00000036448|transcript|ENST00000520072|processed_transcript||n.*1311G>A|||||1311|,A|downstream_gene_variant|MODIFIER|MYOM2|ENSG00000036448|transcript|ENST00000519631|processed_transcript||n.*1303G>A|||||1303|,A|intron_variant|MODIFIER|MYOM2|ENSG00000036448|transcript|ENST00000262113|protein_coding|34/36|c.4002-248G>A||||||,A|intron_variant|MODIFIER|MYOM2|ENSG00000036448|transcript|ENST00000523438|protein_coding|21/23|c.2277-248G>A||||||,A|intron_variant|MODIFIER|MYOM2|ENSG00000036448|transcript|ENST00000523595|processed_transcript|4/6|n.455-248G>A||||||,A|intron_variant|MODIFIER|MYOM2|ENSG00000036448|transcript|ENST00000519268|processed_transcript|3/5|n.356-248G>A||||||,A|intron_variant|MODIFIER|MYOM2|ENSG00000036448|transcript|ENST00000520298|processed_transcript|1/2|n.144+1243G>A||||||,A|intron_variant|MODIFIER|MYOM2|ENSG00000036448|transcript|ENST00000520779|processed_transcript|2/4|n.76-248G>A|||||| |
| MYOM2 | rs2294071 | 37 | 8 | 2102729 | 2102729 | + | SNP | C | C | T | SRR8586413 | Missense_Mutation | 0/1:299,135:434:99:3661,0,7859 | 434;ANN=T|intron_variant|MODIFIER|MYOM2|ENSG00000036448|transcript|ENST00000520779|processed_transcript|4/4|n.155-10347C>T|||||| |
| CTD-2547L16.2 | rs405308 | 37 | 8 | 18057290 | 18057290 | + | SNP | G | G | A | SRR8586413 | Nonsense_Mutation | 0/1:348,185:533:99:4411,0,8821 | 534;ANN=A|downstream_gene_variant|MODIFIER|CTD-2547L16.2|ENSG00000254015|transcript|ENST00000518144|processed_pseudogene||n.*1143C>T|||||1143|,A|intron_variant|MODIFIER|NAT1|ENSG00000171428|transcript|ENST00000517441|processed_transcript|2/4|n.93-10000G>A||||||,A|intron_variant|MODIFIER|NAT1|ENSG00000171428|transcript|ENST00000535084|protein_coding|1/3|c.-260-10000G>A|||||| |
| CTD-2547L16.2 | rs574114 | 37 | 8 | 18057350 | 18057350 | + | SNP | A | A | T | SRR8586413 | Nonsense_Mutation | 0/1:281,50:331:99:1178,0,11524 | 338;ANN=T|downstream_gene_variant|MODIFIER|CTD-2547L16.2|ENSG00000254015|transcript|ENST00000518144|processed_pseudogene||n.*1083T>A|||||1083|,T|intron_variant|MODIFIER|NAT1|ENSG00000171428|transcript|ENST00000517441|processed_transcript|2/4|n.93-9940A>T||||||,T|intron_variant|MODIFIER|NAT1|ENSG00000171428|transcript|ENST00000535084|protein_coding|1/3|c.-260-9940A>T|||||| |
| CTD-2547L16.2 | rs417661 | 37 | 8 | 18057410 | 18057410 | + | SNP | A | A | T | SRR8586413 | Nonsense_Mutation | 0/1:289,151:440:99:3953,0,8065 | 447;ANN=T|downstream_gene_variant|MODIFIER|CTD-2547L16.2|ENSG00000254015|transcript|ENST00000518144|processed_pseudogene||n.*1023T>A|||||1023|,T|intron_variant|MODIFIER|NAT1|ENSG00000171428|transcript|ENST00000517441|processed_transcript|2/4|n.93-9880A>T||||||,T|intron_variant|MODIFIER|NAT1|ENSG00000171428|transcript|ENST00000535084|protein_coding|1/3|c.-260-9880A>T|||||| |
| RP5-857K21.4 | rs7518996 | 37 | 1 | 631862 | 631862 | + | SNP | G | G | A | SRR8586414 | Missense_Mutation | 0/1:42,290:332:3:8687,0,3 | 336;ANN=A|intron_variant|MODIFIER|RP5-857K21.4|ENSG00000230021|transcript|ENST00000440200|lincRNA|1/2|n.169+23550C>T|||||| |
| ZNF683-LIN28A | rs752085945 | 37 | 1 | 26729854 | 26729854 | + | SNP | T | T | C | SRR8586414 | Missense_Mutation | 0/1:24,12:36:99:411,0,1163 | 36;ANN=C|intergenic_region|MODIFIER|ZNF683-LIN28A|ENSG00000176083-ENSG00000131914|intergenic_region|ENSG00000176083-ENSG00000131914|||n.26729854T>C|||||| |
| FOXJ3 | rs1570433 | 37 | 1 | 42779865 | 42779865 | + | SNP | C | C | T | SRR8586414 | Missense_Mutation | 0/1:10,8:18:99:248,0,282 | 18;ANN=T|intron_variant|MODIFIER|FOXJ3|ENSG00000198815|transcript|ENST00000361346|protein_coding|1/12|c.-17-3084G>A||||||,T|intron_variant|MODIFIER|FOXJ3|ENSG00000198815|transcript|ENST00000372572|protein_coding|3/14|c.-18+1152G>A||||||,T|intron_variant|MODIFIER|FOXJ3|ENSG00000198815|transcript|ENST00000372573|protein_coding|1/12|c.-17-3084G>A||||||,T|intron_variant|MODIFIER|FOXJ3|ENSG00000198815|transcript|ENST00000361776|protein_coding|1/11|c.-17-3084G>A||||||,T|intron_variant|MODIFIER|FOXJ3|ENSG00000198815|transcript|ENST00000545068|protein_coding|1/12|c.-18+1152G>A||||||,T|intron_variant|MODIFIER|FOXJ3|ENSG00000198815|transcript|ENST00000445886|protein_coding|1/7|c.-17-3084G>A||||||WARNING_TRANSCRIPT_NO_STOP_CODON |
| NOTCH2 | rs1699761 | 37 | 1 | 120489416 | 120489416 | + | SNP | C | C | A | SRR8586414 | Nonsense_Mutation | 0/1:160,81:241:99:2006,0,3956 | 242;ANN=A|downstream_gene_variant|MODIFIER|NOTCH2|ENSG00000134250|transcript|ENST00000479412|retained_intron||n.*3327G>T|||||3327|,A|intron_variant|MODIFIER|NOTCH2|ENSG00000134250|transcript|ENST00000256646|protein_coding|17/33|c.2752+1621G>T|||||| |
| NOTCH2 | rs113514538 | 37 | 1 | 120489584 | 120489584 | + | SNP | T | T | G | SRR8586414 | Nonsense_Mutation | 0/1:100,36:136:99:757,0,2981 | 136;ANN=G|downstream_gene_variant|MODIFIER|NOTCH2|ENSG00000134250|transcript|ENST00000479412|retained_intron||n.*3159A>C|||||3159|,G|intron_variant|MODIFIER|NOTCH2|ENSG00000134250|transcript|ENST00000256646|protein_coding|17/33|c.2752+1453A>C|||||| |
| NOTCH2 | rs1698582 | 37 | 1 | 120493284 | 120493284 | + | SNP | G | G | A | SRR8586414 | Missense_Mutation | 0/1:108,96:204:99:2698,0,3133 | 204;ANN=A|intron_variant|MODIFIER|NOTCH2|ENSG00000134250|transcript|ENST00000256646|protein_coding|15/33|c.2479+63C>T||||||,A|non_coding_transcript_exon_variant|MODIFIER|NOTCH2|ENSG00000134250|transcript|ENST00000479412|retained_intron|14/14|n.2680C>T||||||WARNING_REF_DOES_NOT_MATCH_GENOME |
| RP11-763B22.9 | rs1664022 | 37 | 1 | 148889827 | 148889827 | + | SNP | G | G | T | SRR8586414 | Missense_Mutation | 0/1:304,64:368:99:1194,0,8405 | 368;ANN=T|intron_variant|MODIFIER|RP11-763B22.9|ENSG00000231448|transcript|ENST00000444424|unprocessed_pseudogene|8/9|n.1017+138G>T|||||| |
| DRD5P2 | rs1778581 | 37 | 1 | 148903195 | 148903195 | + | SNP | C | C | T | SRR8586414 | Nonsense_Mutation | 0/1:40,15:55:99:386,0,1134 | 55;ANN=T|downstream_gene_variant|MODIFIER|DRD5P2|ENSG00000175658|transcript|ENST00000535197|processed_pseudogene||n.*92C>T|||||92|,T|downstream_gene_variant|MODIFIER|DRD5P2|ENSG00000175658|transcript|ENST00000309892|processed_pseudogene||n.*282C>T|||||282|,T|non_coding_transcript_exon_variant|MODIFIER|DRD5P2|ENSG00000175658|transcript|ENST00000421395|processed_pseudogene|2/2|n.1492C>T|||||| |
| RP11-14N7.2 | rs3124680 | 37 | 1 | 148933084 | 148933084 | + | SNP | T | T | A | SRR8586414 | Nonsense_Mutation | 0/1:53,79:132:99:2362,0,1171 | 132;ANN=A|downstream_gene_variant|MODIFIER|RP11-14N7.2|ENSG00000232527|transcript|ENST00000420597|lincRNA||n.*164T>A|||||164|,A|intron_variant|MODIFIER|RP11-14N7.2|ENSG00000232527|transcript|ENST00000539543|lincRNA|2/3|n.176+164T>A||||||,A|intron_variant|MODIFIER|RP11-14N7.2|ENSG00000232527|transcript|ENST00000452399|lincRNA|2/2|n.199+164T>A||||||,A|intron_variant|MODIFIER|RP11-14N7.2|ENSG00000232527|transcript|ENST00000294715|lincRNA|2/2|n.190+164T>A||||||,A|intron_variant|MODIFIER|RP11-14N7.2|ENSG00000232527|transcript|ENST00000457390|lincRNA|1/1|n.136+164T>A|||||| |
| RP11-14N7.2 | rs512570 | 37 | 1 | 148933778 | 148933778 | + | SNP | A | A | G | SRR8586414 | Nonsense_Mutation | 0/1:34,67:101:99:1876,0,799 | 101;ANN=G|downstream_gene_variant|MODIFIER|RP11-14N7.2|ENSG00000232527|transcript|ENST00000420597|lincRNA||n.*858A>G|||||858|,G|downstream_gene_variant|MODIFIER|RP11-14N7.2|ENSG00000232527|transcript|ENST00000294715|lincRNA||n.*410A>G|||||410|,G|downstream_gene_variant|MODIFIER|RP11-14N7.2|ENSG00000232527|transcript|ENST00000457390|lincRNA||n.*199A>G|||||199|,G|intron_variant|MODIFIER|RP11-14N7.2|ENSG00000232527|transcript|ENST00000539543|lincRNA|3/3|n.254+410A>G||||||,G|intron_variant|MODIFIER|RP11-14N7.2|ENSG00000232527|transcript|ENST00000452399|lincRNA|2/2|n.199+858A>G|||||| |
| RNVU1-17 | rs75500033 | 37 | 1 | 149197768 | 149197768 | + | SNP | C | C | T | SRR8586414 | Missense_Mutation | 0/1:1197,228:1425:99:3838,0,34005 | 1426;ANN=T|upstream_gene_variant|MODIFIER|RNVU1-17|ENSG00000207349|transcript|ENST00000384619|snRNA||n.-3499G>A|||||3499|,T|intergenic_region|MODIFIER|RNVU1-17-RNU1-92P|ENSG00000207349-ENSG00000252826|intergenic_region|ENSG00000207349-ENSG00000252826|||n.149197768C>T|||||| |
| RP11-385F5.5 | rs71559972 | 37 | 1 | 236695563 | 236695563 | + | INS | T | T | TC | SRR8586414 | Frame_Shift_Ins | 0/1:56,16:72:99:232,0,3103 | 84;ANN=TC|downstream_gene_variant|MODIFIER|RP11-385F5.5|ENSG00000273058|transcript|ENST00000608547|antisense||n.*3909_*3910insG|||||3909|,TC|intron_variant|MODIFIER|LGALS8|ENSG00000116977|transcript|ENST00000352231|protein_coding|3/11|c.46-5234dupC||||||,TC|intron_variant|MODIFIER|LGALS8|ENSG00000116977|transcript|ENST00000481485|protein_coding|3/4|c.46-5234dupC||||||WARNING_TRANSCRIPT_INCOMPLETE,TC|intron_variant|MODIFIER|LGALS8|ENSG00000116977|transcript|ENST00000454943|protein_coding|4/7|c.46-5234dupC||||||WARNING_TRANSCRIPT_INCOMPLETE,TC|intron_variant|MODIFIER|LGALS8|ENSG00000116977|transcript|ENST00000527974|protein_coding|2/10|c.46-5234dupC||||||,TC|intron_variant|MODIFIER|LGALS8|ENSG00000116977|transcript|ENST00000430527|protein_coding|3/5|c.46-5234dupC||||||WARNING_TRANSCRIPT_INCOMPLETE,TC|intron_variant|MODIFIER|LGALS8|ENSG00000116977|transcript|ENST00000406509|protein_coding|5/10|c.46-5234dupC||||||WARNING_TRANSCRIPT_INCOMPLETE,TC|intron_variant|MODIFIER|LGALS8|ENSG00000116977|transcript|ENST00000526589|protein_coding|5/13|c.46-5234dupC||||||,TC|intron_variant|MODIFIER|LGALS8|ENSG00000116977|transcript|ENST00000529489|protein_coding|3/3|c.46-5234dupC||||||WARNING_TRANSCRIPT_NO_STOP_CODON,TC|intron_variant|MODIFIER|LGALS8|ENSG00000116977|transcript|ENST00000341872|protein_coding|3/10|c.46-5234dupC||||||,TC|intron_variant|MODIFIER|LGALS8|ENSG00000116977|transcript|ENST00000450372|protein_coding|3/11|c.46-5234dupC||||||,TC|intron_variant|MODIFIER|LGALS8|ENSG00000116977|transcript|ENST00000366584|protein_coding|2/9|c.46-5234dupC||||||,TC|intron_variant|MODIFIER|LGALS8|ENSG00000116977|transcript|ENST00000238181|protein_coding|2/6|c.46-5234dupC||||||WARNING_TRANSCRIPT_INCOMPLETE,TC|intron_variant|MODIFIER|LGALS8|ENSG00000116977|transcript|ENST00000532826|retained_intron|2/3|n.226-5234dupC||||||,TC|intron_variant|MODIFIER|LGALS8|ENSG00000116977|transcript|ENST00000528782|retained_intron|2/4|n.226-5234dupC||||||,TC|intron_variant|MODIFIER|LGALS8|ENSG00000116977|transcript|ENST00000366583|retained_intron|2/6|n.222-5234dupC||||||,TC|intron_variant|MODIFIER|LGALS8|ENSG00000116977|transcript|ENST00000442397|nonsense_mediated_decay|2/4|c.46-5234dupC||||||,TC|intron_variant|MODIFIER|LGALS8|ENSG00000116977|transcript|ENST00000434231|nonsense_mediated_decay|2/4|c.45+6148dupC||||||,TC|intron_variant|MODIFIER|LGALS8|ENSG00000116977|transcript|ENST00000416919|protein_coding|2/8|c.46-5234dupC||||||,TC|intron_variant|MODIFIER|LGALS8|ENSG00000116977|transcript|ENST00000323938|protein_coding|2/9|c.46-5234dupC||||||,TC|intron_variant|MODIFIER|LGALS8|ENSG00000116977|transcript|ENST00000526634|protein_coding|2/9|c.46-5234dupC||||||,TC|intron_variant|MODIFIER|LGALS8|ENSG00000116977|transcript|ENST00000525042|protein_coding|1/7|c.46-5234dupC|||||| |
| MTR-RPL35P1 | rs56114377 | 37 | 1 | 237080608 | 237080608 | + | SNP | G | G | A | SRR8586414 | Missense_Mutation | 0/1:7,40:47:99:1645,0,314 | 48;ANN=A|intergenic_region|MODIFIER|MTR-RPL35P1|ENSG00000116984-ENSG00000237991|intergenic_region|ENSG00000116984-ENSG00000237991|||n.237080608G>A|||||| |
| RYR2 | rs115898570 | 37 | 1 | 237345474 | 237345474 | + | SNP | T | T | A | SRR8586414 | Missense_Mutation | 0/1:51,22:73:99:616,0,2632 | 73;ANN=A|intron_variant|MODIFIER|RYR2|ENSG00000198626|transcript|ENST00000366574|protein_coding|1/104|c.49-88323T>A|||||| |
| RYR2 | rs763305618 | 37 | 1 | 237700464 | 237700464 | + | SNP | A | A | C | SRR8586414 | Missense_Mutation | 0/1:139,155:294:99:5497,0,4416 | 295;ANN=C|intron_variant|MODIFIER|RYR2|ENSG00000198626|transcript|ENST00000360064|protein_coding|26/106|c.2900+6654A>C||||||,C|intron_variant|MODIFIER|RYR2|ENSG00000198626|transcript|ENST00000366574|protein_coding|25/104|c.2906+6654A>C||||||,C|intron_variant|MODIFIER|RYR2|ENSG00000198626|transcript|ENST00000542537|protein_coding|24/103|c.2858+6654A>C||||||WARNING_TRANSCRIPT_NO_START_CODON |
| RP11-492M23.2-LYZL1 | rs71489679 | 37 | 10 | 29501462 | 29501462 | + | SNP | A | A | G | SRR8586414 | Missense_Mutation | 0/1:53,31:84:99:780,0,1378 | 84;ANN=G|intergenic_region|MODIFIER|RP11-492M23.2-LYZL1|ENSG00000229605-ENSG00000120563|intergenic_region|ENSG00000229605-ENSG00000120563|||n.29501462A>G|||||| |
| RP11-492M23.2-LYZL1 | rs1547169 | 37 | 10 | 29554904 | 29554904 | + | SNP | C | C | T | SRR8586414 | Missense_Mutation | 0/1:53,81:134:99:2492,0,1259 | 137;ANN=T|intergenic_region|MODIFIER|RP11-492M23.2-LYZL1|ENSG00000229605-ENSG00000120563|intergenic_region|ENSG00000229605-ENSG00000120563|||n.29554904C>T|||||| |
| IFITM1 | rs748890801 | 37 | 11 | 314309 | 314309 | + | SNP | T | T | A | SRR8586414 | Missense_Mutation | 0/1:309,25:334:99:201,0,13323 | 334;ANN=A|missense_variant|MODERATE|IFITM1|ENSG00000185885|transcript|ENST00000328221|protein_coding|2/3|c.139T>A|p.Leu47Met|478/844|139/378|47/125||,A|missense_variant|MODERATE|IFITM1|ENSG00000185885|transcript|ENST00000528780|protein_coding|2/3|c.139T>A|p.Leu47Met|332/730|139/378|47/125||,A|missense_variant|MODERATE|IFITM1|ENSG00000185885|transcript|ENST00000408968|protein_coding|1/2|c.139T>A|p.Leu47Met|457/855|139/378|47/125||,A|upstream_gene_variant|MODIFIER|RP11-326C3.7|ENSG00000254910|transcript|ENST00000526612|antisense||n.-3168A>T|||||3168|,A|upstream_gene_variant|MODIFIER|RP11-326C3.11|ENSG00000251661|transcript|ENST00000602429|antisense||n.-4344T>A|||||4344|,A|upstream_gene_variant|MODIFIER|IFITM1|ENSG00000185885|transcript|ENST00000525554|processed_transcript||n.-109T>A|||||109|,A|upstream_gene_variant|MODIFIER|RP11-326C3.11|ENSG00000251661|transcript|ENST00000508004|antisense||n.-4331T>A|||||4331|,A|upstream_gene_variant|MODIFIER|RP11-326C3.11|ENSG00000251661|transcript|ENST00000602756|antisense||n.-4363T>A|||||4363|,A|downstream_gene_variant|MODIFIER|IFITM2|ENSG00000185201|transcript|ENST00000399817|protein_coding||c.*5144T>A|||||4914|,A|downstream_gene_variant|MODIFIER|IFITM2|ENSG00000185201|transcript|ENST00000602569|protein_coding||c.*5144T>A|||||4914|,A|downstream_gene_variant|MODIFIER|IFITM2|ENSG00000185201|transcript|ENST00000527146|nonsense_mediated_decay||c.*5507T>A|||||4917|WARNING_TRANSCRIPT_NO_START_CODON,A|intron_variant|MODIFIER|IFITM2|ENSG00000185201|transcript|ENST00000399815|protein_coding|1/1|c.31-613T>A||||||WARNING_TRANSCRIPT_NO_START_CODON |
| IFITM1 | rs778527366 | 37 | 11 | 314315 | 314315 | + | SNP | T | T | A | SRR8586414 | Missense_Mutation | 0/1:321,29:350:99:218,0,13748 | 350;ANN=A|missense_variant|MODERATE|IFITM1|ENSG00000185885|transcript|ENST00000328221|protein_coding|2/3|c.145T>A|p.Trp49Arg|484/844|145/378|49/125||,A|missense_variant|MODERATE|IFITM1|ENSG00000185885|transcript|ENST00000528780|protein_coding|2/3|c.145T>A|p.Trp49Arg|338/730|145/378|49/125||,A|missense_variant|MODERATE|IFITM1|ENSG00000185885|transcript|ENST00000408968|protein_coding|1/2|c.145T>A|p.Trp49Arg|463/855|145/378|49/125||,A|upstream_gene_variant|MODIFIER|RP11-326C3.7|ENSG00000254910|transcript|ENST00000526612|antisense||n.-3174A>T|||||3174|,A|upstream_gene_variant|MODIFIER|RP11-326C3.11|ENSG00000251661|transcript|ENST00000602429|antisense||n.-4338T>A|||||4338|,A|upstream_gene_variant|MODIFIER|IFITM1|ENSG00000185885|transcript|ENST00000525554|processed_transcript||n.-103T>A|||||103|,A|upstream_gene_variant|MODIFIER|RP11-326C3.11|ENSG00000251661|transcript|ENST00000508004|antisense||n.-4325T>A|||||4325|,A|upstream_gene_variant|MODIFIER|RP11-326C3.11|ENSG00000251661|transcript|ENST00000602756|antisense||n.-4357T>A|||||4357|,A|downstream_gene_variant|MODIFIER|IFITM2|ENSG00000185201|transcript|ENST00000399817|protein_coding||c.*5150T>A|||||4920|,A|downstream_gene_variant|MODIFIER|IFITM2|ENSG00000185201|transcript|ENST00000602569|protein_coding||c.*5150T>A|||||4920|,A|downstream_gene_variant|MODIFIER|IFITM2|ENSG00000185201|transcript|ENST00000527146|nonsense_mediated_decay||c.*5513T>A|||||4923|WARNING_TRANSCRIPT_NO_START_CODON,A|intron_variant|MODIFIER|IFITM2|ENSG00000185201|transcript|ENST00000399815|protein_coding|1/1|c.31-607T>A||||||WARNING_TRANSCRIPT_NO_START_CODON |
| IFITM1 | rs747717593 | 37 | 11 | 314316 | 314316 | + | SNP | G | G | C | SRR8586414 | Missense_Mutation | 0/1:318,29:347:99:218,0,13759 | 347;ANN=C|missense_variant|MODERATE|IFITM1|ENSG00000185885|transcript|ENST00000328221|protein_coding|2/3|c.146G>C|p.Trp49Ser|485/844|146/378|49/125||,C|missense_variant|MODERATE|IFITM1|ENSG00000185885|transcript|ENST00000528780|protein_coding|2/3|c.146G>C|p.Trp49Ser|339/730|146/378|49/125||,C|missense_variant|MODERATE|IFITM1|ENSG00000185885|transcript|ENST00000408968|protein_coding|1/2|c.146G>C|p.Trp49Ser|464/855|146/378|49/125||,C|upstream_gene_variant|MODIFIER|RP11-326C3.7|ENSG00000254910|transcript|ENST00000526612|antisense||n.-3175C>G|||||3175|,C|upstream_gene_variant|MODIFIER|RP11-326C3.11|ENSG00000251661|transcript|ENST00000602429|antisense||n.-4337G>C|||||4337|,C|upstream_gene_variant|MODIFIER|IFITM1|ENSG00000185885|transcript|ENST00000525554|processed_transcript||n.-102G>C|||||102|,C|upstream_gene_variant|MODIFIER|RP11-326C3.11|ENSG00000251661|transcript|ENST00000508004|antisense||n.-4324G>C|||||4324|,C|upstream_gene_variant|MODIFIER|RP11-326C3.11|ENSG00000251661|transcript|ENST00000602756|antisense||n.-4356G>C|||||4356|,C|downstream_gene_variant|MODIFIER|IFITM2|ENSG00000185201|transcript|ENST00000399817|protein_coding||c.*5151G>C|||||4921|,C|downstream_gene_variant|MODIFIER|IFITM2|ENSG00000185201|transcript|ENST00000602569|protein_coding||c.*5151G>C|||||4921|,C|downstream_gene_variant|MODIFIER|IFITM2|ENSG00000185201|transcript|ENST00000527146|nonsense_mediated_decay||c.*5514G>C|||||4924|WARNING_TRANSCRIPT_NO_START_CODON,C|intron_variant|MODIFIER|IFITM2|ENSG00000185201|transcript|ENST00000399815|protein_coding|1/1|c.31-606G>C||||||WARNING_TRANSCRIPT_NO_START_CODON |
| IFITM1 | rs771883098 | 37 | 11 | 314317 | 314317 | + | SNP | G | G | C | SRR8586414 | Missense_Mutation | 0/1:320,29:349:99:233,0,13614 | 349;ANN=C|missense_variant|MODERATE|IFITM1|ENSG00000185885|transcript|ENST00000328221|protein_coding|2/3|c.147G>C|p.Trp49Cys|486/844|147/378|49/125||,C|missense_variant|MODERATE|IFITM1|ENSG00000185885|transcript|ENST00000528780|protein_coding|2/3|c.147G>C|p.Trp49Cys|340/730|147/378|49/125||,C|missense_variant|MODERATE|IFITM1|ENSG00000185885|transcript|ENST00000408968|protein_coding|1/2|c.147G>C|p.Trp49Cys|465/855|147/378|49/125||,C|upstream_gene_variant|MODIFIER|RP11-326C3.7|ENSG00000254910|transcript|ENST00000526612|antisense||n.-3176C>G|||||3176|,C|upstream_gene_variant|MODIFIER|RP11-326C3.11|ENSG00000251661|transcript|ENST00000602429|antisense||n.-4336G>C|||||4336|,C|upstream_gene_variant|MODIFIER|IFITM1|ENSG00000185885|transcript|ENST00000525554|processed_transcript||n.-101G>C|||||101|,C|upstream_gene_variant|MODIFIER|RP11-326C3.11|ENSG00000251661|transcript|ENST00000508004|antisense||n.-4323G>C|||||4323|,C|upstream_gene_variant|MODIFIER|RP11-326C3.11|ENSG00000251661|transcript|ENST00000602756|antisense||n.-4355G>C|||||4355|,C|downstream_gene_variant|MODIFIER|IFITM2|ENSG00000185201|transcript|ENST00000399817|protein_coding||c.*5152G>C|||||4922|,C|downstream_gene_variant|MODIFIER|IFITM2|ENSG00000185201|transcript|ENST00000602569|protein_coding||c.*5152G>C|||||4922|,C|downstream_gene_variant|MODIFIER|IFITM2|ENSG00000185201|transcript|ENST00000527146|nonsense_mediated_decay||c.*5515G>C|||||4925|WARNING_TRANSCRIPT_NO_START_CODON,C|intron_variant|MODIFIER|IFITM2|ENSG00000185201|transcript|ENST00000399815|protein_coding|1/1|c.31-605G>C||||||WARNING_TRANSCRIPT_NO_START_CODON |
| IFITM1 | rs12432 | 37 | 11 | 314323 | 314323 | + | SNP | T | T | C | SRR8586414 | Nonsense_Mutation | 0/1:297,29:326:99:291,0,12830 | 326;ANN=C|sequence_feature|MODERATE|IFITM1|ENSG00000185885|lipidation-site:S-palmitoyl_cysteine|ENST00000328221|protein_coding|2/3|c.153T>C||||||,C|synonymous_variant|LOW|IFITM1|ENSG00000185885|transcript|ENST00000328221|protein_coding|2/3|c.153T>C|p.Cys51Cys|492/844|153/378|51/125||,C|synonymous_variant|LOW|IFITM1|ENSG00000185885|transcript|ENST00000528780|protein_coding|2/3|c.153T>C|p.Cys51Cys|346/730|153/378|51/125||,C|synonymous_variant|LOW|IFITM1|ENSG00000185885|transcript|ENST00000408968|protein_coding|1/2|c.153T>C|p.Cys51Cys|471/855|153/378|51/125||,C|upstream_gene_variant|MODIFIER|RP11-326C3.7|ENSG00000254910|transcript|ENST00000526612|antisense||n.-3182A>G|||||3182|,C|upstream_gene_variant|MODIFIER|RP11-326C3.11|ENSG00000251661|transcript|ENST00000602429|antisense||n.-4330T>C|||||4330|,C|upstream_gene_variant|MODIFIER|IFITM1|ENSG00000185885|transcript|ENST00000525554|processed_transcript||n.-95T>C|||||95|,C|upstream_gene_variant|MODIFIER|RP11-326C3.11|ENSG00000251661|transcript|ENST00000508004|antisense||n.-4317T>C|||||4317|,C|upstream_gene_variant|MODIFIER|RP11-326C3.11|ENSG00000251661|transcript|ENST00000602756|antisense||n.-4349T>C|||||4349|,C|downstream_gene_variant|MODIFIER|IFITM2|ENSG00000185201|transcript|ENST00000399817|protein_coding||c.*5158T>C|||||4928|,C|downstream_gene_variant|MODIFIER|IFITM2|ENSG00000185201|transcript|ENST00000602569|protein_coding||c.*5158T>C|||||4928|,C|downstream_gene_variant|MODIFIER|IFITM2|ENSG00000185201|transcript|ENST00000527146|nonsense_mediated_decay||c.*5521T>C|||||4931|WARNING_TRANSCRIPT_NO_START_CODON,C|intron_variant|MODIFIER|IFITM2|ENSG00000185201|transcript|ENST00000399815|protein_coding|1/1|c.31-599T>C||||||WARNING_TRANSCRIPT_NO_START_CODON |
| AMBRA1 | rs568863082 | 37 | 11 | 46428617 | 46428617 | + | SNP | A | A | G | SRR8586414 | Missense_Mutation | 0/1:9,91:100:99:2684,0,104 | 101;ANN=G|intron_variant|MODIFIER|AMBRA1|ENSG00000110497|transcript|ENST00000458649|protein_coding|17/17|c.3403+1446T>C||||||,G|intron_variant|MODIFIER|AMBRA1|ENSG00000110497|transcript|ENST00000298834|protein_coding|16/16|c.3223+1446T>C||||||,G|intron_variant|MODIFIER|AMBRA1|ENSG00000110497|transcript|ENST00000314845|protein_coding|18/18|c.3133+1446T>C||||||,G|intron_variant|MODIFIER|AMBRA1|ENSG00000110497|transcript|ENST00000426438|protein_coding|16/16|c.3316+1446T>C||||||,G|intron_variant|MODIFIER|AMBRA1|ENSG00000110497|transcript|ENST00000533727|protein_coding|17/17|c.3046+1446T>C||||||,G|intron_variant|MODIFIER|AMBRA1|ENSG00000110497|transcript|ENST00000534300|protein_coding|16/16|c.3223+1446T>C||||||,G|intron_variant|MODIFIER|AMBRA1|ENSG00000110497|transcript|ENST00000526545|protein_coding|2/2|c.277+1446T>C||||||WARNING_TRANSCRIPT_NO_START_CODON,G|intron_variant|MODIFIER|AMBRA1|ENSG00000110497|transcript|ENST00000528950|protein_coding|16/16|c.3316+1446T>C|||||| |
| MADD | rs397516073 | 37 | 11 | 47347856 | 47347856 | + | SNP | C | C | T | SRR8586414 | Missense_Mutation | 0/1:73,243:316:99:7361,0,1931 | 317;ANN=T|upstream_gene_variant|MODIFIER|MADD|ENSG00000110514|transcript|ENST00000469699|retained_intron||n.-1837C>T|||||1837|,T|downstream_gene_variant|MODIFIER|MADD|ENSG00000110514|transcript|ENST00000405573|protein_coding||c.*1560C>T|||||1270|,T|downstream_gene_variant|MODIFIER|MADD|ENSG00000110514|transcript|ENST00000494403|processed_transcript||n.*1009C>T|||||1009|,T|intron_variant|MODIFIER|MADD|ENSG00000110514|transcript|ENST00000311027|protein_coding|33/35|c.4723-433C>T||||||,T|intron_variant|MODIFIER|MADD|ENSG00000110514|transcript|ENST00000342922|protein_coding|30/32|c.4546-433C>T||||||,T|intron_variant|MODIFIER|MADD|ENSG00000110514|transcript|ENST00000349238|protein_coding|31/33|c.4606-433C>T||||||,T|intron_variant|MODIFIER|MADD|ENSG00000110514|transcript|ENST00000402799|protein_coding|30/32|c.4417-433C>T||||||,T|intron_variant|MODIFIER|MADD|ENSG00000110514|transcript|ENST00000406482|protein_coding|30/31|c.4416+1728C>T||||||,T|intron_variant|MODIFIER|MADD|ENSG00000110514|transcript|ENST00000407859|protein_coding|31/33|c.4477-433C>T||||||,T|intron_variant|MODIFIER|MADD|ENSG00000110514|transcript|ENST00000395344|protein_coding|30/32|c.4405-433C>T||||||,T|intron_variant|MODIFIER|MADD|ENSG00000110514|transcript|ENST00000395336|protein_coding|33/34|c.4722+1728C>T||||||,T|intron_variant|MODIFIER|MADD|ENSG00000110514|transcript|ENST00000402192|protein_coding|30/32|c.4543-433C>T||||||,T|intron_variant|MODIFIER|MADD|ENSG00000110514|transcript|ENST00000460452|retained_intron|3/3|n.346-433C>T|||||| |
| PC | rs774181201 | 37 | 11 | 66639685 | 66639685 | + | DEL | GTT | GTT | G | SRR8586414 | In_Frame_Del | 0/1:15,22:37:99:879,0,5338 | 37;ANN=G|intron_variant|MODIFIER|PC|ENSG00000173599|transcript|ENST00000393955|protein_coding|1/20|c.1-57_1-56delAA||||||,G|intron_variant|MODIFIER|PC|ENSG00000173599|transcript|ENST00000393958|protein_coding|2/21|c.1-57_1-56delAA||||||,G|intron_variant|MODIFIER|PC|ENSG00000173599|transcript|ENST00000393960|protein_coding|3/22|c.1-57_1-56delAA||||||,G|intron_variant|MODIFIER|PC|ENSG00000173599|transcript|ENST00000355677|protein_coding|2/11|c.1-57_1-56delAA||||||,G|intron_variant|MODIFIER|PC|ENSG00000173599|transcript|ENST00000524491|protein_coding|2/11|c.-120-57_-120-56delAA||||||,G|intron_variant|MODIFIER|PC|ENSG00000173599|transcript|ENST00000531614|processed_transcript|2/4|n.203-57_203-56delAA||||||,G|intron_variant|MODIFIER|PC|ENSG00000173599|transcript|ENST00000528403|processed_transcript|3/5|n.277-57_277-56delAA|||||| |
| TSPAN9 | rs58837502 | 37 | 12 | 3211762 | 3211762 | + | SNP | T | T | C | SRR8586414 | Missense_Mutation | 0/1:36,87:123:99:2578,0,832 | 125;ANN=C|intron_variant|MODIFIER|TSPAN9|ENSG00000011105|transcript|ENST00000537971|protein_coding|1/7|c.-18+25143T>C||||||,C|intron_variant|MODIFIER|TSPAN9|ENSG00000011105|transcript|ENST00000444315|nonsense_mediated_decay|2/5|c.-18+18877T>C||||||,C|intron_variant|MODIFIER|TSPAN9|ENSG00000011105|transcript|ENST00000011898|protein_coding|2/8|c.-18+18877T>C|||||| |
| NCOR2 | rs762772073 | 37 | 12 | 124912413 | 124912413 | + | SNP | A | A | G | SRR8586414 | Nonsense_Mutation | 0/1:333,51:384:99:679,0,9896 | 387;ANN=G|downstream_gene_variant|MODIFIER|NCOR2|ENSG00000196498|transcript|ENST00000494460|retained_intron||n.*1528T>C|||||1528|,G|downstream_gene_variant|MODIFIER|NCOR2|ENSG00000196498|transcript|ENST00000542927|protein_coding||c.*2744T>C|||||2743|WARNING_TRANSCRIPT_NO_START_CODON,G|downstream_gene_variant|MODIFIER|NCOR2|ENSG00000196498|transcript|ENST00000448008|protein_coding||c.*2774T>C|||||2774|WARNING_TRANSCRIPT_NO_START_CODON,G|intron_variant|MODIFIER|NCOR2|ENSG00000196498|transcript|ENST00000356219|protein_coding|11/47|c.1150-1067T>C||||||,G|intron_variant|MODIFIER|NCOR2|ENSG00000196498|transcript|ENST00000405201|protein_coding|10/46|c.1150-1067T>C||||||,G|intron_variant|MODIFIER|NCOR2|ENSG00000196498|transcript|ENST00000404621|protein_coding|11/46|c.1147-1067T>C||||||,G|intron_variant|MODIFIER|NCOR2|ENSG00000196498|transcript|ENST00000397355|protein_coding|11/46|c.1150-1067T>C||||||,G|intron_variant|MODIFIER|NCOR2|ENSG00000196498|transcript|ENST00000404121|protein_coding|11/46|c.-141-1067T>C||||||,G|intron_variant|MODIFIER|NCOR2|ENSG00000196498|transcript|ENST00000429285|protein_coding|11/46|c.1147-1067T>C||||||,G|intron_variant|MODIFIER|NCOR2|ENSG00000196498|transcript|ENST00000458234|protein_coding|12/32|c.1150-1067T>C||||||WARNING_TRANSCRIPT_INCOMPLETE,G|intron_variant|MODIFIER|NCOR2|ENSG00000196498|transcript|ENST00000420698|protein_coding|11/13|c.1150-1067T>C||||||WARNING_TRANSCRIPT_INCOMPLETE |
| FREM2 | rs9594332 | 37 | 13 | 39434823 | 39434823 | + | SNP | G | G | A | SRR8586414 | Missense_Mutation | 0/1:12,17:29:99:497,0,281 | 42;ANN=A|intron_variant|MODIFIER|FREM2|ENSG00000150893|transcript|ENST00000280481|protein_coding|14/23|c.7520-745G>A|||||| |
| PRMT5-AS1 | rs753335327 | 37 | 14 | 23384640 | 23384640 | + | SNP | C | C | T | SRR8586414 | Missense_Mutation | 0/1:208,197:405:99:5146,0,9530 | 405;ANN=T|upstream_gene_variant|MODIFIER|PRMT5-AS1|ENSG00000237054|transcript|ENST00000424245|antisense||n.-4025C>T|||||4025|,T|upstream_gene_variant|MODIFIER|RBM23|ENSG00000100461|transcript|ENST00000555691|protein_coding||c.-4038G>A|||||3639|WARNING_TRANSCRIPT_INCOMPLETE,T|upstream_gene_variant|MODIFIER|PRMT5-AS1|ENSG00000237054|transcript|ENST00000609885|antisense||n.-4190C>T|||||4190|,T|downstream_gene_variant|MODIFIER|RBM23|ENSG00000100461|transcript|ENST00000553777|processed_transcript||n.*2133G>A|||||2133|,T|intron_variant|MODIFIER|RBM23|ENSG00000100461|transcript|ENST00000359890|protein_coding|1/13|c.-11+3568G>A||||||,T|intron_variant|MODIFIER|RBM23|ENSG00000100461|transcript|ENST00000555209|protein_coding|1/10|c.-493+3568G>A||||||,T|intron_variant|MODIFIER|RBM23|ENSG00000100461|transcript|ENST00000557667|retained_intron|1/13|n.138+3568G>A||||||,T|intron_variant|MODIFIER|RBM23|ENSG00000100461|transcript|ENST00000399922|protein_coding|1/12|c.-11+3568G>A||||||,T|intron_variant|MODIFIER|RBM23|ENSG00000100461|transcript|ENST00000346528|protein_coding|1/11|c.-11+3568G>A||||||,T|intron_variant|MODIFIER|RBM23|ENSG00000100461|transcript|ENST00000542016|protein_coding|1/11|c.-360+3568G>A||||||,T|intron_variant|MODIFIER|RBM23|ENSG00000100461|transcript|ENST00000307814|retained_intron|1/10|n.151+3568G>A||||||,T|intron_variant|MODIFIER|RBM23|ENSG00000100461|transcript|ENST00000557403|protein_coding|1/5|c.-343+3568G>A||||||WARNING_TRANSCRIPT_INCOMPLETE,T|intron_variant|MODIFIER|RBM23|ENSG00000100461|transcript|ENST00000555722|protein_coding|1/4|c.-284+3568G>A||||||WARNING_TRANSCRIPT_INCOMPLETE,T|intron_variant|MODIFIER|RBM23|ENSG00000100461|transcript|ENST00000553920|processed_transcript|1/4|n.138+3568G>A||||||,T|intron_variant|MODIFIER|RBM23|ENSG00000100461|transcript|ENST00000557227|protein_coding|1/3|c.-306+3568G>A||||||WARNING_TRANSCRIPT_NO_STOP_CODON,T|intron_variant|MODIFIER|RBM23|ENSG00000100461|transcript|ENST00000555714|processed_transcript|1/5|n.151+3568G>A||||||,T|intron_variant|MODIFIER|RBM23|ENSG00000100461|transcript|ENST00000554256|protein_coding|1/5|c.-11+3568G>A||||||WARNING_TRANSCRIPT_NO_STOP_CODON,T|intron_variant|MODIFIER|RBM23|ENSG00000100461|transcript|ENST00000556687|retained_intron|1/3|n.151+3568G>A||||||,T|intron_variant|MODIFIER|RBM23|ENSG00000100461|transcript|ENST00000553902|retained_intron|1/2|n.151+3568G>A||||||,T|intron_variant|MODIFIER|RBM23|ENSG00000100461|transcript|ENST00000554955|retained_intron|1/4|n.112+3568G>A||||||,T|intron_variant|MODIFIER|RBM23|ENSG00000100461|transcript|ENST00000556984|processed_transcript|1/4|n.151+3568G>A||||||,T|intron_variant|MODIFIER|RBM23|ENSG00000100461|transcript|ENST00000557549|protein_coding|1/4|c.-128-3528G>A||||||WARNING_TRANSCRIPT_NO_STOP_CODON,T|intron_variant|MODIFIER|RBM23|ENSG00000100461|transcript|ENST00000555676|protein_coding|1/4|c.-132-1586G>A||||||WARNING_TRANSCRIPT_NO_STOP_CODON,T|intron_variant|MODIFIER|RBM23|ENSG00000100461|transcript|ENST00000557571|protein_coding|2/4|c.-11+1597G>A||||||WARNING_TRANSCRIPT_NO_STOP_CODON,T|intron_variant|MODIFIER|RBM23|ENSG00000100461|transcript|ENST00000556862|protein_coding|1/5|c.-132-1586G>A||||||WARNING_TRANSCRIPT_INCOMPLETE,T|intron_variant|MODIFIER|RBM23|ENSG00000100461|transcript|ENST00000557464|protein_coding|1/4|c.-108-1610G>A||||||WARNING_TRANSCRIPT_NO_STOP_CODON,T|intron_variant|MODIFIER|RBM23|ENSG00000100461|transcript|ENST00000554618|protein_coding|1/5|c.-210-1415G>A||||||WARNING_TRANSCRIPT_INCOMPLETE,T|intron_variant|MODIFIER|RBM23|ENSG00000100461|transcript|ENST00000556365|processed_transcript|1/3|n.151+3568G>A||||||,T|intron_variant|MODIFIER|RBM23|ENSG00000100461|transcript|ENST00000553876|protein_coding|1/2|c.-11+2048G>A||||||WARNING_TRANSCRIPT_NO_STOP_CODON |
| RBM23 | rs178640 | 37 | 14 | 23386360 | 23386360 | + | SNP | A | A | G | SRR8586414 | Translation_Start_Site | 0/1:48,142:190:99:3763,0,1177 | 190;ANN=G|5_prime_UTR_variant|MODIFIER|RBM23|ENSG00000100461|transcript|ENST00000557571|protein_coding|2/5|c.-134T>C|||||5758|WARNING_TRANSCRIPT_NO_STOP_CODON,G|upstream_gene_variant|MODIFIER|PRMT5-AS1|ENSG00000237054|transcript|ENST00000424245|antisense||n.-2305A>G|||||2305|,G|upstream_gene_variant|MODIFIER|PRMT5-AS1|ENSG00000237054|transcript|ENST00000609885|antisense||n.-2470A>G|||||2470|,G|upstream_gene_variant|MODIFIER|PRMT5-AS1|ENSG00000237054|transcript|ENST00000457443|antisense||n.-3488A>G|||||3488|,G|upstream_gene_variant|MODIFIER|PRMT5-AS1|ENSG00000237054|transcript|ENST00000599580|antisense||n.-3760A>G|||||3760|,G|upstream_gene_variant|MODIFIER|PRMT5-AS1|ENSG00000237054|transcript|ENST00000595662|antisense||n.-3887A>G|||||3887|,G|upstream_gene_variant|MODIFIER|PRMT5-AS1|ENSG00000237054|transcript|ENST00000587245|antisense||n.-3887A>G|||||3887|,G|upstream_gene_variant|MODIFIER|PRMT5-AS1|ENSG00000237054|transcript|ENST00000590290|antisense||n.-3890A>G|||||3890|,G|downstream_gene_variant|MODIFIER|PRMT5|ENSG00000100462|transcript|ENST00000324366|protein_coding||c.*3753T>C|||||3360|,G|downstream_gene_variant|MODIFIER|RBM23|ENSG00000100461|transcript|ENST00000553777|processed_transcript||n.*413T>C|||||413|,G|downstream_gene_variant|MODIFIER|PRMT5|ENSG00000100462|transcript|ENST00000397441|protein_coding||c.*3753T>C|||||3374|,G|downstream_gene_variant|MODIFIER|PRMT5|ENSG00000100462|transcript|ENST00000557443|protein_coding||c.*3530T>C|||||3378|WARNING_TRANSCRIPT_NO_START_CODON,G|downstream_gene_variant|MODIFIER|PRMT5|ENSG00000100462|transcript|ENST00000476175|processed_transcript||n.*3378T>C|||||3378|,G|downstream_gene_variant|MODIFIER|PRMT5|ENSG00000100462|transcript|ENST00000397440|protein_coding||c.*3753T>C|||||3378|,G|downstream_gene_variant|MODIFIER|PRMT5|ENSG00000100462|transcript|ENST00000216350|protein_coding||c.*3753T>C|||||3379|,G|downstream_gene_variant|MODIFIER|PRMT5|ENSG00000100462|transcript|ENST00000555454|protein_coding||c.*3896T>C|||||3659|WARNING_TRANSCRIPT_NO_START_CODON,G|downstream_gene_variant|MODIFIER|PRMT5|ENSG00000100462|transcript|ENST00000454731|protein_coding||c.*3753T>C|||||3674|WARNING_TRANSCRIPT_NO_START_CODON,G|downstream_gene_variant|MODIFIER|PRMT5|ENSG00000100462|transcript|ENST00000553915|nonsense_mediated_decay||c.*10445T>C|||||3706|,G|downstream_gene_variant|MODIFIER|PRMT5|ENSG00000100462|transcript|ENST00000538452|protein_coding||c.*3753T>C|||||3706|,G|downstream_gene_variant|MODIFIER|PRMT5|ENSG00000100462|transcript|ENST00000553897|protein_coding||c.*3753T>C|||||3725|,G|downstream_gene_variant|MODIFIER|PRMT5|ENSG00000100462|transcript|ENST00000557758|retained_intron||n.*4981T>C|||||4981|,G|intron_variant|MODIFIER|RBM23|ENSG00000100461|transcript|ENST00000359890|protein_coding|1/13|c.-11+1848T>C||||||,G|intron_variant|MODIFIER|RBM23|ENSG00000100461|transcript|ENST00000555209|protein_coding|1/10|c.-493+1848T>C||||||,G|intron_variant|MODIFIER|RBM23|ENSG00000100461|transcript|ENST00000557667|retained_intron|1/13|n.138+1848T>C||||||,G|intron_variant|MODIFIER|RBM23|ENSG00000100461|transcript|ENST00000399922|protein_coding|1/12|c.-11+1848T>C||||||,G|intron_variant|MODIFIER|RBM23|ENSG00000100461|transcript|ENST00000346528|protein_coding|1/11|c.-11+1848T>C||||||,G|intron_variant|MODIFIER|RBM23|ENSG00000100461|transcript|ENST00000542016|protein_coding|1/11|c.-360+1848T>C||||||,G|intron_variant|MODIFIER|RBM23|ENSG00000100461|transcript|ENST00000307814|retained_intron|1/10|n.151+1848T>C||||||,G|intron_variant|MODIFIER|RBM23|ENSG00000100461|transcript|ENST00000557403|protein_coding|1/5|c.-343+1848T>C||||||WARNING_TRANSCRIPT_INCOMPLETE,G|intron_variant|MODIFIER|RBM23|ENSG00000100461|transcript|ENST00000555722|protein_coding|1/4|c.-284+1848T>C||||||WARNING_TRANSCRIPT_INCOMPLETE,G|intron_variant|MODIFIER|RBM23|ENSG00000100461|transcript|ENST00000553920|processed_transcript|1/4|n.138+1848T>C||||||,G|intron_variant|MODIFIER|RBM23|ENSG00000100461|transcript|ENST00000557227|protein_coding|1/3|c.-306+1848T>C||||||WARNING_TRANSCRIPT_NO_STOP_CODON,G|intron_variant|MODIFIER|RBM23|ENSG00000100461|transcript|ENST00000555714|processed_transcript|1/5|n.151+1848T>C||||||,G|intron_variant|MODIFIER|RBM23|ENSG00000100461|transcript|ENST00000554256|protein_coding|1/5|c.-11+1848T>C||||||WARNING_TRANSCRIPT_NO_STOP_CODON,G|intron_variant|MODIFIER|RBM23|ENSG00000100461|transcript|ENST00000556687|retained_intron|1/3|n.151+1848T>C||||||,G|intron_variant|MODIFIER|RBM23|ENSG00000100461|transcript|ENST00000553902|retained_intron|1/2|n.151+1848T>C||||||,G|intron_variant|MODIFIER|RBM23|ENSG00000100461|transcript|ENST00000554955|retained_intron|1/4|n.112+1848T>C||||||,G|intron_variant|MODIFIER|RBM23|ENSG00000100461|transcript|ENST00000556984|processed_transcript|1/4|n.151+1848T>C||||||,G|intron_variant|MODIFIER|RBM23|ENSG00000100461|transcript|ENST00000557549|protein_coding|1/4|c.-129+1848T>C||||||WARNING_TRANSCRIPT_NO_STOP_CODON,G|intron_variant|MODIFIER|RBM23|ENSG00000100461|transcript|ENST00000555676|protein_coding|1/4|c.-133+1848T>C||||||WARNING_TRANSCRIPT_NO_STOP_CODON,G|intron_variant|MODIFIER|RBM23|ENSG00000100461|transcript|ENST00000556862|protein_coding|1/5|c.-133+1848T>C||||||WARNING_TRANSCRIPT_INCOMPLETE,G|intron_variant|MODIFIER|RBM23|ENSG00000100461|transcript|ENST00000557464|protein_coding|1/4|c.-109+1848T>C||||||WARNING_TRANSCRIPT_NO_STOP_CODON,G|intron_variant|MODIFIER|RBM23|ENSG00000100461|transcript|ENST00000554618|protein_coding|1/5|c.-211+1848T>C||||||WARNING_TRANSCRIPT_INCOMPLETE,G|intron_variant|MODIFIER|RBM23|ENSG00000100461|transcript|ENST00000556365|processed_transcript|1/3|n.151+1848T>C||||||,G|intron_variant|MODIFIER|RBM23|ENSG00000100461|transcript|ENST00000553876|protein_coding|1/2|c.-11+328T>C||||||WARNING_TRANSCRIPT_NO_STOP_CODON |
[truncated: 2,120,454 more chars]
